# ACE-Dependent Alzheimer’s Disease: Blood ACE Phenotyping of the Most Prevalent and Damaging *ACE* Missense Mutation—Y215C (rs3730025)

**DOI:** 10.3390/biomedicines14020275

**Published:** 2026-01-26

**Authors:** Anastasiia A. Buianova, Ivan A. Adzhubei, Olga V. Kryukova, Olga A. Kost, Iaroslav V. Mironenko, Alex S. Kozuch, Galit A. Ilyina, Anna A. Kuznetsova, Zhanna A. Repinskaia, Alexey V. Churov, Steven M. Dudek, Denis V. Rebrikov, Sergei M. Danilov

**Affiliations:** 1 Genomics Laboratory, Pirogov Russian National Research Medical University, 117513 Moscow, Russia; galit.ilyina@gmail.com (G.A.I.); kuznetsova.anna7377@gmail.com (A.A.K.); repinskaia@gmail.com (Z.A.R.); drebrikov@gmail.com (D.V.R.); 2 Department of Biomedical Informatics, Harvard Medical School, Boston, MA 02115, USA; ivan_adzhubey@hms.harvard.edu; 3 Department of Chemistry, M.V. Lomonosov Moscow State University, 119991 Moscow, Russia; so.b11onde@gmail.com (O.V.K.); kost-o@mail.ru (O.A.K.); 4 Institute on Aging Research, Russian Clinical Research Center of Gerontology, Pirogov Russian National Research Medical University, Ministry of Healthcare of the Russian Federation, 129226 Moscow, Russia; jarosmironen@gmail.com (I.V.M.); achurou@yandex.ru (A.V.C.); 5 Department of Medicine, Division of Pulmonary, Critical Care, Sleep and Allergy, University of Illinois at Chicago, Chicago, IL 60612, USA; alexkozuch@gmail.com (A.S.K.); sdudek@uic.edu (S.M.D.)

**Keywords:** angiotensin I-converting enzyme, transport-deficient mutations, plasma ACE levels, ACE shedding, Alzheimer ’s disease, genetic modifiers, protective variants

## Abstract

**Background**: The *ACE* Y215C mutation is a common, functionally damaging missense variant (~1.5% allele frequency) associated with reduced plasma ACE levels and increased Alzheimer’s disease (AD) risk. In CHO and HEK cell models, this mutation caused a ~3–6-fold decrease in ACE surface expression, soluble ACE levels, and ACE enzymatic activity compared to those of wild-type ACE. **Methods**: Circulating ACE levels and activity were measured in EDTA plasma obtained from 84 carriers of the *ACE* Y215C mutation using a set of mAbs to the ACE. The mAbs 5B3/1G12 binding ratio was revealed as a sensitive marker for the circulating Y215C ACE mutant. Whole-exome and whole-genome sequencing (WES/WGS) were performed to identify genetic variants potentially modifying circulating ACE levels. In parallel, published sequencing and proteomic data from 35,559 Icelanders participants were analyzed to identify genes influencing ACE shedding. Sequence comparison was performed between carriers with elevated and reduced ACE concentrations to identify the potential protective variants that may compensate for decreased ACE levels due to the Y215C mutation itself. **Results**: Most carriers of the Y215C *ACE* mutation demonstrated significantly decreased ACE levels (median is 62% of control ACE levels). However, substantial inter-individual variability was observed in plasma ACE activity among carriers. Comparative sequencing analysis revealed 9648 variants unique to individuals with elevated ACE, mapping to 5779 protein-coding genes and enriched for pathways related to intracellular and transmembrane transport. **Conclusions**: The presence of the damaging *ACE* mutation Y215C does not invariably result in low plasma ACE or, likely, elevated AD risk. Therefore, combined blood ACE phenotyping and whole-exome sequencing are recommended to more accurately assess ACE-related AD susceptibility in mutation carriers.

## 1. Introduction

Late-onset Alzheimer’s disease (AD) is a multifactorial neurodegenerative disorder influenced by *APOE* and numerous additional genetic risk loci. It is characterized by progressive cognitive decline and may include neuropsychiatric symptoms such as anxiety, depression, and apathy, which can progress to agitation, aggression, and hallucinations in later stages [1]. Large-scale genomic studies have identified more than 30 [2] and possibly up to 75 [3] independent susceptibility regions implicated in amyloid processing, endosomal trafficking, lipid metabolism, immune signaling [4], and other pathways relevant to AD pathogenesis. Among these, the angiotensin-converting enzyme (ACE; CD143, EC 3.4.15.1) gene has repeatedly been associated with AD risk [2,5,6,7,8]. In addition to genetic predisposition, lifestyle and comorbid conditions—including type 2 diabetes, cardiovascular disease, sleep disturbances, poor diet, chronic inflammation, infections, and low social engagement—modulate the risk and progression of AD [1].

We recently proposed that individuals carrying heterozygous loss-of-function (LoF) *ACE* mutations accompanied by low ACE levels may represent a previously underrecognized high-risk subgroup for late-onset AD. More than 1200 *ACE* variants have been reported, of which >400 are predicted to be damaging by in silico analyses. The combined population frequency of these LoF-predicted *ACE* mutations reaches 5%, comparable to the prevalence of AD in adults older than 70 years. This suggests that the genetically reduced ACE expression to AD risk may be substantially underestimated [9].

The biological connection between ACE dysfunction and AD is mechanistically plausible. ACE has been shown to degrade amyloid-β, the principal component of senile plaques, both in vitro [10] and in mammalian cell lines transfected with human cDNAs encoding amyloid precursor protein (APP) and ACE [11]. The N-terminal domain of ACE, via its catalytic active site, is considered the principal site mediating Aβ42 degradation [12]. Accordingly, reduced ACE activity is expected to promote Aβ42 accumulation, consistent with experimental findings and several independent clinical and genetic datasets [13,14]. Additional mechanisms linking ACE deficiency to AD pathogenesis have also been proposed [9].

The aim of the present study was to characterize the blood ACE phenotype in carriers of the most prevalent and AD-associated *ACE* mutation, Y215C [2,9]. This variant produces a profound reduction in plasma ACE levels, serving as a surrogate marker of diminished surface ACE expression [15,16,17,18,19]. To delineate its functional consequences, we recently used plasmids encoding mutant ACE to establish cell-based models of Y215C transfecting human embryonic kidney (HEK) cells, human neuronal cell line SH-SY5Y, and chinese hamster ovary (CHO) cells. We then systematically evaluated the functional consequences of the Y215C substitution on both membrane-bound and soluble ACE, including analysis of the catalytic properties and conformational epitopes using a panel of monoclonal antibodies (mAbs) together with two ACE-specific substrates. Across all model systems, the Y215C mutation caused a profound loss of surface ACE expression—approximately a six-fold reduction in HEK293 and CHO cells—and a substantial, though less pronounced, decrease in SH-SY5Y cells [20].

In the present work, we measured plasma ACE levels in 84 carriers of the Y215C *ACE* mutation. Although the group exhibited significantly reduced ACE levels, we observed marked inter-individual heterogeneity, raising concerns regarding the interpretability of plasma ACE measurements alone. It is increasingly evident that the interpretation of blood ACE levels requires parallel information on genetic variants in other loci that modulate ACE shedding. Without such data, plasma ACE measurements may be misleading—yielding both false-negative and false-positive assessments of ACE deficiency. To address this, we screened for known mutations influencing ACE secretion (“Iceland’s list”), based on proteogenomic data from 35,559 sequenced Icelanders [21], and evaluated their presence in the cohort of 84 Y215C carriers. The key clinical implication is that the Y215C mutation alone does not ensure low ACE expression and therefore does not uniformly confer increased susceptibility to ACE-related AD. Comprehensive phenotyping of plasma ACE together with whole-exome and whole-genome sequencing (WES/WGS) is essential to avoid the misclassification of risk in individuals carrying this *ACE* mutation.

Finally, because Y215C is likely a transport-deficient mutation, carriers may benefit from therapeutic strategies aimed at restoring proper ACE trafficking. Pharmacological rescue using a combination of chemical and pharmacological chaperones and proteasome inhibitors has previously been shown to correct the surface expression of another transport-deficient *ACE* variant (Q1069R) causal for renal tubular dysgenesis [22], suggesting a novel and potentially clinically important avenue for the targeted prevention or early-stage intervention in *ACE* mutation Y215C carriers.

## 2. Materials and Methods

### 2.1. Study Participants

Blood ACE phenotyping was performed in 84 carriers of the *ACE* Y215C mutation and control subjects. Blood samples were obtained primarily from previously sequenced individuals (WES/WGS) from the Center for Precision Genome Editing and Genetic Technologies for Biomedicine (*n* = 72) and the Institute on Aging Research, Russian Gerontology Research and Clinical Center (*n* = 12), both affiliated with Pirogov Russian National Research Medical University (Moscow, Russia). Some values of blood ACE for the carriers of Y215C mutations were taken from previously published studies [15,16,17,18,19]. The cohorts from the Institute on Aging Research were described in more detail in a previous study from the ACE-dependent AD research series [19]. The collection of human blood samples and the protocol for the whole-exome screening of tested individuals was reviewed and approved by the Ethics Committee of the Kulakov National Medical Research Center for Obstetrics, Gynecology and Perinatology (protocol no. 9 from 22 October 2020). All corresponding procedures were carried out in accordance with institutional guidelines and the Code of Ethics of the World Medical Association (Declaration of Helsinki). All subjects gave written informed consent for the use of any data for scientific purposes. The entire study included EDTA plasma samples from 84 subjects with the Y215C *ACE* mutation, and 129 controls (without non-synonymous *ACE* mutations).

### 2.2. WES and WGS

DNA was isolated from all blood samples. The isolation of genomic DNA, the evaluation of its quality, preparation of a DNA library, pre-capture sample pooling for enrichment following the “RSMU exome” protocol, and sequencing of samples were carried out as described in recent studies [16,23]. WGS was performed for samples from the Institute on Aging Research. The WGS protocol was previously described in [19].

### 2.3. Chemicals

ACE substrates, benzyloxycarbonyl-L-phenylalanyl-L-histidyl-L-leucine (ZPHL) and hippuryl-L-histidyl-L-leucine (HHL) were purchased from Bachem Bioscience Inc. (King of Prussia, PA, USA) and Sigma-Aldrich (St. Louis, MO, USA). Other reagents (unless otherwise indicated) were obtained from Sigma-Aldrich (St. Louis, MO, USA).

### 2.4. Immunological Estimation of the Levels of Blood ACE

ACE levels were determined in EDTA plasma from carriers of the Y215C mutation, as well as controls, using an antibody-based fluorometric assay with two ACE substrates, 2 mM ZPHL or 5 mM HHL [16,24,25].

### 2.5. Identification of the Genetic Variants That Influence Blood ACE Levels

Plasma protein levels were measured with SomaScan multiplex aptamer assay for 4907 proteins, including ACE, in 35,559 Icelanders with genotype and phenotype information [21]. Authors of this paper (and deCODE genetics, Reykjavík, Iceland/Amgen, Inc., Thousand Oaks, CA, USA) made available to us the full GWAS summary statistics for each protein from this study including ACE (https://download.decode.is/form/folder/proteomics, accessed on 20 October 2022).

The initial dataset was derived from the Icelandic population dataset, containing over 24 million genetic variants that demonstrated correlation of blood ACE levels. Due to computational and resource limitations, we extracted a subset of 1,498,823 variants which met a criterion of statistical significance, defined as *p*-value < 0.05. This extraction aimed to reduce the dataset size to a manageable level while retaining potentially meaningful associations. The filtered variants were distributed as follows: chromosomes 1–11: 1,034,999 variants; chromosomes 12–22: 458,654 variants; chromosome X: 5170 variants. Following this initial extraction, we performed additional filtering to isolate only the variants related to protein coding mutations. In addition, we applied additional filtering to identify the variants which significantly correlated with blood ACE. We choose the threshold of beta value of equal or higher than 0.350 in this module. Such beta value was found for blood group antigen variants, in which blood ACE differences could be easily measured using a conventional blood ACE activity assay [25].

We excerpted these GWAS statistics for ACE (i.e., correlation of blood ACE levels with all genetic variants found in 35,500+ Icelanders), which resulted in 2388 variants selected using a nominal significance threshold of *p*-value < 0.05, without correction for multiple testing, to allow inclusion of variants with potentially large effects (Table S1).

### 2.6. Annotating Genetic Variation in ACE Protein Found in UK Biobank (UKB)

Complete GWAS summary statistics data for the ACE pQTL Inflammation II panel [26] were downloaded from the UKB repository. All variants on chromosome 17 with *p*-value < 0.05 were then annotated using Ensembl Variant Effect Predictor (Gencode release 49/Ensembl v115.2) and GRCh37 genome assembly. Coding sequence variants were then mapped onto the ENST00000290866 transcript (MANE Select; UniProt accession: P12821). Population frequency data and scores for several computational methods for predicting variant effects were extracted using dbNSFP version 5.3a as a source [27].

### 2.7. Search of Protective Variants

The GWAS aimed at identifying putative protective variants was performed using PLINK v1.90b6.21 [28]. Prior to association testing, variants were restricted to exonic regions by intersecting the genotype data with a BED file comprising genomic intervals with sufficient sequencing coverage (Q90 > 13) across SureSelect Human All Exon v6, v7, v8 probes (Agilent Technologies, Santa Clara, CA, USA). This step was implemented to minimize batch effects related to differences between genomic and exome data. In addition, regions known to be prone to technical artifacts were inspected based on a curated blacklist [29]. A single quality control filter was applied at the variant level, excluding markers with a genotyping rate below 95% (*--geno 0.05*). After filtering, 33,378 variants and 59 individuals were retained for downstream analyses, with an overall genotyping rate of 97.98%. Among the included individuals, 44 were classified as cases and 15 as controls. To focus on common genetic variation, an additional filtering step based on minor allele frequency ≥ 5% was applied, which reduced the number of variants to 30,130. Allelic association testing was conducted using logistic regression under an additive genetic model. Association statistics were computed using the *--assoc* and *--assoc logistic* options in PLINK. Genomic inflation factors (λ) were estimated based on the median chi-square statistic and indicated minimal inflation (λ = 1.01 for the allelic test and λ = 1.02 for the logistic model). Adjustment for multiple testing was performed as implemented in PLINK (*--adjust*). Functional annotation of associated variants was performed using Ensembl Variant Effect Predictor (VEP) v113 [30].

### 2.8. Statistical Analysis

Values of ACE activity measured with different substrates for each individual, as well as other parameters characterizing ACE phenotype, are expressed as means ± SD from at least 3 independent experiments with duplicates. Significance was analyzed using the Mann–Whitney test.

### 2.9. Use of Generative Artificial Intelligence (AI)

A generative AI system (Grok 4, xAI) was employed as an auxiliary tool to assist in prioritizing candidate metalloproteases potentially involved in ACE ectodomain shedding. Protein names from a predefined list of genes with negative beta values were provided to the AI, which ranked candidates based on known principles of protease biochemistry, cellular localization, and relevance to ACE biology. The AI output was used solely for hypothesis generation; it did not generate new experimental data or replace author-driven analyses. All final candidate selection, interpretation, and biological justification were performed entirely by the authors, and each literature reference suggested by the AI was manually verified to ensure accuracy and relevance. This approach minimized potential bias from AI-assisted ranking while leveraging its capability to integrate complex information.

## 3. Results and Discussion

### 3.1. Quantification of Blood ACE in Carriers of the Y215C ACE Mutation

Previously, we have established an approach for the comprehensive characterization of ACE in the blood (blood ACE phenotyping). This approach includes not only measurement of ACE activity in serum or plasma (heparinized or citrated), but also quantification of immunoreactive ACE protein, as well as the detection of putative conformational changes in a patient’s blood ACE using a set of mAbs to different epitopes on the surface of the ACE globule [24,31,32,33,34]. Unfortunately, most sequencing facilities operate only with EDTA-containing plasma, which makes it impossible to measure ACE activity directly, due to the extraction of the zinc ion from the active centers of the enzyme by the chelating agent. However, ACE levels in EDTA-containing plasma can be estimated by the precipitation of ACE with mAbs to native ACE and the subsequent detection of precipitated ACE activity. Using this method, we have already estimated blood ACE levels in more than 300 carriers of over 50 *ACE* mutations, as well as in over 300 controls, which has given us the ability to assess the impact of these mutations on ACE functions [16,17,18,19]. Moreover, we demonstrated in cell culture models that the Y215C *ACE* mutation could dramatically (3–6-fold) decrease the surface expression of this mutant ACE [20].

Here, we estimated plasma ACE levels in 84 carriers of the Y215C *ACE* mutation, which were identified in several cohorts. Most were found in the collection of the Center for Precision Genome Editing and Genetic Technologies for Biomedicine in Pirogov Russian National Research Medical University (Moscow), and in 129 control plasma samples from the same collection (patients without missense, non-synonymous *ACE* mutations).

**Figure 1:**
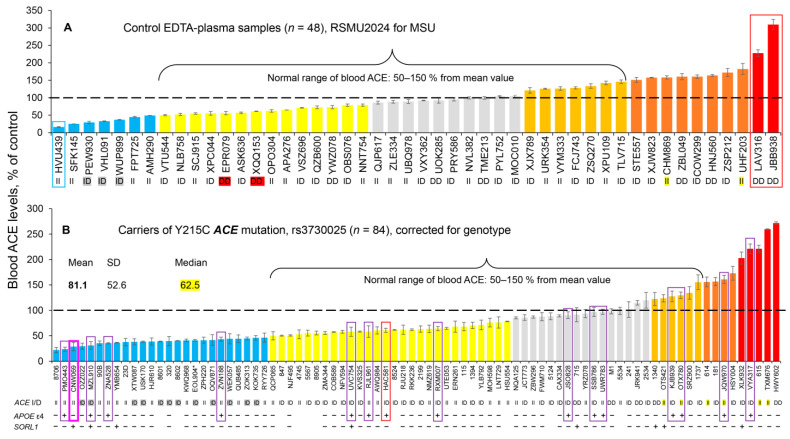
**Quantification of blood ACE levels in carriers of controls and the Y215C *ACE* mutation**. Blood ACE protein was precipitated from EDTA plasma by mAb 9B9, and its activity was then quantified fluorometrically using ZPHL as a substrate. (**A**) The immunoreactive ACE protein was quantified in plasma samples from 48 controls. (**B**) The immunoreactive ACE protein—from 84 carriers of the Y215C *ACE* mutation corrected for genotype [16,19]. ACE levels were presented as the means ± SD of several independent assessments of samples. The data are expressed as % of ACE levels of the corresponding value for control pooled plasma samples from donors without *ACE* mutations. Orange, brown, and red bars indicate samples with ACE levels > 120%, 150%, and 200% of controls, respectively. Yellow and blue bars—samples with ACE levels < 80% and 50% of controls, respectively. Gray bars—ACE levels between 80% and 120% of the control values. The dashed line marks the 100% control baseline.

ACE levels in EDTA-containing plasma samples were determined with mAb 9B9 from the first control set with 48 samples (without missense *ACE* mutations) and then expressed as a percentage (Figure 1A) of the mean blood ACE levels in subjects who were in the normal range, that is 50–150% of a mean based on testing 300 unrelated healthy volunteers [15].

As expected, most of the control samples from this set were in this normal range of blood ACE levels, while some were obvious outliers—with decreased (left blue bars and, especially, blue boxed) or increased (right brown bars, red bars, and, especially, red boxed) blood ACE levels.

Initially, the blood ACE levels measured in the 84 carriers of the Y215C *ACE* mutation were also expressed as a % of the mean ACE levels (as in Figure 1A). However, an exact (quantitative) estimation of the effect of the Y215C *ACE* mutation on blood ACE levels in each individual was deemed inaccurate without also knowing the *ACE* genotype of the I/D polymorphism, because both blood and tissue ACE levels are well-known to be influenced by this *ACE* genotype [24,35,36,37,38,39]. Blood ACE levels in carriers of the DD genotype were reported to be 66% higher than in carriers of the II genotype [24,40]. Therefore, ACE levels in blood samples from carriers with the Y215C mutation were corrected according to the *ACE* genotype for each individual (Figure 1B), as we have previously described [16,17,18,19].

We again demonstrated (but now with more robust statistics) that plasma ACE levels in most patients with the Y215C mutation (rs3730025) were remarkably decreased. The median was 62% of the mean level in the population (Figure 1B). This confirms our previous results that the Y215C mutant allele is weakly functional [20], and it indicates that this relatively frequent (~1.5%) and AD-associated *ACE* mutation [2,6] in the N domain of ACE (i.e., Y215C) is truly damaging [15,16,17] and likely transport-deficient. Thus, the strong and statistically causative association of this *ACE* mutation with AD [2] may be quite direct—simply due to a significant decrease in ACE expression on the cell surface because of transport deficiency (similar to what we previously found with mutation Q1069R [22]).

The distribution of the Y215C variant across various populations demonstrates pronounced population heterogeneity. This variant occurs predominantly in individuals of European ancestry (~1400 per 100,000), whereas it is extremely rare in Asian, African, and Latin American cohorts (Table 1).

Such differences likely reflect evolutionary processes—including isolation, genetic drift, mutational dynamics, assortative mating, migration, and possible selective effects—and highlight that the functional and clinical consequences of Y215C is likely to be most clinically relevant in European cohorts, where the carrier frequency is sufficient for statistically robust evaluation.

In large cohorts (Icelanders [21], UK Biobank [26], and several Russian datasets), a range between dozens and several hundred carriers of the Y215C mutation have been identified, corresponding to a frequency of approximately 1000–2500 per 100,000 individuals (with the highest value of 2571 in the FMBA cohort [41]). Such population differences in allele frequency may theoretically influence the overall genetic contribution of *ACE* to AD risk, although this relationship requires further epidemiological validation. Since the Y215C variant is likely associated with the impaired trafficking of ACE to the cell surface [20], these carriers represent a potential target group (thousands of already identified individuals in mentioned cohorts) for testing therapeutic rescue-of-impaired-traffic strategies, similar to those described by Danilov et al. [22]. This approach is particularly relevant in the context of evidence linking ACE dysfunction with the risk and progression of AD.

**Figure 2:**
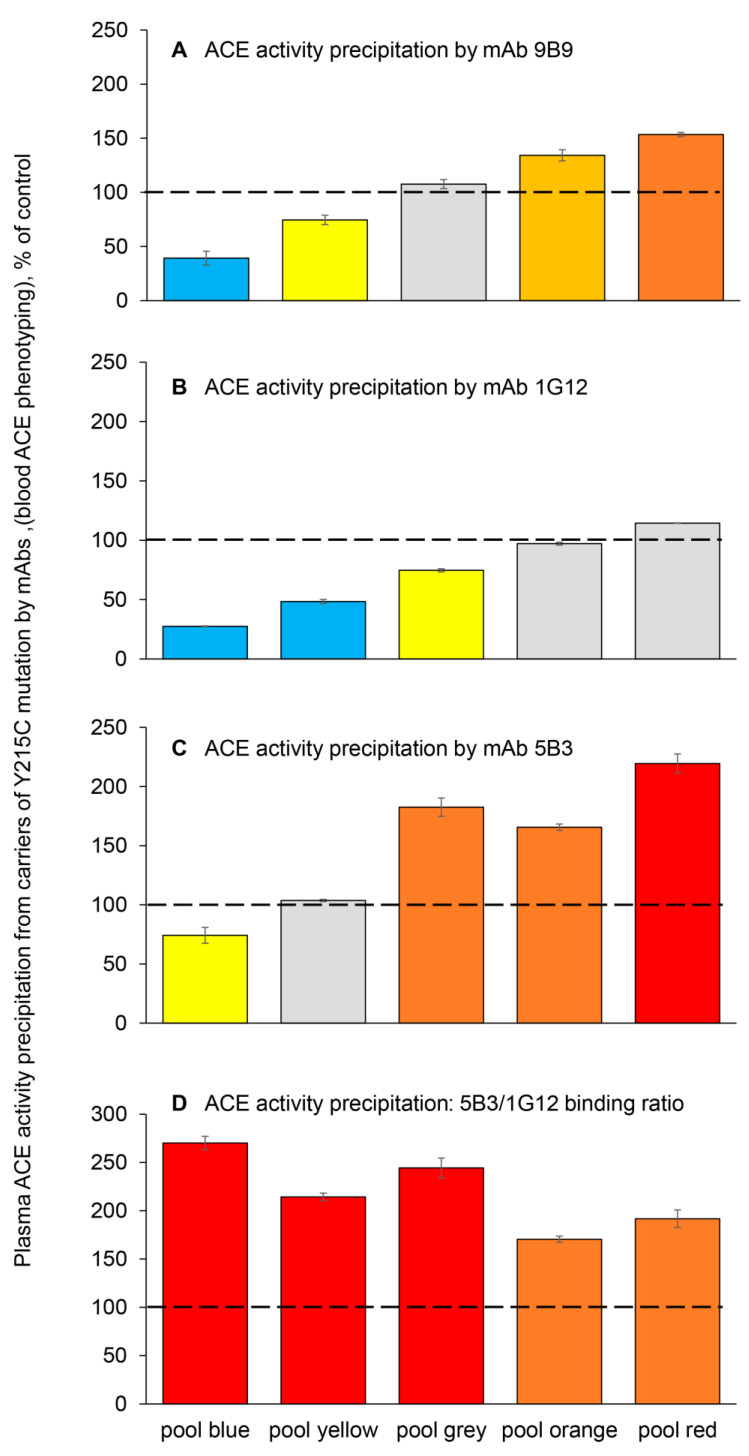
**ACE precipitation by mAbs from the EDTA plasma pools obtained from Y215C *ACE* mutation carriers.** Blood ACE protein was precipitated using 3 mAbs targeting the N domain (9B9, 1G12, and 5B3). Plasma pools were combined according to the data shown in Figure 1A. Samples with ACE levels less than 80% and less than 50% of the control were combined into the blue and yellow pools, respectively. Pools with ACE levels near 100% were combined into the gray pool, and samples with levels greater than 120% and greater than 200% of the control were combined into the orange and red pools, respectively. (**A**) Precipitated ACE activity by mAb 9B9, and its activity, was then quantified fluorometrically using ZPHL as a substrate. (**B**) Precipitated ACE activity by mAb 1G12, the same substrate. (**C**) Precipitated ACE activity by mAb 5B3, the same substrate. (**D**) 5B3/1G12 binding ratio. Coloring is as described in the legend to Figure 1. The dashed line marks the 100% control baseline.

A future goal will be to organize a limited clinical trial using a cocktail of chemical and pharmacological chaperones and proteosome inhibitors (similar to what was effective in rescue of impaired traffic of another transport-deficient *ACE* mutation—Q1098R [22]), or other potential candidates revealed using cell models of this Y215C mutation, in HEK cells [20]. To conduct such a clinical trial, we would need a tool for the quantitative measurement of mutant (Y215C) ACE in the plasma/serum samples from volunteer carriers of the Y215C mutation after taking such a cocktail over a period of time (from one week to one month). We already have found that the 2H9/1G12 binding ratio was increased (by ~50%) in heterozygous carriers of the Y215C mutation, as previously shown in Figure 3 in [16]. However, this analysis was limited by a small number of samples. More recently, we have found that soluble Y215C mutant ACE obtained from HEK cells transfected with a plasmid encoding this mutant had significantly decreased binding to mAbs having overlapping epitopes, i1A8 and 3G8 [20]. This possibly could be due to the differences in the glycosylation of Asn82, which is in the center of the epitopes for these mAbs [42]. However, these mAbs are not very useful for the precipitation of soluble ACE from plasma/serum because the sialylation of circulating ACE in the blood (and, in particular, in Asn82) dramatically decreased the precipitation of circulating ACE by mAbs i1A8 and 3G8 [42]. Recently, however, we generated a novel mAb to ACE, 5B3, whose epitope overlaps with those for mAbs i1A8/3G8, but the extent of ACE precipitation by this mAb was not influenced by the extensive sialylation of circulating ACE [33]. Thus, to enable a more robust investigation, we assembled a larger cohort of samples carrying the Y215C *ACE* mutation. Based on donor blood ACE levels (Figure 1A), we created five distinct EDTA plasma pools marked by different colors in Figure 2: blue (*n* = 19) with ACE levels < 50% of control; yellow (*n* = 20) with ACE levels 50–80% of control; gray (*n* = 10) with ACE levels comparable to control; orange (*n* = 6) with ACE levels 120–200% of control; red (*n* = 4) with ACE levels ~200% of control. We evaluated the binding of three mAbs whose epitopes are located on the N domain of ACE to EDTA plasma ACE. First, mAb 9B9 was used to confirm the relative ACE concentration in each pool. Although the red pool showed a lower-than-expected level, potentially due to prolonged plasma storage, it still exhibited the highest concentration among all pools (Figure 2A). The binding of mAbs 1G12 and 5B3 varied across the pools, with mAb 1G12 binding increasing according to the ACE level (Figure 2B). In contrast, mAb 5B3 binding was elevated for pools with normal and high ACE levels (gray, orange, red), was normal for the yellow pool, and was reduced for the blue pool (Figure 2C).

The key finding was that the 5B3/1G12 binding ratio was markedly elevated (170–270% of control) for all Y215C pools, almost regardless of ACE levels (Figure 2D). Thus, the 5B3/1G12 binding ratio is an excellent marker of Y215C mutant ACE, because it is very sensitive to the presence of this circulating mutant even in a mixture with native ACE from plasma samples of heterozygous carriers of this mutation (Figure 2).

Intuitively, carriers with the lowest levels of blood ACE (reflecting lowest tissue ACE, left part of Figure 1B) should be at higher risk of ACE-dependent AD. Within this cluster, the single Y215C homozygote (highlighted by the bold magenta box) is expected to exhibit the most pronounced functional deficit and therefore the greatest vulnerability.

However, we know that some other factors can also increase susceptibility to AD. A notable example is the epsilon4 isoform of Apolipoprotein E (apoE4)—Doc S1. In our cohort of 84 participants, 15 individuals (17.7%) carry the *APOE* ε4 allele, a key genetic risk factor for AD: 1 (1.2%) appeared to be homozygous (ε4/ε4), while the remaining 14 (16.7%, shown in boxes) were heterozygous (ε2/ε4 or ε3/ε4), which may be associated with increased risk for AD development. The majority (69.1%) had the neutral ε3/ε3 genotype and ε2/ε3 genotype.

Genetic background further modifies this risk. *APOE* ε4 carriers (purple boxes in Figure 1B), and particularly the ε4/ε4 homozygote (red box), would be expected to have additively increased susceptibility to AD. Moreover, several individuals also harbor variants in *SORL1*, a key regulator of APP trafficking implicated in AD pathogenesis [43]. Thus, Y215C carriers who additionally possess *SORL1* risk variants constitute another biologically plausible high-risk subgroup. Together, these annotations in Figure 1B illustrate how plasma ACE levels, Y215C genotype, *APOE* ε4 status, and *SORL1* variation may interact to generate substantially different risk profiles among individuals carrying the same primary *ACE* mutation.

### 3.2. Identification of Possible False-Negative or False-Positive Alterations in Blood ACE Levels

In this and previous studies [16,17,18,19], we found that the blood ACE level was substantially reduced in a significant proportion of carriers of the Y215C *ACE* mutation (Figure 1A,B). Thus, these carriers may be at increased risk for AD.

Initially, we considered the carriers of this *ACE* mutation as having an increased risk of ACE-dependent AD based on the measured blood ACE levels (Figure 1A,B). Generally, blood ACE levels correctly reflect tissue ACE expression [36,37, own unpublished data]. However, it is possible that some carriers of the Y215C *ACE* mutation from Figure 1B may also have mutations in other genes that alter ACE expression or ACE shedding. For example, plasma ACE2 levels measured in 2248 participants exhibited associations with 1011 other plasma proteins in a recent study [44]. The results of our current study on 84 carriers of the Y215C *ACE* mutation demonstrate dramatically decreased ACE levels in a significant (63%) portion of this subject group (30% blue and 33% yellow bars in Figure 1B), but not in all of them.

Potential reasons for this heterogeneity could be an increased expression of ACE, or an increased shedding of ACE, in some carriers of the Y215C mutation (up to 37% in the group). Therefore, to more rigorously consider whether carriers of this (or other) damaging *ACE* mutation are at an increased risk for ACE-dependent AD, it is necessary to accurately determine their blood ACE levels, which normally should reflect tissue ACE levels. It is also important to consider the possible effects of mutations in other genes that may change the rate of ACE shedding or ACE expression in these subjects to help exclude the false-negative or false-positive effects of such mutations on measured blood ACE levels.

How can we identify these gene-modifiers that change the rate of ACE shedding or ACE expression? Researchers from deCODE genetics (Iceland) sequenced the genomes of 35,559 Icelanders and measured the concentration of 4900+ analytes (including ACE) in their blood using SomaScan (aptamer) technology [21]. These investigators provided us with access to a file correlating blood ACE levels with mutations in all 20,000+ genes of the human genome. We correlated blood ACE levels with mutations in the *ACE* gene in these 35,559 Icelanders and identified 23 *ACE* mutations that influenced blood ACE levels (cis-pQTL) (Table S4 in [9]). We have validated this prediction for at least one *ACE* mutation, Y215C. Carriers of this *ACE* mutation, which has a beta value (degree of correlation) of −1.217 (Table S4 in [9]), reproducibly exhibit significantly decreased blood ACE levels in multiple analyses [15,16,17,18,19] and this study—Figure 1.

Note that Table S4 in [9] (which correlates blood ACE levels with *ACE* mutations) includes another *ACE* mutation, the intronic variant rs367727597, with nearly identical allele frequencies observed in the Icelandic cohort (543 carriers of rs367727597 and 542 carriers of rs3730025 among 35,559 individuals), and nearly identical correlation (beta value) −1.216 and *p*-values. Independent data from the Center for Strategic Planning of the FMBA of Russia confirmed strong linkage disequilibrium (LD) between the intronic variant rs367727597 (c.417+414del, intron 2) and the missense mutation rs3730025 (c.731A>C, exon 5), according to transcript NM_000789.4. Carrier frequency among more than 120,000 Russian individuals was 2.584% for rs367727597 and 2.577% for rs3730025 (database of FMBA) [41]. This indicates that the intronic variant rs367727597 (tagging SNP) is a reliable proxy SNP for detecting the functional missense variant rs3730025. The observed LD reflects their co-inheritance within a shared haplotype block. A haplotype denotes a set of neighboring alleles located on the same chromosome which are inherited together across generations [45,46]. Although the physical distance between rs367727597 and rs3730025 is approximately 1.9 kilobases, meiotic recombination within this segment appears to be limited, facilitating the long-term preservation of their linkage.

In addition to the correlation of blood ACE levels with some *ACE* mutations (Table S4 in [9] this “Icelander’s file” from [19]), these data demonstrated a correlation of blood ACE levels with millions of other mutations from thousands of genes. We extracted 1.5 million different mutations from these genes (from the “Icelander’s” file [21]) which showed a significant correlation (*p* < 0.05) with blood ACE levels. Then, we further extracted 7418 missense mutations from this group, which revealed hundreds of genes (or gene families) containing mutations that unexpectedly influenced blood ACE levels. We next filtered this list of 7418 missense mutations (and additional stop-gained and indels mutations) to retain only those variants which showed a significant influence on blood ACE levels. We arbitrarily selected beta values for three missense mutations in the blood group gene—*ABO* (which definitively influence blood ACE levels [25] by more than 0.350 or less than minus 0.350). This analysis resulted in 2388 variants in 2051 genes which are predicted to influence blood ACE levels (Table S1). This list contains more than 90 gene families that influence blood ACE levels significantly and have more than two mutations for each gene. These genes belong to several categories (Table S2). In terms of broad proteome-wide associations, plasma ACE in this study (similar to plasma ACE2 [44]) was associated with various canonical pathways, including clathrin-mediated endocytosis signaling, actin cytoskeleton signaling, and the protein ubiquitination pathway (Table S2).

We had hoped that the putative ACE secretase [47,48,49,50,51] would be revealed within this list of 2388 variants, but this was not clearly evident. We did not identify a definitive metalloprotease gene because there were several candidates with mutations that significantly correlated with blood ACE levels. An intensive search for the putative ACE secretase(s) was performed in the 1990s reviewed in [50], but this work did not lead to discovery of the ACE secretase—described in Doc S2.

**Figure 3:**
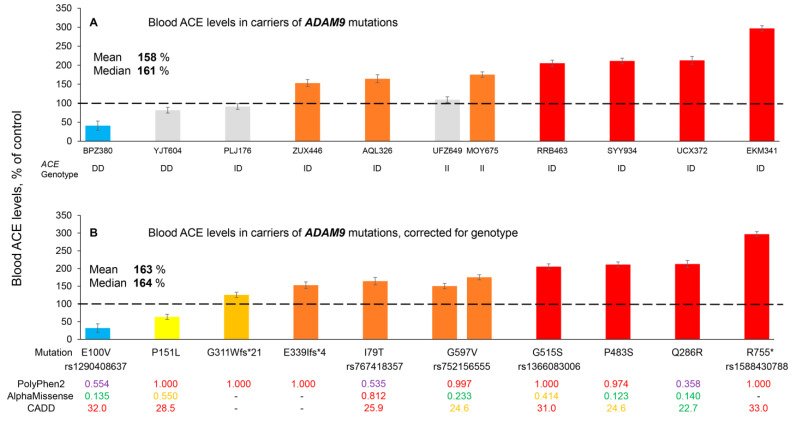
**Quantification of blood ACE levels in carriers of the *ADAM9* mutations.** (**A**) The immunoreactive ACE protein was performed in plasma samples from 11 carriers of 10 different mutations in *ADAM9*. (**B**) Plasma ACE levels adjusted according to the donor’s genotype for the I/D polymorphism ACE levels were presented as the means ± SD of several independent assessments of samples. The data are expressed as % of ACE levels of the corresponding value for control pooled plasma samples from donors without *ACE* mutations. Bottom: Predictions of the potential damaging effects of mutations on the ACE protein were derived from Table S1 [16,19] and based on Poly-Phen2, AlphaMissense, and CADD. For AlphaMissense and CADD: pathogenic predictions are shown in red, uncertain significance in yellow, and benign predictions in green. For Poly-Phen-2 specifically: purple is used for both the ‘possibly damaging’ (score 0.446–0.908) and ‘benign’ (score ≤ 0.445) categories, while ‘probably damaging’ (score ≥ 0.909) is shown in red. Coloring is as described in the legend to Figure 1. The dashed line marks the 100% control baseline.

However, we did identify in Table S1 one mutation in the *ADAM9* gene, which is a protein previously associated with ACE shedding [52]. This is a deletion of an amino acid (Phe) in the 704 position that is in a transmembrane domain of *ADAM9* (rs746548581) and was negatively correlated with blood ACE levels (beta value of −0.691) (Table S1). We next performed blood ACE phenotyping in plasma samples obtained from all available carriers of *ADAM9* mutations in the cohort of 5000+ sequenced subjects of the RSMU collection (Figure 3). The blood ACE levels measured in 11 carriers of 10 different *ADAM9* mutations are shown in Figure 3A, and their values corrected for the *ACE* genotype [19] are shown in Figure 3B. These results indicate that two mutations, E100V and P151L, are associated with significantly decreased blood ACE levels (left side of Figure 3B), while the other eight mutations of *ADAM9* significantly increase blood ACE levels (right side of Figure 3B). Regarding the hypothesis that ADAM9 may be one of the unknown ACE secretases, these results could indicate that E100V and P151L are LoF *ADAM9* mutations, while the other eight mutations are GoF *ADAM9* mutations. However, this concept seems too simplistic. Without knowing how these mutations affect ADAM9 activity, trafficking, turnover, etc., it is difficult to understand the molecular mechanisms involved. Unfortunately, the presence of variants in the carriers of tested *ADAM9* mutations in the Icelandic cohort (Table S4) could not explain the heterogeneity of blood ACE levels in these carriers. In addition, ADAM9 has been implicated in ACE cleavage under inflammatory but not constitutive conditions [52]. Whether ADAM9 can directly cleave ACE, or has an indirect role similar to ADAM17 [53], requires further investigation. This is particularly important given that ADAM9 can itself cleave and regulate ADAM10 and ADAM17 in a more complex protease web [54]. Although early biochemical and antisense-based studies demonstrated that ADAM10 and ADAM17 are not universally required for ACE shedding [55], more recent work in primary human endothelial systems has shown that ADAM10 can act as a major inducible ACE sheddase in a tissue- and stimulus-dependent manner [56]. These findings highlight that ACE ectodomain shedding is highly cell type-, context-, and species-specific, and likely mediated by multiple metalloproteases rather than a single dedicated enzyme [49].

**Figure 4:**
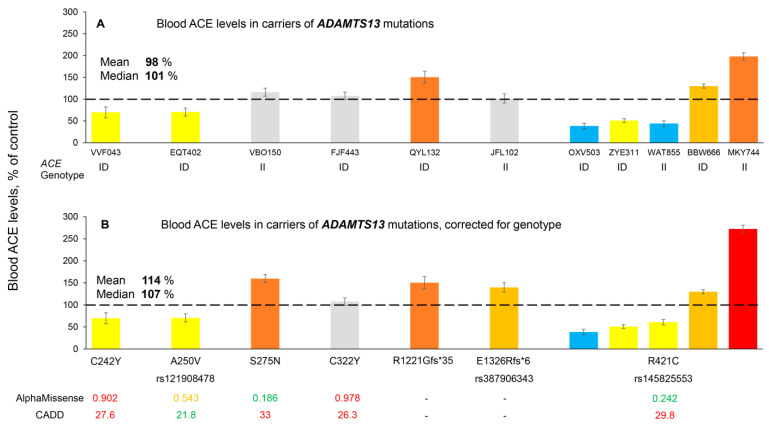
**Quantification of blood ACE levels in carriers of the *ADAMTS13* mutations.** (**A**) The immunoreactive ACE protein was quantified in plasma samples from 11 carriers of 7 different mutation in *ADAMTS13*. (**B**) Plasma ACE levels adjusted according to the donor’s genotype for the I/D polymorphism ACE levels were presented as the means ± SD of several independent assessments of samples. The data are expressed as % of ACE levels of the corresponding value for control pooled plasma samples from donors without *ACE* mutations. Bottom: Predictions of the potential damaging effects of mutations on the ACE protein were derived from Table S1 [16,19] and based on AlphaMissense and CADD. Pathogenic predictions are shown in red, uncertain significance in yellow, and benign predictions in green. Coloring is as described in the legend to Figure 1. The dashed line marks the 100% control baseline.

ADAMTS13 is another metalloprotease in the list of putative ACE secretases (Table S3). The inclusion of ADAMTS13 as a putative ACE secretase (Table S2) was initially suggested by personal unpublished observations of Dr. Valur Emilsson (Iceland). He observed that blood ACE levels were positively (0.246) correlated with blood ADAMTS13 levels, i.e., more ADAMTS13 → more ACE in the blood (based on his data [57]). In addition, one *ADAMTS13* mutation was identified in the Iceland cohort analysis [see below Table 2]. Therefore, we performed ACE phenotyping in samples obtained from 11 carriers of six different *ADAMTS13* mutations. Their blood ACE levels are shown in Figure 4A, and their values after correction for *ACE* genotype (*ACE* I/D polymorphism) [19] are shown in Figure 4B. Again, as in the case for *ADAM9* mutations, no consistent effect of the *ADAMTS13* mutations on blood ACE levels was observed, even for the five carriers of the same mutation (p.R421C). Three carriers of the p.R421C mutation demonstrated a significant decrease in blood ACE levels, but the other two carriers of the same mutation showed a dramatic increase in blood ACE levels (Figure 4B). Interestingly, in vitro experiments using a cell model of this p.R421C mutation demonstrated that its expression led to a 5-fold decrease in ADAMTS13 activity and protein [58]. Again, as in the case of carriers of *ADAM9* mutations (Figure 3), the presence of Icelandic variants in the carriers of these *ADAMTS13* mutations (Table S4) could not explain the heterogeneity in their blood ACE levels.

**Figure 5:**
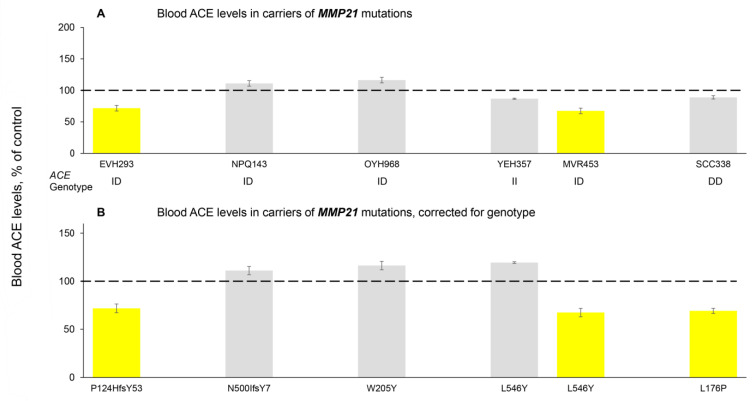
**Quantification of blood ACE levels in carriers of the *MMP21* mutations**. (**A**) The immunoreactive ACE protein was quantified in plasma samples from 6 carriers of 5 different mutations in *MMP21*. (**B**) Plasma ACE levels adjusted according to the donor’s genotype for the I/D polymorphism ACE levels were presented as the means ± SD of several independent assessments of samples. The data are expressed as % of ACE levels of the corresponding value for control pooled plasma samples from donors without *ACE* mutations. Coloring is as described in the legend to Figure 1. The dashed line marks the 100% control baseline.

Another protease under consideration is MMP21 because the Ala97Val variant of this gene (rs148579119) showed a dramatic negative correlation (−1.789) with blood ACE levels (Table S1). Therefore, we performed ACE phenotyping in samples obtained from seven carriers of six *MMP21* mutations. Their blood ACE levels are shown in Figure 4A, and their values after correction for *ACE* genotype (*ACE* I/D polymorphism) [19] are shown in Figure 5B. Due to the heterogeneity of blood ACE levels in the carriers of these *MMP21* mutations (Figure 5), and the absence of a clear pattern of the presence of Icelandic variants in carriers of these *MMP21* mutations, we also could not confirm or exclude MMP21 as a possible ACE secretase.

The dramatic differences in the effects of the same mutation of *ACE*, or other genes, on blood ACE levels (Figure 1B, Figure 3B and Figure 4B, as well as the data from our previous work [19]) strongly supports prioritizing ACE phenotype information over the *ACE* genotype status for the evaluation of the possible AD risk in each individual. The enormous number of variants in 2051 different genes which could influence blood ACE levels suggests that an individual’s blood ACE level results from integrating the effects of many mutations and should be the primary consideration for estimating the risk of ACE-dependent AD. Information about *ACE* mutations is best analyzed together with information about the presence of other mutations from the Icelandic list, and in conjunction with blood ACE phenotype.

Most common diseases and traits are highly pleiotropic, creating a complex polygenic architecture where individual traits are associated with variants at hundreds to thousands of loci in the genome [59]. This, and the additional factors like LD and the presence of non-coding variants which can still impact gene expression, makes it difficult to pinpoint a single causal gene in a GWAS experiment. Thus, many genes might appear associated even if their individual effect size is small [60,61].

After further applying Bonferroni correction to this set of 2388 nominally significant ACE-associated variants at a *p*-value threshold of 3 × 10^−7^, which was previously found reasonable for exome-sequencing studies of rare variants [62], we identified 17 variants in 12 genes that reached exome-wide significance—Table 2. This number of exome-wide significant hits is close to the estimates obtained in FHS [63] analyzing mutations in 71 high-value cardiovascular disease proteins, which influenced blood ACE levels measured in 6861 Framingham Heart Study (FHS) participants. If only non-synonymous substitutions are counted (as we did in our Table S1), the FHS identified seventeen cis-pQTLs and seven trans-pQTLs per one protein associated with blood ACE level changes [63].

This short list (Table 2) contains 4 cis-pQTLs (i.e., 4 mutations in the *ACE* gene) and 3 trans-pQTLs in the *ABO* gene, which confirmed prior observations that variants of the *ABO* gene (actually, blood groups) influence blood ACE levels [25]. The effect of blood group antigens on blood ACE levels seems quite logical: (1) directly, as different “bushes” (sugar moieties) may change the rate of ACE cleavage from the surface by ACE secretase(s); or (2) indirectly via the differential efficacy of ACE trafficking to the cell surface.

This list also contains 10 single trans-pQTLs. Of these, the mutation in the *ADAMTS13* gene (p.R421C, rs145825553) seems to be the most interesting based upon the accumulated evidence in the literature (and our unpublished results) that *ADAMTS13* may be one of the ACE secretases. However, we currently cannot come to a definite conclusion because the apparent effect of this *ADAMTS13* mutation was very heterogeneous. Half of the carriers had increased the blood ACE levels, while the other half dramatically decreased them (Figure 4B).

However, we also speculated that filtering the 2388 Icelandic variants too stringently (*p*-value < 3 × 10^−7^) may be suboptimal because the *p*-value depends on a frequency of a given variant in the population [60,61]. Thus, rare variants of variants, which may significantly change ACE shedding or ACE expression (i.e., those truly affecting measured blood ACE levels), could be lost when using a stringent *p*-value threshold. To help identify additional variants that may influence blood ACE levels, we generated a more condensed list of 215 Icelandic variants that have large beta values (>1.000 in absolute values—Table S5). Future studies will perform blood ACE phenotyping in subjects containing these variants to help validate this approach for identifying other variants that truly influence blood ACE levels. At present, we cannot exclude the possibility that after validation some of these variants will need to be on the list of obligatory mutations that should be evaluated for each carrier of AD-associated *ACE* mutations (i.e., those *ACE* mutations correlated with low blood ACE levels: Y215C, Q259R, T887M, N1007K). Analysis of these variants present in other genes will be necessary to exclude false-negative or false-positive values of blood ACE levels as putative indicators of AD risk.

How can the usefulness of this list of 2388 variants (or shorter list of 215 variants) be proven? We need to validate that these mutations can significantly change blood ACE levels. Figure 1A demonstrates the blood ACE levels in 48 control samples (without *ACE* mutations) which were used for establishing a pool of reference samples (set as 100%) for comparison with blood ACE levels in the carriers of *ACE* mutations. Previously, we measured blood ACE levels (and ACE activity) in 300 random unrelated patients and established that normal values range from 50 to 150% of the mean of the population [15]. Plasma samples belonging to this normal range are marked yellow, gray, and orange (31 samples) in Figure 1A. The normal range in this cohort contains 45 samples when based on mean ± 2SD values. We searched sequences of 48 control samples for expression of any of the 2388 Icelandic variants described above (Table S6). Initially, we identified that 81 variants from this Icelandic list were present in 45 patients with normal blood ACE levels. Variants which were present in control samples from other tested cohorts, which included 129 control samples with normal blood ACE levels (highlighted with light yellow, Table S7), should be excluded from the Icelandic list as an artifact. This is due to potential population-specific differences in the genetic background and allelic structure (LD), as well as the differences in gene–environment interactions in the tested populations. A corrected Iceland list that includes 2248 variants is provided in Table S8.

In addition, three subjects demonstrated dramatic deviations from the normal range (mean + 2SD) in this cohort (one subject on the left and two on the right (boxed) side of Table S6). These three samples should be considered as ACE outliers (Doc S3).

We next applied this approach to search for the presence of the remaining 2248 Icelandic mutations in sequences of 84 carriers of the *ACE* Y215C mutation (Table S9). Before performing the analysis, we predicted that there would be mutations which could significantly change blood ACE levels in at least five subjects who had ACE levels not corresponding to their genotypes (highlighted with yellow in Figure 1B). We found no mutations from the Icelandic list in 50 patients with the *ACE* Y215C mutation (out of 84 carriers). Thus, tissue ACE levels in these patients should be correctly reflected by blood ACE levels (Figure 1B). In the remaining 34 carriers of the Y215C mutation, only 2 (AWQ984 and RKK236) had Icelandic variants (Table S1) with positive correlations (beta values) that would make their blood ACE levels false-positive.

This analysis of 84 carriers of the Y215C *ACE* mutation demonstrated that (1) most carriers of the Y215C mutation had blood ACE which correctly reflect their tissue ACE; (2) however, putative carriers of this (and other) damaging *ACE* mutations should be tested for the Icelandic variants. Validation of the Icelandic variants (i.e., ACE phenotyping of at least the 215 variants with a significant correlation—Table S5) should be continued because other carriers of damaging *ACE* mutations may contain yet-to-be discovered gene-modifiers which could dramatically change the measurable blood ACE levels and thus lead to incorrect conclusions about their risk for ACE-dependent AD.

However, it is necessary to be very cautious about GWAS databases in general, and this Icelandic list in particular, in relation to the influence on ACE levels. Only 30% of the evaluated pQTLs (based on GWAS) were confirmed by MS proteomics, whereas another 30% could not be replicated and are possibly due to the epitope effect [64]. To clarify such a potential discrepancy for our study, we compared the cis-pQTL for missense *ACE* mutations found in 54,219 sequenced subjects in the UK Biobank [26] with that found in 35,549 sequenced subjects in the Iceland cohort [21]—Table S4 in [9]—as shown in Table 3.

Table 3 clearly demonstrates that only 7 cis-pQTLs (*ACE* mutations) found in the UK Biobank (out of 16) were present in the list of 23 pQTLs found in the Iceland cohort (Table S4 in [9]). For this reason, we were not able to confirm that more than 20 variants of different genes from the Icelandic list (Table S1) with high correlations of blood ACE levels (beta values > than 1.00 in absolute values) truly predicted (corresponding) changes in blood ACE levels in these carriers (usually more than five carriers per each variant.

GWAS analysis of both the Iceland cohort (Table S4 in [9]) and the UK Biobank cohort (Table 3) identified the Y215C mutation as the strongest influencer of blood ACE levels. This mutation was considered potentially causal for AD [2] because of its very high frequency (1.5%) in the general population. However, the potential contributions to the AD risk of other *ACE* mutations, which are damaging but much more rare—Table S2 in [9]—are missing if only relying on GWAS data. Therefore, because there are more than 400 damaging *ACE* mutations [9], and especially those that are nonsense and frame shifting *ACE* mutations ([14], Table S2 in [9]), it is absolutely necessary to perform ACE phenotyping in subjects with damaging *ACE* mutations by genotype. Only this additional analysis can appropriately determine the risk group for AD (and not GWAS data).

Regardless of the limitations of GWAS data, and the Iceland cohort in particular, we have identified a solution for outliers in blood ACE levels (e.g., carriers of the Y215C or other mutations), in whom blood ACE levels differ dramatically from those expected (based on *ACE* genotype or based on the effect of given *ACE* mutation of the blood ACE levels [16,17,18,19]). These subjects can be tested more accurately for tissue ACE levels using another independent method. An example is the quantification of ACE proteins on the surface of ACE-expressing cells using flow cytometry with our mAbs to ACE [65,66,67].

### 3.3. Search for Genes (Variants) Protective for ACE-Dependent AD

Some carriers of the Y215C *ACE* mutation (up to 37%) did not show any decrease in blood ACE levels (gray bars in Figure 1B) or even demonstrated an increase in blood ACE (orange, brown, or red bars in Figure 1B), indicating that other acquired, environmental, or trans-acting genetic factors somehow compensate for the damaging effects of the Y215C mutation. This observation raises the theoretical possibility that there are still unknown mutations of other genes which protect against lowering ACE in these subjects (i.e., those shown on the right side of Figure 1B). Such mutations could be protective against ACE-dependent AD. The few variants from the Iceland cohort which we mentioned above as putative gene-modifiers of blood ACE levels are not sufficient to explain the significant heterogeneity of blood ACE levels observed in the 84 carriers of the Y215C *ACE* mutation (Figure 1B). It seems possible that other variants may work as protective genes which can compensate for the dramatic diminishing effect of the Y215C mutation on surface ACE expression [20]. Analogous protective variants were found recently in patients carrying *PSEN1*-E280A mutations, which cause extreme resilience to autosomal dominant AD [68].

How can these other protective variants be found in carriers of the Y215C *ACE* mutation? Intuitively, patients with low blood (i.e., tissue) ACE levels (blue bars on the left side of Figure 1B) are the first candidates for ACE-dependent AD, while patients on the right side of this figure (red, brown, orange, and perhaps even gray bars) may carry some protective variants that somehow compensate for the Y215C *ACE* mutation effects. Therefore, a reasonable initial strategy is to identify the variants which are present in sequences of the subjects shown on the right side of Figure 1B and absent in sequences on the left side (blue bars, or perhaps blue and yellow bars).

As a first step in this approach, we examined the presence of variants in *SORL1*, a candidate modifier gene, among the Y215C mutation carriers. Sorting-related receptor with A-type repeats (SORL1) is an intracellular sorting receptor that directs multiple cargo proteins—including kinases, phosphatases, signaling receptors, and APP—to their appropriate cellular compartments [69,70]. We hypothesized that altered quality control or sorting of the misfolded Y215C ACE protein might facilitate its appearance at the cell surface. However, no LoF variants in *SORL1* [43] were detected among Y215C carriers, and all nine identified missense variants (Table S10) were classified as benign according to ACMG guidelines [71].

Given the lack of statistically significant associations in the GWAS analysis, with *p*-values close to 1 even prior to correction for multiple testing, we applied a systematic variant-level comparison between individuals with elevated and reduced circulating ACE levels. Specifically, we compared the whole-exome variant profiles of 15 individuals with elevated ACE levels, corresponding to the right cluster in Figure 1B, against 44 individuals with decreased ACE levels. This analysis identified 9648 variants that were present exclusively in individuals with elevated ACE levels and were completely absent in individuals with low ACE levels. To reduce stochastic noise from singletons, we focused on variants present in three to five individuals within the high-ACE group, identifying 42 variants in 41 unique genes (Table 4).

Our hypothesis was that patients with elevated levels of ACE, despite the presence of the Y215C mutation, may harbor additional variants affecting the genes involved in protein quality control and trafficking, including molecular chaperones (more than 330 genes [72]), ubiquitination-related pathways (~600 genes annotated under GO:0016567), and components of the protein translocation machinery such as the Sec61 translocon.

Gene Ontology enrichment analysis was performed using ShinyGO v0.85.1 [73] on the set of recurrent variants exclusive to individuals with elevated circulating ACE levels (Table 4), defined as variants observed in three to five individuals within this group. The analysis identified a limited number of enriched Molecular Function terms, which were primarily associated with structural constituents of the postsynaptic compartment, including the postsynaptic density and postsynaptic specialization (GO:0098879, GO:0098919, GO:0098918, GO:0099186). These signals were driven predominantly by the scaffold proteins DLG1 and SHANK3. In addition, enrichment for ionotropic glutamate receptor binding (GO:0035255) further pointed to the involvement of postsynaptic signaling complexes.

Although individual candidate genes such as *MKKS* and *DNAJC21* are functionally linked to protein folding machinery, these genes did not cluster into coherent Gene Ontology categories related to protein translocation, chaperone-mediated folding, ubiquitination, or global protein quality control pathways. Importantly, this lack of clustering does not contradict the working hypothesis, as defects affecting ACE processing and trafficking may be highly individualized and mutation-specific.

Overall, the enrichment analysis primarily highlighted membrane-associated and structural proteins, suggesting that inter-individual variability in circulating ACE levels may arise through indirect mechanisms, such as altered membrane organization or stability of protein complexes, rather than through the uniform disruption of core protein quality control machinery shared across multiple individuals.

Among the 9648 variants observed exclusively in individuals with elevated circulating ACE levels, most were present in only one or two subjects. Restricting the analysis to protein-coding and well-annotated genes revealed that these rare variants mapped to 5779 genes. Functional enrichment analysis of this gene set demonstrated that, despite pronounced inter-individual diversity at the variant level, these genes converged on fundamental cellular processes related to organelle organization (GO:0006996) and cytoskeletal protein binding (GO:0008092). Notably, the significantly enriched terms (FDR < 0.05) further refined these processes to intracellular and microtubule-dependent transport, as well as multiple classes of transmembrane transporters. Importantly, the enrichment of transport-related pathways does not imply canonical or fully efficient trafficking. Rather, it indicates that inter-individual variation in intracellular transport and membrane delivery mechanisms may influence the efficiency with which a subset of partially misfolded Y215C ACE molecules reaches the plasma membrane. Such variability could modulate subsequent ACE shedding and contribute to elevated circulating ACE levels in individual carriers.

Large-scale population sequencing studies have demonstrated that each human genome harbors millions of small variants and thousands of structural variants, resulting in substantial inter-individual genomic diversity. The 1000 Genomes Project Consortium showed that a typical human genome differs from the reference genome at 4.1–5.0 million sites [74]. More recent analyses of nearly 15,000 genomes in gnomAD SV further revealed a median of 7439 structural variants per individual [75]. In this context, the highly heterogeneous variant landscape observed in individuals with elevated ACE levels is consistent with a personalized genetic architecture, in which distinct combinations of rare variants modulate disease-relevant pathways in different patients.

## 4. Limitations of Our Study

Our study has some limitations that should be noted. It is limited by the available sample size, which constrains the statistical power for stratified analyses by age, lifestyle factors, or family history. In addition, repeated longitudinal follow-up of Y215C carriers is challenging, restricting the ability to fully characterize interactions between *ACE* variants, clinical phenotypes, and other AD risk factors. Finally, detailed phenotyping beyond ACE measurements was only available in subsets of participants, and the clinical implications of protective or compensatory mutations remain hypothetical until further functional or interventional studies are performed.

## 5. Conclusions

1. The precipitation of ACE activity from EDTA-containing plasma obtained from 84 carriers of the Y215C *ACE* mutation (using the strong mAb 9B9 to ACE) demonstrated that a significant portion of these carriers had decreased ACE levels in the blood. Thus, they could be considered at increased risk for late-onset AD. The mAbs 5B3/1G12 binding ratio was revealed as a sensitive marker for the circulating Y215C ACE mutant.
2. Some carriers of the Y215C *ACE* mutation demonstrated significant heterogeneity in blood ACE levels. This observation indicates that information about mutations in other genes that can influence ACE shedding should also be obtained for a given individual. Otherwise, blood ACE values could be misleading, as false-negative or false-positive.
3. Analysis of 2388 variants which may change ACE shedding was performed using sequencing and proteomic data from 35,500+ Icelanders [21]. Several gene-modifiers were identified, including two possible candidates for ACE secretases, *ADAM9* and *ADAMTS13*.
4. The presence of this damaging *ACE* mutation, Y215C, does not guarantee a low level of ACE in the subject. Blood ACE phenotyping and analysis of WES also should be performed to identify patients with false-negative or -positive blood ACE to avoid inaccurate conclusions about the risk for ACE-dependent AD in carriers of this *ACE* mutation.
5. Although GWAS did not identify statistically significant protective variants at the cohort level, the comparative analysis of carriers with elevated versus decreased circulating ACE levels revealed numerous rare variants present exclusively in individuals with preserved ACE levels. These variants did not converge on a single canonical pathway, suggesting that compensatory mechanisms counteracting the deleterious effect of the Y215C mutation are mediated by distinct, individual-specific genetic modifiers. This variability underscores the importance of personalized genomic interpretation when assessing ACE-dependent AD risk.

## Figures and Tables

**Table 1:** Frequency of the Y215C variant (rs3730025) in the *ACE* gene across populations and cohorts.

	No. of Subjects	MAF (/100,000)	No. of Y215C Carriers
**Population/race** (dbSNP)			
European	349,976	1444	5054
African	36,268	312	113
Latinos	9570	170	16
Asian	12,238	57	7
South Asian	8960	1	0.1
Total	434,616	1267	5507
TOPMED	264,690	924	2447
**Known major cohorts**			
UK Biobank [26]	54,512	1626	951
Icelanders [21]	35,559	1534	545
**Cohorts theoretically available in Russia**			
FMBA [41]	120,762	2571	3105
RUSEQ (Genetico)	12,976	1318	171
Genomed	37,620	1881	708
RSMU	4657	1181	55
RUSS-AGE	290	2411	7
Longevity	200	2500	5

Notes: MAF/100,000—mean allele frequency, scaled per 100,000 alleles. Discrepancies may arise from methodological differences between sources (e.g., quality filtering criteria, annotation pipelines). MAF estimates in small cohorts (*n* < 1000) should be interpreted with caution due to limited sample size.

**Table 2:** Genes, which mutations influenced blood ACE levels—based on Ferkingstad, 2021 [21].

No.	rsID	Gene	UNIPROT Number	Protein Position	AA	Beta Value	*p*-Value	Poly-Phen-2	ImpMAF/100,000	MAF dbSNP/100,000
1	rs8176743	*ABO*	P16442	234	G/S	0.351	5.356 × 10^−105^	0.168	**6.488**	**10,587**
2	rs8176746	*ABO*	-	265	L/M	0.35	8.358 × 10^−105^	0.09	**6.496**	**10,589**
3	rs8176747	*ABO*	-	267	G/A	0.35	9.314 × 10^−105^	0.003	**6.497**	**10,589**
4	rs750712925	*ACE*	P12821	45	G/R	−1.288	6.8 × 10^−15^	0.142	82	1.9
5	rs3730025	*ACE*	-	244	Y/C	−1.217	3.306 × 10^−291^	**0.998**	**1.534**	**924**
6	rs757694144	*ACE*	-	482	R/P	−1.349	1.50 × 10^−7^	0.246	34	0.4
7	rs372416620	*ACE*	-	1243	V/I	0.371	6.70 × 10^−9^	0.061	**469**	5.7
8	rs145825553	*ADAMTS13*	Q76LX8	421	R/C	0.394	2.40 × 10^−8^	**0.994**	**334**	45
9	rs771742994	*BCAS3*	Q9H6U6	480	S/I	−1.162	2.20 × 10^−12^	**0.991**	88	5.7
10	rs117181531	*DDX42*	Q86XP3	754	S/I	0.429	4.50 × 10^−9^	0.114	**317**	**180**
11	rs117595304	*GBGT1*	Q8N5D6	66	Y/C	−0.363	1.20 × 10^−9^	**0.817**	**457**	**311**
12	rs367887663	*MFSD6L*	Q8IWD5	323	H/D	0.505	3.50 × 10^−8^	**0.902**	**166**	7.2
13	rs1352161075	*MILR1*	Q7Z6M3	159	T/I	0.376	1.20 × 10^−10^	0.035	**539**	3.4
14	rs201723860	*SMARCD2*	Q92925	201	T/M	0.478	7.00 × 10^−8^	**0.985**	**242**	15
15	rs750512077	*STRADA*	Q7RTN6	215	R/H	−0.405	2.80 × 10^−9^	**0.999**	**352**	1.5
16	rs370863314	*TANC2*	Q9HCD6	2015	R/P	0.479	8.20 × 10^−9^	0.066	**232**	10
17	rs780968826	*TEX2*	Q8IWB9	375	E/K	0.366	9.00 × 10^−10^	0.003	**486**	0.5

Notes: After further applying Bonferroni correction at *p*-value threshold 3 × 10^−7^ to 2387 variants in Table S1, 17 variants in 12 genes reached exome-wide significance. Correlation of blood ACE levels with each variant were expressed as beta values. PolyPhen-2 (dbNSFP version 3.3a) annotation is based on HumVar model and consists of score and categorical predictions: probably damaging, score ≥ 0.909), possibly damaging, 0.446 ≤ score ≤ 0.908), benign, score ≤ 0.445). For clarity in the table, the ‘probably damaging’ category (D) is highlighted in bold. Mean allele frequency (MAF) for mutations in Iceland’s population and from dbSNP were expressed per 100,000 subjects. MAF values greater than 100 are highlighted in bold to indicate elevated counts. Effect of one *ACE* mutation on blood ACE level was validated on numerous carriers of this mutation [16,17,18,19], this study—Figure 1. All mutations were missense.

**Table 3:** Correlations of ACE levels with *ACE* mutations in 54,549 sequenced subjects in the UK Biobank.

No.	rsID	Ref	Alt	Mut	Protein	AA	MAF/100,000		Poly-Phen-2	SE	BETA UKBB	Beta Iceland	*p*-Value Iceland
							dbSNP	UK Biobank					
1	rs532691783	-	GCTGCC	ins	13, SP	L/LLP	6	197	-	0.078	−0.288	NA	NA
2	rs3730025	A	G	ms	244	Y215C	924	1682	**0.998**	0.023	−0.422	−1.227	3.31 × 10^−291^
3	rs4303	G	T	ms	261	A232S	1118	337	**0.811**	0.057	−0.152	NA	NA
4	rs149412997	G	A	ms	267	G238R	117	84	**0.965**	0.119	−0.140	NA	NA
5	rs35141294	C	T	ms	324	R295W	851	100	**0.984**	0.106	−0.245	0.136	0.742838
6	rs56394458	G	A	ms	354	G325R	539	884	**0.999**	0.034	0.343	0.265	3.68 × 10^−10^
7	rs150466411	C	T	ms	381	T352M	67	139	**0.993**	0.08	0.0560	−0.281	0.269633
8	rs28730839	C	G	ms	485	P456R	47	95	0.249	0.099	0.305	NA	NA
9	rs12709426	A	G	ms	592	D563G	1588	213	0.072	0.071	−0.254	NA	NA
10	rs147429960	C	G	ms	660	S631C	95	148	0.335	0.089	0.044	NA	NA
11	rs117647476	A	G	ms	798	I769V	192	463	0.004	0.048	0.154	0.145	6.08 × 10^−5^
12	rs3730043	C	T	ms	916	T887M	419	856	**0.980**	0.033	0.044	0.138	0.043456
13	rs141750591	G	A	ms	978	V949M	23	80	**0.996**	0.117	0.018	0.004	0.989591
14	rs4980	G	A	ms	1279	R1250Q	493	496	0.010	0.050	0.295	0.156	0.015164
15	rs4364	C	A	ms	1286	R1257S	156	355	0.130	0.059	−0.294	NA	NA
16	rs12720745	G	A	ms	1290	R1261Q	691	58	0	0.142	−0.046	NA	NA

Notes: Only 5 variants (out of these 7) demonstrated significance for beta values and these 5 variants were also significant in the Iceland cohort. PolyPhen-2 (dbNSFP version 3.3a) annotation is based on HumVar model and consists of score and categorical predictions: D (probably damaging, score ≥ 0.909), P (possibly damaging, 0.446 ≤ score ≤ 0.908), B (benign, score ≤ 0.445). For clarity in the table, the ‘probably damaging’ category (D) is highlighted in bold. Mut—mutation type, ms—missense, ins—insertion.

**Table 4:** Recurrent variants exclusive to individuals with elevated circulating ACE levels.

No	Gene, Coding DNA (rsID)	Patient Identifier															MAF/100,000	
		1737	1340	181	614	615	HSY004	HWY602	JQW970	KJB939	OTS421	OTX780	SRZ900	TXM676	VYA317	XLK932	gnomAD v.4.1.0	FMBA [41]
1	***CD34***, c.398C>T (rs148688256)	-	-	-	-	-	Het	-	Het	-	-	-	-	-	-	Het	192	**1020**
2	***TMEM240****,* c.454G>A (rs146206869)	-	-	-	Het	-	-	-	-	-	-	Het	-	Het	-	-	**3364**	**2825**
3	***CHST15***, c.98C>T (rs34639461)	-	Het	-	-	-	-	-	-	-	-	Het	-	Het	-	-	**2984**	**2880**
4	***ACCSL***, c.133G>A (rs11037840)	-	-	-	-	-	-	Het	Het	-	-	-	Het	-	-	-	450	**1220**
5	***MYRFL***, c.2072C>T (rs61754226)	Het	-	-	-	-	-	-	-	-	-	Het	-	-	-	Het	690	620
6	***TMCC3***, c.564G>A (rs149007412)	-	Het	-	-	-	-	-	Het	-	-	-	-	-	Het	-	674	**1270**
7	***LTBP2***, c.4769T>C (rs139932140)	-	-	-	-	-	-	-	Het	-	-	-	Het	-	-	-	773	**1805**
8	***RPAP1***, c.146C>T (rs112536229)	Het	-	Het	-	-	-	-	Het	-	-	-	-	-	-	-	**1157**	**1680**
9	***CFAP161***, c.850C>T (rs2279997)	-	-	-	-	Het	-	Het	Het	-	-	-	-	-	-	-	**1080**	**1290**
10	***PKD1***, c.10529C>T (rs45478794)	Het	-	-	-	-	-	-	Het	Het	Het	-	-	-	Het	-	**1320**	**1820**
11	***ARMC5***, c.508A>G (rs35923277)	-	-	-	-	-	-	-	-	Het	-	Het	Het	-	-	-	**4147**	**4510**
12	***ZNF469***, c.1994C>T (rs184583062)	-	-	-	-	-	-	-	Het	Het	-	Het	-	-	-	-	783	900
13	***KIF1C***, c.2105C>T (rs138935423)	-	Het	-	Het	Het	-	-	-	-	Het	-	-	-	-	-	752	**1280**
14	***PER1***, c.2575C>T (rs112980285)	-	-	-	-	-	-	-	Het	-	-	-	Het	-	Het	-	**2368**	**1810**
15	***PIEZO2***, c.4203C>G (rs79261438)	Het	-	Het	-	-	-	-	-	-	-	Het	-	-	Het	-	**2986**	**2700**
16	***HSH2D***, c.410C>T (rs36088948)	-	-	Het	-	-	-	-	-	Het	-	-	-	Het	Het	-	**6876**	**4430**
17	***SCAMP4***, c.596C>T (rs75734024)	-	Het	-	-	-	-	-	-	Het	-	-	-	-	Het	-	**1726**	**1460**
18	***ZNF568***, c.1088C>T (rs1667364)	-	-	-	-	-	-	-	-	Het	-	Het	-	-	Het	-	**1874**	**1340**
19	***CEACAM21***, c.253_255dup (rs3030812)	-	-	-	-	Het	-	-	-	-	Het	-	-	Het	-	-	**4181**	**3740**
20	***CEACAM21***, c.333C>G (rs78133615)	-	-	-	-	Het	-	-	-	-	Het	-	-	Het	-	-	**4109**	**3730**
21	***PLIN5***, c.73C>T (rs11085080)	-	-	-	-	-	-	Hom	-	Het	Het	-	-	-	-	-	**6443**	**4910**
22	***NOP53***, c.91G>C (rs78530808)	-	-	-	-	Het	-	Het	-	-	-	-	Het	-	-	-	**1801**	**2850**
23	***NRP2***, c.2716_2717insA (rs200483574)	-	-	Het	-	Het	-	-	-	-	Het	-	-	-	-	-	**4437**	**4340**
24	***GCFC2***, c.1812+399dup (rs11423284)	-	-	Het	Het	-	-	-	-	-	-	Het	-	-	-	-	**3960**	**1638**
25	***MKKS***, c.1015A>G (rs137853909)	Het	-	-	-	-	Het	-	-	-	-	Het	-	-	-	-	417	**1030**
26	***CNBD2***, c.622A>G (rs6142471)	-	-	-	-	Het	-	-	Het	-	Het	-	-	-	-	-	112	749
27	***BPI***, c.1051C>T (rs5743523)	-	-	-	Het	Het	-	Het	-	-	-	Het	-	-	-	-	**2502**	**4070**
28	***SHANK3***, c.2347G>A (rs61729471)	-	-	-	-	-	Het	-	-	-	-	-	Het	-	Het	-	**3357**	**4580**
29	***HCLS1***, c.1162G>A (rs77852202)	Het	-	-	-	-	Het	-	-	-	Het	-	-	-	-	-	**5512**	**3560**
30	***DLG1***, c.2357G>A (rs78190191)	Het	-	Het	-	-	-	-	-	-	-	-	-	-	-	-	**2017**	**3080**
31	***CCDC149***, c.1525C>T (rs74764772)	-	Het	-	-	-	-	-	Het	-	-	-	-	Het	-	-	946	**2600**
32	***DNAJC21***, c.1024G>A (rs144600070)	-	-	-	-	-	Het	-	-	Het	-	-	-	-	-	Het	457	859
33	***PHACTR2***, c.4G>A (rs41285023)	Het	-	-	-	-	-	-	-	-	Het	Het	-	-	-	-	**2244**	**1270**
34	***TBC1D32***, c.3695A>C (rs56300302)	Het	-	-	Het	-	-	-	Het	-	-	-	-	-	-	-	**1632**	**1638**
35	***SCUBE3***, c.1229C>T (rs3800381)	-	-	-	-	-	-	-	Het	-	-	-	-	Het	-	Het	198	392
36	***TSC22D4***, c.1150C>T (rs34666277)	Het	-	-	-	Hom	-	-	-	-	-	-	-	-	Het	-	626	800
37	***CUX1***, c.1573C>G (rs138450169)	-	Het	-	Het	-	-	-	-	Het	-	-	-	-	-	-	689	**1244**
38	***CRB2***, c.278G>A (rs138381817)	Het	-	-	-	-	-	-	-	-	-	-	Het	-	-	-	730	896
39	***DENND1A***, c.2534C>A (rs189947178)	Het	Het	-	-	-	-	-	-	-	-	-	Het	-	-	-	679	896
40	***TMC1***, c.421C>T (rs11143384)	-	Het	Het	Hom	-	-	-	-	-	-	-	-	-	-	-	957	990
41	***MAN1B1***, c.1896+22G>A (rs117994893)	Het	-	Het	-	-	-	-	Het	-	-	-	-	-	-	-	**3454**	**4550**
42	***TXLNG***, c.736A>G (rs5969783)	-	-	Het	-	Het	-	-	-	-	-	-	-	-	-	Het	**2885**	**2600**

Notes: All variants are classified as benign according to ACMG criteria, except *MKKS*(NM_170784.3):c.1015A>G p.(Ile339Val). MAF/100,000—mean allele frequency, scaled per 100,000 alleles. MAF values greater than 1000 are highlighted in bold to indicate elevated counts. Het—heterozygous. Hom—homozygous.

## Data Availability

The data that support the findings of this study are available from the corresponding author upon reasonable request.

## Supplementary Materials

The following supporting information can be downloaded at https://www.mdpi.com/article/10.3390/biomedicines14020275/s1, Doc S1–S3. Table S1: Initial mutations (2388) that may influence blood ACE levels (Iceland’s list). Table S2: Annotation of genes and variants in Table S1 (Iceland’s list). Table S3: List of putative ACE secretases. Table S4: Association of circulating ACE levels with rare coding variants identified in carriers of *ADAM9*, *ADAMTS13*, and *MMP21*. Table S5: Shorter Iceland’s list (215) of variants that may dramatically influence blood ACE levels. Table S6: Mutations from Iceland’s list in 48 control samples. Table S7: Mutations from Iceland’s list in 127 control samples. Table S8: Corrected list of mutations (2248) that may influence blood ACE levels (Iceland’s list). Table S9: Mutations from Iceland’s list in 84 carriers of Y215C mutations. Table S10: *SORL1* mutations in carriers of Y215C *ACE* mutation.
