# Supplementary material for: ACE-Dependent Alzheimer’s Disease: Blood ACE Phenotyping of the Most Prevalent and Damaging ACE Missense Mutation—Y215C (rs3730025)
Source: Biomedicines. 2026 Jan 26;14(2):275. doi: 10.3390/biomedicines14020275 (PMC12937726; doi:10.3390/biomedicines14020275)
Supplement: Supplementary file 1 [file biomedicines-14-00275-s001.zip › Supplementary Materials Part I.pdf]

Supplementary Materials to paper:
ACE-Dependent Alzheimer's Disease: Blood ACE Phenotyping of the Most Prevalent and Damaging ACE Missense Mutation—Y215C (rs3730025)

Anastasiia A. Buianova, Ivan A. Adzhubei, Olga V. Kryukova, Olga A. Kost, Iaroslav V. Mironenko, Alex S. Kozuch, Galit A. Ilyina, Anna A. Kuznetsova, Zhanna A. Repinskaia, Alexey V. Churov, Steven M. Dudek, Denis V. Rebrikov and Sergei M. Danilov

Running Head: Blood ACE phenotype of Y215C ACE mutant.

Doc S1.

Apolipoprotein E4 (apoE4) contributes to the pathogenesis of AD by promoting the accumulation of Aβ and hyperphosphorylated tau, reducing the expression of key synaptic proteins and inhibiting glutamatergic signaling. Additionally, apoE4 induces cholesterol accumulation in the cerebral cortex, triggers inflammatory processes, and disrupts blood-brain barrier integrity, leading to decreased neuronal plasticity, impaired microglial clearance of Aβ, and cerebrovascular damage, thereby increasing the risk of both AD and vascular cognitive impairment and dementia [1, 2]. It is known that by age 85, individuals with two ε4 alleles (ε4/ε4 homozygotes) have a 48.3% risk (95% CI: 40.1–57.3%) of developing AD. In heterozygotes carrying ε3/ε4 or ε2/ε4 genotypes, the risk is substantially lower at 18.4% (95% CI: 16.5–20.4%). For ε3 homozygotes (ε3/ε3), the risk is 8.6% (95% CI: 7.7–9.6%). The lowest risk is observed in individuals carrying at least one ε2 allele (ε2/ε3 or ε2/ε2), estimated at 5.5% (95% CI: 4.1–7.4%) [3].

References for Doc S1:

1. Hunsberger HC, Pinky PD, Smith W, Suppiramaniam V, Reed MN. The role of APOE4 in Alzheimer's disease: strategies for future therapeutic interventions. *Neuronal Signal*. 2019;3:NS20180203. doi:10.1042/NS20180203
2. Raulin AC, Doss SV, Trottier ZA, Ikezu TC, Bu G, Liu CC. ApoE in Alzheimer's disease: pathophysiology and therapeutic strategies. *Mol Neurodegener*. 2022;1:72.
3. van der Lee SJ, Wolters FJ, Ikram MK, et al. The effect of APOE and other common genetic variants on the onset of Alzheimer's disease and dementia: a community-based cohort study. *Lancet Neurol*. 2018;17:434-444. doi:10.1016/S1474-4422(18)30053-X

Doc S2. Search for ACE secretase(s).

ACE is a type I transmembrane zinc metallopeptidase existing predominantly on endothelial surfaces such as lung and vasculature. Soluble form of ACE is produced by proteolytic cleavage of a juxtamembrane stalk region by some secretase [1, 2] which has not yet been identified. Previous studies have established that ACE secretase is a membrane-bound metalloprotease [1], which should be co-expressed in ACE-rich vascular/endothelial tissues.
ACE secretase activity can be inhibited by hydroxamate-based compounds [3, 4] which inhibit a variety of metalloproteases. Moreover, unlike several other secretases, the ACE-secretase does not

require specific amino acid residues at or around the cleavage site [5-7]. Instead, the site is at a fixed distance from the transmembrane domain and the cleavage is dictated by the distal ectodomain of the protein. The possibility of the existence of several ACE secretases was also proclaimed [8].

Therefore, to identify the putative protease(s) responsible for ACE shedding from the membrane, we performed an extensive analysis of existing literature. We initially hoped to identify these "ACE secretases" from the Icelandic database of 2,388 variant associations. However, this approach did not yield a single definitive metalloprotease gene, as not one, but several candidates contained mutations significantly correlated with circulating ACE levels.

Consequently, we employed artificial intelligence (AI) to analyze the Icelandic dataset for potential secretase candidates. The AI proposed a list of 35 putative candidates. This list was critically evaluated and refined through a thorough literature review, resulting in the exclusion of several candidates. Because ACE sheddase should be a metalloprotease, namely zinc-dependent metalloprotease, we excluded most of non-metalloproteases or non-peptidases like NEIL1/TF/CLCA/ADGR/ADAMTSL. Furthermore, we independently compiled a list of proteases implicated in the literature, including by our co-authors. The consolidated results are presented in Table S3.

We previously analyzed sequence of patient 47S (Genotek data) with extremely low serum ACE activity (less than 50%) but without *ACE* mutations and found 19 candidate genes, with *ADAMTS17*, calpain inhibitor (*CAPN2*), and proprotein convertase *PCSK5* being the most probable – Table S2 in [9]. These candidate genes were also included in Table S3. Notably, all three genes are represented in the Icelandic population variation database. A potential role for calpains is rather interesting as our earlier data showed that calpain inhibitors increase ACE shedding in cell culture, suggesting calpain may proteolytically inactivate the ACE secretase [4].

Previously, we analyzed the expression of ADAMs in the prostate epithelial cells (using Human Protein Atlas Data https://www.proteinatlas.org/). This analysis was based on the assumption that, due to 50X more ACE in the seminal fluid than in the blood, expression of putative ACE secretases should be exceptionally higher in these cells comparing to endothelial cells. This analysis gave us three candidates among ADAMs – *ADAM28, ADAM29* and *ADAM33*, which are highly expressed in these cells [10-12].

Notable interspecies differences in serum ACE levels may offer further clues. The guinea pig exhibits extraordinarily high serum ACE activity and a high serum-to-lung ACE ratio compared to humans, mice, rats, and rabbits [13]. This phenotype may be driven by correspondingly high expression or activity of a dominant sheddase, such as an ADAMTS family member, in this species.

Indirect genetic evidence further implicates ADAM/ADAMTS family members. Analysis of all missense mutations within *ADAMTS* genes in the Icelandic database revealed a substantial number in *ADAMTS7* (63 mutations) and *ADAMTS13* (29 mutations). Their high mutational load suggests functional diversity that could influence protease activity [Icelandic deCODE Genetics Database].

Early genome-wide association studies (GWAS) for serum ACE levels identified not only the strong cis-QTL at the ACE locus but also suggestive trans-QTL signals on chromosomes 9 (containing ADAMTS13), 4 (containing ADAM29), and 1 (containing CAPN2) [14].

ADAM10 and the tumor necrosis factor-alpha converting enzyme (TACE, ADAM17) were not previously considered as themain ACE secretases which shed ACE from the membranes of human SH-SY5Y cells [15]. Further studies *in vivo* using conditional endothelial-specific *ADAM10* knockout mice, which exhibited a substantial reduction in plasma ACE levels, however, claimed ADAM10 as the major physiological ACE secretase [16]. Finally, the most recent results established that **ADAM10 is the primary ACE sheddase**, directly cleaving ACE from the membrane in response to stimuli, while **ADAM17 contributes indirectly** by promoting the release of soluble mediators which enhance ADAM10-mediated shedding [17].

Thus, we included several ADAMs into Table S3.

In addition, we would like to note that possibly several secretases might regulate ACE shedding. While one secretase can serve as a principal sheddase, another one can contribute to ACE regulation through transcriptional mechanisms rather than direct proteolytic cleavage. Moreover, we could also imagine secretases with different efficacy towards ACE shedding. Certainly, this idea requires further studies.

References for Doc S2:

1. Oppong, S.Y.; Hooper, N.M. Characterization of a secretase activity which releases angiotensin-converting enzyme from the membrane. *Biochem. J.* **1993**, 292 (Pt 2), 597–603.
2. Chattopadhyay, S.; Karan, G.; Sen, I.; Sen, G.C. A small region in the angiotensin-converting enzyme distal ectodomain is required for cleavage-secretion of the protein at the plasma membrane. *Biochemistry* 2008, 47, 8335–8341.
3. Parkin, E.T.; Trew, A.; Christie, G.; Turner, A.J.; Hooper, N.M. Structure-activity relationship of hydroxamate-based inhibitors on the secretases that cleave the amyloid precursor protein, angiotensin converting enzyme, CD23, and pro-tumor necrosis factor-alpha. *Biochemistry* 2002, 41, 4972–4981.
4. Balyasnikova, I.V.; Karran, E.H.; Albrecht, R.F., 2nd; Danilov, S.M. Epitope-specific antibody-induced cleavage of angiotensin-converting enzyme from the cell surface. *Biochem. J.* 2002, 362, 585–595.
5. Sadhukhan, R.; Sen, G.C.; Ramchandran, R.; Sen, I. The distal ectodomain of angiotensin-converting enzyme regulates its cleavage-secretion from the cell surface. *Proc. Natl. Acad. Sci. USA* 1998, 95, 138–143.
6. Schwager, S.L.; Chubb, A.J.; Scholle, R.R.; Brandt, W.F.; Mentele, R.; Riordan, J.F.; Sturrock, E.D.; Ehlers, M.R. Modulation of juxtamembrane cleavage ("shedding") of angiotensin-converting enzyme by stalk glycosylation: evidence for an alternative shedding protease. *Biochemistry* 1999, 38, 10388–10397.
7. Pang, S.; Chubb, A.J.; Schwager, S.L.; Ehlers, M.R.; Sturrock, E.D.; Hooper, N.M. Roles of the juxtamembrane and extracellular domains of angiotensin-converting enzyme in ectodomain shedding. *Biochem. J.* 2001, 358, 185–192.
8. Alfalah, M.; Parkin, E.T.; Jacob, R.; Sturrock, E.D.; Mentele, R.; Turner, A.J.; Hooper, N.M.; Naim, H.Y. A point mutation in the juxtamembrane stalk of human angiotensin I-converting enzyme invokes the action of a distinct secretase. *J. Biol. Chem.* 2001, 276, 21105–21109.
9. Samokhodskaya, L.M.; Jain, M.S.; Kurilova, O.V.; Bobkov, A.P.; Kamalov, A.A.; Dudek, S.M.; Danilov, S.M. Phenotyping Angiotensin-Converting Enzyme in Blood: A Necessary Approach for Precision Medicine. *J. Appl. Lab. Med.* 2021, 6, 1179–1191.
10. Hohlbrugger, G.; Pschorr, J.; Dahlheim, H. Angiotensin I converting enzyme in the ejaculate of fertile and infertile men. *Fertil. Steril.* 1984, 41, 324–325.
11. Krassnigg, F.; Niederhauser, H.; Fink, E.; Frick, J.; Schill, W.B. Angiotensin converting enzyme in human seminal plasma is synthesized by the testis, epididymis and prostate. *Int. J. Androl.* 1989, 12, 22–28.
12. Nikolaeva, M.A.; Balyasnikova, I.V.; Alexinskaya, M.A.; Metzger, R.; Franke, F.E.; Albrecht, R.F., 2nd; Danilov, S.M. Testicular isoform of angiotensin I-converting enzyme (ACE, CD143) on the surface of human spermatozoa: revelation and quantification using monoclonal antibodies. *Am. J. Reprod. Immunol.* 2006, 55, 54–68.
13. Balyasnikova, I.V.; Yeomans, D.C.; McDonald, T.B.; Danilov, S.M. Antibody-mediated lung endothelium targeting: in vivo model on primates. *GeneTher.* 2002, 9, 282–290.
14. Kammerer, C.M.; Gouin, N.; Samollow, P.B.; VandeBerg, J.F.; Hixson, J.E.; Cole, S.A.; MacCluer, J.W.; Atwood, L.D. Two quantitative trait loci affect ACE activities in Mexican-Americans. *Hypertension* 2004, 43, 466–470.

15. Allinson, T.M.; Parkin, E.T.; Condon, T.P.; Schwager, S.L.; Sturrock, E.D.; Turner, A.J.; Hooper, N.M. The role of ADAM10 and ADAM17 in the ectodomain shedding of angiotensin converting enzyme and the amyloid precursor protein. *Eur. J. Biochem.* 2004, 271, 2539–2547.
16. Webers, M.; Yu, Y.; Eyll, J.; Vanderliek-Kox, J.; Schun, K.; Michely, A.; Schumertl, T.; Garbers, C.; Dietrich, J.; Jonigk, D. D.; et al. The metalloproteinase ADAM10 sheds angiotensin-converting enzyme (ACE) from the pulmonary endothelium as a soluble, functionally active convertase. *FASEB J.* 2024, 38, e70105. doi:10.1096/fj.202402069R
17. Yu, Y.; Babendreyer, A.; Pabst, A.; Michely, A.; Martin, C.; Kühnel, M.P.; Jonigk, D.D.; Düsterhöft, S.; Ludwig, A. ADAM10 and ADAM17 differently mediate induced pulmonary ACE release by either direct proteolysis or indirect upregulation protein synthesis. *BBA - Mol. Cell Res.* 2026, 120112; *in press*.

Doc S3.

Within the control cohort summarized in Table S6, we identified one variant, rs115931022 in the *AXIN2* gene, in patient HVU439, who exhibited markedly reduced circulating ACE levels (16.1% of control). Although this variant could be formally considered a candidate modifier, its modest negative beta value (−0.385) is unlikely to account for such a pronounced reduction in ACE levels. This observation suggests that non-coding variants affecting ACE transcription, such as promoter-region mutations not captured by WES, may underline the phenotype in this individual.

Two additional variants – rs187885262 in *CFAP92* and rs189416564 in *FMNL2* – were detected in patient LAV316 (Table S6), who exhibited elevated circulating ACE levels (227.2%). In contrast, a unique variant identified in patient JBB938 (rs146197324 in *TXNDC11*) was deemed unlikely to explain the markedly increased ACE level (309.6%), given its negative beta value (−0.411).

To further contextualize these findings, we examined exome data from all 129 control individuals with normal circulating ACE levels. This broader analysis revealed that only two variants, located in *ALPP* and *CFAP65*, consistently segregated with ACE phenotypes and therefore warrant targeted ACE phenotyping in future studies.

Finally, we investigated genotype–phenotype discrepancies in five control individuals (highlighted in Fig. 1A), in whom circulating ACE levels deviated from expectations based on genotype. Among these, patient XQQ153 (61.2% of control ACE levels) carried the DD genotype and harbored a single Icelandic variant, rs144679292 in *ZC3H13*, which exhibits a negative beta value (−0.564) (Table S6).

Table S1. Mutations (2388) that significantly influenced blood ACE levels (“Iceland’s list”). (GWAS summary statistics for blood ACE levels with different mutations).

Gene	rsID	Effect	UNIPROT	Protein position	AA subs.	Beta value	P value	Poly-Phen-2	ImpMAF /100 000	MAF dbSNP /100 000
AAAS	rs138043864	ms	Q9NRG9	37	D/N	-0,497	0,049174	0,018	35	1,5
AATF	rs761744080	ms	Q9NY61	221	V/G	0,816	0,018534	0,856	14	0,4
AATF	rs202139717	ms	-	178	S/N	-0,465	0,001559	0,003	67	8,7
ABCA1	rs138880920	ms	O95477	776	K/N	0,395	0,043339	0,986	46	202
ABCA10	rs773731325	ms	Q8WWZ4	1402	E/A	-1,195	0,014695	0,942	12	0
ABCA13	rs764466926	ms	Q86UQ4	4182	T/I	0,417	0,045727	0,006	33	0,7
ABCA13	rs1562947824	ms	-	2254	V/L	-0,626	0,046415	0,003	13	0
ABCA13	rs202134320	ms	-	4046	V/I	-0,879	0,010743	0,000	17	9,4
ABCA6	rs199889249	ms	Q8N139	296	G/V	0,414	0,000036	0,988	150	30
ABCA8	rs749671878	ms	O94911	996	M/I	-1,088	0,045480	0,003	15	0,4
ABCA8	rs4147979	ms	-	331	G/S	-0,648	0,035039	0,386	13	24
ABCA8	rs1567829652	ms	-	1079	L/F	0,455	0,022878	0,031	43	0
ABCB1	7:87544848:CT/G	fs	P08183	680	R/X	-0,797	0,029990	1,000	12	N/A
ABCB1	rs149196148	ms	-	388	I/S	0,620	0,000816	0,993	81	1,9
ABCB5	rs778503529	ms	Q2M3G0	620	V/L	0,362	0,038571	0,027	55	0
ABCC1	rs182118381	ms	P33527	1103	G/D	-0,696	0,007635	1,000	27	130
ABCC2	rs766399139	ms	Q92887	1064	I/N	0,535	0,046997	0,975	16	0,4
ABCC3	rs151079073	ms	O15438	436	Q/K	0,740	0,024662	0,997	13	112
ABCC3	rs748107210	ms	-	1168	R/W	-0,438	0,017761	0,806	62	0,8
ABCF2	rs1563617859	ms	Q9UG63	139	P/S	-1,281	0,030709	0,993	14	0
ABCG1	rs750210537	ms	P45844	228	A/V	0,725	0,000721	0,695	39	1,1
ABCG4	rs1320169801	ms	Q9H172	631	R/Q	-1,157	0,000822	0,968	16	0,4
ABL1	rs370992010	ms	P00519	1021	R/Q	-0,424	0,019859	0,705	57	1,1
ABL2	rs745638514	ms	P42684	4	G/E	-0,963	0,004482	0,998	18	0,4
ABL2	rs148985886	ms		608	P/S	-0,769	0,027151	0,062	10	79
ABLIM2	rs752430305	ms	Q6H8Q1	20	T/M	-0,395	0,029098	0,577	43	8,3
ABO	rs8176747	ms	P16442	267	G/A	0,350	9,3E-105	0,003	6,497	10589
ABO	rs8176746	ms	-	265	L/M	0,350	8,4E-105	0,090	6,496	10589
ABO	rs8176743	ms	-	234	G/S	0,351	5,4E-105	0,168	6,488	10587
ABRACL	rs768476146	ms	Q9P1F3	74	V/I	-0,368	0,041042	0,065	46	0,8
ACACA	rs748364574	ms	Q13085	1943	V/M	-0,849	0,040345	0,015	17	1,5
ACAN	rs764412011	ms	P16112	1619	A/P	-0,394	0,005607	0,020	97	0,4
ACAN	rs548481167	ms	-	2347	E/K	0,676	0,024650	0,001	24	23
ACAP1	rs746367494	ms	Q15027	217	R/Q	0,458	0,009814	0,455	50	2,3
ACE	rs750712925	ms	P12821	45	G/R	-1,288	6,83E-15	0,142	82	1,9
ACE	rs757694144	ms	-	482	R/P	-1,349	1,5E-07	0,246	34	0,4
ACE	rs141186617	ms	-	305	N/I	-0,692	0,022607	0,017	18	11
ACE	rs141543325	ms	-	228	R/C	0,933	0,007057	1,000	17	30
ACE	rs372416620	ms	-	1243	V/I	0,371	6,66E-09	0,061	469	5,7
ACE	rs571848794	ms	-	1013	G/S	-0,468	0,001102	0,183	93	6,8
ACE	rs3730025	ms	-	244	Y/C	-1,217	3,3E-291	0,998	1,534	924
ACKR1	rs529272627	ms	Q16570	124	R/L	0,416	0,007551	0,000	74	5,8
ACLY	rs750779834	ms	P53396	296	G/V	0,364	0,000331	0,437	159	3,0
ACOX3	rs759828894	ms	O15254	22	D/H	0,625	0,002260	0,067	31	2,6
ACP4	rs745993743	ms	Q9BZG2	42	G/S	-0,363	0,024882	1,000	69	0,6
ACR	rs1445899395	ms	P10323	368	P/S	0,784	0,024021	0,015	24	1,4
ACRBP	rs760419396	ms	E7EP66	162	R/C	-0,706	0,032420	0,679	15	0,4
ACSL3	rs760062740	ms	O95573	632	L/V	0,688	0,037619	0,634	11	0,3
ACSS3	rs61745251	ms	Q9H6R3	163	H/Y	0,508	0,005001	0,001	55	5548
ACTR1A	rs746393164	ms	-	340	T/M	0,381	0,032302	1,000	42	0,3
ADAM9	rs746548581	del	Q13443	704	F/-	-0,691	0,044961	-	21	1,9
ADAM11	rs776835838	ms	O75078	678	P/A	-0,467	0,016173	0,080	42	0,1
ADAM22	rs201832352	ms	Q9P0K1	630	G/E	-0,999	0,032157	0,021	12	14
ADAM29	rs755175800	ms	Q9UKF5	408	G/R	0,589	0,005401	0,918	29	0,4
ADAMDEC1	rs1207275267	ms	O15204	318	R/C	1,294	0,013085	0,970	10	0,4
ADAMTS10	rs144596955	ms	Q9H324	669	V/L	0,742	0,032795	0,003	11	29
ADAMTS13	rs145825553	ms	Q76LX8	421	R/C	0,394	2,45E-08	0,994	334	45
ADAMTS13	rs748223519	ms	-	439	E/K	-0,891	0,003181	0,205	24	0,7
ADAMTS14	rs150863733	ms	Q8WXS8	1110	S/L	-0,536	0,015959	0,000	29	41
ADAMTS17	rs146934810	ms	Q8TE56	183	K/R	-0,407	0,006986	0,000	79	221
ADAMTS18	rs148442712	ms	Q8TE60	660	S/N	0,519	0,008542	0,007	38	77
ADAMTS7	rs376434066	ms	Q9UKP4	1506	G/R	-0,918	0,005338	1,000	11	0,7
ADAMTS7	rs61754850	ms	-	126	P/L	0,539	0,025395	0,028	24	192
ADAMTS9	rs75938827	ms	Q9P2N4	1912	V/I	0,356	0,005406	0,000	96	192
ADAMTSL1	rs758299352	ms	Q8N6G6	387	A/T	-0,516	0,029702	0,802	32	1,5
ADAMTSL5	rs142558769	ms	Q6ZMM2	432	R/H	1,494	0,010884	0,995	15	411
ADAR	rs1557881072	ms	P55265	601	T/I	-0,593	0,027836	0,000	21	0
ADAT1	rs147999655	ms	Q9BUB4	417	Q/R	0,492	0,018278	0,953	32	33
ADCY2	rs1560953294	ms	Q08462	668	S/T	-0,351	0,046270	0,198	50	0,2
ADCY2	rs765899703	ms	-	129	G/S	0,471	0,044594	0,177	25	0
ADGRA2	rs749263093	ms	Q96PE1	386	T/I	-1,077	0,006264	0,521	12	0,1
ADGRA3	rs756147612	ms	Q8IWK6	955	P/T	-0,395	0,000399	0,274	111	2,3

ADGRB3	rs147658947	ms	O60242	730	D/H	0,444	0,012257	**0,842**	**47**	0,4
ADGRE1	rs372416153	ms	Q14246	655	G/S	-0,578	0,036681	**0,761**	**20**	5,3
ADGRF4	rs755972498	ms	Q8FZF3	136	R/H	-0,878	0,001113	0,003	**21**	5,4
ADGRG6	rs191332808	ms	Q86SQ4	575	V/A	0,403	0,022701	0,003	**50**	**15**
ADORA2B	rs1567785802	ms	P29275	219	M/K	0,384	0,024868	0,001	**44**	0
ADORA2B	rs759669984	ms	-	8	A/E	-0,399	0,041289	0,077	**65**	0,7
ADPRHL1	rs532183571	ms	Q8NDY3	1195	G/D	0,498	0,044893	**0,970**	**21**	0
ADRA2B	rs200889434	ms	P18089	358	R/H	0,584	0,050762	**0,953**	**20**	**13**
AEN	rs140747161	ms	Q8WTP8	19	I/F	0,370	0,003689	0,031	**91**	**12**
AEN	rs775685764	ms	-	131	R/C	0,725	0,033441	**0,958**	**24**	3,4
AFAP1L2	rs147799118	ms	Q8N4X5	381	H/R	0,512	0,043082	**0,759**	**29**	**13**
AFAP1L2	rs561584886	ms	-	218	V/M	-0,697	0,016007	**0,942**	**22**	0,4
AFF3	rs897323624	ms	P51826	801	P/L	0,784	0,046995	0,001	**13**	3,8
AFTPH	rs3770740	ms	Q6ULP2	301	E/K	**-1,384**	0,001161	0,006	**18**	**12911**
AGBL3	rs753584513	ms	Q8NEM8	878	L/I	0,613	0,020071	**0,955**	**22**	0,4
AGFG2	rs749263005	ms	O95081	241	P/A	0,833	0,019771	-	**14**	0,7
AGMAT	rs745384069	ms	Q9BSE5	285	A/V	0,600	0,041364	**0,987**	**19**	4,9
AGRN	rs149159118	ms	O00468-6	1118	T/K	0,700	0,020151	0,413	**21**	152
AGT	rs369425934	ms	P01019	118	V/M	0,446	0,040524	0,005	**34**	4,9
AGTPBP1	rs547428998	ms	Q9UPW5	691	R/C	-0,370	0,003025	**0,720**	121	1,5
AGXT	rs121908529	ms	P21549	170	G/R	-0,701	0,033683	**0,986**	**18**	**53**
AHCTF1	1:246850185:T/C	ms	Q8WYP5	1941	R/G	-0,545	0,032890	0,212	**15**	N/A
AHDC1	rs1557659784	ms	Q5TGY3	1063	V/I	-0,656	0,006647	0,097	**27**	0
AHNAK2	rs767586966	ms	Q8IVF2	680	F/Y	-0,683	0,029822	**0,731**	**18**	1,9
AHNAK2	rs772834815	ms	-	5598	Q/H	-0,652	0,015460	**0,737**	**21**	0,2
AHNAK2	rs1566908248	ms	-	2573	S/R	0,640	0,016098	**0,514**	**25**	0
AHNAK2	rs202107671	ms	-	661	Q/K	0,361	0,042874	0,041	**49**	**19**
AIFM2	rs771695112	ms	Q9BRQ8	244	G/S	-0,857	0,006595	**0,993**	**18**	0,8
AIP	rs145047094	ms	O00170	16	R/H	-0,677	0,040426	**0,615**	**23**	171
AKAP12	rs199947814	ms	Q02952	1621	T/A	0,737	0,014305	0,000	**13**	**12**
ALDH1B1	rs1821278295	ms	P30837	135	Y/C	0,462	0,044309	0,030	**28**	0,2
ALDH1B1	rs142427338	stop	-	378	Q/*	0,399	0,026535	**1,000**	**43**	102
ALDH1L1	rs149080804	ms	O75891	107	P/L	-0,452	0,029996	**0,970**	**36**	**71**
ALDH3B1	rs369689965	ms	P43353	431	R/C	-0,560	0,044907	**0,997**	**20**	1,1
ALDH3B2	rs144564033	ms	P48448	148	A/T	0,510	0,037128	**0,981**	**37**	**14**
ALDH9A1	rs1065756	ms	P49189	221	S/T	0,514	0,026897	0,000	**30**	**2041**
ALMS1	rs199573929	ms	Q8TCU4	679	Y/C	-0,754	0,030229	0,123	**23**	148
ALMS1	rs200266868	ms	-	1821	P/L	-0,796	0,021100	**0,962**	**11**	**82**
ALOX5AP	rs201182270	ms	P20292	117	R/C	-0,469	0,039286	**1,000**	**27**	0,8
ALPI	rs751686908	ms	P09923	212	N/D	-0,498	0,038468	**0,998**	**34**	1,9
ALPK3	rs767926913	ms	Q96L96	1107	Q/E	0,878	0,038700	0,038	**11**	8,3
ALPK3	rs139666355	ms	-	1134	A/V	-0,928	0,046406	0,007	**12**	**92**
ALPP	rs2981374	ms	P05187	501	P/H	**1,097**	0,012381	0,000	**17**	0,8
ALX3	rs975515598	ms	Q95076	100	A/D	0,992	0,004368	**0,699**	**12**	0
AMBN	rs781315593	ms	Q9NP70	299	G/S	-0,972	0,012688	**0,980**	**14**	0
AMT	rs201189946	ms	P48728	379	E/A	0,436	0,024511	**0,864**	**47**	**19**
AMY1C	rs1430206652	ms	P0DTE8	9	T/S	-0,583	0,009503	0,000	**33**	0
AMY2B	rs753265856	ms	P19961	404	R/H	0,755	0,002053	0,209	**24**	3,0
ANAPC11	rs756571746	ms	Q9NYG5	71	Q/H	-0,390	0,042152	0,062	**73**	0,9
ANAPC13	rs756834266	ms	Q9BS13	18	A/V	-0,455	0,017991	**0,548**	**44**	0,9
ANGEL2	1:213013315:T/C	ms	Q5VTE6	55	M/V	0,477	0,051281	0,007	**23**	N/A
ANGPTL4	rs140744493	ms	Q9BY76	336	R/C	0,468	0,016221	**0,980**	**45**	246
ANKFN1	rs773925251	ms	Q8N957	120	T/A	0,812	0,026185	0,013	**12**	1,9
ANKFN1	rs770360843	ms	-	14	N/I	0,755	0,016978	0,000	**18**	0
ANKFY1	rs763606938	ms	Q9P2R3	1131	R/H	0,682	0,024838	**0,999**	**19**	0,1
ANKFY1	rs1567972969	ms	-	15	L/H	**-1,160**	0,002731	**0,998**	**14**	0
ANKHD1	rs745822360	ms	Q8IWZ3	2151	T/A	0,376	0,006643	**0,511**	**76**	0,4
ANKRD12	rs778977438	ms	Q6UB98	89	W/C	-0,824	0,017677	**0,625**	**16**	4,3
ANKRD20A1	rs1379856671	ms	Q5TYW2	403	I/K	0,617	0,007206	0,098	**25**	0,4
ANKRD27	rs372391792	ms	Q96NW4	944	S/L	0,541	0,019908	0,017	**31**	2,6
ANKRD30A	rs772253614	ms	Q9BXX3	235	D/G	-0,368	0,039127	**0,637**	**46**	1,5
ANKRD34B	rs200804923	ms	A5PLL1	282	N/S	-0,374	0,002712	0,006	126	**26**
ANKRD62	18:12125936:A/C	ms	A6NC57	705	Q/H	-0,736	0,000679	**0,788**	**33**	N/A
ANKS1A	rs986675244	ms	Q92625	438	M/V	**-1,015**	0,050749	0,000	**13**	0,4
ANO6	rs372908985	ms	Q4KMQ2	579	Y/S	-0,549	0,027306	**0,730**	**29**	4,5
ANO7	rs138809031	ms	Q6IWH7	54	R/W	-0,542	0,044076	0,226	**19**	**18**
ANTXRL	rs750794572	ms	A6NF34	6	S/C	-0,380	0,023433	**0,791**	**69**	4,7
ANXA2R	rs756512520	ms	Q37CQ2	94	P/S	-0,505	0,001979	**0,982**	**66**	0,2
ANXA4	rs2228202	ms	P09525	137	S/R	**-1,320**	0,028467	**0,718**	**15**	**9410**
AOC3	rs145964446	ms	Q16853	272	D/N	0,541	0,041555	0,003	**18**	**12**
AP2A2	rs200859967	ms	O94973	658	S/L	-0,500	0,046662	**0,883**	**43**	**25**
AP2B1	rs749754793	ms	P63010	235	R/G	-0,665	0,002945	0,263	**30**	4,7
AP3D1	rs25673	ms	O14617	1134	I/L	-0,537	0,038390	0,007	**26**	**11762**
AP4B1	rs200590674	ms	Q9Y6B7	415	C/Y	-0,615	0,050783	**0,673**	**18**	**24**
APAF1	rs770595178	ms	O14727	51	Q/R	0,770	0,050745	0,003	**14**	0,4
APC	rs34919187	ms	P25054	2274	A/V	-0,545	0,031215	0,005	**17**	**94**
APCDD1	rs200939423	ms	Q8J025	349	R/H	-0,565	0,029242	0,048	**30**	**13**
APCDD1L	rs145873693	ms	Q8NCL9	330	V/M	**1,311**	0,002047	0,069	**21**	**67**
APOA1	rs138407155	ms	P02647	95	F/Y	0,450	0,010564	**0,904**	**63**	**47**
APOA5	rs747958115	ms	Q6Q788	211	R/L	0,961	0,024496	0,238	**15**	3,8

**APOA5**	rs201201147	ms	-	321	H/L	-0,790	0,016733	0,771	12	15
APOB	rs374473614	stop	P04114	725	Q/*	1,016	0,005874	1,000	19	0,4
APOBEC3D	22:39031830:T/C	ms	Q96AK3	300	V/A	0,603	0,019377	0,962	16	N/A
APOL1	rs73885316	ms	O14791	264	N/K	0,937	0,001830	0,572	21	839
APPL2	rs775024469	ms	Q8NEU8	532	M/K	-0,899	0,005984	0,014	17	0
ARAP2	rs1560721800	ms	Q8WZ64	190	T/A	-1,043	0,041357	0,001	16	0
ARFGEF3	rs145180404	ms	Q5TH69	691	S/P	-0,366	0,018544	0,000	48	33
**ARHGAP32**	rs746664458	ms	A7KAX9	1202	G/R	-0,459	0,001278	0,308	71	0,5
**ARHGAP32**	rs377531423	ms	-	1957	R/S	0,400	0,008489	0,996	64	9,1
ARHGAP35	rs373252908	ms	Q9NRY4	577	R/W	0,645	0,042049	0,943	22	1,1
ARHGDIA	rs150457411	ms	P52565	155	E/K	-0,810	0,025235	-	13	7,2
ARHGEF11	rs766422545	ms	O15085	520	M/V	0,542	0,044379	0,070	23	1,1
ARHGEF16	rs745895895	ms	Q5VV41	147	R/W	-0,532	0,041445	0,995	33	1,5
ARHGEF28	rs756796241	ms	Q8N1W1	593	P/R	0,696	0,026927	0,305	25	0,4
ARHGEF40	rs892869054	ms	Q8TER5	695	R/W	-0,474	0,029272	0,982	28	0,8
ARHGEF5	rs2053728428	ms	Q12774	1306	L/F	-0,680	0,040086	0,998	21	0
ARID5B	rs199637139	ms	Q14865	901	T/M	-1,574	0,008946	-	12	2,0
ARL11	rs779514414	ms	Q969Q4	129	A/T	0,665	0,014751	0,999	14	0,6
ARPC5L	rs367615280	ms	Q9BPX5	99	S/T	-0,743	0,010944	0,010	28	2,6
ARPP19	rs541368973	ms	P56211	29	E/A	0,575	0,039796	0,011	14	0,6
ART1	rs199916833	stop	P52961	29	R/*	-0,574	0,002220	1,000	39	16
AS3MT	rs200488263	ms	Q9HBK9	117	Y/H	0,573	0,011871	0,108	17	14
ASAP3	rs140136454	ms	Q8TDY4	717	A/D	0,449	0,001145	-	93	527
ASB14	rs202057906	ms	A6NK59	386	P/L	0,549	0,048831	0,998	18	8,7
ASB16	rs755814254	ms	Q96NS5	350	P/L	0,506	0,020507	0,391	32	6,0
ASB3	rs760580086	ms	Q9Y575	140	G/R	0,792	0,044430	0,803	22	0,4
ASGR2	rs377144273	ms	P07307	116	V/M	0,652	0,013707	0,106	28	13
ASIC1	rs746957755	ms	P78348	253	D/N	0,459	0,049083	0,019	20	0,4
ASPHD2	rs140657637	ms	Q6ICH7	53	V/M	0,553	0,020682	0,212	31	2,3
ASPM	rs1557956556	ms	Q8IZT6	1117	M/V	1,062	0,041921	0,970	19	0,4
ASXL3	rs201152513	ms	Q9C0F0	1140	P/Q	-0,495	0,004076	0,382	84	3,8
ATAD2	rs746856878	ms	Q6PL18	1339	T/P	0,722	0,012543	0,085	19	0
ATAD5	rs112921454	ms	Q96QE3	307	E/K	0,419	0,037253	0,162	45	95
ATAT1	rs150614928	ms	Q5SQI0	313	G/V	0,453	0,030066	0,151	57	678
ATCAY	rs147684273	ms	Q86WG3	124	V/M	0,906	0,013274	0,382	15	5,7
ATF7IP	rs777624434	ms	Q6VMQ6	750	T/A	-0,731	0,020773	0,003	14	0,8
ATG9A	rs779637846	ms	Q7Z3C6	731	H/Y	0,457	0,044702	0,934	29	0,4
ATP12A	13:24706450:G/A	ms	P54707	719	G/D	-0,647	0,049475	0,994	10	N/A
ATP1A4	rs377713967	ms	Q13733	1019	P/L	0,368	0,021009	0,928	69	35
ATP2C2	rs763173008	ms	O75185	567	T/M	-0,713	0,001096	0,308	35	0,8
ATP5IF1	rs758401235	ms	Q9UII2	79	V/F	0,603	0,016526	0,058	17	0
ATP5PB	rs151251385	ms	P24539	208	R/C	0,616	0,035845	0,994	15	4,3
ATP6V0A1	rs141898946	ms	Q93050	774	V/M	0,522	0,019485	0,272	22	39
ATP6V0E2	rs761485568	ms	Q8NHE4	46	V/I	-0,444	0,024509	0,174	57	0,8
ATPAF1	rs757491853	ms	Q5TC12	245	E/Q	-0,451	0,007595	0,937	65	0,1
ATRAID	rs147821107	ms	Q6UW56	114	R/C	-0,393	0,050338	0,830	49	10
ATRN	rs775375659	ms	O75882	820	N/S	0,538	0,006562	0,025	45	30
ATRNL1	rs1229952017	ms	Q5VV63	971	H/Y	1,074	0,003547	0,000	12	0,8
ATXN2	rs7969300	ms	Q99700	88	S/N	-0,726	0,009207	0,036	37	8372
AXIN2	rs115931022	ms	Q9Y2T1	412	N/S	-0,385	0,002435	0,000	124	494
B3GNTL1	rs201686706	ms	Q67FW5	203	G/S	0,586	0,027334	0,001	23	0,7
BAG2	rs573834496	ms	O95816	181	I/T	-0,360	0,044023	0,532	71	4,5
BAG6	rs755726048	ms	P46379	634	D/H	-0,369	0,048306	0,811	47	1,9
BARD1	rs142155101	ms	Q99728	761	S/N	-0,574	0,023373	0,005	25	73
**BAZ1A**	rs759984179	ms	Q9NRL2	1372	P/T	0,733	0,026105	0,981	11	1,4
**BAZ1A**	rs1566551387	ms	-	1078	H/R	0,758	0,015930	0,033	12	0,4
BBIP1	rs1473657985	ms	A8MTZ0	45	I/M	1,050	0,007087	0,581	11	0,8
BCAS3	rs771742994	ms	Q9H6U6	480	S/I	-1,162	2,19E-12	0,991	88	6,4
BCHE	rs116097205	ms	P06276	61	Y/C	0,863	0,033205	1,000	12	0,8
**BCKDHA**	rs779476349	ms	P12694	31	R/W	-0,753	0,009169	0,245	19	0
**BCKDHA**	rs11549936	ms	-	39	P/R	-0,662	0,044499	0,000	16	8739
BCO1	rs139655280	ms	Q9HAY6	288	D/N	0,406	0,011986	0,265	62	6,0
BDKRB1	rs757505732	ms	P46663	219	V/I	0,662	0,017464	0,005	22	1,1
BEAN1	rs755281092	ms	Q3B7T3	42	V/I	-0,469	0,034902	0,972	30	9,8
BEST3	rs199554316	ms	Q8N1M1	211	M/I	-0,374	0,023061	0,129	51	29
BICD1	rs200717707	ms	Q96G01	397	R/W	0,440	0,025753	0,916	44	0,8
BIN3	rs746437989	ms	Q9NQY0	233	R/W	-0,532	0,009151	0,676	40	1,3
BIRC6	rs1353906962	ms	Q9NR09	4009	G/E	0,870	0,041127	0,997	10	0,4
**BIRC7**	rs149161695	ms	Q96CA5	206	R/T	0,369	0,019169	0,005	60	22
**BIRC7**	rs201155031	ms	-	245	R/Q	-1,306	0,000911	0,000	12	1,9
BLTP2	rs200477170	ms	Q14667	36	R/W	-0,770	0,025382	0,091	16	1,5
**BMAL1**	rs769220676	ms	O00327	58	H/N	-0,772	0,025251	0,051	14	0,2
**BMAL1**	rs777322872	ms	-	46	Y/C	0,471	0,027682	0,874	27	1,5
BMP3	rs61729826	ms	P12645	345	K/N	-0,590	0,023115	0,998	24	1103
BMS1	rs117641896	ms	Q14692	864	A/T	1,062	0,003955	0,054	20	566
BOC	rs78524315	ms	Q9BWV1	534	R/C	0,513	0,008626	0,566	40	35
BOLA2	16:29454204:T/G	ms	Q9H3K6	17	E/A	-0,453	0,025124	0,010	29	N/A
BOP1	rs1160618575	ms	Q14137	673	P/R	-1,512	0,003735	0,015	11	0
BORCS5	rs565487491	ms	Q969J3	135	R/C	0,386	0,021797	0,989	54	12
BPIFA2	rs1075435	ms	Q96DR5	236	V/L	-1,106	0,033823	0,000	15	8747

BPTF	rs780473362	ms	Q12830	1622	K/E	0,451	0,012177	0,309	54	3,8
BPTF	rs748420257	ms	-	240	N/S	-1,662	0,031778	0,043	13	0
BRD2	rs34530779	ms	P25440	569	A/T	0,456	0,001069	-	99	610
BRD3	rs201916092	ms	Q15059	429	A/V	-0,399	0,050012	-	40	0,4
BRME1	rs769178284	ms	Q0VDD7	519	S/Y	-0,430	0,031732	0,788	40	4,2
BSN	rs749714827	ms	Q9UPA5	1867	M/L	-0,429	0,006319	0,001	62	0,4
BSN	rs148125466	ms	-	355	A/T	-0,397	0,048019	0,958	43	289
BTBD17	rs145160027	ms	AGNE02	117	D/E	0,484	0,033120	0,042	45	80
BTBD17	rs752498641	ms	-	117	D/N	-0,538	0,014078	0,869	30	1,1
BTBD8	rs778129490	ms	Q5XKL5	1263	K/R	-0,445	0,003843	0,999	83	0
BYSL	rs368935062	ms	Q13895	25	A/G	-0,387	0,020314	0,003	60	0,7
BYSL	rs754194109	ms	-	364	F/L	-0,519	0,000570	0,994	59	0,3
C10orf95	rs755941591	ms	Q9H7T3	171	T/A	-0,560	0,002708	0,129	54	26
C11orf58	rs771370191	ms	O00193	155	E/K	0,950	0,001622	0,807	10	1,1
C12orf43	rs141346965	ms	Q96C57	168	A/V	0,561	0,001704	0,983	46	40
C12orf60	rs140864709	ms	Q5U649	56	I/F	0,565	0,000894	0,085	47	226
C13orf42	rs529205663	ms	A0A1B0GVH6	154	E/K	0,810	0,039636	0,011	14	4,3
C14orf39	rs1566665407	ms	Q8N1H7	369	K/Q	0,747	0,009816	0,440	16	0
C15orf39	rs1567139783	ms	Q6ZRI6	629	P/A	0,920	0,030618	0,000	17	0,1
C16orf96	rs1567456672	ms	A6NNT2	907	F/Y	-0,590	0,013213	0,970	26	0
C18orf54	rs1568181806	ms	Q8IYD9	192	N/D	-0,548	0,040945	0,306	16	0,2
C19orf67	rs553160046	ms	A6NJJ6	65	T/M	0,793	0,043969	0,000	14	14
C1orf159	rs139486403	ms	Q96HA4	304	D/E	-0,845	0,022047	0,631	15	34
C1QTNF9B	rs371732332	ms	B2RNN3	69	T/A	0,553	0,003163	0,000	50	20
C1R	rs200899486	ms	P00736	294	D/N	-0,384	0,040329	0,012	40	11
C20orf204	rs557949001	ms	A0A1B0GTL2	110	G/R	-0,368	0,017654	0,788	67	139
C2CD2L	rs140064567	ms	O14523	112	E/K	0,434	0,002238	0,111	105	92
C2orf92	rs143227941	fs	A0A1B0GVN3	246	E/X	0,414	0,024967	1,000	54	5084
C2orf92	rs17029878	ms	-	188	Q/L	0,414	0,024967	0,617	54	5091
C3orf20	rs750004016	stop	Q8ND61	606	R/*	-0,470	0,016256	1,000	40	1,4
C3orf70	rs777199280	ms	A6NCL5	110	S/P	0,827	0,051272	0,000	16	2,3
C4A	6:31992420:G/A	ms	P0C0L4	582	R/Q	0,571	0,048817	0,017	29	N/A
C4B	6:32025158:G/A	ms	P0C0LS	582	R/Q	0,571	0,048817	0,057	29	N/A
C4orf3	rs190403527	ms	Q8WVX3	24	G/R	0,718	0,051515	0,000	13	87
C5AR1	rs200740207	ms	P21730	44	F/L	0,475	0,039870	0,000	34	11
C6	rs778566926	ms	P13671	593	A/V	-0,697	0,003610	0,791	33	0,8
C8A	rs142382705	ms	P07357	207	R/W	-1,172	0,046057	0,999	10	5,4
CA9	rs1563923396	ms	Q16790	332	T/S	-0,599	0,016175	1,000	17	0,1
CABP1	rs755254500	ms	Q9NZU7	66	R/C	-0,653	0,012227	0,976	31	0,7
CACNA1C	rs749190892	ms	Q13936	1704	G/D	0,362	0,001148	0,762	103	0
CACNA1C	rs121912775	ms	-	490	G/R	-0,595	0,039862	0,795	16	44
CACNA1G	rs368939625	ms	O43497	1574	D/Y	-0,957	0,032026	0,426	10	3,3
CACNA1H	rs774565789	ms	O95180	281	T/M	1,508	0,000317	0,830	10	2,3
CACNB1	rs141322943	ms	Q02641	233	G/S	0,721	0,015278	0,000	19	322
CADM4	rs112876885	ms	Q8NFZ8	159	Y/H	-0,489	0,010187	0,802	37	536
CADPS	rs755251601	ms	Q9ULU8	32	R/L	0,382	0,003534	0,000	116	1,1
CADPS2	rs1563339160	ms	Q86UW7	787	M/V	-0,763	0,038445	-	13	0
CAMSAP1	rs748961061	ms	Q5T5Y3	76	P/L	0,420	0,023329	0,999	57	0,8
CAMSAP1	rs758753847	ms	-	1592	P/L	-0,474	0,016720	-	48	0,4
CAMTA1	rs141259598	ms	Q9Y6Y1	707	S/C	-0,428	0,036325	0,441	42	48
CAND2	rs763105870	ms	O75155	575	L/R	-0,899	0,007464	0,300	15	0,4
CAPN10	rs200682095	ms	Q9HC96	340	R/Q	0,824	0,012042	0,007	21	14
CAPN12	rs147906486	ms	Q6ZSI9	211	V/M	-0,362	0,004801	0,927	86	56
CAPN2	rs745412257	stop	P17655	612	R/*	0,363	0,048989	1,000	37	1,1
CAPN3	rs1414687788	ms	P20807	248	M/L	-0,828	0,051781	0,001	11	0,8
CAPNS2	rs146637108	ms	Q96L46	125	R/Q	-0,589	0,045470	0,962	16	164
CAPZA3	rs146799170	ms	Q96KX2	154	C/F	-1,019	0,009792	0,998	13	178
CARD10	rs1569161077	ms	Q9BWT7	1030	S/I	1,304	0,005090	0,774	19	0,6
CARD6	rs1561216545	ms	Q9BX69	603	K/T	-0,635	0,043358	0,883	23	0
CARD6	rs148780446	ms	-	857	S/L	0,875	0,011883	0,000	17	257
CASC3	rs768468747	ms	Q15234	271	P/L	0,685	0,005181	0,077	21	2,7
CASD1	rs1562947072	ms	Q96PB1	494	L/V	0,650	0,010219	0,434	30	0
CASKIN2	rs150397249	ms	Q8WXE0	410	V/M	-0,718	0,002670	0,114	32	35
CASP14	rs368748073	ms	P31944	90	G/R	-0,454	0,022962	1,000	35	2,6
CASP4	rs56008239	ms	P49662	344	R/Q	-1,008	0,004577	0,167	16	23
CASZ1	rs1194855522	ms	Q86V15	1171	A/V	-0,688	0,009429	0,972	19	0,7
CATSPER4	rs371089081	ms	Q7RTX7	205	R/H	0,599	0,049872	0,145	13	2,8
CATSPER4	rs778169515	ms	-	73	A/T	-0,622	0,020198	0,003	15	4,0
CAVIN3	rs1228057325	ms	Q969G5	83	A/V	0,711	0,023820	0,972	21	0,7
CBARP	rs771515759	fs	Q8N350	407	P/X	-0,789	0,029755	1,000	14	65
CBARP	rs777136090	ms	Q8N350	429	A/G	0,507	0,038990	0,000	37	12
CBFA2T3	rs201916925	ms	O75081	447	G/S	-0,374	0,005197	-	88	11
CBLIF	rs748783434	ms	P27352	266	M/R	-0,508	0,022442	0,949	39	0,7
CCDC110	rs141263491	ms	Q8TBZ0	540	M/V	0,483	0,049447	0,014	38	27
CCDC15	rs371930839	ms	Q0P6D6	121	S/L	0,801	0,021411	0,194	18	11
CCDC17	rs766641176	ms	Q96LX7	231	Y/H	0,983	0,002674	0,665	13	0,7
CCDC172	10:116378454:G/A	ms	P0C7W6	229	D/N	-0,677	0,040231	0,511	10	N/A
CCDC178	rs140513669	ms	Q5BJE1	504	R/H	0,434	0,050775	0,000	31	8,7
CCDC18	rs774207938	ms	Q5T9S5	915	E/Q	-0,638	0,014526	0,171	32	1,1
CCDC181	rs137855652	ms	Q5TID7	167	E/G	-0,398	0,049541	0,574	36	49

CCDC182	rs149716379	ms	A6NF36	131	R/Q	-0,749	0,003280	0,000	32	339
CCDC182	rs769238436	ms	-	60	R/G	-0,554	0,049648	0,062	26	5,9
CCDC47	rs778565800	ms	Q96A33	390	I/L	0,420	0,006050	0,015	68	1,1
CCDC77	rs148861801	ms	Q9BR77	338	S/N	-0,699	0,001850	0,000	35	7,9
CCDC8	rs774700757	ms	Q9H0W5	268	K/N	0,479	0,038348	0,752	33	0
CCDC82	rs772526152	ms	Q8N4S0	280	E/A	-0,392	0,050512	0,140	35	0,8
CCDC88C	rs763668804	ms	Q9P219	697	R/C	-0,392	0,003467	0,692	83	2,6
CCDC88C	rs769113965	ms	-	82	V/L	-0,365	0,010916	0,176	73	0,8
CCDC9	rs201241449	ms	Q9Y3X0	230	R/W	0,647	0,034373	0,000	19	92
CCER1	rs377744456	ms	Q8TC90	164	A/V	0,410	0,049208	0,044	47	4,2
CCER2	rs778686851	ms	I3L3R5	74	A/V	-0,897	0,004277	0,000	24	1,9
CCER2	rs181740944	ms	-	209	R/H	0,461	0,027214	0,000	36	235
CCM2	rs747384033	ms	Q9BSQ5	15	S/L	-0,485	0,001587	0,514	51	0,4
CCN4	rs752881842	ms	O95388	365	I/T	-0,705	0,024856	0,893	15	4,9
CCNG2	rs1560422355	ms	Q16589	152	E/Q	0,458	0,044025	0,036	20	0
CCNI	4:77055296:T/G	ms	Q14094	182	T/P	0,523	0,030330	0,929	20	N/A
CCR4	rs1410395535	ms	P51679	89	V/M	1,068	0,000605	0,999	21	0,4
CCR7	rs143854738	ms	P32248	359	R/Q	0,760	0,025907	0,192	18	0,4
CCT8	rs776634991	ms	P50990	4	H/R	-0,363	0,047644	0,006	50	0,4
CCT8	rs766748030	ms	-	459	K/E	-0,363	0,047655	0,763	50	0
CCT8L2	rs149623775	ms	Q96SF2	133	R/Q	-0,432	0,015507	0,366	51	99
CD200R1	rs200744866	ms	Q8TD46	277	I/V	0,600	0,017731	0,017	19	8,3
CD300E	rs772510010	ms	Q496F6	117	R/C	-0,625	0,015550	0,946	19	0,9
CD3E	rs148647954	ms	P07766	71	D/H	0,398	0,006876	0,000	94	49
CD44	rs762054400	ms	P16070	304	I/V	0,994	0,019366	-	15	0,2
CD53	rs150734121	ms	P19397	165	R/Q	0,764	0,004364	0,000	25	65
CD81	rs538164293	ms	P60033	152	E/Q	0,376	0,022388	0,820	53	0,8
CD93	rs1000470607	ms	Q9NPY3	409	C/Y	-0,453	0,015333	0,998	36	0,8
CD93	rs1050339456	ms	-	249	D/N	0,565	0,014834	0,162	27	2,0
CDA	rs758866204	ms	P32320	38	V/A	-0,422	0,034594	1,000	32	0,4
CDA	rs752974013	ms	-	38	V/L	-0,422	0,034594	0,876	32	0,4
CDC14A	rs755456667	ms	Q9UNH5	609	P/L	0,365	0,044713	0,130	53	2,3
CDC23	5:138202147:TT/G	fs	Q9UJX2	127	E/X	0,377	0,020556	1,000	47	N/A
CDC45	rs1568931035	ms	O75419	305	V/L	-0,789	0,044752	0,001	16	0
CDCP2	rs150513591	ms	Q5VXM1	180	P/S	-0,970	0,022753	0,000	11	19
CDH13	rs190049290	ms	P55290	565	A/S	-0,620	0,038192	0,996	20	0,8
CDH23	rs74145660	ms	Q9H251	1806	D/E	-0,376	0,030683	0,057	50	882
CDH23	rs111033494	ms	-	2199	I/S	-0,983	0,020435	0,129	14	402
CDH23	rs764515216	ms	-	211	D/E	0,700	0,011638	0,013	18	1,9
CDH4	rs202190196	ms	P55283	565	T/M	0,589	0,041327	0,863	32	14
CDH8	rs1264566952	ms	P55286	524	D/N	-0,847	0,013990	0,115	22	1,1
CDHR1	rs766579684	ms	Q96JP9	223	D/N	0,914	0,008024	0,003	13	1,5
CDHR3	rs201055622	ms	Q6ZTQ4	347	K/N	0,451	0,012901	0,035	50	7,9
CDHR4	rs768814405	ms	A6H8M9	229	V/F	0,532	0,030119	0,152	18	6,5
CDK12	17:39530722:G/C	ms	Q9NYV4	1293	L/F	0,533	0,044982	0,000	28	N/A
CDK15	rs757611284	ms	Q96Q40	152	L/V	0,477	0,048545	0,999	19	3,0
CEBPD	rs935024096	ms	P49716	130	A/V	-0,350	0,023110	0,011	67	15
CEBPZ	rs757307902	ms	Q03701	206	K/R	-0,671	0,000429	0,080	37	0,5
CEMIP	rs144685141	ms	Q8WUJ3	1277	F/L	-0,593	0,040341	0,000	16	7,9
CEND1	rs538479515	ms	Q8N111	62	K/T	-0,549	0,025033	0,617	47	4,9
CENPC	rs756146915	ms	Q03188	607	D/E	0,486	0,040916	0,000	15	0
CENPF	rs775339410	ms	P49454	2591	K/E	-1,463	0,015151	0,010	14	0,2
CENPJ	rs17081389	ms	Q9HC77	55	P/A	0,874	0,026512	0,061	13	2747
CENPP	rs770185935	ms	Q6IPU0	46	N/S	0,589	0,011606	0,000	23	1,1
CEP112	rs777140697	ms	Q8N8E3	73	M/T	0,415	0,000004	0,781	195	0,4
CEP164	rs1324239157	stop	Q9UPV0	1021	Q/*	-0,909	0,021243	1,000	12	0,1
CEP290	rs765211180	ms	O15078	31	S/P	0,559	0,025991	0,351	35	0,2
CEP290	rs184018899	ms	-	557	R/H	1,030	0,048276	0,946	10	68
CEP295	rs748809577	ms	Q9C0D2	2470	R/H	0,432	0,037640	0,067	42	3,0
CERK	rs143397270	ms	Q8TCT0	384	V/I	-0,535	0,019237	0,003	27	100
CETN1	rs115842359	ms	Q12798	2	A/T	0,654	0,045462	0,131	16	272
CFAP*418*	rs369850560	ms	Q96NL8	47	A/P	0,621	0,032667	0,003	14	1,9
CFAP46	rs140205673	ms	Q8IYW2	1328	A/T	0,699	0,001589	0,000	34	205
CFAP54	rs1565919386	ms	Q96N23	1347	M/V	-0,722	0,050280	0,000	14	0,8
CFAP54	rs746538349	ms	-	1492	H/R	0,357	0,030149	0,899	61	11
CFAP57	rs769255988	ms	Q96MR6	217	T/R	-0,796	0,011386	0,029	14	0,9
CFAP65	rs773282060	ms	Q6ZU64	403	E/K	-0,614	0,031171	0,001	17	4,5
CFAP73	rs61738699	ms	A6NFT4	45	A/T	-0,412	0,026376	0,031	52	675
CFAP74	rs771618335	ms	Q9C0B2	58	T/S	0,593	0,036773	0,003	18	4,9
CFAP92	rs187885262	ms	Q9ULG3	294	D/G	0,359	0,045027	0,646	46	221
CFAP95	rs142028833	ms	Q5VTT2	159	D/G	-0,581	0,046827	0,123	23	82
CFHR1	rs145057542	stop	Q03591	331	*/W	-0,513	0,016653	1,000	23	41
CFHR5	rs1558287665	ms	Q9BXR6	308	M/V	0,531	0,024341	-	25	0,3
CGN	rs376782764	ms	Q9P2M7	211	R/W	-1,060	0,005845	0,999	14	3,4
CHAC2	rs531154748	ms	Q8WUX2	127	I/T	0,558	0,045585	0,936	19	0,7
CHAT	rs3810948	ms	P28329	47	D/E	-0,964	0,040130	0,000	17	3232
CHCHD6	rs1559811150	ms	Q9BRQ6	118	A/T	-0,820	0,035315	0,006	12	0
CHCT1	rs756919587	ms	Q86WR6	233	E/D	-0,548	0,006172	0,330	45	26
CHD3	rs1567833092	ms	Q12873	70	K/T	0,498	0,024487	0,413	28	0,4
CHD4	rs769476360	ms	Q14839	1762	N/S	-0,706	0,032468	0,983	15	1,9

CHD6	rs1345334517	ms	Q8TD26	2487	M/T	**-1,596**	0,023908	0,025	**12**	0,4
CHD8	rs754064184	ms	Q9HCK8	1469	R/H	0,519	0,045465	**0,892**	**25**	0,4
CHRDL2	rs141077727	ms	Q6WN34	15	A/T	**1,269**	0,001294	0,087	**16**	168
CHRM3	rs757383989	ms	P20309	275	A/V	-0,724	0,016255	0,101	**14**	0
CHRND	rs777430398	ms	Q07001	481	M/T	0,878	0,010930	-	**14**	3,8
CHRNE	rs121909516	ms	Q04844	163	S/L	-0,449	0,004708	**0,951**	**70**	**46**
CHRNG	rs375987895	ms	P07510	332	R/Q	-0,392	0,035836	-	**52**	2,5
CHST5	rs145210445	ms	Q9GZS9	22	M/I	0,386	0,010940	0,390	**56**	**43**
CHTF18	rs200765427	ms	Q8WVB6	571	D/N	-0,526	0,004792	0,049	**51**	320
CIC	rs1568487984	ms	Q96RK0	821	P/L	0,653	0,034965	**0,856**	**12**	0
CIC	rs1568486138	ms	-	372	A/V	-0,578	0,045390	0,015	**16**	0
CIDEA	rs149990783	ms	O60543	98	H/R	-0,719	0,001551	**0,631**	**30**	**15**
CIITA	rs763945523	ms	P33076	383	A/D	0,490	0,025484	**0,811**	**22**	9,8
CIT	rs747447396	ms	O14578	947	R/C	0,538	0,044801	0,441	**25**	1,1
CIT	rs1204187421	ms	-	1910	I/M	**-1,115**	0,001211	**0,960**	**11**	0,2
CKM	rs200170574	ms	P06732	83	E/K	-0,569	0,034546	0,353	**16**	0,4
CKMT2	rs199560112	ms	P17540	116	Y/C	-0,764	0,006132	**1,000**	**24**	1,9
CLASP2	rs114138303	ms	O75122	476	A/T	-0,809	5,57E-05	**0,697**	**42**	**58**
CLCA4	rs199643661	ms	Q14CN2	464	A/V	-0,598	0,012462	0,127	**27**	**92**
CLDN9	rs755885384	ms	O95484	215	D/V	-0,480	0,016891	0,274	**38**	1,5
CLEC16A	rs202155030	ms	Q2KHT3	501	V/M	0,470	0,031765	**0,996**	**23**	**12**
CLEC18C	16:70184579:A/G	ms	Q8NCF0	357	N/S	0,382	0,016033	**0,622**	**71**	N/A
CLEC2L	rs1430032217	ms	P0C7M8	101	P/S	-0,875	0,039988	**0,829**	**14**	2,6
CLEC4M	rs140767813	ms	Q9H2X3	263	R/H	-0,976	0,004897	**0,771**	**15**	**17**
CLEC9A	rs201379734	ms	Q6UXN8	202	E/Q	-0,560	0,051893	0,003	**17**	**15**
CLMN	rs148836979	ms	Q96JQ2	158	P/A	**-1,077**	0,001091	**0,635**	**26**	**96**
CLN6	rs769900670	ms	Q9NWW5-1	310	R/Q	-0,370	0,000388	0,000	131	1,5
CLTC	rs769713826	ms	Q00610	1138	M/T	-0,431	0,011711	0,280	**57**	0
CLUAP1	rs35065818	ms	Q96AJ1	196	V/F	0,707	0,031917	**0,726**	**16**	838
CMKLR2	rs779420589	ms	P46091	137	I/N	-0,689	0,006507	**0,607**	**14**	0,6
CMTR1	rs771375770	ms	Q8N1G2	605	G/S	-0,437	0,024352	0,000	**51**	4,2
CMYA5	rs769699893	ms	Q8N3K9	1459	V/I	-0,567	0,005201	0,007	**44**	0,8
CNBD1	rs750531941	ms	Q8NA66	421	K/R	0,479	0,020795	**0,565**	**32**	0,3
CNGA3	rs141086649	ms	Q16281	37	S/L	0,506	0,026202	0,000	**40**	**16**
CNGB1	rs369817749	ms	Q14028	311	P/L	0,459	0,029420	0,005	**51**	5,3
CNIH3	rs529360966	ms	Q8TBE1	85	A/V	-0,749	0,040748	**0,682**	**23**	0,4
CNNM4	rs561036245	ms	Q6P4Q7	122	N/I	**-1,037**	0,003385	0,261	**19**	9,8
CNTF	rs776744796	ms	P26441	172	S/A	0,494	0,007477	**0,989**	**61**	0
CNTN3	rs1559690789	ms	Q9P232	655	G/R	**1,346**	0,000260	**0,999**	**10**	0
CNTN4	rs375898825	ms	Q8IWV2	86	I/F	-0,377	0,047936	**0,971**	**44**	1,9
CNTN6	rs149799168	ms	Q9UQ52	310	G/S	0,423	0,008229	**0,999**	**67**	103
CNTNAP3B	rs1200081323	ms	Q96NU0	817	V/M	**1,319**	0,012235	0,085	**10**	1,3
CNTNAP3B	rs1191153640	ms	-	838	D/Y	-0,801	0,006177	**0,994**	**11**	0,7
CNTNAP5	rs757893588	ms	Q8WYK1	1054	L/S	0,724	0,016171	**0,999**	**16**	0
COG1	rs751977123	ms	Q8WTW3	892	T/M	0,603	0,025804	0,051	**19**	1,5
COG4	rs772833758	ms	Q9H9E3	461	I/L	0,734	0,034230	0,292	**18**	0,8
COIL	rs138873632	ms	P38432	419	R/Q	0,479	0,011847	**0,987**	**45**	**22**
COL16A1	rs760281570	ms	Q07092	20	H/R	0,394	0,045730	0,000	**30**	0,2
COL18A1	rs772587390	ms	P39060	146	Q/H	0,886	0,004456	0,000	**21**	0,1
COL1A2	rs1282395129	ms	P08123	437	D/V	-0,632	0,012548	-	**23**	0,4
COL20A1	rs199924678	ms	Q9P218	782	G/E	0,556	0,036858	**0,669**	**25**	4,9
COL22A1	rs764460053	ms	Q8NFW1	604	R/Q	-0,764	0,038113	0,000	**13**	0,4
COL24A1	rs773642410	stop	Q17RW2	1175	Q/*	0,877	0,005274	**1,000**	**14**	6,8
COL27A1	rs752587458	ms	Q8IZC6	303	T/S	0,535	0,007147	0,000	**46**	0,4
COL4A4	rs1559515185	ms	P08123	843	G/A	-0,689	0,003875	0,000	**27**	0,3
COL5A1	rs147589613	ms	P20908	114	A/D	0,603	0,009283	0,000	**21**	**88**
COL6A5	rs182112053	ms	P53420	1936	R/W	-0,396	0,025719	0,003	**48**	136
COL9A2	rs781428852	ms	Q14050	634	N/K	0,375	0,051653	**0,826**	**61**	3,3
COL9A3	rs780489761	ms	Q14055	584	G/S	-0,886	0,043537	**0,998**	**10**	0,4
COLEC12	rs1001323032	ms	Q5KU26	496	R/H	-0,766	0,027514	0,000	**12**	1,0
COPA	rs1557861801	ms	P53621	751	N/H	0,761	0,006373	0,058	**25**	0,4
COPG2	rs900527557	ms	Q9UBF2	513	L/I	-0,702	0,009171	**0,719**	**21**	**17**
COPG2	rs1174813614	ms	Q9UBF2	466	T/M	**1,005**	0,010779	**0,518**	**23**	3,0
COPS4	rs769306260	ms	Q9BT78	347	R/C	0,487	0,013098	**0,999**	**37**	0,9
CPAMD8	rs199816113	ms	Q8IZJ3	51	V/M	-0,848	0,031603	**0,948**	**11**	**11**
CPD	rs766105447	ms	O75976	318	K/R	-0,571	0,041620	0,017	**25**	0,4
CPEB1	rs549292365	ms	Q8BZB8	23	I/S	0,633	0,005429	0,000	**31**	9,1
CPEB1	rs200188266	ms	-	114	R/C	0,470	0,023830	**0,879**	**36**	**16**
CPSF2	rs140484405	ms	Q9P2I0	526	T/A	0,382	0,041955	0,074	**49**	**96**
CPT1A	rs1566348900	fs	P50416	569	F/X	**-1,527**	0,004625	**1,000**	**15**	0
CPT1B	rs773375016	ms	Q92523	517	R/W	-0,887	0,015027	**0,999**	**14**	0,4
CR1	rs1553292784	ms	P17927	1415	I/T	0,387	0,041084	-	**56**	686
CR1	rs1306545385	ms	-	965	I/T	0,392	0,035544	-	**56**	**1180**
CR1	rs1553290579	ms	-	515	I/T	0,392	0,035562	-	**57**	0
CRACDL	rs753045151	ms	Q6NV74	692	S/L	-0,372	0,044683	**0,996**	**52**	1,0
CRAT	rs762626138	ms	P43155	605	H/R	0,358	0,041665	0,000	**52**	9,4
CRAT	rs759917365	ms	-	244	N/K	-0,824	0,004506	0,045	**35**	5,7
CRB2	rs545270434	ms	Q5IJ48	327	R/W	-0,375	0,018390	**0,761**	**49**	0,8
CRB2	rs753967204	ms	-	747	V/M	-0,679	0,009851	**0,988**	**25**	0,9
CRHR1	rs372181383	ms	P34998	95	A/T	0,365	0,000237	0,001	168	6,4

CRHR1	rs2062265678	ms	-	179	E/K	-0,803	0,049250	0,030	11	0,4
CRHR2	rs151248954	ms	Q13324	367	R/C	0,514	0,011909	0,901	47	6,4
CRISP1	rs150372078	ms	P54107	71	A/T	0,964	0,014500	0,924	13	87
CRISP2	rs781375574	ms	P16562	176	M/V	-0,824	0,012522	0,000	20	0,4
CRNN	rs780465907	ms	Q9UBG3	363	S/R	-1,140	0,003788	0,649	17	0,3
CRY1	rs749506981	ms	Q16526	348	R/C	-0,838	0,010967	0,984	15	0,8
CRYBG1	rs1676015	ms	Q9Y4K1	1853	T/S	0,404	0,021599	0,353	42	6642
CSH1	rs1426728052	ms	P0DML2	124	A/D	0,438	8,57E-05	0,251	153	0,4
CSH2	rs1439972034	ms	P0DML3	124	A/D	0,438	8,56E-05	0,010	153	0,3
CSHL1	rs149910727	ms	Q14406	98	T/M	-0,419	0,043080	0,171	46	5,3
CSMD1	rs571852185	ms	Q96PZ7-1	167	I/M	-1,032	0,015289	0,845	10	0,8
CSMD1	rs571113460	ms	-	1485	P/L	0,519	0,038605	0,985	32	2,5
CSMD1	rs552956828	ms	-	2464	R/L	-0,455	0,034290	0,300	34	1,1
CSN1S1	rs201502829	ms	P47710	4	L/P	-1,057	0,007322	0,568	13	15
CSN3	rs757594677	ms	Q9UNS2	118	I/T	0,626	0,013363	0,131	19	0
CSPG4	rs142305247	ms	Q6UVK1	660	R/Q	0,593	0,049003	0,046	23	82
CSPG4	rs144427836	ms	-	657	V/M	0,593	0,049003	0,905	23	88
CSRP3	rs759269008	ms	P50461	145	F/S	0,731	0,035730	-	12	0,3
CST3	rs776109203	ms	P01034	8	P/R	-0,635	0,003369	0,000	37	0,6
CSTF2T	rs1564612476	ms	Q9H0L4	118	P/S	0,642	0,025865	-	22	0
CTAGE15	rs1444511885	ms	A4D2H0	148	C/Y	0,907	0,024606	0,013	11	0
CTC1	rs780355193	ms	Q2NKJ3	744	R/H	0,586	0,003944	0,711	38	0,7
CTC1	rs778742090	ms	-	86	S/L	-0,511	0,016442	0,514	41	2,6
CTDSP1	rs547460809	ms	Q9GZU7	48	R/W	0,670	0,012971	0,457	22	1,5
CTNND1	rs767264268	ms	O60716	34	R/G	-0,443	0,018404	0,978	43	7,6
CTNS	rs759156059	ms	O60931	77	V/L	0,600	0,007065	0,000	29	0,4
CTSV	9:97036711:C/A	ms	-	145	G/C	-0,765	0,029408	1,000	12	N/A
CUX1	rs761867945	ms	P39880	436	R/H	-0,529	0,040149	0,963	25	0,4
CUX1	7:102111745:C/G	ms	-	193	A/G	0,696	0,031200	0,178	13	N/A
CUX2	rs201097767	ms	Q14529	1242	G/R	0,514	0,020960	0,106	35	21
CYB561D1	rs368199735	ms	Q8N8Q1	152	R/W	0,583	0,030487	0,434	25	0,8
CYBC1	rs142011515	ms	Q9BQA9	129	R/W	-0,802	0,036527	0,999	11	11
CYFIP2	rs766783609	ms	Q96F07	323	A/T	-0,654	0,020488	0,005	20	0,1
CYP2C9	rs772291418	ms	P11712	202	N/D	0,470	0,018604	0,098	60	0
CYP2D6	rs77578877	ms	P10635	185	V/L	0,791	0,011583	0,096	30	18
CYP4A22	rs369829008	ms	Q5TCH4	17	G/R	0,537	0,040217	0,250	23	2,6
CYP8B1	rs35764459	ms	Q9UNU6	238	K/R	-0,448	0,023120	0,101	31	2505
DACT1	rs771524719	ms	Q9NYF0	564	V/I	-0,694	0,016292	0,170	19	1,1
DAGLA	rs778280333	ms	Q9Y4D2	768	A/V	-0,719	0,025970	0,000	12	6,0
DBNL	rs139647339	ms	Q9UJU6	91	D/H	-0,427	0,040463	-	41	90
DBT	rs185492864	ms	P11182	301	R/C	-0,474	0,004526	0,861	49	23
DCAF7	rs780790401	ms	P61962	154	Q/H	0,498	0,030969	0,003	38	0
DCAF7	rs745412598	ms	-	41	E/G	0,511	0,021338	0,979	30	0,2
DCLK1	rs145262053	ms	O15075	711	A/V	-0,379	0,031671	-	46	382
DCTN1	rs774489951	ms	Q14203	992	E/G	1,005	0,009554	-	11	0
DDX42	rs117181531	ms	Q86XP3	754	S/I	0,429	4,48E-09	0,114	317	180
DDX5	rs559742081	ms	P17844	461	R/H	0,496	0,001674	0,887	61	3,0
DDX5	rs758505488	ms	-	75	T/P	0,356	0,047379	0,374	35	0,1
DDX50	rs148713144	ms	Q9BQ39	132	R/C	0,366	0,017729	0,000	80	116
DDX54	rs761117277	ms	Q8TDD1	765	R/P	0,545	0,032962	0,540	26	0,2
DDX56	rs1562584773	ms	Q9NY93	285	T/I	-0,663	0,050008	0,148	12	0
DDX60	4:168221750:T/C	ms	Q8IV21	1652	I/M	0,695	0,034865	0,003	13	N/A
DEFB1	rs140503947	ms	P60022	35	Y/C	0,847	0,036104	0,981	12	3,3
DEFB106A	8:7825178:T/C	start_lost	Q8N104	1	M/T	0,627	0,020502	0,044	18	N/A
DEFB110	rs770964208	ms	Q30KQ9	55	Y/C	-0,387	0,014267	0,622	62	0
DEFB4A	8:7896588:C/A	ms	O15263	58	T/K	0,867	0,016606	0,000	13	N/A
DEFB4B	rs756098185	ms	O15263	58	T/K	0,672	0,008552	0,000	22	5,0
DENND1A	rs745972611	ms	Q8IVD6	1064	K/R	-0,617	0,051954	0,003	13	0,7
DENND1C	rs760319460	ms	Q8IV53	751	P/S	-0,964	0,008908	0,511	11	1,3
DENND2D	rs201272881	ms	Q9H6A0	420	R/Q	-0,530	0,010901	0,352	24	19
DENND2D	rs145695008	stop	-	16	R/*	-0,626	0,010945	1,000	26	58
DENND4B	rs752750941	ms	O75064	1097	L/I	0,511	0,011794	0,903	32	0,4
DGKB	rs1355918054	ms	Q9Y6T7	34	E/G	-0,910	0,000213	0,074	34	0,8
DGKI	rs770128558	ms	O75912	132	S/L	0,558	0,024197	0,003	28	1,1
DHRS7	rs377160812	ms	Q9Y394	227	I/V	-0,385	0,032310	0,994	41	2,2
DHX34	rs116000939	ms	Q14147	159	R/Q	0,460	0,007256	0,023	57	321
DHX36	rs749710536	ms	Q9H2U1	137	E/K	-0,963	0,038784	0,010	11	0,4
DHX40	rs141945224	ms	Q8IX18	713	R/C	-0,365	0,002525	0,349	148	15
DHX57	rs745664385	ms	Q6P158	408	V/I	-0,361	0,032686	0,998	62	7,0
DHX9	rs752226276	ms	Q08211	1223	R/Q	-0,504	0,046347	-	28	0,6
DIAPH3	rs200189161	ms	Q9NSV4	1042	R/H	-0,547	0,007344	0,975	46	36
DIP2C	rs746715099	ms	Q9Y2E4	77	R/Q	0,556	0,007849	0,018	32	4,2
DISC1	rs79978593	ms	Q9NRI5	13	A/G	0,863	0,028379	0,000	17	269
DISC1	rs138886515	ms	-	470	E/K	-0,488	0,049751	0,925	25	2,6
DLX2	rs1422322241	ms	Q07687	22	T/R	0,744	0,023351	0,031	12	0,3
DLX5	rs148894146	ms	P56178	238	H/N	-0,518	0,013816	0,006	26	14
DMAC1	rs765696614	ms	Q96GE9	108	A/T	-0,996	0,011941	0,911	15	0,4
DMGDH	rs138871430	ms	Q9UI17	643	K/E	0,390	0,007524	0,029	65	73
DMRTC2	rs141103699	stop	Q8IXT2	346	R/*	0,409	0,023076	1,000	55	19
DMWD	rs766488376	ms	Q09019	10	S/L	0,583	0,035267	0,000	31	20

**DMXL2**	rs762703183	ms	Q8TDJ6	2895	S/C	-0,591	0,003875	0,322	**44**	5,0
**DMXL2**	rs143811213	ms	-	372	N/H	-0,652	0,030206	0,367	**16**	**16**
DNAH10	12:123790015:T/C	ms	Q8IVF4	570	V/A	**-1,378**	0,008236	**0,571**	**10**	N/A
DNAH11	rs749125298	ms	Q96DT5	358	R/C	0,444	0,051080	0,000	**23**	3,0
DNAH14	rs761301731	ms	Q0VDD8	3062	K/E	0,862	0,006152	0,290	**18**	0
DNAH17	rs775238626	ms	Q9UFH2	4432	R/H	0,593	0,020575	**0,980**	**28**	6,4
**DNAH3**	rs772685691	ms	Q8TD57	534	K/R	-0,674	0,019741	0,003	**12**	0,2
**DNAH3**	rs766110188	ms	-	269	Y/D	0,936	0,027800	**0,937**	**12**	0,5
DNAH5	rs140700961	ms	Q8TE73	192	A/T	0,515	0,007894	0,068	**42**	**25**
DNAH6	rs200844717	ms	Q9C0G6	2666	S/Y	0,818	0,014248	**0,997**	**19**	129
DNAH7	rs189913342	ms	Q8WXX0	2595	L/Q	-0,903	0,013169	**1,000**	**11**	**10**
DNAH8	rs862432	ms	Q96JB1	2661	T/N	**1,587**	0,002190	0,012	**12**	**7598**
DNAJA4	rs777655450	stop	Q8WW22	284	R/*	-0,460	0,030013	**1,000**	**39**	0,9
DNAJC13	rs762566567	ms	O75165	1396	P/T	-0,563	0,007391	**0,998**	**23**	0,8
DNASE1	rs8176927	ms	P24855	2	R/S	0,618	0,026663	0,003	**21**	**3914**
DNER	rs772555280	ms	Q8NFT8	256	R/P	0,626	0,050652	**0,554**	**21**	0,4
DOC2B	rs548884378	ms	Q14184	4	R/Q	**-1,129**	0,019165	0,000	**14**	**60**
DOCK1	rs140799120	ms	Q14185	255	I/F	0,457	0,035335	0,342	**33**	0,4
DOCK6	rs763348704	ms	Q96NP0	615	F/L	-0,896	0,042099	**0,994**	**16**	9,8
**DOCK8**	rs530454954	ms	Q8NF50	1766	R/Q	-0,765	0,051173	0,017	**24**	4,5
**DOCK8**	rs373337056	ms	-	1484	T/I	-0,735	0,048313	0,422	**11**	0,8
**DOCK8**	rs202110964	ms	-	249	D/N	-0,773	0,037826	**0,529**	**11**	**10**
DOP1B	21:36263581:C/T	ms	Q9Y3R5	1784	S/L	**1,174**	0,024190	**0,461**	**11**	N/A
DPH6	rs143353998	ms	Q7L8W6	46	D/N	-0,592	0,047582	**0,953**	**23**	**76**
DPP10	rs752853823	ms	Q8N608	701	T/I	0,853	0,004632	0,106	**21**	0,2
DPP9	rs1309548680	ms	Q86TI2	692	R/W	0,521	0,018901	**0,981**	**28**	0,7
DPYSL4	rs757669088	ms	O14531	381	V/I	0,410	0,017294	0,266	**54**	0,8
**DROSHA**	rs748009983	ms	Q9NRR4	1102	P/A	0,412	0,043789	**0,783**	**31**	0,2
**DROSHA**	rs750540831	ms	-	250	R/Q	-0,663	0,033701	0,234	**25**	7,8
DSCAML1	rs549967851	ms	Q8TD84	1980	P/Q	0,544	0,030873	-	**24**	**47**
DSP	rs144465638	ms	P15924	1349	S/N	0,761	0,010453	0,005	**20**	2,0
**DSPP**	rs61738509	ms	Q9NZW4	231	P/T	-0,743	0,044043	**0,949**	**12**	**1091**
**DSPP**	rs61738508	ms	-	268	G/W	-0,742	0,044153	**0,999**	**12**	**1091**
DST	rs768885705	ms	Q96J76	507	E/V	0,582	0,015948	**0,890**	**50**	0
DTNA	rs150147476	ms	Q9Y4J8	124	H/R	-0,409	0,019098	0,001	**80**	9,8
DUOX2	rs202111673	ms	Q9NRD8	1425	Q/E	0,434	0,033503	0,006	**37**	6,0
DUSP4	rs1817800187	ms	Q13115	61	I/M	0,961	0,039400	**0,859**	**10**	0,1
DUSP8	rs755470031	ms	Q13202	75	R/C	**-1,148**	0,000238	**0,846**	**10**	0,4
DYNC1H1	rs771773704	ms	Q14204	3925	Q/E	0,687	0,006623	0,000	**41**	0,4
DYNC2H1	rs745928254	ms	Q8NCM8	1850	N/S	-0,553	0,016142	0,057	**53**	1,1
DYNC2I1	rs73167274	ms	Q8WVS4	923	P/Q	0,462	0,029006	**0,987**	**44**	238
DYNC2I2	rs778308337	ms	Q96EX3	467	K/Q	0,917	0,026003	0,046	**15**	1,3
DYRK1A	21:37505403:G/A	ms	Q13627	445	D/N	**1,248**	0,038008	0,140	**11**	N/A
DYSF	rs1559177530	ms	O75923	936	F/Y	0,823	0,036955	**0,997**	**13**	0
E2F7	rs765296391	ms	Q96AV8	643	G/S	0,352	0,036073	0,005	**50**	0,4
EBF4	rs1479421192	stop	Q9BQW3	568	Q/*	-0,549	0,038848	**1,000**	**25**	1,1
ECEL1	2:232486040:A/G	ms	O95672	205	I/T	-0,630	0,032974	**0,907**	**11**	N/A
EDNRA	rs192190120	ms	P25101	86	I/V	-0,367	0,034706	0,003	**44**	**46**
EEFSEC	rs768235736	ms	P57772	566	A/T	0,588	0,014005	0,000	**24**	0,7
EFCAB11	rs147386709	ms	Q9BUY7	19	E/A	-0,797	0,044479	0,005	**14**	**35**
EFCAB12	rs759073143	ms	Q6NXP0	375	T/I	0,823	0,042306	**0,998**	**10**	0,8
EFCAB13	rs746496282	ms	Q8IY85	23	N/D	0,438	0,021181	0,000	**49**	0,1
EFCAB3	rs1567727823	ms	Q8N7B9	191	K/T	0,729	0,045934	0,139	**16**	0
EFCAB6	rs750687549	ms	Q5THR3	1458	D/N	0,553	0,033623	**0,993**	**14**	0,4
EFTUD2	rs760166112	ms	Q15029	245	H/R	0,744	0,023227	**0,839**	**24**	0,4
EGFL8	rs138835391	ms	Q99944	219	R/L	-0,405	0,010530	0,174	**55**	**63**
EGFR	rs771995749	ms	P00533	730	L/R	-0,472	0,037279	**0,794**	**33**	0,8
EHMT1	rs144871446	ms	Q9H9B1	246	R/Q	0,551	0,050310	0,090	**29**	108
EIF2AK3	rs752973018	ms	Q9NZJ5	779	N/S	0,608	0,009257	0,000	**26**	0,1
EIF5B	rs192772936	ms	O60841	616	R/Q	-0,461	0,012354	0,416	**49**	**49**
ELAPOR2	rs34772926	ms	A8MWY0	605	R/H	**-1,099**	0,034969	0,146	**12**	**46**
ELK3	rs138659793	ms	P41970	315	A/V	-0,385	0,041795	**0,685**	**35**	5,3
**ELL**	rs201725032	ms	P55199	424	R/C	0,372	0,027766	0,003	**63**	**91**
**ELL**	rs139501652	ms	-	243	D/N	-0,659	0,007326	0,404	**28**	165
ELMO1	rs765065086	ms	Q92556	331	A/T	**-1,249**	0,037612	**1,000**	**11**	0
ELN	rs139718810	ms	P15502	162	G/S	0,493	0,039395	0,000	**27**	3,0
ELP2	rs148576942	ms	Q6IA86	618	T/R	0,367	0,002537	**0,868**	**98**	7,6
EMID1	rs201794555	ms	Q96A84	129	R/Q	**-1,117**	0,026225	0,263	**16**	1,1
EMILIN2	rs781538463	ms	Q9BXX0	40	P/R	0,560	0,031525	**0,778**	**14**	147
**ENGASE**	rs199859024	ms	Q8NFI3	421	V/I	-0,609	0,026188	0,038	**28**	9,4
**ENGASE**	rs753581791	ms	-	334	R/G	-0,435	0,028193	**0,999**	**66**	1,7
**ENPP7**	rs1568484654	ms	Q6UWV6	18	P/L	-0,964	0,031065	0,000	**14**	0
**ENPP7**	rs144007702	ms	-	238	R/S	-0,651	0,012946	0,007	**39**	0
EP300	rs142823793	ms	Q09472	543	M/V	-0,824	0,018030	0,295	**15**	1,9
EPC2	rs770290421	ms	Q52LR7	98	K/Q	0,477	0,007762	**0,995**	**37**	0
EPDR1	rs149095633	ms	Q9UM22	121	P/L	0,363	0,039689	0,280	**51**	**80**
EPG5	rs778147277	ms	Q9HCE0	502	V/E	0,623	0,029146	**0,975**	**11**	0
EPHA1	rs780968060	ms	P21709	233	P/T	-0,641	0,051975	0,090	**18**	0
EPHA3	rs1559602030	ms	P29320	187	G/D	-0,594	0,006089	**0,999**	**38**	0
EPHA7	rs756964921	ms	Q15375	552	Q/R	0,836	0,016201	**0,772**	**14**	1,5

EPHB3	rs779216599	ms	P54753	436	P/L	1,433	0,005975	0,548	13	0,2
EPHB3	rs749686296	ms	-	970	V/I	0,690	0,040025	0,674	19	0,4
EPHB4	rs1562967203	ms	P54760	791	A/S	-0,783	0,038120	0,999	11	0
EPPK1	rs371322120	ms	P58107	1687	T/M	0,371	0,026482	0,939	73	11
EPS8L2	rs780112279	ms	Q9H6S3	62	M/L	-0,373	0,006073	0,262	88	13
EPS8L2	rs1564981510	ms	-	721	A/V	-0,704	0,006751	0,000	19	0,8
EPX	rs758186180	ms	P11678	112	F/L	-0,392	0,002923	0,089	123	6,4
ERAP2	rs34261036	ms	Q6P179	411	L/R	2,240	0,030061	0,980	13	352
ERBB2	rs767813285	ms	P04626	964	E/Q	0,514	0,004527	0,708	47	0,8
ERBB3	rs769634421	ms	P21860	94	F/V	-0,781	0,009530	0,628	16	0,7
ERCC1	rs771438243	ms	P07992	152	Y/C	0,572	3,74E-05	-	97	1,1
ERCC5	rs1882915924	ms	P28715	889	L/F	-0,874	0,011984	0,540	14	4,8
ERCC8	rs770585176	ms	Q13216	379	S/T	0,600	0,046391	0,000	14	0,1
ERFL	rs776261803	ms	A0A1W2PQ73	117	F/L	-0,389	0,044303	0,096	48	0
ERN1	rs374518749	ms	O75460	374	K/Q	-0,368	0,008824	0,199	64	7,6
ERV3-1	rs1259230422	ms	Q14264	514	R/C	-0,659	0,036640	0,537	13	6,7
ERVV-2	rs191385202	ms	B6SEH9	526	F/L	-0,601	0,045985	0,494	25	44
ESYT3	rs200676435	ms	A0FGR9	236	G/E	-0,416	0,003008	0,976	70	3,8
ETNPPL	rs143646148	ms	Q8TBG4	296	A/G	0,533	0,047569	0,855	11	149
ETV2	rs780706581	ms	O00321	112	A/T	-1,176	0,005721	0,193	21	15
ETV5	rs1467805143	ms	P41161	245	R/W	-1,061	0,007116	0,970	17	0,3
ETV5	rs1281944233	ms	-	482	P/L	1,242	0,017184	0,998	13	0,4
EVPL	rs199543579	ms	Q92817	1033	A/T	0,410	0,001503	0,013	98	27
EVPL	rs769457633	ms	-	1119	R/H	-0,411	0,004464	0,924	95	1,3
EVX2	rs151290628	ms	Q03828	114	M/R	0,626	0,046649	0,502	16	6,0
EXD1	rs777472629	ms	Q8NHP7	535	V/E	0,417	0,049266	0,000	22	0,8
EXOC3L2	rs1216771253	ms	Q2M3D2	645	R/G	0,652	0,037995	0,674	11	0,5
EXOC6	rs141813516	ms	Q8TAG9	89	K/N	-0,507	0,019405	0,138	25	19
EXOC6B	rs762944459	ms	Q9Y2D4	639	S/N	0,467	0,044997	0,983	27	0,2
EXPH5	rs777861207	ms	Q8NEV8	431	A/S	-0,720	0,017973	0,040	16	0,8
EXPH5	rs762635762	ms	-	1969	D/N	0,822	0,000308	1,000	52	0,7
EXTL1	rs760822796	ms	Q92935	208	R/Q	-0,994	0,014590	0,146	23	0,4
EXTL3	rs1563222520	ms	O43909	786	L/V	0,564	0,008993	0,438	34	0,4
F13B	rs764924610	ms	P05160	577	L/W	-0,511	0,017159	0,871	22	0,9
FAAP20	rs369043951	ms	Q6NZ36	164	D/E	-0,356	0,031282	0,385	63	3,0
FAM118A	rs777058813	ms	Q9NWS6	85	R/W	-1,027	0,007808	0,397	14	11
FAM120B	rs775947746	ms	Q96EK7	623	R/H	0,766	0,010501	0,994	31	2,6
FAM151A	rs374572716	ms	Q8WW52	37	R/Q	0,538	0,008563	0,022	38	23
FAM151A	rs768990252	ms	-	71	T/S	-0,390	0,008306	0,421	75	0,4
FAM163B	rs555269190	ms	P0C2L3	136	G/R	0,850	0,007457	0,213	14	4,2
FAM171A2	rs570503128	ms	A8MVW0	128	R/W	0,601	0,039658	0,991	20	9,4
FAM200A	rs147653498	ms	Q8TCP9	126	L/V	0,525	0,037952	0,024	21	15
FAM20A	rs754357363	ms	Q96MK3	48	G/S	-0,643	0,000141	0,019	69	3,4
FAM228B	rs777345681	ms	P0C875	30	C/R	-0,394	0,027970	0,001	48	0,7
FAM237A	rs200894319	ms	A0A1B0GTK4	65	V/I	-0,416	0,013907	0,000	47	21
FAM72A	rs782341403	ms	Q5TYM5	27	A/P	-0,933	0,002435	0,161	20	2129
FAM83F	rs149665361	ms	Q8NEG4	334	R/C	-0,580	0,036183	0,924	13	87
FAM83H	rs1336225373	stop	Q6ZRV2	1143	E/*	0,630	0,039507	1,000	11	0,8
FAM86B2	rs1470859049	ms	P0C5J1	10	E/K	0,415	0,014609	0,003	52	4,9
FAM91A1	rs776239012	ms	Q658Y4	281	L/M	-0,601	0,009923	0,963	23	3,4
FANCA	rs200220791	ms	O15360	292	H/D	-0,373	0,014696	0,000	75	16
FANCF	rs1055714341	ms	Q9NPI8	66	W/R	1,130	0,007984	0,694	10	0,7
FANCG	rs754927660	ms	O15287	480	L/F	-0,508	4,91E-05	0,995	113	0,2
FANCM	rs550238354	ms	Q8IYD8	573	R/Q	-0,585	0,043229	1,000	27	0,8
FARSA	rs747427583	ms	Q9Y285	23	S/G	-0,533	0,011976	0,896	34	0,8
FASN	rs760665615	ms	P49327	342	A/S	-0,767	0,019194	0,194	12	15
FASN	rs2228307	ms	-	1888	I/V	-0,767	0,019199	0,041	12	3017
FASTKD2	rs771505646	ms	Q9NYY8	70	S/L	-0,535	0,047246	0,037	21	0
FAT1	rs774367320	ms	Q14517	4055	P/L	-0,384	0,010429	0,999	72	0,4
FAT2	rs149549832	ms	Q9NYQ8	2694	P/S	-0,864	0,039211	0,998	12	0,7
FAT3	rs201524480	ms	Q8TDW7	1152	I/T	1,142	0,003821	0,479	12	29
FAT3	rs188857169	ms	-	800	N/Y	-0,624	0,038320	0,912	23	16
FBLN2	rs200898109	ms	P98095	642	R/C	0,443	0,028885	0,897	33	4,5
FBN3	rs148024558	ms	Q75N90	371	M/T	0,475	0,046090	0,005	22	286
FBN3	rs149806821	ms	-	445	G/S	0,385	0,016567	0,998	56	26
FBN3	rs1568355723	ms	-	2535	P/S	-0,841	0,004318	0,996	26	0,4
FBXL5	rs374329849	ms	Q9UKA1	212	V/I	-0,401	0,022558	0,993	49	7,6
FBXO27	rs774999328	ms	Q8NI29	267	Y/C	0,673	0,027529	0,976	21	0,4
FBXW10	rs774691557	ms	Q5XX13	124	W/R	-0,496	0,004442	0,994	50	0,4
FBXW10B	rs1201781215	ms	O95170	15	R/H	-1,111	0,002331	0,003	12	0,8
FCGBP	rs150766794	ms	Q9Y6R7	872	Y/C	0,699	0,033889	1,000	17	99
FCGBP	rs376286887	ms	-	1135	S/N	0,788	0,045419	0,015	11	5,3
FCRL4	rs769507733	ms	Q96PJ5	512	D/N	-0,466	0,017274	0,386	38	0
FCSK	rs764653879	stop	Q8N0W3	969	E/*	-1,058	0,032116	1,000	10	0,4
FEM1B	rs1567114340	ms	Q9UK73	181	N/D	0,530	0,011089	0,612	32	0
FER1L5	rs368841036	ms	A0AVI2	43	V/M	0,612	0,012861	0,738	27	5,5
FER1L6	rs1160334898	ms	Q2WGJ9	459	E/K	0,703	0,036451	0,080	25	1,0
FETUB	rs143600252	ms	Q9UGM5	335	Q/E	0,413	0,021014	0,081	39	1,1
FGF6	rs139049599	ms	P10767	120	I/V	-0,787	0,005064	0,830	39	22
FGFBP2	rs142331717	ms	Q9BYJ0	101	R/H	-0,372	0,007183	0,000	87	86

FGFR2	rs536181987	ms	P21802	450	R/C	0,500	0,028125	**0,672**	**30**	5,7
FGGY	rs199980192	ms	Q96C11	17	V/I	**1,017**	0,020776	**1,000**	**11**	108
FHAD1	rs1419575009	ms	B1AJZ9	1300	K/Q	0,844	0,015240	0,224	**13**	0,8
FHIP1A	rs775802962	ms	Q05DH4	437	K/N	0,369	0,033960	**0,648**	**47**	0,2
FIBIN	rs138273386	ms	Q8TAL6	96	R/H	-0,356	0,039715	**0,957**	**56**	316
FILIP1L	rs764581624	ms	Q4L180	964	E/K	-0,957	0,009495	**0,468**	**16**	1,1
FKBP10	rs781768559	ms	Q96AY3	408	C/F	-0,669	0,002322	**1,000**	**34**	0,4
FLII	rs61741784	ms	Q13045	364	E/V	0,392	0,006232	0,165	**97**	429
FLNA	rs187029309	ms	P21333	1991	S/L	0,655	0,002158	-	**28**	216
FLNC	rs374847180	ms	Q14315	437	R/C	0,544	0,015231	**0,953**	**37**	4,9
FLVCR2	rs199824003	ms	Q9UPI3	303	R/Q	-0,710	0,017058	0,025	**11**	2,6
FLYWCH1	rs200267787	ms	Q4VC44	387	R/Q	0,508	0,007436	0,003	**50**	**39**
FLYWCH1	rs748059598	ms	-	528	R/Q	0,532	0,008791	**0,978**	**25**	0,4
FMN1	rs368779133	ms	Q68DA7	199	A/V	-0,391	0,033370	0,000	**48**	3,8
FMNL2	rs189416564	ms	Q96PY5	110	L/V	0,755	0,020482	**0,578**	**29**	152
FMO2	rs145876121	ms	P31512	438	E/K	-0,373	0,046652	0,336	**43**	**69**
FMO2	rs772086521	ms	-	439	L/F	**1,258**	0,004932	**0,971**	**15**	4,2
FNDC1	rs774630537	ms	Q4ZHG4	165	R/C	0,469	0,027246	**0,685**	**33**	3,0
FNDC7	1:108730742:A/G	ms	Q5VTL7	565	I/V	**1,349**	0,025181	0,000	**10**	N/A
FNDC7	rs3006870	ms	-	367	N/S	-0,862	0,028966	**0,483**	**15**	**9237**
FOXD1	rs917127030	ms	Q16676	356	A/G	0,406	0,000325	0,000	127	**37**
FOXD4L4	rs1256582592	ms	Q8WXT5	171	R/H	-0,841	0,023928	**1,000**	**14**	**76**
FOXD4L5	rs1404314114	ms	Q5VV16	343	R/S	0,658	0,049006	0,234	**12**	0,4
FOXI2	rs1377314581	ms	Q6ZQN5	53	Y/C	-0,395	0,023236	0,000	**55**	0
FOXI3	rs745918234	ms	A8MTJ6	369	S/G	-0,773	0,021071	0,003	**10**	0,4
FOXQ1	rs773628191	ms	Q9C009	375	P/S	-0,588	0,041341	**0,960**	**25**	**30**
FRAS1	rs748159035	ms	Q86XX4	3585	A/T	-0,363	0,003615	0,001	105	0,8
FRAS1	rs774824172	ms	-	644	G/R	**-1,211**	0,009411	**0,830**	**12**	0,7
FREM1	rs760645412	ms	Q5H8C1	1529	L/V	-0,608	0,045681	0,001	**14**	1,1
FREM1	rs201154402	ms	-	441	D/N	0,846	0,033524	**0,599**	**11**	**13**
FRMPD2	rs760390374	ms	Q68DX3	666	H/N	0,640	0,047273	0,001	**15**	0,4
FSIP2	rs113773415	ms	Q5CZC0	3988	S/F	**1,725**	0,000945	0,000	**12**	**63**
FSIP2	rs111265848	ms	-	568	Y/F	**1,725**	0,000945	0,000	**12**	132
FTCDNL1	rs1559218391	ms	E5RQL4	135	E/K	0,571	0,010084	0,000	**38**	0
FTSJ3	rs765433762	ms	Q8IY81	626	E/K	-0,585	0,048324	0,007	**21**	0,8
FTSJ3	rs747668148	ms	-	138	L/V	0,408	0,002506	**0,999**	**90**	1,7
FTSJ3	rs146889707	ms	-	356	S/P	0,413	0,009661	0,312	**88**	**62**
FTSJ3	rs760234926	ms	-	776	R/Q	-0,497	0,030306	**0,968**	**34**	0,8
FTSJ3	rs767462503	ms	-	494	R/Q	0,613	0,018297	0,000	**23**	1,5
FUCA1	rs768929138	ms	P04066	73	G/A	-0,719	0,029409	0,273	**22**	0,8
FXYD3	rs202118232	ms	Q14802	48	R/G	-0,694	0,042966	0,000	**11**	**27**
FYB1	rs768403381	ms	O15117	533	Q/P	0,511	0,009373	**0,997**	**29**	0
FYB2	rs755707826	ms	Q5VWT5	558	I/T	0,501	0,022191	0,110	**22**	0,2
GAB4	rs61740195	ms	Q2WGN9	414	E/D	0,646	0,025323	0,224	**18**	**83**
GABRD	rs139300921	ms	O14764	220	R/C	-0,378	0,000311	**0,741**	150	**70**
GABRG3	rs375624145	ms	Q99928	395	V/I	0,552	0,000925	0,009	**53**	4,2
GAD1	rs45566933	ms	Q99259	228	I/L	0,539	0,045535	0,062	**33**	**48**
GANC	rs370258614	ms	Q8TET4	583	V/M	-0,644	0,050599	**0,788**	**15**	7,0
GARIN1A	rs879682991	ms	Q6NXP2	106	A/T	-0,779	0,048088	0,273	**13**	3,4
GARIN4	rs780034561	ms	Q8IYT1	387	M/T	-0,403	0,018124	0,000	**57**	1,0
GBGT1	rs117595304	ms	Q8N5D6	66	Y/C	-0,363	1,22E-09	**0,817**	457	311
GBP3	rs139129340	ms	Q8WXF7	163	N/S	**1,332**	0,010678	0,074	**12**	**99**
GBP3	rs760485167	ms	-	432	C/R	-0,962	0,020912	0,000	**14**	0,8
GBP7	rs772256061	ms	Q8N8V2	283	E/Q	-0,416	0,046400	0,124	**47**	0,4
GCKR	rs755683893	stop	Q14397	610	Q/*	0,493	0,043509	**1,000**	**42**	2,6
GCKR	rs545938878	ms	-	612	R/L	**-1,565**	0,000795	**0,455**	**13**	0,4
GEMIN2	rs762142842	start_lost	O14893	1	M/L	0,399	0,021143	0,000	**70**	1,1
GEMIN5	rs749935126	ms	Q8TEQ6	333	H/R	-0,939	0,027477	**0,994**	**22**	0,7
GFRA4	rs148812242	ms	Q9GZZ7	8	A/V	-0,432	0,042235	0,009	**37**	7,2
GGA3	rs751312773	ms	Q9NZ52	71	A/T	0,564	0,046101	**0,999**	**20**	0,6
GGCT	rs539867008	ms	O75223	109	G/E	0,409	0,026381	0,000	**65**	**55**
GGN	rs1375969871	ms	Q86UU5	464	P/T	0,868	0,041273	0,000	**11**	0
GGTLC2	rs146282307	ms	Q14390	107	S/L	-0,556	0,049955	**0,710**	**21**	**17**
GH1	rs61762497	ms	P01241	82	E/D	-0,393	0,036652	0,005	**58**	9,8
GH2	rs199558095	ms	P01242	103	R/C	-0,429	0,000203	**1,000**	129	**11**
GH2	rs16947229	ms	-	208	G/E	0,449	8E-05	0,000	141	4,2
GHR	rs6182	ms	P10912	440	C/F	-0,356	0,000832	0,078	113	**1604**
GHR	rs6184	ms	-	579	P/T	-0,356	0,000832	0,071	113	**1602**
GIMD1	rs769087374	ms	P0DJR0	60	R/H	0,383	0,015758	**0,711**	**67**	3,4
GIN1	rs759917916	ms	Q9NXP7	491	T/M	0,655	0,004077	0,019	**31**	3,4
GINS1	rs137901350	ms	Q14691	83	R/C	-0,376	0,024916	**1,000**	**52**	**33**
GJB5	rs116644255	ms	O95377	22	R/C	0,509	0,043272	-	**20**	**88**
GLG1	rs752528979	ms	Q92896	393	S/L	0,536	0,016260	0,173	**24**	0,8
GLI1	rs1565600997	ms	P08151	516	G/C	0,771	0,010149	-	**11**	0
GLO1	rs777974212	ms	Q04760	105	W/R	-0,583	0,000106	**1,000**	**66**	0
GLYATL1B	rs112516845	ms	A0A0U1RQE8	244	G/A	-0,428	0,040621	0,000	**28**	2,6
GMPPA	rs772485317	ms	Q96IJ6	200	R/H	0,595	0,027245	0,003	**14**	2,6
GMPR2	rs1566680407	stop	Q9P2T1	91	Q/*	-0,889	0,007035	**1,000**	**15**	0
GNA13	rs1062597	ms	Q14344	221	V/L	-0,520	1,07E-06	0,044	153	220
GNAT2	rs1189352767	ms	P19087	254	C/R	**-1,066**	0,037318	**1,000**	**12**	0,2

GNE	rs751107200	ms	Q9Y223	14	Q/E	0,565	0,005558	0,000	46	0,1
GNL1	rs142398523	ms	P36915	326	E/K	0,453	0,030063	0,235	57	534
GOLGA2	rs16912752	ms	Q08379	456	M/V	1,206	0,021948	0,006	10	10362
GOLGA8H	rs767587258	ms	P0CJ92	141	Q/E	-2,113	0,002525	0,003	16	0,8
GOLGA8M	rs576118690	ms	H3BSY2	136	Q/K	-0,507	0,044758	0,022	19	55
GOLGA8M	rs563033964	ms	-	136	Q/R	-0,507	0,044758	0,226	19	159
GOLGA8M	rs564738040	ms	-	217	E/Q	0,670	4,2E-05	0,000	59	195
GOLGA8S	rs777558701	ms	H3BPF8	635	R/G	-0,818	0,005821	0,984	10	0
GOLGA8T	rs1160569396	ms	H3BQL2	324	A/S	0,478	0,030840	0,011	33	0
GON4L	rs774683992	ms	Q3T8J9	1105	L/P	-0,442	0,033314	0,116	43	1,5
GON4L	rs756015284	ms	-	1821	P/S	-1,091	0,007105	0,007	12	1,1
GORASP1	rs145676119	ms	Q9BQQ3	302	V/I	0,602	0,000523	0,992	57	39
GOT2	rs1567487563	ms	P00505	225	Q/E	-0,770	0,036597	0,416	15	0
GP1BA	rs756370087	ms	P07359	222	P/A	1,948	0,011850	0,874	12	0,4
GP6	rs779914827	ms	Q9HCN6	360	R/Q	-0,716	0,051903	0,773	14	0
GP9	rs3796130	ms	P14770	156	A/T	1,198	0,046718	0,191	11	2150
GPATCH8	rs118151586	ms	Q9UKJ3	957	R/W	-0,448	0,016902	0,000	67	179
GPC1	rs137923533	ms	P35052	528	K/T	0,654	0,037541	0,070	16	32
GPD1L	rs72552293	ms	Q8N335	124	I/V	-0,392	0,046614	0,000	40	91
GPLD1	rs61754637	ms	P80108	103	N/S	-0,752	0,012543	0,786	13	125
GPR107	rs1564670898	ms	Q5VW38	265	L/V	0,511	0,026339	0,144	19	0
GPR108	rs368266462	ms	Q9NPR9	308	L/F	0,424	0,030025	0,358	39	3,0
GPR148	rs1332617530	ms	Q8TDV2	210	Q/H	-0,606	0,016837	0,003	19	0,8
GPR162	rs141534197	ms	Q16538	380	R/W	0,729	0,014786	0,349	13	50
GPR20	rs765239165	ms	Q99678	159	R/C	0,541	0,002123	0,041	44	1,9
GPR83	rs756043372	ms	Q9NYM4	51	D/G	0,469	0,013400	0,352	30	0,1
GPX1	rs769147194	ms	P07203	85	V/A	0,551	0,013208	0,001	44	1,0
GRAMD1B	rs200540342	ms	Q3KR37	507	V/I	0,414	6,62E-05	0,162	147	156
GRB14	rs779123855	ms	Q14449	358	S/N	-0,820	0,017937	0,010	17	0,1
GRB7	rs775378357	ms	Q14451	125	R/C	-0,433	0,035827	1,000	43	4,2
GRB7	rs758631692	ms	-	18	C/Y	0,514	0,004529	0,000	47	2,3
GREB1	rs760195039	ms	Q4ZG55	465	R/H	0,841	0,031107	0,937	11	1,3
GREB1	rs142904474	ms	-	1464	N/D	-0,432	0,050712	0,999	35	77
GREB1L	rs764201914	ms	Q9C091	1263	A/G	0,630	0,029345	0,095	18	32
GRIK1	rs73197503	ms	P39086	450	I/T	0,571	0,039277	0,739	22	116
GRIK5	rs766852259	ms	Q16478	127	R/H	-0,892	0,010232	0,851	12	1,5
GRIP1	rs776702622	ms	Q9Y3R0	1081	V/L	-0,863	0,025966	-	23	0,4
GRIP2	rs188992337	ms	Q9C0E4	9	T/I	0,469	0,017865	0,046	47	234
GRK7	rs34429284	ms	Q8WTQ7	81	R/H	0,616	0,008353	0,299	27	283
GRM3	rs776290250	ms	Q14832	102	D/Y	-1,100	0,034914	1,000	12	0,2
GRPEL1	rs753276386	ms	Q9HAV7	25	R/Q	-0,477	0,002695	0,883	61	1,9
GSAP	rs763484733	ms	A4D1B5	142	L/R	-0,551	0,029210	0,999	44	0,1
GSC2	rs73390724	ms	O15499	205	C/S	-0,493	0,010473	0,267	45	253
GSE1	rs771372000	ms	Q14687	230	D/N	0,726	0,020826	0,000	12	0,4
GSG1	rs374196061	ms	Q2KHT4	18	E/V	0,755	0,022384	0,965	15	5,3
GSR	rs151187899	ms	P00390	289	V/A	1,016	0,000033	0,987	29	148
GSR	rs200685394	ms	-	500	T/M	-0,451	0,037941	0,998	42	2,6
GSTK1	rs41275042	ms	Q9Y2Q3	161	T/M	-0,774	0,033967	0,717	16	34
GTF2IRD2B	rs1467815120	ms	Q6EKJ0	393	I/T	0,426	0,035733	0,999	34	0
GTF3C3	rs375124162	ms	Q9Y5Q9	802	R/Q	-0,402	0,004566	0,077	61	0,8
GTF3C5	rs150056568	ms	Q9Y5Q8	162	Q/K	-0,458	2,66E-06	0,763	155	77
GTF3C5	rs995906811	ms	-	248	R/W	-0,623	0,048089	0,806	15	0,5
GULP1	rs777660706	ms	Q9UBP9	206	S/N	0,637	0,009319	0,000	22	0,1
GYG1	rs140175164	ms	P46976	147	N/D	-0,467	0,031398	0,189	38	100
H2AC1	rs1159899764	ms	Q96QV6	63	I/V	-1,070	0,011965	0,000	11	1,4
H2AC11	rs765983355	ms	P0C0S8	129	G/D	0,713	0,040453	0,010	19	0,2
H2AC16	rs190008233	ms	-	83	H/R	-0,462	0,012271	0,511	40	8,2
H2AC4	rs139976301	ms	P04908	74	N/K	-0,793	0,031548	0,999	11	12
HADHA	rs772164983	ms	P40939	354	F/S	1,133	0,014997	0,653	11	0
HAL	rs137949606	ms	P42357	165	G/S	0,462	0,047890	0,998	21	14
HAL	rs1565990642	ms	-	343	G/V	1,008	0,030083	0,999	11	0,1
HAP1	rs142535684	ms	P54257	187	P/H	0,471	0,016691	0,127	46	113
HAPLN4	rs771835797	ms	Q86UW8	210	N/K	-0,443	0,037575	0,995	39	1,5
HCRTR1	rs199680510	ms	O43613	197	R/L	-2,238	0,000139	0,000	11	4,2
HDAC4	rs757619942	ms	P56524	185	A/V	-0,385	0,016945	0,814	61	5,7
HDAC4	rs745530862	ms	-	362	G/S	-1,438	0,002058	0,042	15	2,5
HDAC7	rs369848964	ms	Q8WUI4	681	A/T	0,654	0,047512	0,018	13	9,9
HDAC9	rs199944204	ms	Q9UKV0	167	G/A	0,621	0,020500	0,998	28	0,8
HEATR1	rs148574750	ms	Q9H583	783	V/M	0,604	0,030069	0,001	13	3,0
HEATR4	rs761598455	stop	Q86WZ0	191	W/*	-0,738	0,015735	1,000	26	4,2
HECTD4	rs748066013	ms	Q9Y4D8	3726	P/L	0,705	0,028014	0,199	21	3,0
HEG1	rs201404346	ms	Q9ULI3	342	T/M	-0,526	0,020948	0,011	39	40
HELZ	rs777941893	ms	P42694	625	T/S	-0,475	0,019221	0,014	51	0,4
HELZ	rs369453596	ms	-	1480	N/S	-0,636	0,006468	0,000	41	4,5
HELZ2	rs201125994	ms	Q9BYK8	824	G/S	-0,369	0,017372	0,009	68	6,8
HERC2	rs149493788	ms	O95714	876	A/V	0,811	0,039166	0,019	10	11
HERC6	rs374213974	ms	Q8IVU3	545	Q/E	-0,594	0,027431	0,007	16	0,4
HERPUD2	rs201373686	ms	Q9BSE4	104	H/Y	-0,973	0,036842	0,025	17	0,8
HGS	rs34868130	ms	O14964	400	E/D	-0,767	0,019232	0,003	13	1360
HIGD2A	rs752348331	ms	Q9BW72	47	P/L	-0,436	0,014798	0,981	51	0,4

HIKESHI	rs769149509	ms	Q53FT3	86	G/D	-0,515	0,003007	0,600	49	0,7
HIP1R	rs748671805	ms	O75146	809	R/H	-0,621	0,024931	0,993	25	2,3
HJURP	rs146575864	ms	Q8NCD3	585	D/N	-0,436	0,036637	0,989	28	45
HLCS	rs191115811	ms	P50747	72	K/M	0,752	0,041216	0,710	21	72
HLTF	rs749916690	ms	Q14527	320	I/N	0,611	0,023301	0,003	20	0,4
HLTF	rs769261535	ms	-	617	T/K	0,509	0,007557	0,770	47	8,3
HMBS	rs189159450	ms	P08397	22	R/C	0,651	0,024626	0,983	18	2,3
HMCN1	rs771301411	ms	Q96RW7	2028	P/S	0,714	0,017879	0,259	15	0,2
HMCN2	rs1564815835	fs	Q8NDA2	1945	V/X	-0,683	0,038865	1,000	23	0
HMGXB3	rs374393844	ms	Q12766	1169	T/M	0,622	0,045074	0,009	17	28
HNF1B	rs140562402	ms	P35680	82	D/N	0,374	0,044434	0,539	61	76
HNF1B	rs141193981	ms	-	532	M/V	-0,761	0,002714	0,978	26	17
HNRNPA1L3	rs756869693	ms	A0A2R8Y4L2	41	T/M	0,646	0,000441	0,163	47	6,4
HOOK1	rs1011037152	ms	Q9UJC3	16	D/E	0,357	0,037369	-	45	1,1
HOXB13	rs778047967	stop	Q92826	126	E/*	-0,572	0,000410	1,000	72	0,4
HOXB3	rs200264312	ms	P14651	284	P/S	0,365	0,040866	0,000	53	15
HRNR	rs764209221	ms	Q86YZ3	853	S/T	-0,671	0,033062	0,000	11	7,4
HRNR	rs1158627978	ms	-	1893	H/N	0,354	0,048229	0,000	38	0,7
HRNR	rs374494412	ms	-	807	Y/D	-0,671	0,033062	0,000	11	4,2
HSD17B1	rs61738799	ms	P14061	62	T/R	-0,360	0,038249	0,041	62	0,8
HSP90B1	rs144242572	ms	P14625	293	M/V	-0,549	0,013010	0,000	48	134
HTR7	rs114969659	ms	P34969	279	P/L	0,901	0,014548	0,026	10	180
HUNK	rs1349105042	ms	P57058	675	R/C	-0,861	0,013189	0,993	14	1,5
HVCN1	rs749906449	ms	Q96D96	162	R/C	0,494	0,023462	0,153	23	0,4
HYAL4	rs117488620	ms	Q2M3T9	477	R/Q	-0,525	0,013421	0,000	53	285
HYAL4	rs143985361	ms	-	404	S/G	-0,628	0,012993	0,147	19	170
HYDIN	rs778831543	ms	Q4G0P3	4151	I/V	-0,511	0,016457	-	41	4,2
IAH1	rs768894310	ms	Q2TAA2	173	C/S	-0,845	0,002448	0,093	19	0,8
IBTK	rs149695576	ms	Q9P2D0	1168	N/S	0,484	0,009750	0,005	37	22
ICE1	rs199992370	ms	Q9Y2F5	1026	R/S	-0,639	0,033751	0,041	15	9,8
ICOSLG	rs759358911	ms	O75144	199	V/M	1,690	0,015019	0,915	13	3,2
IDUA	rs781136336	ms	P35475	526	L/P	0,762	0,015288	0,975	17	41
IFFO1	rs1027116185	ms	Q0D2I5	188	S/L	-0,388	0,038344	0,024	60	0
IFNA5	rs769493970	ms	P01569	83	M/I	0,817	0,038985	-	19	0,2
IFNGR1	rs1887415	ms	P15260	467	L/P	2,081	0,000546	0,090	13	1603
IFNK	rs751052890	ms	Q9P0W0	3	T/I	0,753	0,004263	0,003	11	1,2
IFT172	rs149117098	ms	Q9UG01	1645	V/I	-0,393	0,050352	0,022	49	49
IFT56	rs772818942	ms	A0AVF1	162	Y/S	0,460	0,008833	0,952	60	1,9
IGF2R	rs8191808	ms	P11717	817	L/V	-0,496	0,019458	-	32	559
IGHMBP2	rs147038490	ms	P38935	790	R/Q	0,717	0,039389	0,012	36	34
IGLL1	rs8138122	ms	P15814	189	R/H	0,657	0,022017	0,006	18	3692
IGSF22	rs191927164	ms	Q8N9C0	1036	V/L	0,770	0,010599	0,938	23	35
IL10RA	rs202121581	ms	Q13651	262	R/H	-0,493	0,017318	0,007	40	6,8
IL27RA	rs772182491	ms	Q6UWB1	118	V/I	-0,735	0,001623	0,001	28	2,6
IMMP1L	rs146915142	ms	Q96LU5	116	E/Q	0,905	0,021772	0,603	14	27
INMT	rs201737289	ms	O95050	110	A/V	0,391	0,041941	0,000	50	11
INO80	rs766550330	ms	Q9ULG1	327	K/R	-0,410	0,040996	0,040	41	0,8
INO80D	rs768886465	ms	Q53TQ3	689	G/E	0,720	0,022210	0,202	18	0,4
INPPL1	rs17847215	ms	O15357	303	K/N	-0,747	0,017606	-	19	1218
INSC	rs556785106	ms	Q1MX18	227	R/C	-0,404	0,044130	0,824	43	1,1
INSR	rs768172890	ms	P06213	690	E/V	-0,935	0,000131	0,000	30	0,8
INTS14	rs369418631	ms	Q96SY0	367	A/T	-0,996	0,035397	0,000	10	1,5
IPO4	rs747440936	ms	Q8TEX9	182	R/C	1,054	0,038898	0,870	12	0,7
IQCA1L	rs199771708	ms	A6NCM1	209	A/T	0,444	0,050860	0,978	42	147
IQCC	rs765596362	ms	Q4KMZ1	182	N/S	-0,756	0,039833	0,497	30	0,4
IQCD	rs60284450	ms	Q96DY2	19	R/K	-0,412	0,026384	0,005	52	1718
IQGAP2	rs759083181	ms	Q13576	786	K/R	-0,407	0,046218	-	49	0,2
IRF3	rs1568461744	ms	Q14653	101	H/D	-1,830	0,000084	0,841	11	0
IRS2	rs1046261311	ms	Q9Y4H2	446	G/S	-0,537	0,024641	-	24	3,4
ISX	rs7291048	ms	Q2M1V0	158	A/V	1,265	0,015182	0,191	13	1181
ITGA11	rs368307734	ms	Q9UKX5	936	R/Q	-0,691	0,046327	0,000	15	3,4
ITGA6	rs778993295	ms	P23229	1019	L/P	0,603	0,036043	0,862	16	1,1
ITGAV	rs754174458	ms	P06756	428	R/L	-0,722	0,050363	0,000	10	2,3
ITGAV	rs781221559	ms	-	131	K/Q	-0,656	0,050991	0,007	15	0
ITPRID1	rs137892596	ms	Q6ZRS4	958	H/P	0,514	0,047501	0,009	25	15
ITSN1	rs1164637837	ms	Q15811	1588	R/H	-0,389	0,007964	-	78	0,4
JAK3	rs747131454	ms	P52333	651	R/W	-0,845	0,014988	0,050	16	0,4
JCAD	rs184216138	ms	Q9P266	1074	I/T	1,115	0,004479	0,039	12	56
JMY	rs759301275	ms	Q8N9B5	397	R/Q	-0,367	0,028494	0,358	45	0,4
JUP	rs374008304	ms	P14923	50	G/R	-0,979	0,045561	0,000	11	3,4
KANK1	9:738430:G/C	ms	Q14678	1160	G/A	0,707	0,033633	0,750	20	N/A
KANK4	rs377253083	ms	Q5T7N3	185	P/S	0,933	0,028470	0,001	11	6,6
KANSL1	rs151099014	ms	Q7Z3B3	765	A/V	0,494	0,026922	0,000	43	46
KANSL1	rs780685635	ms	-	943	R/Q	-0,786	0,031409	0,932	11	0,1
KAT6A	rs1291997106	ms	Q92794	311	R/L	0,742	0,027106	-	15	0,8
KAT6B	rs1311062859	ms	Q8WYB5	1242	L/P	-0,992	0,030772	-	19	0,4
KAT8	rs185459113	ms	Q9H7Z6	446	R/Q	-1,639	0,026243	-	11	89
KATNIP	rs750193636	ms	O60303	1036	D/N	0,360	0,040810	0,876	29	7,6
KCNA10	rs1159665771	ms	Q16322	407	S/N	-0,634	0,013252	0,982	26	0,2
KCNA3	rs779056951	ms	P22001	531	V/L	0,522	0,051738	0,014	39	0,4

KCNH6	rs369961665	ms	Q9H252	450	G/S	-0,440	0,000141	0,009	129	1,9
KCNH6	rs148230267	ms	-	726	R/Q	0,380	3,74E-06	0,000	258	11
KCNJ16	rs761312168	ms	Q9NPI9	115	V/I	0,765	0,027167	0,988	11	3,8
KCNK13	rs200021633	ms	Q9HB14	148	R/H	-0,960	0,024121	0,998	13	0,8
KCNK5	rs41273124	ms	O95279	365	K/I	-0,554	0,033594	0,136	18	197
KCNT1	rs753401695	ms	Q5JUK3	674	M/V	1,002	0,007134	0,001	14	3,0
KCNT1	rs566157365	ms	-	657	P/L	0,467	0,014815	0,001	49	1,1
KCNU1	rs775599941	ms	A8MYU2	1121	P/L	1,014	0,051835	0,000	12	4,2
KCNV1	rs1035112026	ms	Q6PIU1	474	R/W	0,511	0,043188	0,476	20	0,2
KCNV2	rs147022958	ms	Q8TDN2	254	F/L	0,392	0,022353	0,036	52	0
KCTD1	rs192197412	ms	Q719H9	106	E/V	-1,059	0,000749	0,000	21	114
KCTD13	rs1167342922	ms	Q8WZ19	40	S/N	-0,571	0,027479	0,059	25	0,4
KCTD20	rs748850127	ms	Q7Z5Y7	195	I/V	-0,472	0,017077	0,007	45	2,6
KCTD8	rs138935237	ms	Q6ZWB6	137	F/Y	-0,825	0,002433	1,000	30	23
KDM4E	rs182574403	ms	B2RXH2	110	P/L	-0,434	0,037430	0,998	49	38
KDM6B	rs748896517	ms	O15054	212	A/V	-0,419	0,027861	0,494	41	0,6
KDM8	rs145879289	ms	Q8N371	156	R/H	-0,724	0,037239	0,000	16	2,9
KIAA0232	rs780512122	ms	Q92628	1060	P/S	-0,476	0,002782	0,996	61	0,4
KIAA1671	rs6004404	ms	Q9BY89	1400	R/W	-0,449	0,016244	0,003	33	17
KIAA1755	rs202213297	ms	Q5JYT7	486	P/L	0,665	0,034246	0,370	17	4,7
KIF13B	rs761773190	ms	Q9NQT8	369	R/Q	-0,453	0,027397	0,943	56	2,3
KIF13B	rs200053841	ms	-	1812	D/N	-0,640	0,033592	0,015	20	49
KIF14	rs145426227	ms	Q15058	1207	Q/R	-0,375	0,031677	0,000	41	20
KIF14	rs771069928	ms	-	1583	E/G	-1,025	0,009378	0,010	13	0
KIF15	rs149445851	ms	Q9NS87	1120	E/G	0,790	0,031478	0,170	11	41
KIF15	rs112882527	ms	-	938	V/I	-0,695	0,022159	0,003	18	73
KIF15	rs776781117	ms	-	842	V/I	-0,390	0,040532	0,000	36	3,8
KIF16B	rs200695292	ms	Q96L93	1200	A/S	-0,787	0,048349	0,057	12	14
KIF16B	rs192942360	ms	-	1315	G/R	-0,953	0,025066	0,999	11	15
KIF16B	rs565686380	ms	-	1274	V/M	0,405	0,017903	0,784	52	0,7
KIF16B	rs759579752	stop	-	1584	R/*	-0,395	0,040948	1,000	32	6,0
KIF19	rs61746578	ms	Q2TAC6	891	R/W	-0,496	0,011660	0,891	40	1331
KIF24	rs556845604	ms	Q5T7B8	497	P/R	-0,480	0,032741	0,997	22	0,7
KIF26A	rs774219038	ms	Q9ULI4	695	R/H	-0,529	0,042261	0,868	26	8,3
KIF26B	rs762051944	ms	Q2KJY2	463	R/Q	0,718	0,051657	0,052	17	0
KIF5C	rs749228157	ms	O60282	57	T/M	0,419	0,026273	0,958	48	0,4
KIF7	rs151317163	ms	Q2M1P5	746	R/Q	-0,610	0,019396	0,000	18	58
KIR2DL1	rs145961423	ms	P43626	141	A/T	0,537	0,043491	0,014	20	102
KIRREL1	rs762885869	ms	Q96J84	377	R/Q	0,427	0,033668	0,706	34	19
KLF18	rs768832850	ms	A0A0U1RQI7	969	E/K	-0,352	0,026052	0,017	72	1,1
KLHDC4	rs757939376	ms	Q8TBB5	432	R/H	0,677	0,007467	0,999	21	2,3
KLHDC4	rs74931760	ms	-	229	T/A	0,898	0,034908	0,009	13	1170
KLHL1	rs144867124	ms	Q9NR64	4	S/C	0,358	0,038566	0,996	50	44
KLHL14	rs143044789	ms	Q9P2G3	172	L/F	0,480	0,008325	0,005	58	1,1
KLHL18	rs374972341	ms	O94889	292	A/T	-1,039	0,004474	0,997	18	45
KLHL29	rs910946041	ms	Q96CT2	205	S/L	0,782	0,005359	0,028	22	1,1
KLHL5	rs758807194	ms	Q96PQ7	369	A/V	0,568	0,041489	0,224	19	0,6
KLHL5	rs767326522	ms	-	280	R/C	-0,636	0,043276	0,998	22	0,8
KLK14	rs201296079	ms	Q9P0G3	203	D/G	-0,762	0,028208	1,000	13	230
KLK14	rs1318729894	start_lost	-	1	M/K	0,578	0,037013	0,234	16	1,9
KLRC3	rs61743085	ms	Q07444	228	R/W	-0,874	0,044607	0,685	15	6,0
KMT2A	rs748830529	ms	Q03164	3146	Q/R	0,385	0,012663	0,163	54	0
KMT2D	rs747613578	ms	O14686	2207	A/V	-0,458	0,005881	0,000	72	16
KMT5A	rs1309240986	ms	Q9NQR1	37	P/S	-1,555	0,022263	0,621	14	0,4
KNL1	rs369348708	ms	Q8NG31	1148	I/V	0,417	0,049210	0,009	21	7,2
KPTN	rs142867197	ms	Q9Y664	63	R/Q	0,455	0,006389	0,048	66	137
KRI1	rs755712390	ms	Q8N9T8	50	D/V	-0,367	0,001517	0,473	102	3,8
KRT13	rs144967807	ms	P13646	426	V/I	0,569	0,012923	0,000	41	104
KRT19	rs149987744	ms	P08727	327	T/M	0,645	0,033629	0,975	24	96
KRT23	rs1567799084	ms	Q9C075	183	D/G	0,539	0,027269	1,000	19	0,3
KRT31	rs1567681376	ms	Q15323	378	C/F	-0,564	0,048518	0,142	24	0
KRT32	rs117304287	ms	Q14532	99	T/I	0,695	0,017061	1,000	17	186
KRT7	rs138391469	ms	P08729	64	R/C	-0,436	0,044907	0,672	55	76
KRT71	rs749611724	ms	Q3SY84	205	E/G	-0,473	0,011448	0,987	42	0,4
KRT9	rs374710110	ms	P35527	326	M/I	0,386	0,008387	0,952	67	0,4
KRTAP4-12	rs368157227	ms	Q9BQ66	31	C/R	-0,747	0,031087	0,000	17	12
KRTAP4-8	rs777688954	ms	Q9BYQ9	176	R/H	-0,357	0,026763	0,000	73	2,9
KSR2	rs376902515	ms	Q6VAB6	373	A/T	-0,493	0,023456	0,000	32	11
L2HGDH	14:50267794:A/C	ms	Q9H9P8	341	F/L	0,739	0,032981	0,009	16	N/A
L3HYPDH	rs776937282	ms	Q96EM0	66	M/L	-0,648	0,029111	0,012	14	0,8
LACTBL1	rs780707524	stop	A8MY62	515	Q/*	-0,403	0,028944	1,000	53	34
LAMA4	rs767693296	ms	Q16363	229	Y/C	0,391	0,002163	0,419	124	0,3
LAMB1	rs766731970	ms	P07942	445	Y/C	-1,216	0,042208	0,989	10	0,4
LAMB2	rs760500807	ms	P11047	532	P/L	0,365	0,049489	0,134	44	0
LAMB2	rs760185781	ms	-	37	P/L	-0,946	0,016490	0,000	16	0,2
LAMB3	rs767004520	ms	Q13751	552	R/C	0,594	0,041677	0,975	22	7,9
LAMB3	rs747338921	ms	-	907	A/D	-1,040	0,018506	0,791	12	0,7
LAMB4	rs755389093	ms	A4D0S4	712	Q/R	-0,670	0,000170	0,000	56	0
LAMB4	rs758145192	stop	-	942	Q/*	0,691	0,010285	1,000	22	1,1
LAMC1	rs150421474	ms	-	1435	A/T	0,546	0,020550	0,000	19	41

LANCL2	rs758368934	ms	Q9NS86	88	M/V	-0,413	0,005007	0,052	58	0
LARGE2	rs376236828	ms	Q8N3Y3	426	P/S	0,517	0,002353	0,003	51	6,0
LARP1B	rs371480626	ms	Q659C4	748	H/Y	0,633	0,023153	0,811	16	0,4
LBR	rs773059198	ms	Q14739	310	G/R	0,464	0,018569	0,878	32	19
LCOR	rs1247398150	ms	Q96JN0	373	S/N	-0,662	0,030349	0,528	17	0,8
LDHAL6A	rs139641299	ms	Q6ZMR3	288	F/L	-0,905	0,033644	0,999	11	155
LDHB	rs746464388	ms	P07195	181	I/V	0,547	0,013969	0,059	27	0,1
LENG1	rs150820847	ms	Q96BZ8	176	S/R	0,563	0,051777	0,000	25	127
LETM1	rs115233072	ms	O95202	587	K/R	-0,970	0,000796	0,986	20	1712
LGALS4	rs748499184	ms	P56470	41	E/Q	-0,935	0,027414	0,010	13	0
LIG4	rs756725710	ms	P49917	208	H/R	0,692	0,041797	1,000	22	0,3
LINGO4	rs61746299	ms	Q6UY18	444	T/S	0,464	0,047103	0,089	43	7045
LIPA	rs762796693	ms	P38571	4	R/Q	-0,508	0,051383	0,001	26	0,8
LIPF	rs17333991	ms	P07098	348	P/S	-0,439	0,017173	0,854	48	208
LIPG	rs748470810	ms	Q9Y5X9	212	D/N	0,537	0,000592	0,893	55	0,4
LIPH	rs1720462199	ms	Q8WWY8	43	L/V	0,826	0,051507	0,992	16	0,4
**LLGL1**	rs761027899	ms	Q15334	329	E/K	-1,410	0,009989	0,001	11	2,3
**LLGL1**	rs200829360	ms	-	11	A/T	0,391	0,006363	0,000	97	265
LMF2	rs373075701	ms	Q9BU23	177	R/C	0,936	0,001848	1,000	19	12
LOC112267897	rs148363228	ms	A0A3B3IRQ3	89	G/A	0,492	0,026246	0,000	40	4849
LONRF1	rs200496434	ms	Q17RB8	457	V/I	0,660	0,045201	0,000	30	59
LOXHD1	rs754654876	ms	Q8IVV2	1572	R/Q	1,369	0,001659	0,918	14	0,8
LPCAT3	rs200922476	ms	Q6P1A2	331	T/I	-0,578	0,003930	0,409	38	12
LRP1	rs150485916	ms	Q07954	2015	V/I	0,531	0,040819	0,974	23	12
LRP1B	rs754422175	ms	Q9NZR2	104	P/L	0,788	0,001864	0,005	34	3,8
**LRP2**	rs1365452628	ms	P98164	3815	L/M	-0,657	0,030641	0,080	15	1,5
**LRP2**	rs140022108	ms	-	3726	R/C	0,751	0,040530	1,000	19	1,1
LRP6	rs773818278	ms	O75581	356	L/S	-0,749	0,003452	0,204	34	0,2
**LRP8**	rs139703435	ms	Q14114	725	M/R	-1,016	0,010003	0,246	10	31
**LRP8**	rs201950519	ms	-	571	N/S	0,684	0,049347	0,993	15	20
LRPPRC	rs771539737	ms	P42704	1306	S/T	0,470	0,040782	0,009	43	0,1
LRRC25	rs772139017	ms	Q8N386	107	R/H	0,430	0,035373	0,031	32	1,5
LRRC37A	17:46297037:T/C	ms	A6NMS7	635	I/T	0,564	0,018485	0,012	32	N/A
**LRRC37A2**	17:46514616:T/C	ms	A6NM11	635	I/T	0,678	0,006997	0,000	27	N/A
**LRRC37A2**	17:46513049:T/C	ms	-	113	W/R	-0,695	0,039575	0,030	17	N/A
**LRRC37A3**	rs77916588	ms	O60309	1130	V/I	-0,453	4,17E-05	0,017	144	505
**LRRC37A3**	rs1463425308	ms	-	483	P/L	0,406	0,001153	0,998	95	1,2
**LRRC37A3**	rs200759066	ms	-	17	R/H	0,558	0,047344	0,370	17	5,4
LRRC39	rs140255458	ms	Q96DD0	72	W/R	-0,443	0,021005	0,997	51	24
LRRC41	rs774231085	ms	Q15345	39	G/C	0,585	0,034917	0,742	17	12
**LRRC46**	rs146740911	ms	Q96FV0	242	V/M	-0,568	0,044504	0,142	26	55
**LRRC46**	17:47835758:T/C	ms	-	122	I/T	-0,896	0,030450	0,990	14	N/A
LRRC66	rs201283430	ms	Q68CR7	11	I/V	-0,722	0,028597	0,030	19	2,0
LRRC7	rs144289156	ms	Q96NW7	1160	A/T	-0,451	0,015763	0,056	53	19
LRRFIP1	rs773197164	ms	Q32MZ4	314	T/A	0,495	0,044210	0,421	18	0,4
LRRFIP2	rs776740745	ms	Q9Y608	114	F/L	0,548	0,035700	0,000	18	2,6
LRRIQ1	rs143253287	ms	Q96JM4	835	S/G	0,615	0,047287	0,000	23	96
LRRN2	rs374611937	ms	O75325	577	R/Q	-1,001	0,002439	0,001	22	7,6
**LSR**	rs771635414	ms	Q86X29	579	D/N	0,409	0,002513	0,355	104	0,5
**LSR**	rs749613670	ms	-	576	S/W	0,435	0,046716	0,874	21	0
LTBP3	rs770492133	ms	Q14767	549	P/R	0,818	0,026933	0,001	14	0,7
LVRN	rs747264765	ms	Q6Q4G3	768	R/H	-0,674	0,025079	0,986	19	1,1
LY6E	rs199552296	ms	Q16553	81	V/I	-0,684	0,018000	0,003	28	12
LY75	rs201265039	ms	O60449	48	K/E	0,656	0,014886	0,084	17	0,4
**LZTS3**	rs1391065205	ms	O60299	645	G/R	-0,717	0,051715	0,251	13	0,4
**LZTS3**	rs759999966	ms	-	199	G/R	0,372	0,050002	0,955	53	1,4
MACF1	rs140978060	ms	Q9UPN3	1734	A/V	-0,583	0,049995	0,162	14	34
MACIR	rs140904641	ms	Q96GV9	88	M/L	0,362	0,046181	0,000	35	0,7
MACROH2A2	rs1489682038	ms	Q9P0M6	22	I/V	0,557	0,036316	0,125	18	0,2
MAD1L1	rs200917713	ms	Q9Y6D9	696	R/H	0,512	0,001665	0,001	50	74
MADD	rs755791135	ms	Q8WXG6	831	P/A	-0,672	0,007887	0,999	31	1,5
MAG	rs778697016	ms	P20916	624	R/Q	-0,598	0,015200	0,645	21	5,1
MAGEL2	rs1566783726	ms	Q9UJ55	944	S/N	-1,094	0,019026	0,020	11	0,9
MAGI2	rs145648453	ms	Q86UL8	1304	G/D	-0,778	0,003904	-	16	197
MAGI3	rs1557839712	ms	Q5TCQ9	300	E/A	0,626	0,030652	0,999	16	0,4
MAN2B2	rs142638040	ms	Q9Y2E5	975	R/H	-0,660	0,045838	0,001	13	12
**MANSC1**	rs748098987	ms	Q9H8J5	301	A/T	0,378	0,018408	0,717	71	2,3
**MANSC1**	rs370523906	ms	-	243	K/N	-0,679	0,031011	0,532	15	16
MAP1A	rs200211262	ms	P78559	1185	R/C	-0,525	0,050933	0,255	25	18
MAP3K1	rs764525244	ms	Q13233	504	E/K	1,291	0,031982	0,447	14	4,2
MAP3K13	rs199572011	ms	O43283	474	R/Q	0,958	0,020641	0,078	13	12
**MAP3K19**	rs56349597	ms	Q56UN5	1154	R/H	-0,458	0,035948	0,997	46	63
**MAP3K19**	rs3905317	ms	-	812	E/G	-0,475	0,012722	0,826	56	2713
**MAP3K19**	rs1112542	ms	-	676	E/Q	-0,475	0,012721	0,089	58	6756
MAP3K21	rs767156199	ms	Q5TCX8	1029	E/Q	0,382	0,013969	0,003	59	1,2
MAP3K3	rs774603132	ms	Q99759	185	R/L	-0,912	0,048087	0,028	11	0,7
MAP6	rs770189526	ms	Q96JE9	701	P/S	-0,707	0,008969	0,180	24	0
MAPK3	rs375146669	ms	P27361	31	V/G	-0,383	0,022901	0,235	67	0,4
MAPK8IP2	rs1569064809	ms	Q13387	421	A/T	-1,040	0,027579	0,000	18	0,7
MAPK8IP3	rs201803478	ms	Q9UPT6	1273	S/L	-0,496	0,035550	0,000	27	33

MARCHF10	rs140159918	ms	Q8NA82	123	S/N	0,357	0,000233	0,054	191	**14**
MARCHF10	rs200034579	ms	-	309	C/Y	-0,422	0,003276	0,000	**87**	**39**
MARF1	rs1567589768	ms	Q9Y4F3	215	L/V	0,724	0,007106	**0,991**	**19**	0
MARF1	rs761713203	ms	-	1631	S/L	-0,568	0,003283	**0,998**	**22**	0,8
MARK3	rs56157932	ms	P27448	468	A/V	0,679	0,024066	0,051	**15**	3,0
MBD1	rs142015383	ms	Q9UIS9	315	E/K	**1,077**	0,001920	**0,712**	**15**	**41**
MBD3	rs371220154	ins	O95983	278-279	-/E	0,793	0,005774	-	**26**	**3385**
MBLAC1	rs199848609	ms	A4D2B0	231	H/Q	0,516	0,031111	**0,995**	**20**	1,9
MBLAC2	rs147831525	ms	Q68D91	42	V/L	0,393	0,027521	0,009	**49**	**29**
MBTPS1	rs776110165	ms	Q14703	332	M/I	-0,588	0,001421	**0,637**	**43**	5,3
MBTPS1	rs768417679	ms	-	367	I/M	-0,480	0,039689	**0,872**	**22**	0,8
MCC	rs75148264	ms	P23508	183	A/T	0,589	0,034567	0,007	**15**	197
MCEE	rs111033538	stop	Q96PE7	47	R/*	**1,215**	0,009228	**1,000**	**11**	**30**
MCF2L	rs145119738	ms	O15068	564	S/F	-0,602	0,030563	**0,796**	**25**	110
MCF2L2	rs773833576	ms	Q86YR7	376	K/E	**-1,063**	0,012535	0,215	**12**	0,4
MCM2	rs376214773	ms	P33993	198	R/H	-0,504	0,045674	**0,987**	**26**	1,5
MCM4	rs755560078	ms	P33991	487	G/R	-0,476	0,051773	**0,992**	**27**	0,8
MCM7	rs747875490	ms	-	455	R/H	0,681	0,018502	**0,998**	**19**	0,6
MCM8	rs764765085	ms	Q9UJA3	726	L/M	0,385	0,029704	0,037	**69**	0
MCM9	rs553491848	ms	Q9NXL9	563	R/Q	0,527	0,027734	0,151	**24**	5,6
MCM9	rs182607789	ms	-	882	H/R	0,586	0,020552	0,000	**28**	1,1
MCM9	rs185696063	ms	-	715	D/V	0,518	0,025751	0,213	**38**	166
MCMDC2	rs1563368437	ms	Q4G0Z9	165	I/M	0,420	0,042213	0,318	**35**	0
MCOLN1	rs760411989	ms	Q9GZU1	472	D/N	-0,542	0,043040	**0,988**	**15**	0,5
MCU	rs766307644	ms	Q8NE86	351	D/H	-0,792	0,039889	**0,993**	**14**	1,3
MDGA2	rs1566621686	ms	Q7Z553	377	Y/C	0,776	0,033505	**1,000**	**19**	0,4
MDK	rs750294696	ms	P21741	122	V/I	-0,543	0,044057	**0,673**	**32**	0,4
MDN1	rs1562203020	ms	Q9NU22	664	A/V	-0,722	0,028505	**0,832**	**15**	0,2
MECR	rs1558480060	ms	Q9BV79	110	A/V	-0,653	0,025026	**0,492**	**20**	0
MED13	rs35998015	ms	Q9UHV7	39	T/A	-0,584	0,033484	**0,896**	**26**	218
MED24	rs775765230	ms	O75448	789	P/S	0,662	0,015664	**1,000**	**31**	0,8
MED27	rs780440896	ms	Q6P2C8	211	R/Q	-0,447	0,051771	**0,560**	**24**	0,3
MED30	rs770113741	ms	Q96HR3	142	I/L	-0,479	0,048220	0,003	**43**	0,2
MEF2B	rs781653583	ms	Q02080	308	G/S	-0,512	0,045871	0,003	**27**	0,8
MEFV	rs104895112	ms	O15553	329	R/H	0,459	0,015755	0,000	**56**	124
MEIOC	rs745376874	ms	A2RUB1	934	H/R	0,768	0,026627	0,003	**11**	0,8
MELK	rs146537035	ms	Q14680	331	R/H	0,420	0,029377	0,031	**37**	**16**
MEP1B	rs751683259	ms	Q16820	511	R/C	-0,741	0,001473	**0,975**	**37**	0,8
MEX3D	rs949994849	del	Q86XN8	133	P/-	-0,378	0,017986	-	**64**	1,4
MFN2	rs387906991	ms	O95140	362	T/M	0,728	0,035929	**0,930**	**15**	4,2
MFSD5	rs142428878	ms	Q6N075	278	G/R	0,715	0,014290	**0,552**	**19**	**16**
MFSD5	rs751354324	ms	-	85	Y/F	-0,485	0,048668	0,003	**20**	0,6
MFSD6L	rs367887663	ms	Q8IWD5	323	H/D	0,505	3,46E-08	**0,902**	166	7,2
MGA	rs759572856	ms	Q8IWI9	2622	L/V	-0,409	0,041401	**0,991**	**41**	0,8
MGAM	rs1563229730	ms	O43451	2634	I/T	-0,446	0,044981	**0,941**	**29**	0
MGAT4B	rs776812428	ms	Q9UQ53	495	R/W	0,543	0,049365	**0,850**	**21**	1,1
MIA2	rs147941751	ms	Q96PC5	569	S/P	0,579	0,045106	0,092	**14**	**33**
MICAL2	rs376168249	ms	O94851	740	R/H	0,667	0,035817	-	**16**	3,8
MICALL2	rs138933154	ms	Q8IY33	532	P/S	0,552	0,001273	-	**47**	3,0
MIER1	rs759562232	ms	Q8N108	158	D/Y	0,480	0,004103	**0,711**	**55**	1,1
MIER1	rs189050888	ms	-	460	S/P	-0,358	0,048007	0,370	**65**	181
MILR1	rs1352161075	ms	Q7Z6M3	159	T/I	0,376	1,22E-10	0,035	539	3,4
MIPOL1	rs749772798	ms	Q8TD10	306	N/S	-0,628	0,037037	0,337	**15**	1,1
MITF	rs542163629	ms	O75030	377	R/Q	-0,806	0,015633	0,413	**18**	2,6
MIXL1	rs1471523698	ms	Q9H2W2	49	P/S	0,648	0,049749	**0,650**	**19**	0
MKI67	rs1565001712	stop	P46013	2225	Q/*	**1,181**	0,011340	**1,000**	**16**	0,1
MKI67	rs61729196	ms	-	1059	A/E	0,514	0,015813	-	**45**	**82**
MKNK1	rs764595768	ms	Q9BUB5	226	D/N	0,811	0,031747	**1,000**	**21**	1,5
MKS1	rs199832333	ms	Q9NXB0	40	H/Y	0,371	0,042693	0,034	**49**	**59**
MLH3	rs61752722	ms	Q9UHC1	809	M/V	0,546	0,036119	0,000	**31**	182
MLLT3	rs767771641	ms	P42568	416	E/K	-0,501	0,037361	**0,597**	**31**	0,4
MLST8	rs766259271	ms	Q9BVC4	117	R/Q	0,840	0,048451	0,053	**13**	2,2
MLXIP	rs777847505	ms	Q9HAP2	161	R/Q	-0,402	0,029331	**0,970**	**37**	0,1
MMD2	rs761881427	ms	Q8IY49	172	P/L	-0,492	0,024161	**1,000**	**33**	0,1
MME	rs769414577	ms	P08473	77	R/G	0,919	0,015592	0,000	**24**	0,4
MMP20	rs17099008	ms	O60882	169	I/L	-0,440	0,000324	-	**96**	**1045**
MMP20	rs61730849	ms	-	130	T/I	-0,591	0,049882	-	**26**	**99**
MMP21	rs554501102	ms	O75900	97	A/V	**-1,789**	0,002314	0,392	**11**	**60**
MMP3	rs148579119	ms	P08254	153	Y/H	0,449	0,004839	-	**73**	232
MMRN1	rs763071708	ms	Q13201	62	S/L	0,537	0,039639	0,006	**13**	0,4
MMRN1	rs745711366	stop	-	177	R/*	-0,943	0,031835	**1,000**	**16**	0,8
MMRN2	rs200500870	ms	Q9H8L6	53	V/I	-0,373	0,011629	0,001	**71**	1,9
MN1	rs200030766	ms	Q10571	19	G/D	0,440	0,026800	**0,982**	**50**	114
MN1	rs529726950	ms	-	58	P/T	-0,756	0,040886	**0,478**	**14**	0,7
MN1	rs1568986531	ms	-	618	F/L	-0,676	0,001428	0,079	**57**	0
MOB3A	rs201880293	ms	Q96BX8	55	E/K	-0,499	0,031221	-	**21**	4,9
MPHOSPH9	rs147793766	ms	Q99550	447	T/A	-0,454	0,008938	0,037	**56**	254
MPIG6B	rs530881306	ms	O95866	173	P/Q	0,529	0,015135	0,003	**27**	1,3
MPP2	rs76832034	ms	Q14168	261	I/M	0,484	0,042905	**0,995**	**44**	128
MRAP2	rs545003385	ms	Q96G30	106	R/H	0,433	0,010710	0,001	**57**	6,8

MRC1	rs368257559	ms	P22897	295	R/C	-0,494	0,002556	0,652	47	1,3
MRC2	rs776632141	ms	Q9UBG0	1439	R/C	-0,584	0,003967	0,998	30	3,0
MRC2	rs751681043	ms	-	1459	P/S	0,637	0,000527	0,154	36	0,4
MRC2	rs755074437	ms	-	547	R/H	-0,973	5,91E-06	0,826	29	2,6
MRC2	rs201959743	ms	-	114	R/W	0,397	0,000104	0,900	169	32
MRC2	rs146172137	ms	-	352	S/R	0,391	9,07E-06	0,951	203	111
MRC2	rs749626570	ms	-	1054	M/I	-0,442	0,000327	0,007	118	0,7
MRGPRX3	rs749858617	ms	Q96LB0	55	R/H	-0,682	0,023600	0,078	10	5,4
MROH2A	rs770367514	ms	A6NES4	997	T/I	0,500	0,012736	0,007	39	0,1
MROH2B	rs563729861	ms	Q7Z745	868	A/T	-0,637	0,049621	0,990	26	5,3
MROH7	rs775705383	ms	Q68CQ1	623	D/V	-0,561	0,044513	0,029	27	1,7
MROH9	rs368219054	ms	Q5TGP6	179	C/W	0,475	0,047506	0,988	13	1,5
MRPL10	rs941871889	ms	Q7Z7H8	11	P/L	-0,792	0,000694	0,000	33	6,7
MRPL20	rs775815488	ms	Q9BYC9	126	E/K	1,240	0,006840	0,996	19	1,1
MRPL32	rs148103531	ms	Q9BYC8	18	G/E	0,357	0,037544	0,061	59	14
MRPL45	rs781545260	ms	Q9BRJ2	30	A/V	0,864	0,024341	0,024	11	1,2
MRPS12	rs760757324	ms	O15235	97	G/R	-0,357	0,018666	0,985	74	0,2
MS4A8	rs748203143	ms	Q9BY19	143	I/V	-0,392	0,003871	0,567	71	0
MSH3	rs778661757	ms	P20585	908	Q/P	0,367	0,033334	0,934	46	0,8
MSS51	rs372657650	ms	Q4VC12	242	R/Q	-0,408	0,038662	0,045	52	7,2
MST1R	rs149428633	ms	Q04912	778	N/T	-0,800	0,030054	0,007	19	140
MST1R	rs373976686	ms	-	1305	R/H	-0,569	0,008189	0,999	50	3,0
MTAP	rs775627882	ms	Q13126	274	A/T	-0,486	0,004044	0,054	58	0,8
MTCL1	rs34690009	ms	Q9Y4B5	879	M/V	-0,386	0,029320	0,423	49	1206
MTCL1	rs147496732	ms	-	728	R/C	1,525	0,003417	1,000	12	2,3
MTCL2	rs190032659	ms	O94964	1052	R/W	-0,428	0,022210	0,990	30	40
MTMR4	rs369444194	ms	Q9NYA4	127	R/W	-0,514	0,000037	0,994	86	9,1
MTMR7	rs370669270	ms	Q9Y216	131	D/N	-0,629	0,009392	0,001	28	16
MTOR	rs778866134	ms	P42345	534	D/N	-0,620	0,000863	0,122	36	3,0
MTOR	rs748631718	ms	-	173	R/C	-0,939	0,002697	0,892	15	1,1
MTPAP	rs151258340	ms	Q9NVV4	152	K/M	0,494	0,005145	0,852	40	1,9
MTPAP	rs765614188	ms	-	170	P/A	0,950	0,000770	0,005	20	0
MTR	rs774231870	ms	Q99707	674	R/H	0,976	0,021926	0,999	12	0,4
MUC12	rs548320109	ms	Q9UKN1	1353	P/L	-0,569	0,005400	0,000	37	0,8
MUC16	rs200135063	ms	Q8WXI7	6559	A/E	0,395	0,009397	0,000	78	123
MUC16	rs752215341	ms	-	6148	V/F	0,530	0,022957	0,000	24	0,8
MUC16	rs768836638	ms	-	1615	T/I	-0,725	0,048685	0,617	10	0,4
MUC16	rs751898712	ms	-	6623	T/R	-0,783	0,000263	0,000	39	0,7
MUC16	rs142323715	ms	-	13218	V/I	-0,773	0,049538	0,253	17	831
MUC16	rs998924452	ms	-	7103	T/I	-0,783	0,000263	0,000	39	0,1
MUC17	rs377637063	ms	Q685J3	723	T/N	0,376	0,016549	0,000	50	6,8
MUC17	rs374623804	stop	-	2753	L/*	-0,459	0,041900	1,000	36	5,7
MUC20	rs115826755	ms	Q8N307	672	P/L	0,887	0,004812	0,000	14	130
MUC4	rs148587168	stop	Q99102	5215	R/*	0,521	0,019339	1,000	27	58
MUC5AC	rs1325110644	ms	P98088	3523	R/Q	0,862	0,002865	0,007	23	6,7
MUC5AC	rs1324038977	ms	-	1773	I/F	-0,826	0,049518	0,677	14	2,8
MUC5AC	rs1564913808	ms	-	2250	S/F	-0,829	0,045330	0,000	13	0
MUC5AC	rs1428522876	ms	-	3956	R/Q	0,862	0,002866	0,007	23	0,4
MUC5B	rs190158159	ms	Q9HC84	4893	T/R	0,432	0,016783	0,000	49	28
MUS81	rs1178001089	ms	Q96NY9	143	L/Q	0,464	0,041881	0,999	33	0,4
MYBL1	rs191885262	ms	P10243	365	I/T	-0,538	0,028764	-	33	6,0
MYBPC1	rs747245712	ms	Q00872	608	T/P	0,357	0,025341	-	89	0
MYBPC1	rs750820996	ms	-	92	G/R	0,372	0,048947	-	62	2,3
MYBPC3	rs730880619	ms	Q14896	165	E/D	0,707	0,020949	-	18	1,9
MYCBP2	rs1315072581	ms	O75592	56	P/H	-0,607	0,000951	0,000	47	0,4
MYH13	17:10320355:T/C	ms	Q9UKX3	1085	K/E	0,779	0,024411	0,176	20	N/A
MYH15	rs199682590	ms	Q9Y2K3	1910	N/S	0,501	0,020825	0,970	48	9,1
MYH2	rs1567727764	stop	Q9UKX2	1551	E/*	0,560	0,018655	1,000	30	0,7
MYH2	rs201040489	ms	-	445	R/H	-0,406	0,019307	0,985	48	18
MYH3	rs143973840	ms	P11055	56	Q/E	-0,369	0,022519	0,000	54	55
MYH9	rs727503281	ms	P35579	1936	R/W	0,534	0,015971	0,831	24	4,2
MYO15A	rs145292219	ms	Q9UKN7	462	G/D	0,384	0,006460	0,934	104	310
MYO15B	rs769135244	ms	Q96JP2	673	R/C	-0,450	0,011251	0,007	62	0
MYO18A	rs748958172	ms	Q92614	785	M/V	-0,393	0,049062	0,000	50	0,1
MYO1F	rs557720157	ms	O00160	658	R/W	-1,000	0,009984	0,846	12	0,4
MYO1G	rs751701889	ms	B0I1T2	566	I/M	-0,385	0,033327	0,933	44	0
MYO1H	rs1566045601	stop	Q8N1T3	907	K/*	-0,583	0,030419	1,000	31	0,1
MYO5B	rs750051198	ms	Q9ULV0	125	Y/C	-0,598	0,050211	0,124	17	0,4
MYO7B	rs547914012	ms	Q6PIF6	394	R/Q	-0,668	0,010504	0,027	39	0,8
MYO7B	rs143550775	ms	-	1918	R/Q	0,556	0,030326	0,984	29	137
MYO9B	rs1568284597	ms	Q13459	682	V/M	0,462	0,022236	0,485	23	0,7
MYOM1	rs866006864	ms	P52179	229	A/V	-1,019	0,007773	0,000	12	6,8
MYRFL	rs765678851	ms	Q96LU7	716	R/Q	-0,621	0,020464	0,000	37	2,6
N4BP2	rs769146729	ms	Q86UW6	739	L/F	-0,714	0,023186	0,000	16	0,2
NAA25	rs746012353	ms	Q14CX7	74	L/R	-0,725	0,009316	0,835	16	6,0
NAA25	rs12231744	ms	-	876	K/R	-0,574	0,039656	0,001	38	7071
NAA35	rs1564301637	ms	Q5VZE5	219	R/G	-1,708	0,001180	0,150	10	0,1
NAALADL1	rs147209948	ms	Q9UQQ1	211	A/V	-0,949	0,025595	0,006	16	220
NABP1	rs764450900	ms	Q96AH0	198	P/L	-0,442	0,035796	0,999	27	0,8
NACAD	rs552765962	ms	O15069	1241	P/T	-0,416	0,021649	0,010	46	105

NACC2	rs759435250	ms	Q96BF6	541	E/K	0,888	0,000350	0,646	18	7,6
NADSYN1	rs143959747	ms	Q6IA69	60	S/L	-0,431	0,001849	0,653	93	4,5
NAGPA	rs1567142778	ms	Q9UK23	173	R/C	-1,106	0,000437	0,977	19	0,8
NAIF1	rs138010554	ms	Q69YI7	297	R/C	-0,986	0,001908	0,874	17	60
NAPG	rs376629414	ms	Q99747	293	D/G	-0,602	0,045270	0,003	15	9,1
NAV2	rs776348337	ms	Q8IVL1	1647	R/C	0,593	0,039251	-	23	4,5
NBEAL1	rs756929853	ms	Q6ZS30	2124	Y/C	0,536	0,028777	1,000	39	0,1
NBPF14	rs1449518994	ms	Q5TI25	2974	T/R	-0,440	0,039055	0,000	39	2,1
NBPF8	1:120449308:G/C	ms	Q3BBV2	298	Q/H	-0,674	0,041223	0,921	20	N/A
NBPF9	rs1349107435	ms	P0DPF3	714	W/C	0,922	0,003405	0,000	30	12
NBPF9	rs1418431567	ms	-	520	R/W	-0,670	0,019821	0,024	31	1,5
NCAPD3	rs199849895	ms	P42695	1404	E/K	-0,921	0,005266	0,000	18	16
NCF4	rs150976323	ms	Q15080	151	R/C	0,796	0,017743	0,942	14	1,5
NCKAP5	rs370409499	ms	O14513	795	Y/C	0,592	0,033956	0,003	17	4,9
NCMAP	rs113438005	ms	Q5T1S8	73	A/T	0,607	0,043935	0,000	18	6,8
NCOA6	rs113308271	ms	Q14686	163	A/V	0,441	0,041936	0,102	22	3,8
NCOR2	rs745312035	ms	Q9Y618	2483	A/T	-1,147	0,000139	0,036	24	12
NCOR2	rs1396687230	ms	-	1523	A/V	-0,537	0,007622	0,201	31	2,3
NCOR2	rs761145072	ms	-	2423	R/G	1,079	0,003446	0,999	24	0,7
NDRG1	rs145871479	ms	Q92597	11	A/T	0,495	0,038489	0,011	35	175
NDST1	rs138889348	ms	P52848	707	A/V	-0,392	0,019422	0,043	43	2,0
NDUFA8	rs776160822	ms	P51970	35	Q/R	-0,836	0,011373	0,903	17	0,4
NEB	rs750990726	ms	P20929	3295	R/W	-0,628	0,037046	0,988	21	3,4
NEDD4L	rs746580879	ms	Q96PU5	3	R/C	0,822	0,002146	0,203	28	7,6
NEIL1	rs759056574	ms	Q96FI4	373	R/Q	-0,655	0,037206	0,000	12	6,8
NEK3	rs34076988	ms	P51956	461	D/N	0,882	0,003367	0,282	20	23
NELFB	rs1564441238	ms	Q8WX92	139	K/N	0,391	0,049650	0,142	25	0,4
NELL2	rs375958961	ms	Q99435	766	R/C	-0,822	0,037169	0,741	16	12
NEMP1	rs753618647	ms	O14524	379	R/Q	0,690	0,036435	1,000	15	2,3
NEMP1	rs1006583185	ms	-	397	T/M	0,649	0,042793	0,336	23	1,8
NF2	rs900545157	ms	P35240	497	S/T	0,462	0,033501	0,021	30	2,6
NFIC	rs35952068	ms	P08651	265	T/S	0,854	0,002184	0,144	21	63
NFILZ	rs755954670	ms	A0A5F9ZHS7	153	R/Q	1,066	0,012246	0,014	18	0,7
NGEF	rs764647434	ms	Q8N5V2	591	R/C	0,689	0,031945	0,761	16	0,3
NHERF1	rs141613848	ms	O14745	301	D/V	0,568	0,020556	-	28	108
NHERF2	rs376297831	ms	Q15599	274	R/L	-0,479	0,021758	-	44	1,1
NHERF4	rs759297320	ms	Q86UT5	25	L/I	0,632	0,016991	0,374	25	1,1
NID1	rs201024490	ms	P14543	274	V/M	0,849	0,018617	0,910	13	4,2
NID1	rs34606818	ms	-	334	P/L	-0,950	0,004262	0,033	31	4,5
NID2	rs149533842	ms	Q14112	462	T/M	0,369	0,012335	0,000	78	62
NIFK	rs750327736	ms	Q9BYG3	17	Q/R	-0,358	0,034571	0,012	52	0,2
NIPAL1	rs1560326020	ms	Q6NVV3	287	V/I	-0,751	0,025387	0,416	12	0
NKAPD1	rs761966583	ms	Q6ZUT1	129	Q/E	0,970	0,037813	0,935	10	0
NKD1	rs140589227	ms	Q969G9	67	V/M	0,764	0,000447	0,003	38	5,3
NLRC3	rs138457262	ms	Q7RTR2	601	R/H	0,707	0,031914	0,791	16	568
NLRP11	rs1199059111	ms	P59045	113	F/S	-0,949	0,013463	0,025	11	1,3
NLRP12	rs200996095	ms	P59046	341	T/I	0,445	0,017156	1,000	41	15
NME6	3:48295131:T/C	ms	O75414	113	D/G	0,893	0,021765	0,163	10	N/A
NME8	rs754300578	ms	Q8N427	204	I/K	0,648	0,039185	0,003	16	0,4
NME9	rs2052125802	ms	Q86XW9	248	V/A	0,633	0,018887	0,040	12	0
NMI	rs185889672	ms	Q13287	264	G/D	-0,407	0,035144	0,000	32	2,3
NMUR2	rs4958535	ms	Q9GZQ4	298	S/I	-0,502	0,009866	0,011	38	14705
NOL11	rs2291284	ms	Q9H8H0	115	V/A	-0,672	5,69E-05	0,061	75	210
NOS2	rs745804011	ms	P35228	1002	H/R	-0,603	0,021928	0,003	20	0,8
NOS2	rs1567632363	ms	-	1002	H/Q	-0,603	0,021928	0,120	20	0,1
NOTCH1	rs536167222	ms	P46531	2455	T/A	-0,399	0,001527	0,265	104	3,4
NOVA1	14:26448097:T/G	ms	P51513	462	E/D	-1,074	0,011270	0,149	11	N/A
NOVA1	rs777508869	ms	-	384	A/G	0,501	0,025642	0,095	18	0
NPAP1	rs148062269	ms	Q9NZP6	698	N/S	-0,444	0,050974	0,497	41	6,4
NPC2	rs202134174	ms	P61916	157	W/R	-0,628	0,014741	0,000	25	4,2
NPEPPS	rs201842079	ms	P55786	912	R/Q	0,712	0,015034	0,020	18	1,5
NPEPPS	rs571371035	ms	-	297	N/S	-0,560	0,015577	0,040	32	16
NPHP3	rs1559999380	ms	Q7Z494	1231	Q/H	0,509	0,030262	0,988	26	0,4
NPHP4	rs372565083	ms	O75161	482	P/L	0,916	0,008471	0,003	14	14
NPIPA1	rs539477828	ms	Q9UND3	303	D/E	0,647	0,030701	0,930	26	69
NPIPA2	16:14750871:C/T	ms	E9PIF3	20	A/V	0,628	0,018339	0,125	25	N/A
NPIPA3	16:14711747:C/T	ms	F8WFD2	20	A/V	0,663	0,021293	0,000	26	N/A
NPIPB15	rs920554826	ms	A6NHN6	194	R/Q	0,822	0,048740	0,992	16	0
NPIPB2	rs530343762	ms	A6NJ64	280	T/I	0,774	0,010180	0,998	26	0,8
NPIPB5	rs1421452584	ms	A8MRT5	646	M/V	0,559	0,043620	0,000	24	0
NPY4R2	rs782151881	ms	P0DQD5	23	P/L	-0,776	0,009952	0,000	11	0,7
NQO2	rs766428660	ms	P16083	136	G/S	0,610	0,000903	0,998	59	5,4
NRG1	rs1563846753	ms	Q02297	28	S/C	0,581	0,030867	0,996	23	0
NRIP1	rs758399379	ms	P48552	624	T/M	0,449	0,025039	0,793	39	0,4
NSUN6	rs775368041	ms	Q8TEA1	181	R/C	-0,406	0,022017	1,000	46	1,5
NT5C	rs923470976	ms	Q8TCD5	86	E/K	-0,879	0,033730	0,360	11	1,5
NTF4	rs750511722	ms	P34130	110	A/T	-0,972	0,022342	0,995	15	0,4
NTN3	rs775996713	ms	O00634	321	R/H	-0,417	0,008602	0,995	42	2,6
NTN5	rs770743005	ms	Q8WTR8	461	R/Q	0,352	0,003457	0,065	120	1,5
NTSR2	rs1227438548	ms	O95665	120	Y/H	-0,398	0,028385	0,706	50	0,8

NUDT22	rs771324654	ms	Q9BRQ3	126	A/V	0,629	0,028987	0,003	10	0
NUP107	rs139991199	ms	P57740	54	R/Q	-0,492	0,028343	0,009	29	57
NUP107	rs200326452	ms	-	885	R/H	-0,355	0,046728	0,015	43	9,4
NUP205	rs78254699	ms	Q92621	1019	N/S	-0,408	0,004439	0,198	56	79
NUP210	rs138332591	ms	Q8TEM1	1178	R/Q	0,459	0,024832	0,953	49	8,7
NUP214	rs28594669	ms	P35658	1592	G/A	0,432	0,004022	0,278	65	970
NUP35	rs770715723	ms	Q8NFH5	236	I/L	-0,433	0,019623	0,298	32	1,3
NUP62	rs772416904	ms	P37198	74	F/L	0,577	0,014900	0,903	42	2,6
NUP88	rs772267425	fs	Q99567	212	I/X	-0,396	0,013610	1,000	70	0,4
NUP88	17:5405165:A/T	stop	-	312	C/*	0,933	0,037030	1,000	15	N/A
NUTM2A	rs770632205	ms	Q8IVF1	784	S/L	0,370	0,002694	0,042	98	1,1
NUTM2B	rs1181064857	ms	A6NNL0	230	V/I	-0,370	0,027757	0,006	64	9,3
NUTM2E	rs1428647934	ms	B1AL46	230	V/I	-0,447	0,009085	0,010	61	0
NXNL2	rs751445572	stop	Q5VZ03	142	Q/*	1,212	0,004748	1,000	14	0,4
OAS3	rs377672802	ms	Q9Y6K5	443	R/C	-0,974	0,013515	0,001	12	5,3
OAT	rs778350564	ms	P04181	150	C/S	-0,550	0,048573	0,985	27	0,4
OBSCN	rs201385101	ms	Q5VST9	5935	G/D	0,736	0,018230	0,962	14	20
OBSCN	rs375770928	ms	-	570	A/V	-0,436	0,048912	0,161	33	0,4
OBSCN	rs765706284	stop	-	6524	R/*	0,869	0,027590	1,000	12	3,0
OCA2	rs372899234	ms	Q04671	421	R/W	-0,510	0,048989	0,978	18	5,7
OCSTAMP	rs1568897913	stop	Q9BR26	243	W/*	1,173	0,024410	1,000	10	0
OLFML2A	rs200109271	ms	Q68BL7	103	E/K	-0,444	0,028625	0,706	40	17
ONECUT2	rs781095849	ms	O95948	22	P/L	0,526	0,044109	0,007	17	1,1
OPN1LW	rs148583295	ms	P04000	230	I/T	0,510	0,042997	0,000	10	301
OPN1MW	X:154191728:G/A	ms	P04001	207	V/M	-0,479	0,025769	0,982	57	N/A
OPN3	rs138406816	ms	Q9H1Y3	317	R/Q	-0,523	0,039876	0,992	25	65
OPRM1	rs200207721	ms	P35372	67	M/V	-0,361	1,72E-05	0,000	260	0,4
OR10A4	rs1564922932	ms	Q9H209	153	G/E	0,749	0,017180	0,995	15	0
OR10A5	rs780579227	ms	Q9H207	220	T/S	0,386	0,034426	0,037	35	0
OR10C1	rs767356219	ms	Q96KK4	282	P/S	-0,383	0,011076	0,237	62	0
OR10G9	rs768689141	ms	Q8NGN4	15	L/F	-0,402	0,026754	0,005	52	3,0
OR10H4	rs1568263662	ms	Q8NGA5	129	H/Y	-0,838	0,020365	0,014	19	0
OR11H1	rs201166644	ms	Q8NG94	52	V/F	1,324	0,027400	0,195	11	133
OR11H4	rs142720326	ms	Q8NGC9	9	V/M	-0,503	0,016773	0,795	36	114
OR1M1	rs771073707	ms	Q8NGA1	97	C/G	0,703	0,025232	0,986	11	0,8
OR2A5	rs201829543	ms	Q96R48	223	A/G	-1,014	0,010106	0,001	15	150
OR2AG1	rs2659879	ms	Q9H205	299	R/W	0,471	0,013044	0,457	45	24862
OR2D3	rs768917280	ms	Q8NGH3	152	M/T	-0,549	0,041307	0,431	25	0
OR2M3	1:248203600:T/C	ms	Q8NG83	178	F/S	0,696	0,037344	0,932	20	N/A
OR2M5	rs142232947	ms	A3KFT3	80	K/T	-1,026	0,026984	0,885	10	48
OR2T34	rs755824111	ms	Q8NGX1	197	V/I	-0,428	0,031577	0,003	45	0,9
OR2T35	rs768155855	ms	Q8NGX2	14	V/I	0,389	0,021787	0,000	54	8,0
OR2V1	rs768276571	ms	Q8NHB1	255	A/E	-0,815	0,009587	0,982	17	5,3
OR3A1	rs201927505	ms	P47881	131	R/Q	-0,549	0,011163	0,003	39	4,4
OR4K13	rs763176758	ms	Q8NH42	139	R/W	0,427	0,048124	0,115	30	2,5
OR51C1P	rs141670434	ms	A0A3B3IT45	122	R/H	-1,053	0,039579	0,125	12	105
OR51E1	rs149084031	ms	Q8TCB6	73	D/G	0,521	0,022172	0,661	23	11
OR51F1	rs781353738	ms	A6NGY5	174	P/R	0,794	0,044223	0,005	20	0,4
OR52H1	rs761950199	ms	Q8NGJ2	263	A/T	1,162	0,000300	0,179	13	1,1
OR52I1	rs768334451	ms	Q8NGK6	91	S/L	0,358	0,045049	0,099	41	6,8
OR52K1	rs200081615	ms	Q8NGK4	53	A/S	0,698	0,025046	0,001	15	2,0
OR56A4	rs1474585239	ms	Q8NGH8	216	L/F	0,718	0,022558	0,568	21	1,5
OR5AN1	rs777894055	ms	Q8NGI8	39	S/Y	0,423	0,008503	0,006	74	0,2
OR5D14	rs144838984	ms	Q8NGL3	299	V/M	-0,612	0,032912	0,964	25	94
OR5D14	rs201786123	ms	-	291	I/T	0,642	0,007345	0,995	21	8,3
OR5K1	rs759801175	ms	Q8NHB7	194	I/T	0,360	0,034809	0,801	47	0,4
OR5L2	rs143953099	ms	Q8NGL0	254	T/I	1,505	0,012528	0,984	13	49
OR5M3	rs143720540	stop	Q8NGP4	162	Y/*	-0,566	0,031033	1,000	23	1,9
OR5V1	rs151167090	ms	Q9UGF6	121	D/G	0,507	0,006861	0,997	45	10
OR6B1	rs769317140	ms	O95007	218	Y/H	0,577	0,037836	1,000	13	0,1
OR6J1	rs536617937	ms	Q8NGC5	144	V/I	0,573	0,023570	0,018	20	3,8
OR6K3	rs754875892	ms	Q8NGY3	52	D/N	-0,460	0,006309	0,052	65	8,1
OR6K3	rs138237790	ms	Q8NGY3	37	F/C	0,466	0,028677	0,999	25	4,5
OR7D2	rs77836061	ms	Q96RA2	277	M/L	0,398	0,031073	0,062	47	353
OR8B12	rs146463957	ms	Q8NGG6	35	T/M	0,540	0,033026	0,000	30	20
OR8H2	rs146032852	ms	Q8N162	30	M/V	-0,708	0,046489	0,000	12	0,8
OR9Q1	rs148066479	ms	Q8NGQ5	287	L/I	0,642	0,007341	0,031	21	31
ORM2	rs779305528	ms	P19652	104	G/R	0,790	0,012866	0,996	13	0,8
OSBPL10	rs1559483397	ms	Q9BXB5	181	A/V	1,191	0,010324	-	10	0
OSBPL10	rs146089422	ms	-	228	R/Q	0,406	0,001694	-	114	230
OSCP1	rs773468810	stop	Q8WVF1	160	Q/*	0,587	0,051420	1,000	11	0,4
OSGIN1	rs147230915	ms	Q9UJX0	347	Q/R	0,390	0,040093	0,003	47	76
OSGIN2	rs115758475	ms	Q9Y236	449	K/N	0,366	0,037508	0,443	49	107
OTOG	rs755375116	ms	Q6ZRI0	516	R/C	0,467	0,015782	0,956	32	8,1
OTOGL	rs766038825	stop	Q3ZCN5	1635	Q/*	-0,783	0,017680	1,000	13	0,2
OTOP1	rs150117288	ms	Q7RTM1	206	S/L	0,621	0,003479	0,036	39	3,4
OTOP3	rs773800453	ms	Q7RTS5	68	A/T	-0,558	0,012593	0,063	26	0,7
OTUD1	rs1294078058	ms	Q5VV17	109	T/M	-0,544	0,047053	0,928	14	1,8
OXA1L	rs756119586	ms	Q15070	342	L/V	-0,591	0,049629	0,245	15	6,1
OXSR1	rs750858309	ms	O95747	126	I/V	-0,392	0,033042	0,020	38	0

P4HA2	rs371295093	ms	O15460	380	R/W	0,514	0,028003	1,000	40	0,8
PABIR1	rs777582132	ms	Q96E09	49	P/L	0,395	0,026398	0,000	52	0,4
PACS1	rs762496747	ms	Q6VY07	716	G/R	0,857	0,012978	1,000	10	0,8
PAN2	rs185741202	ms	Q9HBH5	492	R/H	0,596	0,015335	0,538	33	0
PAN2	rs147436117	ms	-	208	F/C	0,557	0,045798	0,897	18	41
PAOX	rs1167907763	ms	Q6QHF9	391	M/V	0,887	0,037675	0,621	12	0,4
PAPOLG	rs374304234	ms	Q9BWT3	23	S/T	-0,572	0,048085	0,780	32	3,0
PARD6G	rs1007114977	ms	Q9BYG4	278	G/V	0,591	0,016185	-	33	0,4
PARP10	rs769913263	ms	Q53GL7	216	T/A	-0,640	0,002238	0,532	53	0,3
PARP4	rs4986818	ms	Q9UKK3	792	P/L	-0,762	0,011319	0,996	19	76
PATE1	rs754444895	ms	Q8WXA2	76	M/T	-0,490	0,014732	0,001	43	0,1
PCCB	rs770769499	ms	P05166	34	S/F	-0,582	0,007726	0,176	27	0,2
PCDHA11	rs745813666	ms	Q9Y5I1	353	S/F	0,385	0,005343	-	77	0,7
PCDHA12	rs73263833	ms	Q9UN75	662	T/M	-0,487	0,003067	-	44	590
PCDHB1	rs141650927	ms	Q9Y5F3	768	F/V	0,395	0,029803	-	54	11
PCDHB14	rs199617011	ms	Q9Y5E9	509	D/G	-0,355	0,024234	-	68	11
PCDHB15	rs61745117	ms	Q9Y5E8	508	T/A	0,509	0,042938	-	21	252
PCDHGC4	rs1562119204	ms	Q9Y5F7	747	N/S	1,417	0,002367	0,060	11	0,3
PCGF1	rs1558594802	ms	Q9BSM1	215	P/T	-0,798	0,011231	0,219	20	0
PCGF6	rs571651624	ms	Q9BYE7	17	T/S	-0,549	0,027269	0,000	30	22
PCK1	rs200480357	ms	P35558	436	R/C	-0,396	0,010804	1,000	60	6,4
PCLO	rs1562983254	ms	Q9Y6V0	879	P/H	-0,935	0,027890	0,000	14	0,1
PCLO	rs778633853	ms	-	570	P/R	0,925	0,046309	0,000	11	0,5
PCM1	rs749269864	ms	Q15154	1062	Y/C	-0,907	0,039011	1,000	11	2,6
PCMTD2	rs778503642	ms	Q9NV79	285	R/C	-0,356	0,046388	0,544	42	3,0
PCNT	rs757715830	ms	O95613	899	R/C	0,390	0,016158	0,000	52	0,8
PCNT	rs373849607	ms	-	2142	I/T	-0,452	0,001901	0,158	80	6,8
PCSK5	rs1824271458	ms	Q92824	846	N/S	-0,801	0,008268	0,007	11	0,4
PCSK6	rs372998470	ms	P29122	661	T/I	-0,601	0,044507	0,000	28	138
PDCD4	rs138747298	ms	Q53EL6	129	Q/E	-0,863	0,042689	0,000	11	1,1
PDE10A	rs1430464939	ms	Q9Y233	39	R/Q	-0,699	0,034116	0,000	17	0,4
PDE4DIP	rs1375289217	ms	Q5VU43	2342	Q/R	0,475	0,038551	0,177	20	1828
PDLIM5	rs768674311	ms	Q96HC4	150	F/C	0,846	0,015182	0,993	13	0,4
PDS5B	rs771715623	ms	Q9NTI5	775	L/V	0,630	0,044944	0,113	13	0,8
PDX1	13:27920241:T/G	ms	P52945	35	C/G	-0,606	0,006410	0,994	20	N/A
PECAM1	rs1568032195	ms	P16284	247	M/V	-0,382	0,035283	0,000	47	0
PEG10	rs201604172	ms	Q86TG7	577	V/L	-0,749	0,007122	0,034	18	41
PELO	rs754185927	ms	Q9BRX2	301	K/E	0,604	0,036723	0,015	15	0
PELP1	rs746811925	ms	Q8IZL8	242	R/Q	0,468	0,026459	0,000	32	0,8
PEPD	rs1380412288	fs	P12955	419-420	-/X	0,781	0,033998	1,000	14	1,1
PER2	rs141480763	ms	O15055	240	H/R	0,558	0,006834	0,890	41	3,4
PERCC1	rs545814553	ms	A0A1W2PR82	127	R/Q	0,362	0,049707	0,030	44	81
PEX1	rs748239213	ms	O43933	581	R/W	0,353	0,036555	0,952	49	0,8
PFKP	rs778429333	ms	Q01813	410	N/S	0,568	0,049244	0,006	30	3,8
PGM3	rs781153408	ms	O95394	474	P/A	0,403	0,016322	1,000	58	0,1
PGPEP1	rs367878913	ms	Q9NXJ5	145	G/S	0,566	0,000600	1,000	58	2,3
PHC2	rs147330538	ms	Q8IXK0	532	L/F	0,645	0,050832	0,994	15	60
PHF12	17:28913910:T/C	ms	Q96QT6	421	E/G	-0,814	0,035586	0,462	17	N/A
PHF2	rs146063352	ms	O75151	480	P/L	-0,395	0,000723	0,981	125	242
PHF20	20:35941003:A/G	ms	Q9BVI0	951	D/G	-0,786	0,004681	0,990	22	N/A
PHGDH	rs141408688	ms	O43175	528	A/D	0,769	0,051341	0,778	11	3,1
PHKG2	rs930832189	ms	P15735	322	A/V	-0,637	0,027631	0,017	11	0,4
PHLDB2	rs370795450	ms	Q86SQ0	1054	E/K	-0,427	0,049802	0,163	37	9,4
PHLPP1	rs772545306	ms	O60346	497	L/F	0,467	0,026163	0,996	24	0,4
PHLPP2	rs772778829	ms	Q6ZVD8	841	E/G	-0,462	0,033747	0,998	37	0,7
PHRF1	rs202005033	ms	Q9P1Y6	744	H/R	1,327	0,003328	0,234	19	48
PHYHIPL	10:59245166:T/G	ms	Q96FC7	236	F/V	-0,925	0,028552	0,965	14	N/A
PIGF	rs139098189	ms	Q07326	124	T/A	-0,813	0,039281	0,462	15	162
PIGP	rs2276231	ms	P57054	112	R/S	-1,306	0,029909	0,255	10	2302
PIGQ	rs767700650	ms	Q9BRB3	487	L/P	-0,688	0,012124	0,902	36	0,1
PIGQ	rs750660902	ms	-	589	R/C	-0,620	0,038659	0,118	19	1,7
PIK3C2A	rs61755370	ms	O00443	133	F/L	0,936	0,026456	0,000	12	207
PIK3C2G	rs201664666	ms	O75747	202	E/V	0,367	0,044890	0,286	42	23
PIK3C3	rs777277652	ms	Q8NEB9	394	M/V	0,556	0,008726	0,106	34	0
PIK3CA	rs200404201	ms	P42336	694	A/S	0,537	0,014547	0,053	47	11
PIK3CG	rs542155244	ms	P48736	1095	K/R	-0,428	0,024623	0,948	36	1,5
PIK3R4	rs779308946	ms	Q99570	86	P/L	-0,593	0,019138	0,996	27	0,1
PILRA	rs1563114512	ms	Q9UKJ1	101	W/C	-1,492	0,013181	0,998	15	0
PINLYP	rs193020817	ms	A6NC86	5	T/N	-0,367	0,023278	0,899	61	115
PIP5K1C	rs772615202	ms	O60331	681	P/S	-0,442	0,041192	0,027	34	0
PIWIL4	rs752333176	ms	Q7Z3Z4	272	T/M	0,359	0,048195	-	41	1,1
PJA2	rs187439518	ms	O43164	272	N/D	0,625	0,036766	0,001	37	0,3
PKD1	rs375440448	ms	P98161	1961	A/V	0,458	0,044017	0,140	30	16
PKD1	rs770514810	ms	-	1336	N/K	0,627	0,037144	0,992	12	13
PKD1	rs753792665	ms	-	3670	M/T	-0,942	0,009349	0,104	16	1,5
PKD1	rs763775092	ms	-	2479	F/L	0,810	0,004974	0,691	24	0,4
PKD1L1	rs767709684	ms	Q8TDX9	365	D/A	0,482	0,043967	0,014	31	4,9
PKD2	rs1426011277	ms	Q13563	67	P/S	0,373	0,028957	0,862	47	0,8
PKHD1	rs201812542	stop	P08F94	4048	Q/*	-0,418	0,012452	1,000	63	15
PKHD1L1	8:109508249:G/A	ms	Q86WI1	3794	V/I	0,619	0,048753	0,216	13	N/A

PKM	rs777998040	ms	P14618	162	K/E	-0,879	0,024313	**0,999**	**20**	0,4
PLA2G4E	rs756827449	ms	Q3MJ16	319	R/W	-0,402	0,009013	**1,000**	**61**	0,8
PLCE1	rs776791387	ms	Q9P212	657	L/F	-0,491	0,051383	**0,832**	**26**	0,4
PLCE1	rs752386035	ms	-	614	T/M	0,433	0,001883	**0,898**	111	3,4
PLCG2	rs573799583	ms	P16885	641	H/N	0,822	0,012706	-	**15**	0,7
PLCH1	rs752280073	ms	Q4KWH8	1521	V/M	-0,649	0,026774	0,031	**25**	3,4
PLD4	rs1566891650	ms	Q96BZ4	503	V/L	0,603	0,035020	0,003	**18**	0,4
PLEC	rs200887085	ms	Q15149	746	R/H	0,432	0,019276	0,000	**40**	149
PLEC	rs763436354	ms	-	3530	C/Y	0,371	0,026375	0,000	**76**	1,2
PLEC	rs953750194	ms	-	1835	L/F	0,418	0,035443	**0,998**	**42**	0,4
PLEC	rs781892147	ms	-	681	R/W	0,603	0,037025	0,000	**22**	0,8
PLEKHA4	rs150889080	ms	Q9H4M7	75	R/H	-0,496	0,033019	-	**50**	**22**
PLEKHM1	rs762560450	ms	Q9Y4G2	1027	V/I	0,652	0,006516	**0,927**	**22**	5,3
PLEKHN1	rs779526083	ms	Q494U1	362	R/C	0,791	0,023007	0,003	**13**	6,4
PLG	rs149145958	ms	P00747	200	T/A	-0,663	0,005447	**0,959**	**33**	**66**
PLIN5	rs758198765	ms	Q00G26	463	F/L	0,399	0,045737	**0,938**	**38**	0,4
PLPP6	rs72695803	ms	Q8IY26	293	S/R	-0,682	0,035006	0,036	**12**	**83**
PLXNA1	rs576960383	ms	Q9UIW2	2	P/L	-0,596	0,039574	0,003	**24**	**58**
PLXNA1	rs147504334	ms	-	1635	S/G	**1,029**	0,048631	0,373	**12**	127
PLXNA1	rs370434411	ms	-	443	R/Q	**1,060**	0,002239	0,009	**18**	8,3
PLXNA2	rs770760535	ms	O75051	1728	S/N	-0,628	0,010733	-	**24**	1,1
PLXNA2	rs368547025	ms	-	1474	I/T	-0,979	0,008718	**0,999**	**19**	3,8
PLXNA3	rs369435958	ms	P51805	519	R/H	0,421	0,047746	0,049	**30**	0
PLXNA3	rs370403729	ms	-	287	V/M	-0,354	0,021806	0,202	**70**	**17**
PLXND1	rs1445315536	ms	Q9Y4D7	897	E/K	0,710	0,018356	0,376	**15**	0,4
PM20D1	rs148054590	ms	Q6GTS8	78	E/D	-0,496	0,002180	0,017	**57**	**46**
PMAIP1	rs772510569	ms	Q13794	40	Q/K	-0,797	0,021814	**0,737**	**15**	0,4
PMPCA	rs746125261	ms	Q10713	487	R/K	-0,869	0,043216	0,001	**27**	3,4
PMPCB	rs761593488	ms	O75439	122	E/A	0,744	0,043541	**0,961**	**19**	0,4
PNKP	rs1353421318	ms	Q96T60	296	N/S	0,711	0,029379	-	**18**	0,4
PNLDC1	rs745361800	ms	Q8NA58	498	R/H	0,458	0,043923	**0,844**	**25**	1,5
PNMA1	rs762946571	ms	Q8ND90	55	F/L	0,366	0,026697	0,356	**51**	1,3
PNPLA6	rs143072391	ms	Q8IY17	1238	Q/E	-0,399	0,019805	0,076	**68**	**16**
PODN	rs754036511	ms	Q7Z5L7	80	V/A	-0,795	0,031269	**0,978**	**13**	2,6
POFUT2	rs184052047	ms	Q9Y2G5	130	G/D	-0,471	0,023682	**0,993**	**44**	0,8
POGK	rs894770585	ms	Q9P215	524	N/H	0,728	0,048584	**0,907**	**15**	1,8
POLA2	rs199754207	ms	Q14181	175	G/S	0,518	0,007575	**0,994**	**43**	**40**
POLD3	rs2031617851	ms	Q15054	49	R/Q	0,675	0,046564	**0,485**	**19**	0,4
POLN	rs201300268	ms	Q7Z5Q5	630	R/S	-0,762	0,010469	**1,000**	**12**	5,7
POLN	rs142462434	ms	-	681	V/M	0,710	0,001735	**0,990**	**29**	**20**
POLN	rs148062138	ms	-	630	R/H	-0,762	0,010469	**1,000**	**12**	**43**
POLR1H	rs17187658	ms	Q9P1U0	14	Q/H	0,465	0,023167	0,384	**55**	**1909**
POLR2H	rs776239606	ms	P52434	98	R/S	0,405	0,036203	-	**49**	0,4
POLR2J2	7:102666957:G/C	ms	Q9GZM3	150	P/A	-0,472	0,007951	**0,550**	**48**	N/A
POLRMT	rs139383492	ms	O00411	870	D/N	-0,538	0,011386	0,350	**31**	**1027**
POM121	rs759615955	ms	Q96HA1	993	S/C	0,630	0,030783	0,000	**19**	0,4
POM121C	rs202010067	ms	A8CG34	764	Q/H	0,389	0,040861	0,003	**38**	267
POM121C	rs201031798	ms	-	766	T/I	0,417	0,010488	0,157	**55**	0,4
POM121L12	rs1156584443	ms	Q8N7R1	172	P/S	-0,653	0,006138	0,162	**19**	0,4
POMGNT1	rs200643988	ms	Q8WZA1	168	A/V	0,413	0,020751	0,120	**33**	0,8
PON1	rs141948033	ms	P27169	19	N/D	0,597	0,022134	0,000	**32**	151
POR	rs779082897	ms	P16435	565	G/S	-0,519	0,029963	**0,997**	**37**	6,4
POSTN	rs771635660	ms	Q15063	569	P/S	0,801	0,013628	0,318	**12**	0,1
POTEF	rs755588157	ms	A5A3E0	883	R/W	**1,136**	0,014870	0,431	**13**	0,4
POTEG	rs201401586	ms	Q6S5H5	389	R/T	0,425	0,050212	**0,984**	**46**	10,0
POU5F1B	rs562626736	ms	Q06416	17	G/C	-0,979	0,011605	**0,959**	**21**	0,4
PPARGC1B	rs930128955	ms	Q86YN6	4	N/S	0,520	0,051483	0,000	**19**	0,4
PPIP5K2	rs559400858	ms	O43314	1083	R/H	0,601	0,004589	0,020	**36**	4,0
PPL	rs200709609	ms	O60437	275	S/G	0,773	0,024901	0,017	**23**	**18**
PPM1D	rs770814597	ms	O15297	321	P/S	-0,585	0,032458	0,221	**31**	1,9
PPM1E	rs200685038	ms	Q8WY54	750	W/L	-0,407	0,033836	0,057	**58**	0,8
PPP1R12B	rs770806770	ms	O60237	18	Q/R	-0,931	0,018316	**0,617**	**20**	6,4
PPP1R13B	rs140395624	ms	Q96KQ4	170	I/V	-0,600	0,014284	0,000	**36**	376
PPP1R15A	rs557806	ms	O75807	251	R/Q	-0,471	0,023359	0,030	**51**	**31678**
PPP1R21	rs1558437735	ms	Q6ZMI0	151	A/V	-0,544	0,027062	0,201	**22**	0,3
PPP1R3A	7:113879662:A/T	ms	Q16821	477	I/N	0,746	0,004033	0,035	25	N/A
PPP1R3C	rs778620061	fs	Q9UQK1	224	P/X	-0,461	0,028877	**1,000**	**34**	0,4
PPP1R9B	rs539885092	ms	Q96SB3	138	P/L	0,862	0,025998	**0,722**	**10**	0,1
PPP6R1	rs547677018	ms	Q9UPN7	763	R/C	-0,470	0,017089	0,000	**46**	**24**
PRAMEF15	rs1394626954	ms	P0DUQ1	442	N/K	-0,565	0,008879	0,000	**49**	0,4
PRAMEF17	rs555191694	ms	Q5VTA0	306	A/T	-0,610	0,035304	0,000	**11**	**21**
PRAMEF17	rs201071276	ms	-	294	P/L	-0,610	0,035305	0,082	**11**	0,4
PRB3	rs369321112	ms	Q04118	274	G/R	0,389	0,010377	0,000	**65**	**48**
PRDM1	rs753053806	ms	O75626	558	G/S	-0,549	0,027141	**0,980**	**19**	0,8
PRDM2	rs776188300	ms	Q13029	1097	M/V	0,451	0,049946	-	**41**	0,4
PRDM5	rs757199736	ms	Q9NQX1	135	Q/R	-0,908	0,032976	0,070	**26**	0
PRKAR2A	rs748291376	ms	P13861	131	Q/R	-0,631	0,019133	**0,768**	**13**	0,2
PRKCA	rs1567761731	ms	P17252	48	T/I	0,798	0,014934	**0,654**	**18**	0,1
PRKCA	rs748202197	ms	-	381	V/M	**1,074**	0,001778	**0,920**	**12**	1,5
PRKDC	rs55924155	ms	P78527	3677	P/S	-0,785	0,023872	0,174	**13**	**55**

PRKDC	rs56080897	ms	-	1337	V/I	0,726	0,027659	0,003	11	92
PRMT9	rs139425779	ms	Q86XK2	594	L/V	0,664	0,002135	0,214	25	24
PROM2	rs147967943	ms	Q8N271	790	F/L	-0,664	0,000308	0,143	51	110
PRORP	rs45626032	ms	O15091	229	A/V	-0,769	0,048835	0,668	13	130
PROSER2	rs200622168	ms	Q86WR7	362	A/T	-0,460	0,030193	0,000	35	48
PRPF3	rs1560126950	ms	O43395	675	S/N	-0,723	0,003864	0,090	21	0
PRPF31	rs367896277	ms	Q8WWY3	408	R/W	0,659	0,043020	1,000	16	0,7
PRPF40B	rs745405961	ms	Q6NWY9	333	Y/C	0,368	0,045906	0,997	38	0,2
PRR12	rs376580810	ms	Q9ULL5	373	A/V	-0,412	0,011867	0,000	44	77
PRR14	rs778010296	ms	Q9BWN1	99	G/R	-0,695	0,021036	0,000	17	3,8
PRR14	rs761866966	ms	-	43	P/S	0,378	0,048100	0,046	43	0
PRR15	rs774986809	ms	Q8IV56	96	R/L	-0,476	0,041195	0,773	36	0,8
PRR29	rs774072605	ms	P0C7W0	109	H/Y	0,815	0,035005	0,999	13	0,4
PRR29	rs1567838278	ms	-	29	L/F	-0,672	0,014169	0,019	20	0
PRR30	rs377666702	ms	Q53SZ7	257	R/P	-0,393	0,050246	0,153	49	0
PRRC2A	rs201074309	ms	P48634	1397	R/W	0,416	0,051172	0,000	32	65
PRRC2B	rs908183601	ms	Q5JSZ5	1106	S/R	1,112	0,018219	0,200	14	1,3
PRRT2	rs1567380661	ms	Q7Z6L0	331	S/F	0,469	0,049994	0,998	32	0
PRRT3	rs199883920	ms	Q5FWE3	150	P/S	0,831	0,015907	0,987	14	46
PRSS33	rs186640650	ms	Q8NF86	278	V/I	0,643	0,031603	0,005	17	83
PRSS54	rs753895790	ms	Q6PEW0	326	G/E	0,758	0,021477	0,000	19	0
PSAPL1	rs201866250	ms	Q6NUJ1	364	R/H	-0,361	0,010182	0,803	74	162
PSD4	rs1558893290	ms	Q8NDX1	637	R/G	1,206	0,014207	0,018	12	0,4
PSMC5	rs1231484066	ms	P62195	43	R/W	-0,591	0,025967	0,692	15	0,4
PSMD1	rs201791497	ms	Q99460	89	N/S	-0,383	0,050862	0,007	33	8,7
PSMD12	rs780349428	ms	O00232	234	D/N	0,774	0,004686	0,024	32	3,4
PSME3IP1	rs746174590	ms	Q9GZU8	146	K/T	-0,809	0,000273	0,964	30	0,4
PSRC1	rs116496512	ms	Q6PGN9	86	A/V	0,963	0,014683	0,986	13	177
PTCD3	rs144366369	ms	Q96EY7	682	D/N	0,845	0,032231	0,000	18	60
PTPN13	rs758235474	ms	Q12923	156	H/L	-0,377	0,042326	0,898	58	0,4
PTPN13	rs772350639	ms	-	1893	L/M	1,182	0,020599	-	22	1,5
PTPN14	rs148337405	ms	Q15678	1068	T/M	0,375	0,009301	0,999	95	23
PTPN3	rs200101992	ms	P26045	436	H/Y	0,508	0,010660	0,000	49	7,6
PTPN5	rs753563589	ms	P54829	45	E/K	0,690	0,027725	0,000	19	1,1
PTPRC	rs140403368	ms	P08575	433	D/N	-0,407	0,037750	0,000	48	90
PTPRD	rs760402372	ms	P23468	1869	V/G	-0,498	0,036245	0,997	34	0
PTPRF	rs887326029	ms	P10586	493	A/V	0,932	0,018146	0,611	13	5,7
PTPRF	rs771277235	ms	-	1336	D/N	0,523	0,045164	0,143	22	0,4
PTPRJ	rs779058301	ms	Q12913	1336	I/N	0,547	0,001375	0,920	64	1,5
PTPRJ	rs183923997	ms	-	959	R/H	0,520	0,009112	0,335	61	27
PTPRO	12:15569445:T/C	ms	Q92729	926	Y/H	0,780	0,034337	0,921	11	N/A
PTPRS	rs4807697	ms	Q13332	1457	C/R	0,698	0,042750	0,000	17	118
PTPRZ1	rs139790820	ms	P23471	187	T/A	0,582	0,044226	0,000	28	60
PTPRZ1	rs1188819495	ms	-	1466	L/F	-0,676	0,031690	0,000	14	0,4
PUS1	rs199863621	ms	Q9Y606	122	R/Q	0,397	0,026247	0,007	36	6,0
PUS7	rs752082590	ms	Q96PZ0	253	S/T	0,533	0,031816	0,333	24	0
PYCR1	rs757343922	ms	P32322	4	G/C	0,419	0,003520	1,000	64	0,4
PYGM	rs115690781	ms	P11217	259	N/S	-0,393	0,042959	0,935	31	6,4
QRICH2	rs759856315	ms	Q9H0J4	277	S/R	0,550	0,008128	0,000	45	0,8
QTRT1	rs139560362	ms	Q9BXR0	117	L/M	0,523	0,017103	0,909	36	160
R3HCC1L	rs145569032	ms	Q7Z5L2	539	I/M	-0,784	0,017497	0,264	17	19
RAB11FIP1	rs375424635	ms	Q6WKZ4	966	A/S	-0,450	0,024418	0,230	28	2,3
RAB11FIP3	rs1352095348	ms	O75154	643	R/W	0,758	0,015808	0,046	13	1,5
RAB11FIP5	rs114626386	ms	Q9BXF6	933	E/K	-1,078	0,000999	0,000	13	745
RAB34	rs1567732776	ms	Q9BZG1	90	E/Q	0,685	0,018760	0,999	11	0,4
RAB37	rs772174577	ms	Q96AX2	133	Q/P	-0,621	0,016129	0,994	19	14
RAB3GAP2	rs377291864	ms	Q9H2M9	991	A/G	-0,525	0,042343	0,000	30	1,6
RAB3GAP2	rs754005017	ms	-	303	Q/R	0,350	0,035170	0,292	49	0,4
RAB3GAP2	rs747310001	ms	-	120	W/C	-0,672	0,020130	0,977	16	0,1
RAB3IL1	rs1281765508	stop	Q8TBN0	249	R/*	-0,461	0,048476	1,000	21	0
RABEP1	rs761934519	ms	Q15276	862	T/S	0,706	0,009912	0,958	22	2,2
RABEP1	rs368832529	ms	-	793	V/I	-0,364	0,037696	0,014	52	4,9
RABGEF1	rs1562896004	ms	Q9UJ41	447	R/T	0,793	0,016162	0,019	10	0,2
RABGEF1	rs373315327	ms	-	96	R/C	0,409	0,042234	0,676	34	0,4
RABL6	rs761584878	ms	Q3YEC7	595	P/S	0,374	0,041172	0,647	62	0,9
RAD51C	rs756727559	ms	O43502	183	I/T	0,433	0,041228	0,600	38	1,9
RAD51C	rs199886026	ms	-	244	I/V	0,425	0,000102	0,127	152	3,8
RAD54L2	rs746877630	ms	Q9Y4B4	534	I/T	0,510	0,021923	0,734	24	0,3
RALGAPA1	rs376013955	ms	Q6GYQ0	405	R/T	-0,603	0,024713	0,428	33	2,6
RALGAPA2	rs778008706	ms	Q2PPJ7	1050	E/K	-0,428	0,014171	0,000	54	1,1
RALGDS	rs764488895	ms	Q12967	262	S/L	0,728	0,007455	0,316	24	2,6
RANBP6	rs1842548384	ms	O60518	202	A/P	0,832	0,003456	0,962	11	0,2
RAP1B	rs372360254	ms	P61224	111	M/V	-0,579	0,037921	0,922	16	2,7
RASA2	rs764501312	ms	Q15283	210	T/I	-0,484	0,021478	0,972	23	0
RASA4B	rs1253896488	ms	C9J798	633	G/S	-0,780	0,047755	0,000	11	0
RASA4B	7:102501161:T/C	ms	-	193	M/V	0,661	0,031486	0,010	29	N/A
RBM12B	rs200294553	ms	Q8IXT5	685	R/Q	0,834	0,023712	0,030	25	16
RBM12B	rs202226883	ms	-	498	R/C	-0,520	0,019329	0,000	21	128
RBM19	rs763546088	ms	Q9Y4C8	125	E/V	0,675	0,008847	0,160	23	0,2
RBM33	rs371318332	ms	Q96EV2	836	A/P	0,501	0,043719	0,536	24	2,3

RC3H1	rs377392987	ms	Q5TC82	557	I/V	0,445	0,012498	0,000	54	3,0
RCAN1	rs1568938287	ms	P53805	58	E/D	-0,880	0,010558	0,046	14	0
RCN3	rs764847109	ms	Q96D15	57	E/K	-0,948	0,018724	0,958	13	0,8
RD3	rs747677203	ms	Q7Z3Z2	44	Q/K	0,548	0,035771	0,550	26	0,8
RECQL5	rs756694533	ms	O94762	490	G/D	0,746	0,018287	0,766	10	2,7
RELCH	rs755098911	ms	Q9P260	325	A/T	0,727	0,015258	0,079	15	0,4
RELCH	rs746069802	ms	-	700	H/Y	0,562	0,005905	0,073	52	0,4
REST	rs1560451173	ms	Q13127	342	S/A	0,761	0,020945	0,993	10	0
RETSAT	rs139043592	ms	Q6NUM9	218	V/M	-0,361	0,012008	0,020	78	1145
REXO1	rs544520974	ms	Q8N1G1	566	P/L	0,488	0,020417	0,000	50	15
RFX5	rs149605765	ms	P48382	284	L/P	0,700	0,012148	0,000	26	19
RFXAP	rs1483760429	ms	O00287	19	P/S	-0,714	0,039846	0,810	12	3,4
RGPD1	rs1266171383	ms	P0DJD0	977	Q/P	-0,489	0,042248	0,548	22	0
RGPD2	2:87784094:T/G	ms	P0DJD1	977	Q/P	-0,489	0,042290	0,801	23	N/A
RGPD5	2:109803997:C/T	ms	Q99666	91	P/S	-0,649	0,033501	0,880	20	N/A
RGPD6	2:110540064:G/A	ms	-	865	P/S	0,550	0,047134	0,022	24	N/A
RGPD8	rs200798390	ms	O14715	1128	A/T	-0,431	0,014526	-	60	94
RGPD8	rs1369419755	ms	-	91	P/S	-0,431	0,014526	0,961	60	105
RGR	rs146536539	ms	Q8IZJ4	77	L/F	-0,496	0,004957	0,036	64	44
RGS1	rs150745219	ms	Q08116	156	R/Q	0,400	0,002236	-	92	16
RGS16	rs753151197	ms	O15492	22	R/H	-0,833	0,006492	0,506	21	4,2
RGS3	rs137990160	ms	P49796	875	G/S	-0,514	0,008371	0,015	39	42
RHBDF1	rs368764349	ms	Q96CC6	234	R/C	-0,435	0,045636	0,023	41	7,9
RHOBTB2	rs146491810	ms	Q9BYZ6	245	V/A	0,486	0,011830	0,499	36	132
RHPN1	rs371033148	ms	Q8TCX5	347	K/N	-0,935	0,042249	0,999	13	13
RIC8B	rs762850738	ms	Q9NVN3	479	K/R	0,688	0,047682	0,848	13	4,9
RILP	rs34982553	ms	Q96MT3	281	R/Q	-1,114	0,041984	0,968	11	897
RIMBP3C	rs1555881974	ms	A6NJZ7	667	G/R	-0,392	0,049856	0,022	57	0
RIOK1	rs547473770	ms	Q9BRS2	220	R/W	0,772	0,047832	1,000	14	0,8
RLBP1	rs760538477	ms	P12271	60	E/K	0,433	0,015995	0,039	38	0,4
RMDN2	rs142128542	ms	Q96LZ7	226	D/N	-1,132	0,015245	0,020	12	18
RND1	rs148733686	ms	Q92730	145	A/V	-0,475	0,019541	0,001	44	19
RND2	rs748749886	ms	P52198	127	L/R	-0,712	0,011781	0,962	18	0
RNF115	rs774535104	ms	Q9Y4L5	276	D/E	0,640	0,027036	0,084	29	0,4
RNF123	rs761494332	ms	Q5XPI4	38	Y/C	-0,429	0,006319	0,985	62	0,4
RNF123	rs2080162391	ms	-	846	L/P	-0,538	0,036043	0,382	25	5,1
RNF145	rs545563752	ms	Q96MT1	536	I/T	1,006	0,045136	0,307	10	0,2
RNF169	rs759737139	ms	Q8NCN4	119	R/H	-2,509	0,000674	0,837	12	75
RNF17	rs776307815	ms	Q9BXT8	1182	V/I	-0,468	0,009183	0,007	45	1,1
RNF213	rs1568136656	ms	Q63HN8	3911	A/D	-1,633	0,000401	0,006	11	0
RNF39	rs139005614	ms	Q9H2S5	305	R/G	0,474	0,018086	0,132	40	393
RNF4	rs776582971	ms	P78317	138	G/R	-0,675	0,003795	0,019	18	0,8
RNF44	rs201402074	ms	Q7L0R7	199	P/S	-0,798	0,042629	0,034	15	23
RNH1	rs150334174	ms	O60930	317	S/L	0,698	0,035384	0,210	15	34
ROBO4	rs776645625	ms	Q8WZ75	435	V/I	-0,516	0,036068	0,033	17	0,4
ROGDI	rs1567600743	ms	Q9GZN7	139	G/D	0,801	0,042088	0,712	13	0
ROGDI	rs773589345	ms	-	187	D/H	1,212	0,023385	0,984	12	1,9
RP1	rs758050819	ms	P49842	15	T/M	-0,467	0,000847	0,462	104	0,4
RP1L1	rs200588941	ms	Q8IWN7	2204	E/A	0,861	0,008967	0,000	12	25
RPF1	rs145776320	ms	Q9H9Y2	4	A/V	0,403	0,032791	0,000	38	373
RPGRIP1L	rs376935464	ms	Q68CZ1	1195	S/R	-0,393	0,020723	-	62	1,1
RPL6	rs754262209	ms	Q02878	248	I/V	0,679	0,024131	0,000	14	0,7
RPP25	rs192149396	ms	Q9BUL9	156	P/T	-0,489	0,041293	0,001	22	1,1
RPRD2	rs375366241	ms	Q5VT52	780	R/Q	0,956	0,024926	0,000	10	8,7
RPRD2	rs373089873	ms	-	1176	E/Q	-0,603	0,044977	0,539	18	0,4
RRBP1	rs1021672375	ms	Q9P2E9	1162	A/T	0,532	0,046064	0,021	23	3,0
RREB1	rs140206154	ms	Q92766	315	R/C	-0,562	0,026290	0,997	37	38
RRN3	rs774512208	ms	Q9NYV6	111	S/N	0,514	0,037743	0,003	27	0,4
RRP12	rs776080417	ms	Q5JTH9	646	T/M	0,545	0,042882	0,883	18	1,5
RTEL1	rs1568726633	ms	Q9NZ71	1215	S/F	-0,428	0,032638	0,377	34	0,4
RTN4RL1	rs201050462	ms	Q86UN2	67	G/S	0,358	0,043787	0,003	59	86
RUFY3	rs764540621	ms	Q7L099	601	E/K	0,656	0,008922	0,120	29	0,2
RUNDC3B	rs752955187	ms	Q96NL0	26	A/V	0,641	0,020537	0,195	19	0,4
RXRA	rs779808577	ms	P19793	215	R/W	0,691	0,047013	0,549	18	0,8
RYR1	rs138874610	ms	P21817	708	D/N	-0,489	0,008015	1,000	67	59
S100PBP	rs757895019	ms	Q96BU1	177	L/F	-0,809	0,001500	0,240	25	5,7
S1PR4	rs779911002	ms	O95977	128	A/T	-0,649	0,038833	0,719	14	4,0
SAFB	rs749699724	ms	Q15424	886	R/W	-0,780	0,034375	0,000	10	5,1
SAMD1	rs370432894	ms	Q6SPF0	291	P/L	0,488	0,021868	0,000	38	1,3
SAMD11	rs1557610691	stop	Q96NU1	625	E/*	0,683	0,038339	1,000	14	0,1
SAMD14	rs369309802	ms	Q8IZD0	402	R/Q	-0,679	0,020367	0,000	21	5,3
SANBR	rs746911974	ms	Q6NSI8	269	N/K	-1,057	0,006760	0,834	12	0
SARDH	rs200897249	ms	Q9UL12	642	D/E	0,449	0,032484	0,013	53	15
SART1	rs773035344	ms	O43290	86	A/G	0,353	0,032103	0,000	67	15
SBF2	rs775360425	ms	Q86WG5	1578	K/R	1,109	0,033647	0,005	12	1,5
SBNO2	rs200703494	ms	Q9Y2G9	142	P/S	-1,299	0,026765	0,005	18	7,2
SCAF1	rs763412878	ms	O14548	329	P/A	-0,445	0,027164	0,000	38	0
SCAF8	rs147069918	ms	Q9UPN6	56	A/V	-0,518	0,003842	0,000	61	295
SCAMP4	rs201067147	ms	Q969E2	181	G/R	-1,149	0,013593	0,837	11	1,5
SCAMP4	rs369144370	ms	-	136	S/L	0,702	0,015522	0,161	20	20

SCARF1	rs4790250	ms	Q14162	667	G/S	0,964	0,005359	0,010	**22**	**8191**
SCARF1	rs3760460	ms	-	748	G/V	-0,964	0,005360	**0,919**	**21**	**4493**
SCG3	rs748925597	ms	Q8WXD2	323	E/G	0,670	0,041935	**0,987**	**17**	0,1
SCIMP	rs554330535	ms	Q6UWF3	109	S/P	-0,410	0,004949	**0,514**	**87**	**63**
SCLY	rs762082693	ms	Q96I15	367	R/Q	0,394	0,035662	0,000	**41**	2,0
SCML4	rs142985964	ms	Q8N228	158	G/D	0,420	0,018696	**0,987**	**52**	170
SCN3A	rs202004044	ms	Q9NY46	563	R/C	-0,530	0,042408	**0,953**	**18**	**15**
SCN4A	rs202106192	ms	P35499	1564	V/I	0,410	0,000173	0,009	129	209
SCN4A	rs1567828204	ms	-	260	C/Y	-0,858	0,026601	**0,995**	**16**	0
SCN4A	rs531694454	ms	-	1765	A/T	-0,599	0,020264	-	**18**	3,8
SCN4A	rs78592515	ms	-	18	R/S	0,420	2,09E-06	0,091	223	174
SCN4A	rs754847636	ms	-	148	M/I	-0,469	2,17E-06	0,254	167	0
SCN4A	rs201148948	ms	-	875	P/S	-0,959	0,000647	0,100	**27**	190
SCNN1D	rs141251506	ms	P51172	739	A/G	-0,501	0,036542	**0,860**	**32**	204
SCP2D1	rs376844337	ms	Q9UJQ7	111	P/L	-0,592	0,009206	**0,646**	**26**	8,3
SCRN1	rs139915877	ms	Q12765	62	V/I	0,615	0,001822	**0,743**	**34**	0,4
SDK1	rs370417123	ms	Q7Z5N4	233	I/L	0,493	0,009438	**0,982**	**48**	**15**
SDK2	rs749556489	ms	Q58EX2	885	P/L	-0,356	0,044763	-	**55**	0,1
SDK2	rs761561326	ms	-	1689	N/S	-0,406	0,049264	-	**33**	7,6
SEC14L6	rs751086784	ms	B5MCN3	347	V/L	0,420	0,020258	0,069	**40**	**12**
SEC16A	rs375839951	ms	O15027	1027	P/L	-0,529	0,024748	0,258	**39**	6,9
SEC16A	rs201222771	ms	-	346	R/H	0,858	0,014483	0,000	**12**	**94**
SEC23B	rs121918223	ms	Q15437	530	R/W	0,439	0,011174	**1,000**	**50**	0,6
SECISBP2L	rs1566849658	ms	Q93073	1077	G/E	-0,662	0,044472	0,000	**10**	0,2
SEL1L2	rs200767636	ms	Q5TEA6	124	Q/H	-0,772	0,049975	**0,997**	**11**	**35**
SELE	rs138813218	ms	P16581	136	A/S	0,707	0,000913	**0,999**	**49**	0
SELENOF	rs189422667	ms	O60613	38	S/L	0,551	0,029690	0,242	**22**	**87**
SELENON	rs982364753	ms	Q9NZV5	2	G/C	-0,547	0,050071	0,000	**11**	**27**
SEMA3F	rs757110934	ms	Q13275	628	P/A	**-1,202**	0,016683	0,015	**11**	0,7
SEMA3F	rs768027884	ms	-	724	T/M	0,564	0,017273	**0,738**	**26**	3,0
SEMA3G	rs758775695	ms	Q9NS98	728	R/S	0,446	0,017453	**0,904**	**63**	8,1
SEMA4C	rs145704940	ms	Q9C0C4	665	V/M	0,500	0,040567	**0,666**	**13**	163
SEMA4D	rs765159211	ms	Q92854	5	T/I	-0,581	0,019263	0,003	**32**	0
SENP7	rs758250523	ms	Q9BQF6	756	T/A	-0,480	0,046683	0,015	**21**	0,2
SEPTIN3	rs761632941	ms	Q9UH03	3	H/Y	-0,826	0,012211	0,000	**14**	**35**
SERINC4	rs554825719	ms	A6NH21	204	A/T	0,948	0,006917	**0,999**	**17**	1,0
SERPINA10	rs142013081	ms	Q9UK55	386	T/A	0,470	0,042241	0,000	**35**	5,3
SETD1A	rs746152746	ms	O15047	31	A/S	-0,680	0,005272	**0,862**	**43**	0,4
SETD1B	rs751321721	ms	Q9UPS6	353	P/L	0,450	0,030122	**0,742**	**32**	**65**
SETD2	rs2107638067	ms	Q9BYW2	1982	D/N	0,739	0,023289	**0,996**	**16**	0
SFI1	rs756654178	ms	A8K8P3	877	A/V	0,498	0,037112	**0,994**	**28**	**11**
SFPQ	rs1283676090	ms	P23246	640	I/V	0,539	0,033185	0,146	**17**	0,2
SFXN1	rs769192280	ms	Q9H9B4	301	E/K	-0,377	0,030243	**0,701**	**49**	1,3
SFXN5	rs764726890	ms	Q8TD22	268	P/L	-0,955	0,017489	**0,968**	**12**	8,3
***SG**CA*	rs765429436	ms	Q16586	351	R/H	0,474	0,022045	**0,999**	**45**	1,9
SGCE	rs142744182	ms	O43556	46	I/T	0,881	0,011272	0,000	**16**	214
SGCG	rs774245371	ms	Q13326	115	A/V	0,578	0,030930	**0,984**	**25**	1,1
SGCZ	rs748048633	ms	Q96LD1	289	G/D	-0,413	0,021895	**0,999**	**35**	0,4
SGO1	rs770048758	ms	Q5FBB7	379	D/N	-0,892	0,010254	**0,715**	**13**	1,9
SGSM1	rs772084972	ms	Q2NKQ1	303	D/N	-0,533	0,009606	**0,913**	**27**	0
SH2D3C	rs1564413099	ms	Q8N5H7	417	P/R	0,827	0,037400	**0,999**	**13**	0,1
SH2D3C	rs61761896	ms	-	443	A/S	**-1,026**	0,000266	0,003	**24**	**29**
SH2D5	rs761666159	ms	Q6ZV89	416	E/K	-0,441	0,013324	**0,955**	**66**	2,3
SH2D7	rs145893682	ms	A6NKC9	46	G/S	0,640	0,017325	**1,000**	**22**	417
SH3BP4	rs73995756	ms	Q9P0V3	471	N/S	0,486	0,034397	0,000	**38**	364
SH3D21	rs761828672	ms	A4FU49	171	D/N	-0,770	0,014400	0,053	**10**	0,2
SH3PXD2A	rs199610107	ms	Q5TCZ1	674	E/G	0,594	0,040126	0,222	**26**	**54**
SH3RF3	rs199936909	ms	Q8TEJ3	533	P/L	-0,723	0,037707	0,003	**13**	110
SH3TC1	rs778072816	ms	Q8TE82	392	S/F	0,482	0,019533	**0,950**	**36**	0
SHANK3	rs1013974683	ms	Q9BYB0	751	E/D	0,356	0,022084	-	**89**	0
SHARPIN	rs1428652617	ms	Q9H0F6	49	D/N	0,385	0,034548	**0,834**	**40**	0
SHPK	rs144071313	stop	Q9UHJ6	119	R/*	-0,467	0,025112	**1,000**	**37**	**70**
SHROOM1	rs751394319	ms	Q2M3G4	757	E/Q	0,663	0,017393	**0,879**	**15**	0,1
SI	rs200451408	stop	P14410	1124	R/*	0,432	0,005961	**1,000**	**77**	**11**
SIK2	rs748313173	ms	Q9H0K1	397	E/G	0,798	0,018517	0,000	**12**	0
SIK2	rs371556602	ms	-	302	R/Q	-0,743	0,013687	0,191	**18**	0,6
SIPA1L3	19:38164839:T/C	ms	O60292	1381	Y/H	-0,878	0,002525	0,015	**16**	N/A
SIRPB1	rs141668681	ms	Q5TFQ8	180	T/I	0,871	0,026947	-	**10**	**60**
SIRPB2	rs773180408	ms	Q5JXA9	144	G/D	-0,410	0,048342	**0,999**	**36**	0
SKIDA1	rs200920908	ms	Q1XH10	789	R/Q	-0,537	0,024759	**0,968**	**49**	**63**
SLA	rs372398209	ms	Q13239	202	R/K	**-1,056**	0,006720	0,000	**16**	2,6
***SLC**10A7*	rs779082973	ms	Q0GE19	86	A/T	-0,485	0,013547	0,000	**30**	0,4
SLC11A2	rs17222449	ms	P49281	290	R/Q	-0,626	0,021349	0,031	**24**	1,5
SLC12A1	rs6493311	stop	Q13621	538	Y/*	**-1,023**	0,051200	**1,000**	**11**	**39432**
SLC17A2	rs774991867	ms	O00624	467	Q/P	0,531	0,007756	**0,996**	**44**	1,9
SLC18A1	rs375952046	ms	P54219	157	V/M	0,408	0,006716	**1,000**	**67**	4,1
SLC1A5	rs747541617	ms	Q15758	135	F/L	-0,561	0,028837	**0,999**	**37**	0
SLC22A2	rs576766802	ms	O15244	136	V/I	0,511	0,029864	**0,999**	**18**	2,3
SLC22A23	rs199852691	ms	A1A5C7	122	D/N	0,835	0,001949	0,356	**14**	**38**
SLC24A4	rs199781252	ms	Q8NFF2	562	V/M	0,978	8,23E-05	**0,929**	**26**	0,8

SLC25A23	rs748793177	ms	Q9BV35	74	Y/S	0,395	0,021268	**0,997**	**47**	0,7
SLC25A39	rs763200621	ms	Q9BZJ4	303	T/A	0,412	0,046614	**0,997**	**43**	0
SLC25A47	rs757658344	ms	Q6Q0C1	73	T/N	-0,811	0,039106	**0,703**	**15**	0,1
SLC27A2	15:50182606:T/G	ms	O14975	60	L/R	0,592	0,013739	0,020	**35**	N/A
SLC27A6	rs199924682	ms	Q9Y2P4	148	I/N	-0,386	0,037309	**0,730**	**73**	**22**
SLC2A1	rs144389023	ms	P11166	417	I/T	0,730	0,032569	0,093	**11**	6,0
SLC2A10	rs763889166	ms	O95528	194	T/I	-0,496	0,049581	0,039	**17**	3,0
SLC2A9	rs149454410	ms	Q9NRM0	243	V/I	0,423	0,011714	0,005	**61**	**37**
SLC30A5	rs752773548	ms	Q8TAD4	248	L/F	0,400	0,019159	0,015	**61**	0,4
SLC30A8	rs770454987	ms	Q8IWU4	327	S/T	-0,580	0,030304	0,027	**18**	4,5
SLC33A1	rs144015992	ms	O00400	484	N/T	0,360	0,022997	0,012	**95**	193
SLC34A2	rs769110830	ms	O95436	423	G/R	-0,846	0,021499	**0,999**	**15**	1,5
SLC34A3	rs202109348	ms	Q8N130	307	L/M	**1,617**	0,002139	0,223	**13**	**54**
SLC35A4	rs1244740037	ms	Q96G79	240	G/S	-0,507	0,051945	**0,997**	**20**	1,1
SLC35B4	rs1563214104	ms	Q969S0	246	N/S	0,682	0,002492	**0,739**	**25**	0,7
SLC39A4	rs782236196	ms	Q6P5W5	20	A/V	0,824	0,016831	0,000	**17**	4,2
SLC3A1	rs745473969	ms	Q07837	366	Q/H	-0,979	0,034114	0,028	**12**	1,9
SLC44A1	rs148518626	ms	Q8WWI5	502	T/A	-0,470	0,036172	0,042	**35**	**72**
SLC45A3	rs765451038	ms	Q96JT2	247	R/H	-0,791	0,032130	0,342	**17**	6,0
SLC46A3	rs200182693	stop	Q7Z3Q1	244	R/*	0,393	0,041880	**1,000**	**49**	**62**
SLC50A1	rs755400797	ms	Q9BRV3	35	M/T	-0,506	0,034739	0,003	**18**	0
SLC6A12	rs143648821	ms	P48065	203	I/V	0,373	0,007983	**0,669**	**56**	**28**
SLC6A17	rs775258138	ms	Q9H1V8	483	T/M	**-1,089**	0,027689	**0,999**	**15**	0,4
SLC6A20	rs141811843	ms	Q9NP91	549	K/R	-0,389	0,041285	0,010	**36**	**25**
SLC6A8	rs782208622	ms	P48029	274	V/M	**1,340**	0,026390	**0,915**	**18**	6,4
SLC7A11	rs762190755	ms	Q9UPY5	119	G/C	-0,770	0,010230	**0,999**	**26**	3,0
SLC7A2	rs370916645	ms	P52569	628	A/T	-0,478	0,008467	0,000	**45**	3,2
SLC8A3	rs144289733	ms	P57103	789	V/M	0,427	0,015218	-	**54**	**62**
SLC9A9	rs191690859	ms	Q8IVB4	254	I/V	0,595	0,039837	0,056	**16**	1,1
SLCO4A1	rs1244058546	ms	Q96BD0	2	P/S	0,539	0,044974	**0,973**	**17**	0,4
SLF2	rs748706051	ms	Q8IX21	133	R/W	-0,582	0,047264	**0,529**	**12**	0,8
SLFN12L	rs1567648042	ms	Q6IEE8	123	N/T	-0,578	0,048330	0,060	**22**	0
SLTM	rs142696944	ms	Q9NWH9	314	G/V	-0,557	0,037421	0,000	**12**	**25**
SLX4	rs114472821	ms	Q8IY92	975	P/L	0,686	0,048410	0,000	**14**	704
SLX4	rs115694169	ms	-	385	P/T	0,707	0,031935	0,007	**16**	987
SLX4	rs114014006	ms	-	942	E/Q	0,686	0,048406	0,127	**15**	**1876**
SLX9	rs140102661	ms	Q9NSI2	180	R/W	0,712	0,006237	**0,868**	**34**	6,4
SMARCC1	rs764145318	ms	Q92922	744	A/G	**-1,039**	0,004475	**0,660**	**18**	**11**
SMARCD2	rs1567759892	ms	Q92925	493	R/T	**1,309**	0,009592	**0,990**	**12**	0
SMARCD2	rs201723860	ms	-	201	T/M	0,478	6,99E-08	**0,985**	242	**15**
SMC4	rs1419891152	ms	Q9NTJ3	432	P/S	-0,911	0,030250	**0,900**	**14**	0,4
SMCO1	rs11926701	ms	Q147U7	64	R/W	0,766	0,011016	0,000	**14**	**10486**
SMCO1	rs1427577811	ms	-	214	N/K	-0,829	0,002956	0,042	**17**	0,4
SMDT1	rs776628036	ms	Q9H4I9	5	A/V	0,662	0,046862	0,003	**18**	**12**
SMG7	rs1239743198	ms	Q92540	698	P/L	**-1,008**	0,003779	0,076	**17**	0,1
SMURF2	rs777947628	ms	Q9HAU4	90	G/D	-0,755	0,003436	-	**32**	0
SMYD2	rs766703409	ms	Q9NRG4	348	G/S	-0,546	0,049639	**0,987**	**30**	0,4
SNAI3	rs369333608	ms	Q3KNW1	123	R/W	-0,750	0,041636	0,228	**15**	3,4
SNAPC4	rs201740904	ms	Q5SXM2	1293	R/C	-0,552	0,016342	0,000	**26**	**19**
SNAPC5	rs151153879	ms	O75971	12	E/K	-0,656	0,051031	0,000	**13**	**85**
SNAPC5	rs143176301	ms	-	12	E/V	-0,656	0,051031	0,000	**13**	**85**
SNRNP35	rs142113918	ms	Q16560	216	P/L	**-1,024**	0,015954	0,000	**18**	**28**
SNTA1	rs141724500	ms	Q13424	147	T/I	0,392	0,004498	-	**68**	**95**
SNX29	rs150300274	ms	Q8TEQ0	285	T/S	-0,900	0,023700	0,034	**10**	320
SON	rs184100114	ms	P18583	788	T/A	-0,598	0,045359	0,209	**23**	6,4
SORCS3	10:105157142:C/G	ms	Q9UPU3	496	A/G	**-1,301**	0,001605	**0,640**	**14**	N/A
SORT1	rs773074476	ms	Q99523	541	S/I	-0,555	0,007670	0,089	**22**	0,2
SOS1	rs371024396	ms	Q07889	1316	H/D	0,466	0,051443	**0,517**	**22**	7,9
SOX5	rs769073811	ms	P35711	635	V/M	-0,440	0,004650	**0,978**	**73**	0,2
SP2	rs1211672947	ms	Q02086	251	S/N	0,698	0,029854	0,024	**20**	0,4
SPAG11B	rs775076868	ms	Q08648	91	L/S	-0,801	0,021330	0,000	**13**	6,4
SPAG17	rs144147243	ms	Q6Q759	2133	G/D	-0,737	0,010565	**0,995**	**17**	4,2
SPART	rs778812974	ms	Q8N0X7	334	R/W	-0,669	0,042445	-	**24**	2,3
SPATA18	rs778351262	ms	Q8TC71	417	S/R	-0,612	0,051557	**0,514**	**13**	2,3
SPATA2	rs756953070	ms	Q9UM82	222	T/M	-0,550	0,025242	0,357	**20**	1,5
SPATA31A3	rs945622093	ms	Q5VYP0	930	S/R	0,472	0,049638	0,292	**25**	4,9
SPATA31F1	9:34725839:A/C	ms	Q6ZU69	467	D/E	-0,351	0,051933	0,053	**44**	N/A
SPATS2	12:49526133:C/A	ms	Q86XZ4	506	H/N	-0,611	0,050625	0,053	**11**	N/A
SPCS3	rs762898441	ms	P61009	116	P/L	-0,954	0,015476	0,220	**12**	0,4
SPDEF	rs2233637	ms	O95238	13	P/R	0,474	0,042292	0,000	**41**	**7333**
SPDEF	rs2233639	ms	-	57	A/T	0,474	0,042292	0,007	**41**	**7438**
SPDYE21	rs567907678	ms	A0A494C086	191	V/M	0,530	0,048343	**0,860**	**21**	6,8
SPDYE2B	rs1791901438	ms	A6NHP3	246	I/V	**1,159**	0,005486	0,332	**18**	0
SPECC1L	rs202183866	ms	Q69YQ0	914	Q/E	-0,554	0,041995	0,091	**28**	7,4
SPEG	rs373080805	ms	Q15772	2131	F/L	**1,171**	0,012117	0,095	**10**	2,6
SPEG	rs779078554	ms	-	2858	A/V	-0,854	0,014106	**0,494**	**13**	1,5
SPEM1	rs571769033	ms	Q8N4L4	253	R/W	0,870	0,009140	**0,663**	**14**	2,6
SPEM1	rs191212954	ms	-	185	V/I	-0,378	0,027498	0,000	**52**	670
SPEN	rs766851755	ms	Q96T58	2013	A/T	-0,354	0,037955	0,066	**37**	3,0
SPEN	rs1438984165	ms	-	1017	K/E	-0,598	0,041807	**0,934**	**21**	0,8

SPEN	rs773726905	ms	-	2880	V/M	0,372	0,039668	0,013	**48**	0,4
SPG11	rs1216723582	ms	Q96JI7	1155	P/T	0,820	0,018216	**0,998**	**18**	1,1
SPHK1	rs1567822886	ms	Q9NYA1	33	L/V	-0,635	0,004021	0,301	**34**	0
SPMIP9	rs201634174	ms	Q96LM6	49	R/W	0,848	0,031485	0,000	**16**	**24**
SPPL2C	rs1490540043	stop	Q8IUH8	567	R/*	-0,901	0,013577	**1,000**	**14**	1,1
SPPL2C	rs748111980	ms	-	600	S/T	-0,663	0,023520	0,077	**29**	1,0
SPPL2C	rs753862379	ms	-	542	S/L	-0,522	0,001102	0,025	**74**	3,8
SPRED1	rs765603808	ms	Q7Z699	334	R/C	0,513	0,015005	-	**37**	2,3
SPRR2D	rs1846857	ms	P22532	20	T/A	**1,148**	0,036101	0,000	**25**	**6190**
SPRR3	rs1557889683	ms	Q9UBC9	23	Q/L	0,873	0,021308	0,000	**15**	0
SPRY4	rs200364529	ms	Q9C004	258	V/M	-0,370	0,005690	0,423	**73**	**28**
SPRYD3	rs776074767	ms	Q8NCJ5	72	R/Q	0,696	0,020823	0,028	**28**	0,4
SPTAN1	rs779759134	ms	Q13813	1027	A/T	0,507	0,028535	-	**27**	1,5
SPTBN2	rs769616053	stop	O15020	2356	R/*	0,524	0,044905	**1,000**	**27**	9,4
SPTBN4	rs753897679	ms	Q9H254	163	R/C	**-1,152**	0,000476	**1,000**	**13**	0,8
SPTBN4	rs748745941	ms	-	818	L/F	0,477	0,008397	0,033	**49**	0,4
SPTBN5	rs756377333	ms	Q9NRC6	372	R/Q	**-1,033**	0,026587	0,018	**16**	1,1
SPTBN5	rs201037092	ms	-	3600	R/W	-0,471	0,023701	0,005	**41**	**11**
SRP68	rs771151407	ms	Q9UHB9	76	R/Q	0,826	0,008951	**0,783**	**13**	0,7
SRP72	rs17524437	ms	O76094	7	G/W	0,464	0,019956	**0,966**	**30**	0
SRRM2	rs139637110	ms	Q9UQ35	2485	S/F	0,834	0,011406	**0,904**	**22**	124
SRRM2	rs1323436648	ms	-	2031	R/K	0,567	0,050105	0,000	**18**	0,4
SSC5D	rs748135422	ms	A1L4H1	867	V/M	-0,367	0,009253	**0,997**	**88**	3,0
SSH3	rs151267958	ms	Q8TE77	642	R/Q	-0,677	0,040438	0,003	**23**	207
SSRP1	rs201685642	ms	Q08945	606	D/H	0,642	0,007345	0,300	**21**	7,9
ST3GAL1	rs762669104	ms	Q11201	232	P/L	-0,627	0,019942	**0,671**	**29**	0,8
ST3GAL4	rs765192401	ms	Q11206	120	C/Y	**-1,186**	0,005391	**1,000**	**11**	0,8
ST6GALNAC5	rs752770582	ms	Q9BVH7	268	P/L	0,415	0,040983	**0,997**	**44**	0,2
ST8SIA6	rs200933958	ms	P61647	315	G/S	0,363	0,039419	**0,994**	**57**	**15**
STAB2	rs141041254	ms	Q8WWQ8	2377	E/K	0,675	0,040900	0,374	**12**	**52**
STAG3	rs1562987401	fs	Q9UJ98	703	L/X	0,482	0,034151	**1,000**	**47**	0
STAT5A	rs759535072	ms	P42229	191	Q/H	-0,587	0,048324	0,417	**14**	0,1
STAU2	rs532770072	ms	Q9NUL3	427	R/L	-0,683	0,018067	0,037	**29**	0,4
STC1	rs146703503	ms	P52823	102	G/R	0,825	0,012403	**1,000**	**14**	**17**
STK10	rs753084325	ms	O94804	918	R/Q	**1,245**	0,006678	**0,860**	**11**	1,9
STK11	rs1169900277	ms	Q15831	573	R/H	**1,068**	0,040474	-	**13**	0,4
STK32A	rs200548298	ms	Q8WU08	109	R/H	-0,490	0,045418	**0,999**	**23**	**24**
STOX1	rs372305853	ms	Q6ZVD7	639	H/R	-0,694	0,043510	0,000	**12**	9,4
STOX2	rs1734108048	ms	Q9P2F5	591	C/R	-0,881	0,023839	**0,963**	**14**	0,4
STPG1	rs560627804	ms	Q5TH74	305	G/V	0,600	0,000916	0,239	**44**	9,4
STPG2	rs1479947625	stop	Q8N412	13	E/*	0,929	0,029064	**1,000**	**10**	0,2
STPG3	rs752473487	ms	Q8N7X2	238	L/V	0,608	0,001892	0,001	**29**	5,9
STRADA	rs750512077	ms	Q7RTN6	215	R/H	-0,405	2,8E-09	**0,999**	352	1,5
STRN4	rs765739479	ms	Q9NRL3	373	P/S	0,358	0,001366	**0,992**	112	1,5
STX5	rs564823443	ms	Q13190	193	V/I	0,404	0,014596	**0,729**	**55**	9,1
SUCLG2	rs201519398	ms	Q96I99	374	N/S	-0,573	0,026768	0,060	**24**	**20**
SULF1	rs140542311	ms	Q8IWU6	359	V/I	-0,680	0,030464	0,249	**13**	**31**
SULF2	rs752358752	ms	Q8IWU5	205	R/H	-0,688	0,003884	**1,000**	**28**	1,3
SULT1A3	rs2073441082	ms	P0DMM9	108	H/Y	-0,362	0,010698	**0,944**	**84**	0,3
SUN1	rs779427205	ms	O94901	406	G/A	-0,533	0,002839	0,061	**45**	0
SUPT3H	rs150635035	ms	O75486	88	R/H	0,505	0,024291	**0,996**	**19**	1,1
SUPV3L1	rs199507911	ms	Q8IYB8	415	N/S	-0,775	0,007378	0,003	**22**	**24**
SUSD2	rs776550297	stop	Q9UGT4	168	Y/*	-0,393	0,050349	**1,000**	**35**	0,2
SUSD2	rs766473126	ms	-	576	P/S	**1,021**	0,005562	0,231	**10**	0,8
SUSD3	rs149456736	ms	Q96L08	222	Q/K	-0,451	0,033956	0,007	**29**	226
SUZ12	rs1272910292	ms	Q15022	691	G/V	0,663	0,005657	0,001	**30**	0
SYCN	rs766050306	ms	Q0VAF6	35	T/R	0,393	0,024871	**0,691**	**52**	0,4
SYNE2	rs200319405	ms	Q8WXH0	757	L/S	-0,775	0,024416	**0,825**	**24**	**34**
SYNE2	rs199743242	ms	-	2171	L/V	-0,775	0,024441	**0,995**	**24**	**51**
SYNE2	rs750823503	ms	-	4	S/T	-0,425	0,018640	0,006	**63**	0,4
SYNGR2	rs11557900	ms	O43760	21	L/V	-0,748	0,034856	0,007	**18**	411
SYNPO	rs371137506	ms	Q8N3V7	39	E/D	0,651	0,011639	**0,871**	**23**	4,9
SYNPO2L	rs1564989451	ms	Q9H987	894	A/S	-0,513	0,032216	**0,688**	**20**	0,1
SYT12	rs141165304	ms	Q8IV01	380	R/H	0,490	0,016793	0,017	**22**	119
SYT4	rs149020215	ms	Q9H2B2	384	R/Q	**1,084**	0,001435	-	**15**	**15**
SYT6	rs370048571	ms	Q5T7P8	481	E/K	0,545	0,030816	-	**23**	1,3
SYTL1	rs1557548678	ms	Q8IYJ3	471	P/S	0,845	0,047288	**1,000**	**15**	0,2
SZT2	rs532357677	ms	Q5T011	1574	R/Q	-0,402	0,008728	0,264	**58**	1,9
SZT2	rs757113547	ms	-	1444	R/H	-0,402	0,008728	0,000	**59**	0,4
TAAR1	rs753259390	ms	Q96RJ0	7	N/T	0,611	0,037382	0,022	**26**	0,4
TACC2	rs142194146	ms	O95359	948	R/W	0,469	0,005440	0,117	**72**	**29**
TAF7	rs139292924	ms	Q15545	131	I/M	0,630	0,045063	**0,723**	**24**	**74**
TAFA1	rs372722058	ms	Q7Z5A9	119	A/T	0,923	0,031314	0,145	**12**	3,0
TALDO1	rs1804554	ms	P37837	300	E/K	0,914	0,031960	**0,953**	**15**	3,4
TANC1	2:159219263:G/A	ms	Q9C0D5	1135	G/D	**-1,032**	0,015280	0,186	**10**	N/A
TANC2	rs370863314	ms	Q9HCD6	2015	R/P	0,479	8,16E-09	0,066	232	**12**
TANC2	rs780001108	ms	-	160	A/T	-0,441	0,000145	**0,896**	132	2,3
TANC2	rs554732900	ms	-	1884	R/Q	-0,388	0,009026	0,114	**78**	0,4
TANC2	rs746422304	ms	-	273	G/E	0,828	0,008412	0,063	**12**	7,2
TAOK2	rs138875458	ms	Q9UL54	750	I/V	0,723	0,048268	0,087	**16**	**19**

TARBP2	rs1565897406	ms	Q15633	12	T/A	0,886	0,022850	0,022	20	0
TAS1R1	rs140284805	ms	Q7RTX1	470	T/A	0,427	0,004335	0,087	71	137
TAS1R1	rs200196173	ms	-	678	G/S	0,377	0,001659	1,000	105	3,6
TAS1R3	rs763938709	ms	Q7RTX0	500	S/L	-0,887	0,015586	0,686	16	0,8
TAS2R19	rs767546611	ms	P59542	260	V/L	-0,462	0,037825	0,062	30	0,1
TAS2R3	rs771250168	ms	Q9NYW6	274	M/V	-0,446	0,043922	0,072	30	0,4
TAS2R31	rs201717335	ms	P59538	92	G/S	0,712	0,008217	0,007	19	58
TAS2R5	rs147887777	ms	Q9NYW4	55	R/Q	-0,546	0,022841	0,970	38	168
TBC1D16	rs1020548320	ms	Q8TBP0	55	L/R	-1,746	0,014260	0,161	17	7,1
TBC1D16	rs755651950	ms	-	116	T/I	-0,478	0,037211	0,000	29	8,7
TBC1D2	rs767385150	ms	Q9BYX2	245	E/V	0,486	0,007072	-	58	1,5
TBC1D31	rs780616960	ms	Q96DN5	487	W/R	0,361	0,030574	1,000	44	1,5
TBC1D32	rs200973240	ms	Q96NH3	770	R/G	1,184	0,049320	0,021	10	21
TBC1D3B	rs1231609801	ms	A6NDS4	284	R/H	-0,659	0,006936	0,941	34	80
TBC1D3F	rs1184505888	ms	A6NER0	323	R/S	0,448	0,037997	0,009	37	0
TBC1D8	rs201324556	ms	O95759	509	R/H	0,523	0,015971	0,983	30	41
TBCD	rs761639016	ms	Q9BTW9	579	I/T	0,644	0,022827	0,823	19	0
TBCE	rs143917509	ms	Q15813	489	L/I	-0,421	0,014077	0,009	55	80
TBCK	rs766079704	ms	Q8TEA7	344	E/Q	-0,430	0,028330	0,012	60	0,7
TBCK	rs781374842	ms	-	43	Q/R	-0,685	0,014036	0,988	30	1,9
TBX10	rs149888346	ms	O75333	200	F/L	0,485	0,017763	0,106	48	25
TBX21	rs2032313569	ms	Q9UL17	417	M/V	-0,971	0,001800	0,000	17	2,9
TBX3	rs762031545	ms	O15119	454	P/L	-0,580	0,037356	0,001	25	6,0
TBX3	rs1012420316	ms	-	71	A/S	0,424	0,037558	0,973	25	0,9
TCAF2	rs1809518551	ms	A6NFQ2	518	C/R	-0,390	0,009113	0,584	81	0
TCAF2	7:143723411:C/G	ms	-	710	R/G	0,663	0,022168	0,928	15	N/A
TCF3	rs138963927	ms	Q9HCS4	77	E/K	0,377	0,045207	0,134	49	11
TCF7L1	rs1434102693	ms	-	67	S/L	-0,988	0,002769	0,015	14	0,8
TCTE1	rs146833594	ms	Q5JU00	310	E/K	0,474	0,022987	0,163	35	152
TDP2	rs763334299	ms	O95551	282	D/A	0,739	0,001455	1,000	34	0
TDRD10	rs146616270	ms	Q5VZ19	263	A/T	-0,585	0,036376	0,996	18	92
TEC	rs761984494	ms	Q92570	269	G/D	0,729	0,006290	0,413	16	0
TECTB	rs746498190	ms	Q96PL2	214	Y/H	0,663	0,022162	0,901	23	4,2
TEPSIN	rs777674986	ms	Q96N21	593	A/S	0,699	0,034135	0,005	10	2,0
TEX14	rs1454744660	ms	Q8IWB6	291	E/K	-0,741	0,008761	0,826	31	1,9
TEX14	rs775971945	ms	-	537	Y/C	-0,790	0,000412	0,010	33	0,4
TEX15	rs1563237429	ms	Q9BXT5	1595	H/R	0,688	0,013468	0,006	20	0,4
TEX2	rs372604630	ms	Q8IWB9	496	S/R	0,593	0,040906	0,595	18	15
TEX2	rs780968826	ms	-	375	E/K	0,366	9E-10	0,003	486	0,5
TEX2	rs150175049	ms	-	463	D/N	-0,868	0,001033	0,000	31	119
TEX29	rs375512703	ms	Q8N6K0	54	A/V	0,433	0,051362	0,871	36	2,3
TF	rs121918677	ms	P02787	671	G/E	-0,373	0,051829	1,000	35	275
TG	rs781688385	ms	P01266	2123	F/Y	0,458	0,001178	0,740	76	0,2
TGFBI	rs201158209	ms	Q15582	354	N/S	-1,230	0,041089	0,261	11	24
TGFBR3	rs137909765	ms	Q03167	790	I/F	0,353	0,021755	0,999	76	82
THEM4	rs776286488	ms	Q5T1C6	12	L/P	-0,358	0,050404	0,553	61	2,1
THEMIS	rs373934545	ms	Q8N1K5	251	E/Q	-1,069	0,003751	0,990	11	2,6
TIAM1	rs16987932	ms	Q13009	844	Q/H	-0,905	0,006001	-	17	5119
TIGIT	rs13098836	ms	Q495A1	33	I/V	0,557	0,023424	0,039	34	278
TIMELESS	rs766677008	ms	Q9UNS1	753	R/C	0,433	0,041829	0,916	30	6,0
TIMM21	rs145075415	ms	Q9BVV7	127	T/M	-0,713	0,040170	0,723	16	1,9
TIMP2	rs769578055	ms	P16035	173	L/P	0,538	0,001621	-	49	5,3
TJAP1	rs1561823029	ms	Q5JTD0	123	S/N	0,649	0,024771	0,567	20	0
TKT	rs781993830	ms	Q16832	559	V/M	-0,908	0,001606	1,000	30	2,3
TKT	rs757757097	ms	-	350	I/V	-0,444	0,002308	0,010	72	1,5
TKT	rs762983878	stop	-	514	E/*	-0,371	0,001071	1,000	128	1,1
TLE6	rs373921962	ms	Q9H808	133	R/Q	0,690	0,028007	0,044	14	3,8
TLK2	rs746810639	ms	Q86UE8	70	Y/H	-0,903	0,029379	0,003	11	0,1
TLR3	rs73025939	ms	O15455	643	R/C	1,422	4,26E-05	0,985	11	138
TM6SF2	rs763226075	ms	Q9BZW4	109	G/A	0,415	0,020088	0,996	50	0,8
TM6SF2	rs201189528	ms	-	139	N/K	0,364	0,030908	0,122	54	83
TMC6	rs147815166	ms	Q7Z403	583	R/Q	0,579	0,024841	0,022	26	98
TMC8	rs144981818	ms	Q8IU68	131	V/L	0,735	0,012062	0,354	18	5,6
TMCO3	rs764955516	ms	Q6UWJ1	561	A/T	-0,559	0,046625	0,062	28	0,8
TMED4	rs771263996	ms	Q7Z7H5	36	E/K	0,678	0,014931	0,675	29	0,2
TMEM101	rs756067708	ms	Q96IK0	136	R/H	0,680	0,005484	0,991	21	0,6
TMEM131	rs889603986	ms	Q92545	13	T/N	0,530	0,046246	0,000	26	42
TMEM138	rs187122512	ms	Q9NPI0	129	E/K	0,423	0,035437	0,000	37	274
TMEM151B	rs753916563	ms	Q8IW70	408	G/D	0,438	0,028596	0,125	36	1,9
TMEM161B	rs547282299	ms	Q8NDZ6	443	P/L	-1,017	0,026711	0,000	14	17
TMEM176B	rs753351031	ms	Q3YBM2	171	F/V	0,503	0,027036	0,000	26	4,3
TMEM208	rs374909823	ms	Q9BTX3	161	R/Q	0,375	0,036014	0,000	42	3,4
TMEM229A	rs376966840	ms	B2RXF0	266	G/E	0,595	0,039742	0,153	29	3,8
TMEM231	rs199605221	ms	Q9H6L2	264	E/A	0,468	0,019860	0,897	35	76
TMEM233	rs371582871	ms	B4DJY2	83	K/M	-0,684	0,007155	0,808	29	7,9
TMEM238L	rs200099798	ms	A6NJY4	10	C/R	-0,761	0,015198	0,598	19	79
TMEM259	rs1568399471	fs	Q4ZIN3	427	S/X	-1,299	0,026843	1,000	15	0
TMEM262	rs765511199	ms	E9PQX1	31	G/D	0,821	0,026048	0,970	16	12
TMEM44	rs142364954	ms	Q2T9K0	369	V/I	0,359	0,018037	0,007	56	28
TMEM88	rs201535986	ms	Q6PEY1	111	R/H	0,498	0,024512	0,711	28	42

TMF1	rs1559628559	ms	P82094	921	R/H	0,670	0,042366	0,005	13	0,1
TMF1	rs768748751	ms	-	284	S/L	1,060	0,042153	0,170	11	0
TMPRSS11B	rs575638339	ms	Q86T26	207	R/H	0,944	0,042826	0,003	13	2,6
TMPRSS13	rs749737407	ms	Q9BYE2	460	P/S	0,365	0,023288	0,042	73	0,1
TMPRSS5	rs1263487635	ms	Q9H3S3	249	A/V	0,909	0,030113	0,482	15	1,1
TMX1	rs766355722	ms	Q9H3N1	269	R/C	0,535	0,044735	-	27	1,9
TNC	rs149986851	ms	P24821	203	G/V	0,369	0,030467	0,980	65	28
TNC	rs751818291	ms	-	1903	A/S	-0,477	0,003632	0,999	62	0,4
TNC	rs139280264	ms	-	1066	R/C	-0,536	0,030794	0,577	23	83
TNFRSF9	rs183916313	ms	Q07011	193	A/V	-0,467	0,015713	0,030	36	3,0
TNFSF8	rs776716866	ms	P32971	58	M/T	-0,936	0,003198	0,111	19	1,9
TNIP1	rs768349035	stop	Q15025	636	*/Q	0,569	0,024516	1,000	21	0,1
TNNT2	rs367785431	ms	P45379	296	R/C	-1,161	0,000845	0,924	14	1,9
TNPO1	rs772921669	ms	Q92973	749	I/M	0,871	0,040867	0,010	14	1,7
TNR	rs770776102	ms	Q92752	347	D/Y	-0,366	0,047540	0,990	48	0
TNRC18	rs200928367	ms	O15417	1549	G/S	1,262	0,003029	0,000	13	46
TNRC6A	rs149360617	ms	Q8NDV7	287	T/A	-0,727	0,026381	0,005	14	61
TNRC6A	16:24789589:C/G	ms	-	316	S/C	-0,687	0,048084	0,308	15	N/A
TNRC6B	rs577376433	ms	Q9UPQ9	556	P/S	1,102	0,000748	0,986	19	3,8
TNS1	rs767046731	ms	Q9HBL0	176	H/R	-0,540	0,023520	0,996	26	0,4
TNS3	rs201649683	ms	Q68CZ2	815	V/I	0,435	0,001819	0,000	84	45
TNXB	rs190411129	ms	P22105	2798	R/C	-0,506	0,006461	0,931	55	23
TNXB	rs185207099	ms	-	1163	G/E	-0,826	0,025638	0,869	14	73
TOB2	rs148432877	ms	Q14106	143	I/V	-0,635	0,021805	0,567	20	65
TOGARAM1	rs770089950	ms	Q9Y4F4	1176	S/P	0,843	0,046743	0,997	12	0,2
TOP2A	rs764177670	ms	P11388	530	T/M	0,506	0,012587	0,931	30	6,4
TOP2A	rs746667611	ms	-	1118	V/I	-1,173	0,008711	0,066	17	1,5
TOP3A	rs777874588	ms	Q13472	542	I/M	-0,799	0,021123	0,969	20	1,1
TOP3A	rs139844084	ms	-	629	E/K	0,392	0,006224	0,213	97	244
TOP3B	rs1250857443	ms	O95985	755	A/V	-0,772	0,000118	0,000	27	0,8
TOPORS	rs200405067	ms	Q9NS56	118	R/C	0,375	0,032095	0,000	53	16
TPCN1	rs199608038	ms	Q9ULQ1	728	R/Q	0,813	0,002531	0,013	25	2,3
TPCN2	rs34510004	ms	Q8NHX9	546	M/I	0,856	0,044722	0,003	16	63
TPM3	rs755506890	ms	P06753	278	A/V	0,406	0,046156	0,189	27	0,2
TPMT	rs1800462	ms	P51580	80	A/P	0,685	0,017907	0,972	24	255
TPTE2	rs747723029	ms	Q6XPS3	399	K/I	-0,455	0,012198	0,927	61	119
TRAF3IP1	rs749544012	ms	Q8TDR0	442	P/T	0,930	0,007497	-	17	0,4
TRANK1	rs559712651	ms	O15050	1500	R/W	-0,659	0,022770	0,812	25	2,3
TRANK1	rs749619179	ms	-	822	I/L	0,548	0,035741	0,005	18	0
TRAP1	rs148549350	ms	Q58FF3	469	R/C	1,098	0,005334	0,999	15	35
TRAPPC12	rs777185825	ms	Q8WVT3	212	D/E	-0,452	0,037985	0,029	37	15
TRAPPC2B	rs1168689303	ms	P0DI82	98	F/L	-0,554	0,038417	0,959	19	1,1
TRAPPC9	rs771541938	ms	Q96Q05	409	R/C	-0,767	0,010254	0,990	15	0,4
TRAPPC9	rs143778652	ms	-	340	A/V	-0,752	0,030515	0,987	12	55
TREM1	rs756591461	ms	Q9NP99	112	Q/H	-0,432	0,025425	0,451	58	0,1
TRIM11	rs758801204	ms	Q96F44	147	R/Q	0,647	0,049985	0,005	19	4,2
TRIM11	rs1558445080	ms	-	303	S/F	-0,949	0,016106	0,985	11	0,4
TRIM22	rs780626396	ms	Q8IYM9	74	L/R	1,771	0,003284	0,995	13	26
TRIM37	rs771449822	ms	O94972	51	C/R	-0,773	0,025588	1,000	11	0
TRIM37	rs376721539	ms	-	777	A/T	0,566	0,038809	0,626	16	4,9
TRIM37	rs143642427	ms	-	789	D/E	-0,395	0,000341	0,005	141	40
TRIM40	rs113286964	ms	Q6P9F5	59	P/H	0,590	0,008042	0,884	51	956
TRIM44	rs777604610	ms	Q96DX7	334	G/R	0,487	0,043492	0,000	19	0,8
TRIM67	rs534389734	ms	Q6ZTA4	273	G/D	0,357	0,021432	0,960	72	154
TRIM71	rs781070829	ms	Q2Q1W2	336	R/Q	0,505	0,030332	0,249	34	11
TRIM72	rs780638717	ms	Q6ZMU5	206	R/L	0,447	0,032042	0,001	30	17
TRIM72	rs757603858	ms	-	196	R/C	0,665	0,036015	0,755	15	1,1
TRIM75	rs750797794	ms	A6NK02	176	V/M	-0,639	0,033711	0,120	59	0,2
TRIO	rs892410126	ms	O75962	2310	G/V	-0,420	0,020194	-	30	16
TRIO	rs201650861	ms	-	507	S/L	-1,500	0,041992	0,000	15	1,5
TRIO	rs1487014323	ms	-	702	S/L	-0,771	0,036487	0,000	11	1,1
TRIP11	rs1566861259	ms	Q15643	534	Q/E	-0,512	0,042055	0,000	17	0
TRIP6	rs778314148	ms	Q15654	160	A/P	0,509	0,044196	0,965	19	0,4
TRIP6	rs1310784349	ms	-	374	G/D	-1,422	0,018084	0,958	11	0,8
TRMT2A	rs771115771	ms	Q8IZ69	604	P/A	0,409	0,021942	0,001	56	0,3
TRMT44	rs778957821	ms	Q8IYL2	11	Y/D	0,614	0,029604	0,000	15	0,4
TRMT6	rs148182864	ms	Q9UJA5	166	R/C	-0,612	0,029056	0,879	29	44
TRNAU1AP	rs774377370	ms	Q9NX07	267	A/T	0,505	0,011431	0,879	41	0,8
TRPC3	rs765481480	ms	Q13507	108	S/N	1,014	0,000277	0,007	16	0
TRPC6	rs745835425	ms	Q9Y210	870	A/G	-0,536	0,046867	0,697	19	0,3
TRPM2	rs747955952	ms	O94759	816	V/M	-0,847	0,029540	0,942	10	1,6
TRPM8	rs139760142	ms	Q7Z2W7	939	L/M	-0,607	0,010846	0,159	26	2,3
TRPM8	rs370999195	ms	-	607	D/N	0,589	0,024029	0,285	27	0,8
TSC2	rs45517203	ms	P49815	607	A/T	0,398	0,044683	0,996	49	53
TSC2	rs45482691	ms	-	91	P/L	-0,434	0,045534	0,998	35	53
TSPAN33	rs150641480	ms	Q86UF1	139	D/H	0,763	0,038298	0,995	15	7,6
TSPOAP1	rs139104560	ms	O95153	1584	A/P	0,428	0,005177	0,000	63	59
TTBK2	rs758756132	ms	Q6IQ55	698	M/V	0,466	0,016410	0,000	40	0,4
TTC13	rs202049212	ms	Q8NBP0	840	T/M	-0,363	0,036853	0,701	58	31
TTC21A	rs199900796	ms	Q8NDW8	650	P/T	0,418	0,017130	0,021	42	23

TTC23	rs774316772	stop	Q5W5X9	401	Q/*	0,887	0,024383	1,000	13	0
TTC28	rs376490616	ms	Q96AY4	550	R/C	-0,558	0,019721	0,999	28	34
TTC3	rs1568881314	fs	P53804	169	L/X	-0,491	0,026343	1,000	34	0
TTLL12	rs1398457638	ms	Q14166	503	N/S	0,702	0,006262	0,027	21	1,2
TTLL2	rs200952819	ms	Q9BWV7	249	I/F	0,381	0,028371	0,961	42	6,0
TTLL2	rs376612341	ms	-	4	R/Q	-0,356	0,009325	0,000	115	3,8
TTLL5	rs367910225	ms	Q6EMB2	697	G/S	-0,565	0,012336	0,000	33	7,9
TTLL7	rs1557607066	ms	Q6ZT98	657	A/D	-0,819	0,006569	0,000	18	0
TTN	rs773791222	ms	Q8WZ42	21335	E/K	0,590	0,008012	-	41	6,0
TTN	rs374656017	ms	-	22197	R/Q	-0,587	0,020496	-	35	15
TTN	rs150667217	ms	-	1202	V/A	0,495	0,000429	-	84	26
TTN	rs758449770	ms	-	18019	E/K	-0,587	0,020514	-	35	0
TTN	rs201825412	ms	-	17261	R/Q	0,444	0,001918	-	83	15
TTN	rs1559263086	ms	-	29106	E/G	-0,691	0,036235	-	14	0,2
TTN	rs56372592	ms	-	25580	T/K	0,495	0,000431	-	84	239
TUBD1	rs776246231	ms	Q9UJT1	151	G/R	0,498	0,009185	1,000	42	0,8
TUBGCP4	rs191224065	ms	Q9UGJ1	323	V/L	-0,525	0,050921	0,007	26	78
TUBGCP6	22:50221172:C/A	ms	Q96RT7	1063	V/F	0,567	0,001637	0,760	64	N/A
TUBGCP6	22:50221173:C/G	ms	-	1062	R/S	0,567	0,001637	0,150	64	N/A
TUFM	rs760673155	ms	P49411	144	T/A	-0,401	0,028354	0,000	41	0,4
TXNDC11	rs151281237	ms	Q6PKC3	900	T/S	-0,542	0,036761	0,035	27	34
TXNDC11	rs146197324	ms	-	875	R/H	-0,411	0,023922	0,001	42	33
TXNDC16	rs147641850	ms	Q9P2K2	331	P/S	0,607	0,043812	0,080	18	36
TXNIP	rs150672123	ms	Q9H3M7	312	R/Q	-0,721	0,007139	0,000	23	50
TYW1	rs370745036	ms	Q9NV66	518	A/V	0,731	0,010431	0,005	21	3,8
UBAP1	rs1563920368	ms	Q9NZ09	175	D/N	0,616	0,028492	0,990	17	0,2
UBAP2L	rs766782624	ms	Q14157	763	G/S	-0,504	0,040444	0,003	29	0,1
UBD	rs200363304	ms	O15205	138	R/K	-1,358	0,009295	0,001	13	6,8
UBXN6	rs200014531	ms	Q9BZV1	45	R/C	-0,734	0,025889	0,940	17	7,2
UCHL5	rs146351256	ms	Q9Y5K5	321	A/T	1,088	0,005784	0,000	14	38
UCN3	rs782142877	ms	Q969E3	94	R/W	1,168	0,012078	0,997	12	1,9
UFL1	rs760861177	ms	O94874	240	D/N	-0,517	0,035256	0,878	30	2,6
UFM1	rs1566029075	ms	P61960	5	S/F	0,782	0,046079	0,710	16	0,4
UGGT2	rs1566664454	fs	Q9NYU1	798-799	-/X	-0,888	0,015228	1,000	22	0
UGP2	rs762421534	ms	Q16851	81	P/H	0,394	0,003477	0,934	99	0,8
UGT1A4	rs760663829	ms	P22310	258	R/Q	0,789	0,022985	0,999	16	1,1
UGT2A3	rs137969795	ms	Q6UWM9	394	F/S	0,600	0,002782	0,994	49	9,1
UGT2B10	rs1976666	ms	P36537	283	P/A	0,490	0,006936	0,941	58	857
UGT2B17	rs186138322	ms	O75795	480	A/T	-1,453	0,000228	0,921	15	59
UGT3A1	rs766764383	ms	Q6NUS8	334	S/C	0,707	0,041943	0,985	12	0
ULK1	rs146186922	ms	O75385	660	T/M	0,751	0,000718	0,959	31	5,3
UMOD	rs199835347	ms	P07911	142	R/Q	0,771	0,019467	0,014	10	144
UMODL1	rs757493344	ms	Q5DID0	30	S/C	0,731	0,023657	0,997	17	1,1
UNC119	rs146916036	ms	Q13432	168	R/C	-0,797	0,021447	1,000	35	45
UNC13B	rs1829737656	ms	O14795	1047	V/I	-0,997	0,031536	0,000	13	0
UNC45A	rs566664532	ms	Q9H3U1	358	P/A	0,752	0,031122	-	12	1,1
UPF3A	rs376489246	ms	Q9H1J1	63	K/E	0,551	0,034245	0,184	27	3,4
URB1	rs771374770	ms	O60287	105	I/V	-0,574	0,042054	0,556	14	2,3
URB2	rs766210522	ms	Q14146	1063	L/F	-0,453	0,046785	0,554	23	0,3
URI1	rs772890952	ms	O94763	176	R/Q	-0,418	0,030960	0,997	45	1,5
USF3	rs745332813	ms	Q68DE3	196	V/L	-1,454	0,005321	0,048	16	2,5
USH2A	rs139089840	ms	O75445	1076	P/S	-0,390	0,047317	0,998	38	23
***USP**13*	rs1488181405	ms	Q92995	285	A/S	-0,992	0,011828	-	15	0,4
USP17L2	rs200055041	ms	Q6R6M4	126	C/Y	-0,439	0,007212	0,917	68	179
USP17L2	rs1563163368	ms	-	19	K/R	-0,776	0,025689	0,618	12	0,7
USP17L2	rs201369910	ms	-	156	G/C	0,351	0,035464	0,985	50	49
USP17L24	4:9326086:T/A	ms	Q0WX57	308	S/T	-0,449	0,027330	0,971	37	N/A
USP17L25	4:9330832:T/A	ms	-	308	S/T	-0,449	0,027326	0,971	38	N/A
USP17L26	4:9335579:T/A	ms	-	308	S/T	-0,449	0,027321	0,971	38	N/A
USP17L3	rs1820676921	ms	A6NCW0	77	A/V	0,458	0,045963	0,012	31	0
USP19	rs1559995833	ms	O94966	1082	S/R	-0,946	0,016492	-	16	0
USP19	rs1560004912	ms	-	884	P/S	0,616	0,040856	-	17	0,1
USP22	rs752874123	ms	Q9UPT9	363	T/M	-0,365	0,051370	0,046	61	0,3
USP28	rs142728209	ms	Q96RU2	631	R/I	1,220	0,042863	0,698	11	42
USP29	rs144679292	ms	Q9HBJ7	784	L/P	-1,131	0,004096	0,122	11	153
USP32	rs140463372	ms	Q8NFA0	1370	I/S	-0,717	0,051820	0,000	14	13
USP36	rs1567975159	ms	Q9P275	44	P/S	-0,956	0,034809	0,998	13	0,2
USP40	rs1559237710	ms	Q9NVE5	846	F/L	0,706	0,041838	0,236	12	0
USP42	rs200908439	ms	Q9H9J4	486	G/R	-0,989	0,011678	0,872	15	18
USP43	rs777462170	ms	Q70EL4	872	L/V	-0,413	0,042508	0,006	51	0
UTP15	rs776437309	ms	Q8TED0	288	Y/D	1,216	0,043359	0,973	10	0
UTP4	rs769827893	stop	Q969X6	546	S/*	-0,366	0,020253	1,000	58	0,8
UTRN	rs748187376	ms	P46939	3310	E/D	-0,408	0,020505	0,003	55	1,5
VAC14	rs145938865	ms	Q08AM6	575	R/W	-0,474	0,042376	0,901	36	76
VASH1	rs746572157	ms	Q7L8A9	334	R/C	-0,522	0,020027	0,993	48	0,7
VASN	rs746313383	ms	Q6EMK4	564	A/T	0,413	0,022480	0,070	50	6,8
VEPH1	rs139246642	ms	Q14D04	512	N/K	-0,385	0,008380	0,009	69	62
VEZF1	rs745913326	ms	Q14119	190	N/S	-0,361	0,044866	0,011	38	1,5
VLDLR	rs6149	ms	P98155	59	V/I	0,724	0,004580	0,042	31	6001
VPS11	rs765997836	ms	Q9H270	870	M/T	-0,681	0,018676	0,018	15	0

VPS13B	rs140061281	ms	Q7Z7G8	2547	S/G	0,972	0,041496	0,000	12	6,0
VPS13B	rs61754112	ms	-	1452	L/F	1,137	0,014719	0,015	15	2,6
VPS13C	rs1567071824	ms	Q709C8	676	T/A	0,786	0,032903	0,585	16	0
VPS13D	rs757964463	ms	Q5THJ4	4228	R/W	-0,684	0,049243	0,990	14	0,1
VPS13D	rs758101809	ms	-	1604	Q/P	0,607	0,011056	0,140	24	5,3
VPS18	rs200156144	ms	Q9P253	835	R/Q	-0,359	0,039112	0,000	36	28
VPS41	rs199884913	ms	P49754	765	V/I	0,909	0,038978	0,838	14	5,3
VPS50	rs146809545	ms	Q96JG6	493	R/H	-0,740	0,042676	0,657	11	17
VPS53	rs752241629	ms	Q5VIR6	626	W/R	0,606	0,045699	0,999	22	1,1
VRK3	rs747070456	ms	Q8IV63	413	P/T	0,577	0,014857	0,003	42	2,7
VWA1	rs747475277	ms	Q6PCB0	71	L/P	-0,539	0,033159	0,748	20	5,7
VWA5B1	rs777449498	ms	Q5TIE3	729	P/L	-0,436	0,012571	0,811	55	1,1
VWA8	rs141997392	ms	A3KMH1	625	P/L	0,601	0,031007	0,266	34	53
VWCE	rs529893923	ms	Q96DN2	465	V/I	0,693	0,026833	0,007	11	2,3
WASF3	rs375670514	ms	Q9UPY6	31	N/S	-1,365	0,019915	0,934	11	1,5
WASHC2C	10:45752594:A/C	ms	Q9Y4E1	337	E/A	0,535	0,028059	0,558	42	N/A
WASHC5	rs200456170	ms	Q12768	406	N/S	0,508	0,004476	0,007	47	7,6
WDCP	rs147244110	ms	Q9H6R7	502	P/L	-0,385	0,041475	0,052	51	247
WDR12	rs1253312677	ms	Q9GZL7	51	V/M	-0,668	0,006640	0,062	15	3,8
WDR19	rs199783864	ms	Q8NEZ3	310	Y/C	-0,775	0,005425	0,030	21	17
WDR27	rs537173344	ms	A2RRH5	413	V/I	-0,366	0,028184	0,000	52	143
WDR59	rs1567687673	ms	Q6PJI9	829	G/E	0,676	0,009492	0,026	16	0
WDR64	rs777137576	ms	B1ANS9	524	L/I	-0,412	0,044196	0,000	50	0,4
WDR81	rs1423977378	ms	Q562E7	1706	L/R	0,719	0,006731	0,995	13	0,4
WDR83	rs774285413	ms	Q9BRX9	68	Y/C	-0,453	0,047944	0,894	32	1,9
WDR90	rs199805518	ms	Q96KV7	701	A/T	-0,551	0,007461	0,792	42	7,2
WDR90	rs766480010	ms	-	918	R/C	0,408	0,023511	0,034	28	4,5
WFS1	rs1805070	ms	O76024	720	I/V	0,533	0,013430	0,014	48	325
WNT10A	rs145641272	ms	Q9GZT5	172	R/W	0,527	0,031530	0,803	33	21
WSCD2	rs780830224	ms	Q2TBF2	234	R/Q	-0,531	0,017038	0,608	15	2,3
WWTR1	rs747164910	ms	Q9GZV5	140	R/K	0,354	0,014274	0,000	53	3,4
YTHDC2	rs760176014	ms	Q9H6S0	921	E/G	-0,429	0,020764	0,089	38	0
ZAN	rs762790622	ms	Q9Y493	333	N/S	-0,705	0,014791	0,007	28	0,1
ZBTB38	rs75545796	ms	Q8NAP3	962	V/L	0,651	0,042930	0,000	16	125
ZBTB38	rs201503155	ms	-	588	N/I	1,113	0,017012	0,225	11	8,3
ZBTB41	rs200742919	ms	Q5SVQ8	206	N/S	-0,385	0,043801	0,000	31	32
ZBTB48	rs141447787	ms	P10074	273	A/V	-1,162	0,000797	0,000	18	5,3
ZC3H11A	rs779675915	ms	O75152	249	V/I	-0,491	0,050451	0,001	27	1,5
ZC3H13	rs144881601	ms	Q5T200	216	S/N	-0,564	0,009475	0,000	33	170
ZC3H13	rs746706384	ms	-	513	R/Q	0,616	0,040346	0,000	19	0,6
ZC3H18	rs200921944	ms	Q86VM9	58	P/L	0,814	0,041785	0,000	17	103
ZC3H7A	rs371372684	ms	Q8IWR0	320	P/S	0,456	0,020846	0,454	40	15
ZC3H7B	rs776780190	ms	Q9UGR2	369	R/Q	-0,656	0,023187	0,777	16	1,1
ZC3H8	rs1558927450	ms	Q8N5P1	135	G/V	0,988	0,007432	0,000	16	0,4
ZCCHC8	rs1400262196	ms	Q6NZY4	360	D/N	-0,677	0,040058	0,370	14	0,2
ZDBF2	rs370681691	ms	Q9HCK1	2047	R/W	-0,415	0,051414	0,668	37	4,9
ZDHHC12	rs201386601	ms	Q96GR4	194	A/P	0,620	0,027403	0,573	35	0,4
ZDHHC8	rs764513650	ms	Q9ULC8	532	R/C	0,835	0,048999	0,728	11	6,0
ZFC3H1	rs761708297	ms	O60293	1037	S/C	0,507	0,029768	0,852	26	0,4
ZFHX3	rs754898697	ms	Q15911	3187	T/M	-0,465	0,048444	0,000	43	0
ZFP28	rs142184982	ms	Q8NHY6	193	E/K	-0,483	0,018205	0,003	43	16
ZFP42	rs761262366	ms	Q96MM3	201	N/D	0,765	0,014942	0,003	11	0,7
ZFP64	rs765375562	ms	Q9NTW7	19	P/L	0,450	0,032128	0,000	55	5,3
ZFR2	rs199859071	ms	Q9UPR6	916	R/W	-0,535	0,046906	0,000	26	64
ZFYVE1	rs1433679547	ms	Q9HBF4	756	P/H	-1,317	0,011957	1,000	12	0
ZFYVE16	rs372767661	ms	Q7Z3T8	1144	L/P	-0,780	0,023639	0,997	20	5,3
ZFYVE26	rs371201801	ms	Q68DK2	1834	H/Y	0,919	0,050384	0,860	12	4,1
ZFYVE26	rs765803005	ms	-	1714	M/V	-0,642	0,032764	0,001	29	0,8
ZGLP1	rs199597072	ms	P0C6A0	193	T/S	-0,530	0,012717	0,000	39	18
ZHX1	rs765258778	ms	Q9UKY1	26	S/T	0,706	0,018957	0,018	13	0
ZIC1	rs138004710	ms	Q15915	110	A/P	0,412	0,030365	0,999	54	34
ZKSCAN2	rs1567354833	stop	Q63HK3	185	R/*	-2,018	0,025704	1,000	12	0,1
ZMIZ1	rs1564594224	ms	Q9ULJ6	461	P/S	-0,548	0,032053	0,009	20	0
ZMIZ1	rs376587677	ms	-	1050	P/A	-0,351	0,040608	0,000	62	1,3
ZMIZ2	rs750569363	ms	Q8NF64	631	N/S	-0,432	0,028222	0,998	53	0,7
ZMIZ2	rs751824063	ms	-	415	S/N	-0,385	0,033384	0,000	44	0
ZNF106	rs371374573	ms	Q9H2Y7	1301	N/K	0,417	0,049275	0,152	21	1,9
ZNF106	rs756910453	ms	-	461	P/S	0,932	0,007023	0,003	17	0,4
ZNF131	rs199798066	ms	P52739	536	T/S	0,394	0,033130	0,082	40	29
ZNF142	rs751858230	ms	P52746	207	R/C	0,759	0,000590	0,824	33	0,5
ZNF16	rs200923173	ms	P17020	269	E/K	0,423	0,031115	0,001	44	2,6
ZNF189	rs200682925	ms	O75820	622	T/I	-1,004	0,000589	0,031	18	0,8
ZNF200	rs61731430	ms	P98182	22	P/L	0,872	0,008199	0,229	17	40
ZNF213	rs377090397	ms	O14771	309	R/Q	0,381	0,039902	0,018	42	36
ZNF213	16:3141064:T/G	ms	-	366	V/G	-0,723	0,009433	0,999	26	N/A
ZNF232	rs200025811	ms	Q9UNY5	291	V/I	0,491	0,035656	0,005	21	6,4
ZNF235	rs757329633	ms	Q14590	140	P/A	0,459	0,046036	0,000	38	0,4
ZNF253	rs1568502375	ms	O75346	442	H/R	-0,677	0,018824	0,994	21	0,6
ZNF263	rs764198314	ms	O14978	139	V/M	-0,694	0,016307	0,000	32	0,7
ZNF268	rs543328816	ms	Q14587	732	S/F	-0,534	0,049956	0,955	14	17

ZNF273	rs768377540	ms	Q14593	448	H/Q	-0,870	0,010722	**1,000**	**11**	0,8
ZNF281	rs779663260	ms	Q9Y2X9	243	P/L	-0,492	0,018547	**0,981**	**27**	0,4
ZNF285	rs143574007	ms	Q96NJ3	507	I/T	-0,856	0,013758	0,000	**10**	**75**
ZNF292	rs201167963	ms	O60281	2224	K/N	-0,386	0,045925	**0,918**	**38**	7,6
ZNF292	rs752386780	ms	-	2048	K/N	0,352	0,044174	0,003	**46**	0,9
ZNF335	rs368892465	ms	Q9H4Z2	657	R/L	0,482	0,014256	0,185	**38**	1,5
ZNF354A	rs151334905	ms	O60765	15	T/M	-0,980	0,025856	**0,999**	**15**	**19**
ZNF395	rs1226932987	ms	Q9H8N7	2	A/V	**1,555**	0,009779	0,000	**12**	0
ZNF396	rs779377576	ms	Q96N95	148	M/V	-0,405	0,009534	0,000	**38**	0
ZNF408	rs769357381	ms	Q9H9D4	442	C/R	**1,523**	0,038991	**0,999**	**12**	1,1
ZNF423	rs201588114	ms	Q2M1K9	468	V/I	0,942	0,001758	0,103	**18**	**36**
ZNF430	rs148598454	ms	Q9H8G1	72	V/A	0,569	0,024384	0,054	**26**	**15**
ZNF430	rs139798103	ms	-	523	S/R	0,569	0,024384	0,003	**26**	**25**
ZNF436	rs773419541	ms	Q9C0F3	62	E/K	-0,856	0,044535	0,301	**11**	0,4
ZNF442	rs371108711	ms	Q9H7R0	309	R/Q	-0,779	0,023752	0,039	**22**	9,1
ZNF445	rs1222577044	ms	P59923	399	K/N	-0,771	0,047554	**0,549**	**13**	0,2
ZNF445	rs1359759859	ms	-	216	P/L	-0,972	0,020343	0,042	**16**	1,5
ZNF446	rs1041860970	ms	Q9NWS9	294	L/S	0,940	0,024878	0,000	**19**	1,1
ZNF462	rs1377404584	ms	Q96JM2	2121	L/F	-0,432	0,045357	0,316	**20**	0,2
ZNF469	rs772817384	ms	Q96JG9	1317	K/R	-0,387	0,022100	0,012	**66**	**46**
ZNF470	rs775542377	ms	Q6ECI4	243	L/I	-0,622	0,043099	**0,969**	**18**	1,5
ZNF493	rs1568384384	fs	Q6ZR52	564	K/X	0,382	0,041242	**1,000**	**55**	0
ZNF497	rs200614748	ms	Q6ZNH5	169	K/E	-0,422	0,043593	**0,998**	**24**	**21**
ZNF507	rs769948845	ms	Q8TCN5	477	N/K	-0,525	0,004293	**0,996**	**53**	9,4
ZNF525	rs572076035	ms	Q8N782	123	T/I	0,542	0,044167	**0,492**	**22**	134
ZNF526	rs750582519	ms	Q8TF50	151	S/L	0,698	0,007361	**0,968**	**25**	0,4
ZNF528	rs1397969661	ms	Q3MIS6	192	G/S	-0,758	0,027722	**0,498**	**16**	**30**
ZNF554	rs199517922	ms	Q86TJ5	208	V/L	-0,558	0,032338	0,007	**16**	**28**
ZNF562	rs200884863	ms	Q6V9R5	163	I/L	-0,462	0,046038	0,000	**37**	**16**
ZNF562	rs200076265	ms	-	162	S/I	-0,462	0,046038	0,000	**37**	**28**
ZNF571	rs1568340343	ms	Q7Z3V5	356	I/F	-0,890	0,023891	**0,840**	**16**	0
ZNF572	rs768125382	ms	Q7Z3I7	186	K/T	0,363	0,000755	**0,998**	151	0,2
ZNF587	rs1302318046	ms	Q96SQ5	83	R/G	0,383	0,049432	0,158	**36**	1,3
ZNF592	rs151234929	ms	Q92610	172	P/L	0,641	0,051305	0,441	**19**	**29**
ZNF598	rs117633338	ms	Q86UK7	367	Q/R	0,470	0,002273	0,000	**51**	**65**
ZNF614	rs1568513712	ms	Q8N883	365	H/Y	0,554	0,026906	**0,998**	**24**	0
ZNF618	rs62620240	ms	Q5T7W0	586	A/V	-0,941	0,042038	0,005	**16**	**77**
ZNF638	rs137918800	ms	Q14966	897	L/F	**1,588**	0,031441	0,044	**13**	1,9
ZNF639	rs780577272	ms	Q9UID6	414	S/G	-0,471	0,034343	**0,974**	**31**	0,2
ZNF654	rs774298577	ms	Q8IZM8	727	I/V	-0,482	0,023273	0,006	**38**	1,9
ZNF66	rs761559630	ms	Q6ZN08	415	H/R	-0,467	0,031163	**0,862**	**20**	1,1
ZNF662	rs200562529	ms	Q6ZS27	19	P/L	-0,651	0,046831	**0,722**	**20**	6,7
ZNF678	rs1558158212	ms	Q5SXM1	335	H/Y	-0,432	0,049199	0,145	**33**	0,4
ZNF682	rs761895895	ms	O95780	411	I/V	-0,412	0,004990	0,000	**85**	3,4
ZNF707	rs201250823	ms	Q96C28	159	R/Q	-0,744	0,024146	0,000	**15**	**15**
ZNF714	rs200293321	ms	Q96N38	489	A/D	0,569	0,024378	0,022	**26**	**13**
ZNF714	rs372317502	ms	-	384	L/F	0,569	0,024378	**0,862**	**26**	**21**
ZNF726	rs1568380835	ms	A6NNF4	501	I/T	-0,762	0,028404	0,025	**11**	0
ZNF727	rs748962952	stop	A8MUV8	126	Q/*	0,415	0,020093	**1,000**	**40**	0
ZNF761	rs200379651	ms	Q86XN6	611	C/Y	0,997	0,006851	**0,987**	**29**	8,7
ZNF763	rs191167657	stop	Q0D2J5	162	Q/*	-0,507	0,008832	**1,000**	**30**	**71**
ZNF77	rs767350646	ms	Q15935	512	R/S	-0,964	0,008910	**0,941**	**12**	0,2
ZNF773	rs112107510	ms	Q6PK81	107	V/M	-0,684	0,014006	0,075	**25**	109
ZNF792	rs200278064	ms	Q3KQV3	614	A/T	0,750	0,041908	**0,818**	**15**	**77**
ZNF8	rs758111446	ms	P17098	116	E/G	-0,690	0,047064	0,413	**22**	1,9
ZNF8	rs145477083	ms	-	234	C/R	-0,486	0,013676	**0,462**	**37**	134
ZNF808	rs779325682	ms	Q8N4W9	807	A/V	0,548	0,027883	0,009	**25**	1,5
ZNF808	rs149957912	ms	-	494	R/H	0,437	0,042813	0,041	**33**	227
ZNF814	rs190903410	ms	B7Z6K7	210	G/R	0,565	0,025339	**0,955**	**19**	164
ZNF814	rs774093902	ms	-	596	R/C	-0,398	0,022114	0,000	**46**	2,6
ZNF821	rs139528518	ms	O75541	65	T/M	-0,885	0,024777	**0,985**	**16**	**74**
ZNF839	rs760906633	ms	A8K0R7	429	R/H	0,586	0,033405	0,391	**27**	1,3
ZNF841	rs372086918	ms	Q6ZN19	69	V/A	0,508	0,049189	0,302	**17**	7,2
ZNF862	rs200663618	ms	O60290	387	R/Q	-0,586	0,014298	0,007	**19**	**20**
ZNF891	rs1566336581	stop	A8MT65	97	Q/*	-0,895	0,014582	**1,000**	**12**	0
ZNF99	rs772978635	ms	A8MXY4	316	E/G	0,797	0,048267	0,015	**16**	0,8
ZNG1F	9:41182447:T/C	ms	Q4V339	120	I/V	0,442	0,030192	**0,893**	**45**	N/A
ZNG1F	rs1396385081	ms	-	157	G/R	0,892	0,035412	**0,998**	**17**	1,4
ZP1	rs1156861968	ms	P60852	395	S/L	-0,419	0,047372	0,013	**24**	0,8
ZSCAN1	rs147014137	ms	Q8NBB4	103	P/S	-0,776	0,017570	**1,000**	**13**	**68**
ZSCAN26	rs16893892	ms	Q16670	59	Y/C	0,645	0,020842	**0,989**	**29**	**3934**
ZSWIM3	rs148673439	ms	Q96MP5	445	R/Q	-0,449	0,039612	0,001	**40**	5,7
ZSWIM4	rs1374935163	ms	Q9H7M6	442	G/D	-0,490	0,016327	0,052	**40**	2,3
ZSWIM4	rs774329836	ms	-	153	V/L	0,925	0,012076	0,135	**16**	0,8
ZSWIM8	rs373698947	ms	A7E2V4	847	R/H	-0,484	0,012556	0,201	**53**	5,1
ZZEF1	rs772878926	ms	O43149	1928	T/M	0,378	0,037554	0,089	**44**	3,4

Plasma protein levels with SomaScan multiplex aptamer assay were measured for 4,719 proteins, including ACE, in 35,559 Icelanders with genotype and phenotype information [Ferkingstad,

2021]. Authors (and deCODE Genetics) made available the full GWAS summary statistics for each protein from this study including ACE (https://download.decode.is/form/folder/proteomics) We excerpted these GWAS statistics for statistically significant (p value < 0.05) correlation of blood ACE levels with different mutations (beta value) and added Mean Allele Frequency (MAF) for each mostly missense mutations found in sequenced Iceland population and compared to MAF in dbSNP (mostly from TOPMED – with 264,690 participants). Those *ACE* mutations, which MAF in Iceland was more than 10-fold higher than in dbSNP cohort (i.e. preferential for Iceland), were highlighted with light magenta. Those mutations which MAF were 10-fold lower in Iceland were highlighted with light yellow. Some of the mutations in 2,388 list, that do not have rsID, were highlighted with yellow. If more than one mutation was found for given gene, the name of that gene was **bolded.**

Correlation of blood ACE levels with each variant were expressed as beta values. Very significant correlations (beta value more than 1.00 in absolute terms were bolded (**black**-positive correlation and **red**-negative correlation). Very low p values ($< 10^{-7}$) were marked with red.

PolyPhen-2: PolyPhen-2 (dbNSFP version 3.3a) annotation based on HumanVar database. This annotation should be used when evaluating rare alleles at loci potentially involved in complex phenotypes, dense mapping of regions identified by genome-wide association studies, and analysis of natural selection from sequence data. The annotation consists of score and categorical prediction. There are three possible predictions: **Probably damaging**, score≥**0.909**), **Possibly damaging**, **0.446**≤score≤**0.908**), Benign, score≤0.445). MAF for mutations in Iceland's population and from dbSNP were expressed per 100 000 subjects and marked as follows: >**1000,** **>**100, >**10**. Families of the genes were marked in the column names – with bold red letters.

Table S2. Annotation of the genes and variants with mutations in Table S1 (Iceland's list). Broad classification of the genes present in Table S2 was presented in **A**.
The number of variants per each gene was shown in **B**.

**A**	**Broad Classification**	**Combined Quantity**
1	Enzymes (Metabolic, DNA/RNA Processing)	520
2	Additional Metabolic & Enzymatic Functions	464
3	Signaling & Regulatory Proteins	250
4	Transcription Factors & Regulators	235
5	Receptors	156
6	Extracellular Matrix & Cell Adhesion	140
7	Complex Assembly & Scaffold Proteins	134
8	Transporters & Channels	111
9	Cytoskeletal & Structural Proteins	103
10	Nuclear Structure & Transport	47
11	Proteases (Non-metalloproteases)	38
12	Vesicular Trafficking & Protein Transport	36
13	Secreted Proteins	26
14	Mitochondrial Proteins	24
15	Immune-related Proteins	13
16	Chaperones & Protein Folding	13
17	Metalloproteases	12
18	Energy & ATPases	3
19	Unclassified / Others	64
	Grand Total	2388

**B**	**Proteins (Genes)**	**No. of variants**
1	*ACE*	8
2	*TTN*	7
3	*MRC2*	6
4	*MUC16*	6
5	*SCN4A*	6
6	*FTSJ3*	5
7	*AHNAK2*	4
8	*KIF16B*	4
9	*MUC5AC*	4
10	*PKD1*	4
11	*PLEC*	4
12	*TANC2*	4
13	*ABCA8*	3
14	*ABCA13*	3
15	*ABO*	3
16	*CDH3*	3
17	*CR1*	3
18	*CSMD1*	3
19	*DOCK8*	3
20	*FBN3*	3
21	*GOLGA8M*	3
22	*HRNR*	3

23	*KIF15*	3
24	*LRRC37A3*	3
25	*MAP3K19*	3
26	*MCM9*	3
27	*MN1*	3
28	*NCOR2*	3
29	*OBSCN*	3
30	*PLXNA3*	3
31	*POLN*	3
32	*RAB3GAP2*	3
33	*SLX4*	3
34	*SPEN*	3
35	*SPPL2C*	3
36	*SYNE2*	3
37	*TEX2*	3
38	*TKT*	3
39	*TNC*	3
40	*TRIM37*	3
41	*TRIO*	3
42	*USP17L2*	3
229 genes		2
1778 genes		1

Table S3. List of putative ACE secretases.

No.	Gene	Rationale for inclusion as putative ACE secretase	Known substrates / shedding activity	References
1	*ADAM9*	Transmembrane ADAM zinc endopeptidase with broad endothelial/vascular expression; known for shedding type I transmembrane proteins (e.g., analogous to APP, TNF-R); ideal for constitutive ACE1 release in circulation.	HB-EGF [1]<br>pro-HGF [2]<br>CD44 [3]<br>APP [4]	[5]
2	*ADAM10*	ADAM10 as a well known sheddase for several proteins, but effect on ACE shedding is controversial: negative in Allinson, 2004 and positive in Webers, 2024	APP [6]<br>Notch [7]<br>EGF, TGF-α [8]	[9, 10]
3	*ADAM28*	ADAM28 is a membrane-anchored metalloprotease that primarily cleaves insulin-like growth factor binding proteins and is implicated in processes like leukocyte migration and cancer progression	IGFBP-3 [11]	
4	*ADAM29*	ADAM29 a disintegrin and metalloproteinase domain-containing protein that functions in cell adhesion, migration, and proteolysis, and is implicated in processes like immune response and cancer progression.	-	
5	*ADAM33*	ADAM33 a membrane-anchored metalloprotease primarily expressed in smooth muscle and fibroblasts, best characterized as an asthma susceptibility gene that regulates airway remodeling and hyperresponsiveness through its role in cell adhesion, signaling, and proteolysis.	TNF-α [12]	
6	*ADAMTS5*	Low: Secreted aggrecanase; cartilage/vascular matrix. Degenerative intervertebral disc disease.	Aggrecan [13]<br>Versican [14]	
7	*ADAMTS7*	Low: Secreted ADAMTS; vascular proteoglycan cleavage in CVD; matrix-oriented; strongly associated with CAD and promotes atherosclerosis [15-16].	COMP [17-18]<br>TSP-1 [17]	[19]
8	*ADAMTS13*	Secreted vascular zinc metalloprotease cleaving von Willebrand factor multimers; plasma/endothelial presence could enable extracellular ACE1 access, but not cell-surface tethered.	von Willebrand factor (vWF) [20-21],<br>FVIII [22]	[23]
9	*ADAMTS14*	Low: Secreted ADAMTS; collagen processing.	Procollagen types I, II and III [24]	
10	*ADAMTS17*	Low: Secreted ADAMTS; developmental.	-	
11	*ADAMTS18*	Low: Secreted ADAMTS; neural.	-	
12	*AGTPBP1*	Very low: Cytosolic zinc carboxypeptidase.	Tubulin (α- and β-tubulin) [25]	
13	*ECE1*	Transmembrane zinc endopeptidase structurally akin to ACE1, highly expressed in endothelium; primarily processes big endothelin but could extend endoproteolytically to ACE1 stalk in vascular contexts.	Big Endothelin-1 [26]<br>Big Endothelin-2/3 [27]<br>Beta-amyloid (Aβ) [28]<br>Bradykinin [29]<br>Substance P [30]	
14	*ECEL1*	Possible: ECE-like transmembrane zinc endopeptidase in neural/vascular tissues; endoproteolytic activity on peptides suggests potential for transmembrane cleavage.	-	
15	*CAPN2*	-	FAK [31]<br>PKC [32]<br>p53 [33]<br>Calcineurin (PP2B) [34]	[35]
16	*CPD*	Very low: Membrane zinc carboxypeptidase; Golgi/plasma, exopeptidase.	Bovine pancreatic polypeptide [36]<br>Bradykinin [37]<br>Amyloid beta peptide (Aβ1-40) [38]	
17	*LVRN*	Lower: Membrane zinc aminopeptidase with some endopeptidase traits; placental/kidney expression; exopeptidase bias reduces fit for stalk-specific cut.		
18	*MEP1B*	Reasonable: GPI-anchored membrane zinc endopeptidase in kidney/epithelium; sheds cytokines/receptors (e.g., IL-18);	Collagen IV [39]	

		renal/endothelial co-localization supports role in localized ACE1 shedding.		
19	*MMP20*	Low: Secreted enamelysin; dental-specific.	Amelogenin (AMELX) [40]<br>Ameloblastin (AMBN) [41]<br>Collagen (type V) [42]	
20	*MMP21*	Secreted zinc endopeptidase; embryonic/cancer.	-	
21	*NPEPPS*	Very low: Cytosolic zinc aminopeptidase; intracellular, no surface access.	Tau protein [43]<br>α-Synuclein [43]	
22	*PEPD*	Very low: Cytosolic zinc dipeptidase.	Bradykinin and neurotensin [44]<br>Vasopressin, oxytocin, substance P, neuropeptide Y [45]	
23	*PCSK5*		uPA [46]	[47]
24	*PMPCA*	Very low: Mitochondrial processing peptidase; organelle-restricted.	Specific peptides [48]	
25	*PM20D1*	Very low: Secreted zinc peptidase; lipid metabolism.	N-acyl amino acids [49]	

References for Table S3:

1. Izumi Y, Hirata M, Hasuwa H, et al. A metalloprotease-disintegrin, MDC9/meltrin-gamma/ADAM9 and PKCdelta are involved in TPA-induced ectodomain shedding of membrane-anchored heparin-binding EGF-like growth factor. *EMBO J*. 1998;17:7260-72.
2. Guaiquil V, Swendeman S, Yoshida T, Chavala S, Campochiaro PA, Blobel CP. ADAM9 is involved in pathological retinal neovascularization. *Mol Cell Biol*. 2009;29:2694-703.
3. Nakamura H, Suenaga N, Taniwaki K, Matsuki H, Yonezawa K, Fujii M, Okada Y, Seiki M. Constitutive and induced CD44 shedding by ADAM-like proteases and membrane-type 1 matrix metalloproteinase. *Cancer Res*. 2004 ;64:876-82.
4. Hotoda N, Koike H, Sasagawa N, Ishiura S. A secreted form of human ADAM9 has an alpha-secretase activity for APP. *Biochem Biophys Res Commun*. 2002 ;293:800-5.
5. English WR, Corvol P, Murphy G. LPS activates ADAM9 dependent shedding of ACE from endothelial cells. *Biochem Biophys Res Commun*. 2012;421:70-5.
6. Lammich S, Kojro E, Postina R, et al. Constitutive and regulated alpha-secretase cleavage of Alzheimer's amyloid precursor protein by a disintegrin metalloprotease. *Proc Natl Acad Sci U S A*. 1999 30;96:3922-7.
7. van Tetering G, van Diest P, Verlaan I, van der Wall E, Kopan R, Vooijs M. Metalloprotease ADAM10 is required for Notch1 site 2 cleavage. *J Biol Chem*. 2009; 284:31018-27.
8. Choi SJ, Han JH, Roodman GD (2007). ADAM8: a novel osteoclast stimulating factor. *J Bone Mineral Research*. 2009; 22: 1490-1499.
9. Webers M, Yu Y, Eyll J, et al. The metalloproteinase ADAM10 sheds angiotensin-converting enzyme (ACE) from the pulmonary endothelium as a soluble, functionally active convertase. *FASEB J*. 2024;38:e70105.
10. Allinson TM, Parkin ET, Condon TP, et al. The role of ADAM10 and ADAM17 in the ectodomain shedding of angiotensin converting enzyme and the amyloid precursor protein. *Eur J Biochem*. 2004;271:2539-47.
11. Mitsui Y, Mochizuki S, Kodama T, et al. ADAM28 is overexpressed in human breast carcinomas: implications for carcinoma cell proliferation through cleavage of insulin-like growth factor binding protein-3. *Cancer Res*. 2006 ;66(20):9913-20.
12. Zou J, Zhu F, Liu J, et al. Catalytic activity of human ADAM33. *J Biol Chem*. 2004; 279:9818-30.
13. Glasson SS, Askew R, Sheppard B, et al. Deletion of active ADAMTS5 prevents cartilage degradation in a murine model of osteoarthritis. *Nature*. 2005;434:644-8.
14. Zhu Z, Liu H, Feng L, et al. Loss of ADAMTS5 promotes vascular calcification via versican/integrin β1/FAK signal. *Atherosclerosis*. 2025;404:119190.

15. Bayoglu B, Arslan C, Tel C, et al. Genetic variants rs1994016 and rs3825807 in ADAMTS7 affect its mRNA expression in atherosclerotic occlusive peripheral arterial disease. *J Clin Lab Anal*. 2018;32:e22174. doi: 10.1002/jcla.22174.
16. van Setten J, Isgum I, Smolonska J, et al. Genome-wide association study of coronary and aortic calcification implicates risk loci for coronary artery disease and myocardial infarction. Atherosclerosis. 2013;228:400–5.
17. Kessler T, Zhang L, Liu Z, et al. ADAMTS-7 inhibits re-endothelialization of injured arteries and promotes vascular remodeling through cleavage of thrombospondin-1. *Circulation*. 2015;131:1191-201.
18. Liu CJ, Kong W, Ilalov K, et al. ADAMTS-7: a metalloproteinase that directly binds to and degrades cartilage oligomeric matrix protein. *FASEB J*. 2006;20:988-90.
19. Mead TJ, Apte SS. ADAMTS proteins in human disorders. *Matrix Biol*. 2018;71-72:225-239.
20. Zheng X, Chung D, Takayama TK, Majerus EM, Sadler JE, Fujikawa K. Structure of von Willebrand factor-cleaving protease (ADAMTS13), a metalloprotease involved in thrombotic thrombocytopenic purpura. *J Biol Chem*. 2001;276:41059-63.
21. Dong JF, Moake JL, Nolasco L, et al. ADAMTS-13 rapidly cleaves newly secreted ultra-large von Willebrand factor multimers on the endothelial surface under flowing conditions. *Blood*. 2002;100:4033-9.
22. Cao W, Krishnaswamy S, Camire RM, Lenting PJ, Zheng XL. Factor VIII accelerates proteolytic cleavage of von Willebrand factor by ADAMTS13, *Proc. Natl. Acad. Sci. U.S.A*. 2008; 105) 7416-7421.
23. Pagliari MT, Lotta LA, de Haan HG, et al. (2016) Next-Generation Sequencing and In Vitro Expression Study of ADAMTS13 Single Nucleotide Variants in Deep Vein Thrombosis. *PLoS One*. 2016; 11: e0165665
24. Colige A, Vandenberghe I, Thiry M, et al. Cloning and characterization of ADAMTS-14, a novel ADAMTS displaying high homology with ADAMTS-2 and ADAMTS-3. *J Biol Chem*. 2002;277:5756-66.
25. Rogowski K, van Dijk J, Magiera MM, et al. A family of protein-deglutamylating enzymes associated with neurodegeneration. *Cell*. 2010;143:564-78.
26. Sahin U, Weskamp G, Kelly K, et al. Distinct roles for ADAM10 and ADAM17 in ectodomain shedding of six EGFR ligands. *J Cell Biol*. 2004;164:769-79.
27. Schmidt M, Kröger B, Jacob E, et al. Molecular characterization of human and bovine endothelin converting enzyme (ECE-1). *FEBS Lett*. 1994;356:238-43.
28. Eckman EA, Adams SK, Troendle FJ, et al. Regulation of steady-state beta-amyloid levels in the brain by neprilysin and endothelin-converting enzyme but not angiotensin-converting enzyme. *J Biol Chem*. 2006; 281:30471-8.
29. Hoang MV, Turner AJ. Novel activity of endothelin-converting enzyme: hydrolysis of bradykinin. *Biochem J*. 1997;327(Pt 1):23-6.
30. Johnson GD, Stevenson T, Ahn K. Hydrolysis of peptide hormones by endothelin-converting enzyme-1. A comparison with neprilysin. *J Biol Chem*. 1999;274:4053-8.
31. Hamadi A, Bouali M, Dontenwill M, Stoeckel H, Takeda K, Rondé P. Regulation of focal adhesion dynamics and disassembly by phosphorylation of FAK at tyrosine 397. *J Cell Sci*. 2005;118(Pt 19):4415-25.
32. Kishimoto A, Mikawa K, Hashimoto K, et al. Limited proteolysis of protein kinase C subspecies by calcium-dependent neutral protease (calpain). *J Biol Chem*. 1989; 264:4088-92.
33. Kubbutat MH, Vousden KH. Proteolytic cleavage of human p53 by calpain: a potential regulator of protein stability. *Mol Cell Biol*. 1997;17:460-8.
34. Wu HY, Tomizawa K, Oda Y, et al. Critical role of calpain-mediated cleavage of calcineurin in excitotoxic neurodegeneration. *J Biol Chem*. 2004;279:4929-40.
35. Balyasnikova IV, Karran EH, Albrecht RF 2nd, Danilov SM. Epitope-specific antibody-induced cleavage of angiotensin-converting enzyme from the cell surface. *Biochem J*. 2002;362(Pt 3):585-595.

36. Song L, Fricker LD. Purification and characterization of carboxypeptidase D, a novel carboxypeptidase E-like enzyme, from bovine pituitary. *J Biol Chem*. 1995;270:25007-13.
37. Dong W, Fricker LD, Day R. Carboxypeptidase D is a potential candidate to carry out redundant processing functions of carboxypeptidase E based on comparative distribution studies in the rat central nervous system. *Neuroscience*. 1999;89:1301-17.
38. Fricker L.D. *Carboxypeptidase D*. In: Handbook of Biologically Active Peptides, 2nd ed., 2013. (Academic Press).
39. Kruse MN, Becker C, Lottaz D, et al. Human meprin alpha and beta homo-oligomers: cleavage of basement membrane proteins and sensitivity to metalloprotease inhibitors. Biochem J. 2004;378(Pt 2):383-9.
40. Bartlett JD, Simmer JP, Xue J, Margolis HC, Moreno EC. Molecular cloning and mRNA tissue distribution of a novel matrix metalloproteinase isolated from porcine enamel organ. *Gene.* 1996;183(1-2):123-8.
41. Chun YH, Yamakoshi Y, Yamakoshi F, et al. Cleavage site specificity of MMP-20 for secretory-stage ameloblastin. *J Dent Res*. 2010;89:785-90.
42. Turk BE, Lee DH, Yamakoshi Y, et al. MMP-20 is predominately a tooth-specific enzyme with a deep catalytic pocket that hydrolyzes type V collagen. *Biochemistry*. 2006; 45:3863-74.
43. Sengupta S, Horowitz PM, Karsten SL, et al. Degradation of tau protein by puromycin-sensitive aminopeptidase in vitro. *Biochemistry*. 2006;45:15111-9.
44. Yoshimoto T, Kanatani A, Shimoda T, Inaoka T, Kokubo T, Tsuru D. Prolyl endopeptidase from Flavobacterium meningosepticum: cloning and sequencing of the enzyme gene. *J Biochem*. 1991;110:873-8.
45. Fülöp V, Böcskei Z, Polgár L. Prolyl oligopeptidase: an unusual beta-propeller domain regulates proteolysis. *Cell*. 1998;94:161-70.
46. Sun X, Essalmani R, Seidah NG, Prat A. The proprotein convertase PC5/6 is protective against intestinal tumorigenesis: in vivo mouse model. *Mol Cancer*. 2009;8:73.
47. Kammerer CM, Gouin N, Samollow PB, et al. Two quantitative trait loci affect ACE activities in Mexican-Americans. *Hypertension*. 2004;43:466-470.
48. Bohovych I, Chan SS, Khalimonchuk O. Mitochondrial protein quality control: the mechanisms guarding mitochondrial health. *Antioxid Redox Signal*. 2015;22:977-94.
49. Long JZ, Roche AM, Berdan CA, et al. Ablation of PM20D1 reveals N-acyl amino acid control of metabolism and nociception. *Proc. Natl. Acad. Sci. USA* 2018;115: E6937-45.

Table S4. Association of circulating ACE levels with rare coding variants identified in carriers of *ADAM9*, *ADAMTS13*, and *MMP21*.

ACE phenotypes in carriers of 10 different ***ADAM9*** mutations identified among more than 5,000 exome-sequenced individuals from the RSMU cohort.

	**Table S4A**	Mutations	**E100V**	**P151L**	**G311fs**	**E339fs**	**ZUX446**	**I79T**	**G597V**	**G515S**	**P483S**	**Q286R**	**R755X**						
	RSMU	*ACE* Genotype	DD	DD	II	II	ID	ID	ID	ID	ID	ID	ID	AA subs.	Beta value	P value	Poly-Phen2	ImpMAF /100 000	MAF dbSNP
	**Pts. with *ADAM9* mutation**	**ACE,%**	31,7	63,4	125,5	150,4	152,9	164,3	175,3	205,2	211,1	212,7	297,0						
		Pts.##	BPZ380	YJT604	PLI176	UFZ649	ZUX446	AQL326	MOY675	RRB463	SYY934	UCX372	EKM341						
#	Gene	rsID																	
1	*ABCA1*	rs138880920												K/N	0,395	0,043339	**0,986**	**46**	202
2	*BRME1*	rs769178284												S/Y	-0,430	0,031732	**0,788**	**40**	4,2
3	*HYAL4*	rs143985361												S/G	-0,628	0,012993	0,147	**19**	170
4	*NPIPA1*	rs539477828												D/E	0,647	0,030701	**0,930**	**26**	69
5	*SLC44A1*	rs148518626												T/A	-0,470	0,036172	0,042	**35**	**72**
6	*TIAM1*	rs16987932												Q/H	-0,905	0,006001	-	**17**	**5119**
7	*ZNF763*	rs191167657												Q/*	-0,507	0,008832	**1,000**	**30**	**71**

ACE phenotypes in carriers of 6 different ***ADAMTS13*** mutations identified among more than 5,000 exome-sequenced individuals from the RSMU cohort.

	**Table S4B**	Mutations	**P421**	**P421**	**P421**	**C242V**	**A250V**	**C322Y**	**P421**	**E1326fs**	**R1221fs**	**S275N**	**P421**						
	RSMU	*ACE* Genotype	ID	ID	II	ID	ID	ID	ID	II	ID	II	II	AA subs.	Beta value	P value	Poly-Phen2	ImpMAF /100 000	MAF dbSNP
	**Pts. with *ADAMTS13* mutation**	**ACE,%**	**38,5**	50,9	60,7	69,8	70,5	107,5	**129,9**	**139,7**	**150,5**	**159,7**	**272,4**						
		Pts.##	OXV503	ZYE311	WAT855	VVF043	EQT402	FJF443	BBW666	JFL102	QYL132	VBO150	MKY744						
No.	Gene	rsID																	
1	*ABCA1*	rs138880920												K/N	0,395	0,043339	**0,986**	**46**	202
2	*ADAMTS13*	rs145825553												R/C	0,394	2,447E-08	**0,994**	334	**45**
3	*ASAP3*	rs140136454												A/D	0,449	0,001145	-	**93**	527
4	*COL6A5*	rs182112053												R/W	-0,396	0,025719	0,003	**48**	136
5	*GAD1*	rs45566933												I/L	0,539	0,045535	0,062	**33**	**48**
6	*KPTN*	rs142867197												R/Q	0,455	0,006389	0,048	**66**	137
7	*KRT13*	rs144967807												V/I	0,569	0,012923	0,000	**41**	104
8	*MROH2B*	rs563729861												A/T	-0,637	0,049621	**0,990**	**26**	5,3
9	*ZNF821*	rs139528518												T/M	-0,885	0,024777	**0,985**	**16**	**74**

ACE phenotypes in carriers of 6 different ***MMP21*** mutations identified among more than 5,000 exome-sequenced individuals from the RSMU cohort.

	**Table S4C**	Mutations	**L546X**	**L176P**	**P124fs**	**N500fs**	**W205X**	**L546X**						
	RSMU	*ACE* Genotype	ID	DD	ID	ID	ID	II	AA subs.	Beta value	P value	Poly-Phen2	ImpMAF /100 000	MAF dbSNP
	**Pts. with *MMP21* mutation**	**ACE,%**	67,4	69,2	71,7	111,1	116,3	119,4						
		Pts.##	MVR453	SCC338	EVH293	NPQ143	OYH968	YEH357						
No.	Gene	rsID												
1	*CARD6*	rs148780446							S/L	0,875	0,011883	0,000	**17**	257
2	*PSRC1*	rs116496512							A/V	0,963	0,014683	**0,986**	**13**	177
3	*PTPRC*	rs140403368							D/N	-0,407	0,037750	0,000	**48**	**90**
4	*UMOD*	rs199835347							R/Q	0,771	0,019467	0,014	**10**	144

For each carrier, the table lists ACE concentration, *ACE* I/D genotype, the co-occurring variant (gene, rsID, amino-acid substitution), predicted functional impact (PolyPhen-2), mean allele frequencies (MAF), and effect size estimates (β) with P-values. Several variants show nominally significant associations with ACE levels, although interpretation is limited by the very small number of carriers. Those mutations which frequencies were 10-fold lower in Iceland than in dbSNP cohort were highlighted with light yellow. Cells highlighted in light green indicate heterozygous carriers of a given mutation.

Table S5. Mutations (215) that dramatically influenced blood ACE levels (Short "Iceland's list").

No.	Gene	rsID	Effect	UNIPROT	Protein position	AA subs.	Beta value	P value	Poly-Phen-2	ImpMAF /100 000	MAF dbSNP /100 000
1	*ABCA10*	rs773731325	ms	Q8WWZ4	1402	E/A	**-1,195**	0,014695	**0,942**	**12**	0
2	*ABCA8*	rs749671878	ms	O94911	996	M/I	**-1,088**	0,045480	0,003	**15**	0,4
3	*ABCF2*	rs1563617859	ms	Q9UG63	139	P/S	**-1,281**	0,030709	**0,993**	**14**	0
4	*ABCG4*	rs1320169801	ms	Q9H172	631	R/Q	**-1,157**	0,000822	**0,968**	**16**	0,4
5	***ACE***	rs750712925	ms	P12821	45	G/R	**-1,288**	6,826E-15	0,142	**82**	1,9
6	***ACE***	rs757694144	ms	-	482	R/P	**-1,349**	1,501E-07	0,246	**34**	0,4
7	***ACE***	rs3730025	ms	-	244	Y/C	**-1,217**	3,306E-291	**0,998**	**1,534**	924
8	*ADAMDEC1*	rs1207275267	ms	O15204	318	R/C	**1,294**	0,013085	**0,970**	**10**	0,4
9	*ADAMTSL5*	rs142558769	ms	Q6ZMM2	432	R/H	**1,494**	0,010884	**0,995**	**15**	411
10	*ADGRA2*	rs749263093	ms	Q96PE	386	T/I	**-1,077**	0,006264	**0,521**	**12**	0,1
11	*AFTPH*	rs3770740	ms	Q6ULP	301	E/K	**-1,384**	0,001161	0,006	**18**	**12 910**
12	*ALPP*	rs2981374	ms	P05187	501	P/H	**1,097**	0,012381	0,000	**17**	0,8
13	*ANKFY1*	rs1567972969	ms	Q9P2R3	15	L/H	**-1,160**	0,002731	**0,998**	**14**	0
14	*ANKS1A*	rs986675244	ms	Q92625	438	M/V	**-1,015**	0,050749	0,000	**13**	0,4
15	*ANXA4*	rs2228202	ms	P09525	137	S/R	**-1,320**	0,028467	**0,718**	**15**	**9 410**
16	*APCDD1L*	rs145873693	ms	Q8NCL9	330	V/M	**1,311**	0,002047	0,069	**21**	**67**
17	*APOB*	rs374473614	stop	P04114	725	Q/*	**1,016**	0,005874	**1,000**	**19**	0,4
18	*ARAP2*	rs1560721800	ms	Q8WZ64	190	T/A	**-1,043**	0,041357	0,001	**16**	0
19	*ARID5B*	rs199637139	ms	Q14865	901	T/M	**-1,574**	0,008946	0,295	**12**	2,0
20	*ASPM*	rs1557956556	ms	Q8IZT6	1117	M/V	**1,062**	0,041921	**0,970**	**19**	0,4
21	*ATRNL1*	rs1229952017	ms	Q5VV63	971	H/Y	**1,074**	0,003547	0,000	**12**	0,8
22	*BBIP1*	rs1473657985	ms	A8MTZ0	45	I/M	**1,050**	0,007087	**0,581**	**11**	0,8
23	*BCAS3*	rs771742994	ms	Q9H6U6	480	S/I	**-1,162**	2,188E-12	**0,991**	**88**	6,4
24	*BIRC7*	rs201155031	ms	Q96CA5	245	R/Q	**-1,306**	0,000911	0,000	**12**	1,9
25	*BMS1*	rs117641896	ms	Q14692	864	A/T	**1,062**	0,003955	0,054	**20**	566
26	*BOP1*	rs1160618575	ms	Q14137	673	P/R	**-1,512**	0,003735	0,015	**11**	0
27	*BPIFA2*	rs1075435	ms	Q96DR5	236	V/L	**-1,106**	0,033823	0,000	**15**	**8 747**
28	*BPTF*	rs748420257	ms	Q12830	240	N/S	**-1,662**	0,031778	0,043	**13**	0
29	*C8A*	rs142382705	ms	P07357	207	R/W	**-1,172**	0,046057	**0,999**	**10**	5,4
30	*CACNA1H*	rs774565789	ms	O95180	281	T/M	**1,508**	0,000317	**0,830**	**10**	2,3
31	*CAPZA3*	rs146799170	ms	Q96KX2	154	C/F	**-1,019**	0,009792	**0,998**	**13**	178
32	*CARD10*	rs1569161077	ms	Q9BWT7	1030	S/I	**1,304**	0,005090	**0,774**	**19**	0,6
33	*CASP4*	rs56008239	ms	P49662	344	R/Q	**-1,008**	0,004577	0,167	**16**	**23**
34	*CCR4*	rs1410395535	ms	P51679	89	V/M	**1,068**	0,000605	**0,999**	**21**	0,4
35	*CENPF*	rs775339410	ms	P49454	2591	K/E	**-1,463**	0,015151	0,010	**14**	0,2
36	*CEP290*	rs184018899	ms	O15078	557	R/H	**1,030**	0,048276	**0,946**	**10**	**68**
37	*CGN*	rs376782764	ms	Q9P2M7	211	R/W	**-1,060**	0,005845	**0,999**	**14**	3,4
38	*CHD6*	rs1345334517	ms	Q8TD26	2487	M/T	**-1,596**	0,023908	0,025	**12**	0,4
39	*CHRDL2*	rs141077727	ms	Q6WN34	15	A/T	**1,269**	0,001294	0,087	**16**	168
40	*CIT*	rs1204187421	ms	O14578	1910	I/M	**-1,115**	0,001211	**0,960**	**11**	0,2
41	*CLMN*	rs148836979	ms	Q96JQ2	158	P/A	**-1,077**	0,001091	**0,635**	**26**	**96**
42	*CNNM4*	rs561036245	ms	Q6P4Q7	122	N/I	**-1,037**	0,003385	0,261	**19**	9,8
43	*CNTN3*	rs1559690789	ms	Q9P232	655	G/R	**1,346**	0,000260	**0,999**	**10**	0
44	*CNTNAP3B*	rs1200081323	ms	Q96NU0	817	V/M	**1,319**	0,012235	0,085	**10**	1,3
45	*COPG2*	rs1174813614	ms	Q9UBF2	466	T/M	**1,005**	0,010779	**0,518**	**23**	3,0
46	*CPT1A*	rs1566348900	fs	P50416	569	F/X	**-1,527**	0,004625	**1,000**	**15**	0
47	*CRNN*	rs780465907	ms	Q9UBG3	363	S/R	**-1,140**	0,003788	**0,649**	**17**	0,3
48	*CSMD1*	rs571852185	ms	Q96PZ7	167	I/M	**-1,032**	0,015289	**0,845**	**10**	0,8
49	*CSN1S1*	rs201502829	ms	P47710	4	L/P	**-1,057**	0,007322	**0,568**	**13**	**15**
50	*DCTN1*	rs774489951	ms	Q14203	992	E/G	**1,005**	0,009554	0,242	**11**	0
51	*DNAH10*	12:123790015:T/C	ms	Q8IVF4	570	V/A	**-1,378**	0,008236	**0,571**	**10**	N/A
52	*DNAH8*	rs862432	ms	Q96JB1	2661	T/N	**1,587**	0,002190	0,012	**12**	**7 598**
53	*DOC2B*	rs548884378	ms	Q14184	4	R/Q	**-1,129**	0,019165	0,000	**14**	**60**
54	*DOP1B*	21:36263581:C/T	ms	Q9Y3R5	1784	S/L	**1,174**	0,024190	**0,461**	**11**	N/A
55	*DUSP8*	rs755470031	ms	Q13202	75	R/C	**-1,148**	0,000238	**0,846**	**10**	0,4
56	*DYRK1A*	21:37505403:G/A	ms	Q13627	445	D/N	**1,248**	0,038008	0,140	**11**	N/A

57	ELAPOR2	rs34772926	ms	A8MWY0	605	R/H	-1,099	0,034969	0,146	12	46
58	ELMO1	rs765065086	ms	Q92556	331	A/T	-1,249	0,037612	1,000	11	0
59	EMID1	rs201794555	ms	Q96A84	129	R/Q	-1,117	0,026225	0,263	16	1,1
60	EPHB3	rs779216599	ms	P54753	436	P/L	1,433	0,005975	0,548	13	0,2
61	ERAP2	rs34261036	ms	Q6P179	411	L/R	2,240	0,030061	0,980	13	352
62	ETV2	rs780706581	ms	O00321	112	A/T	-1,176	0,005721	0,193	21	15
63	**ETV5**	rs1467805143	ms	P41161	245	R/W	-1,061	0,007116	0,970	17	0,3
64	**ETV5**	rs1281944233	ms	-	482	P/L	1,242	0,017184	0,998	13	0,4
65	FAM118A	rs777058813	ms	Q9NWS6	85	R/W	-1,027	0,007808	0,397	14	11
66	FANCF	rs1055714341	ms	Q9NPI8	66	W/R	1,130	0,007984	0,694	10	0,7
67	FAT3	rs201524480	ms	Q8TDW7	1152	I/T	1,142	0,003821	0,479	12	29
68	FBXW10B	rs1201781215	ms	O95170	15	R/H	-1,111	0,002331	0,003	12	0,8
69	FCSK	rs764653879	stop	Q8N0W3	969	E/*	-1,058	0,032116	1,000	10	0,4
70	FGGY	rs199980192	ms	Q96C11	17	V/I	1,017	0,020776	1,000	11	108
71	FMO2	rs772086521	ms	P31512	439	L/F	1,258	0,004932	0,971	15	4,2
72	FNDC7	1:108730742:A/G	ms	Q5VTL7	565	I/V	1,349	0,025181	0,000	10	N/A
73	FRAS1	rs774824172	ms	Q86XX4	644	G/R	-1,211	0,009411	0,830	12	0,7
74	**FSIP2**	rs113773415	ms	Q5CZC0	3988	S/F	1,725	0,000945	0,000	12	63
75	**FSIP2**	rs111265848	ms	-	568	Y/F	1,725	0,000945	0,000	12	132
76	GBP3	rs139129340	ms	Q9H0R5	163	N/S	1,332	0,010678	0,074	12	99
77	GCKR	rs545938878	ms	Q14397	612	R/L	-1,565	0,000795	0,455	13	0,4
78	GNAT2	rs1189352767	ms	P19087	254	C/R	-1,066	0,037318	1,000	12	0,2
79	GOLGA2	rs16912752	ms	Q08379	456	M/V	1,206	0,021948	0,006	10	10 382
80	GOLGA8H	rs767587258	ms	P0CJ92	141	Q/E	-2,113	0,002525	0,003	16	0,8
81	GON4L	rs756015284	ms	Q3T8J9	1821	P/S	-1,091	0,007105	0,007	12	1,1
82	GP1BA	rs756370087	ms	P07359	222	P/A	1,948	0,011850	0,874	12	0,4
83	GP9	rs3796130	ms	P14770	156	A/T	1,198	0,046718	0,191	11	2 150
84	GRM3	rs776290250	ms	Q14832	102	D/Y	-1,100	0,034914	1,000	12	0,2
85	GSR	rs151187899	ms	P00390	289	V/A	1,016	0,000033	0,987	29	148
86	H2AC1	rs1159899764	ms	Q96QV6	63	I/V	-1,070	0,011965	0,000	11	1,4
87	HADHA	rs772164983	ms	P40939	354	F/S	1,133	0,014997	0,653	11	0
88	HAL	rs1565990642	ms	P42357	343	G/V	1,008	0,030083	0,999	11	0,1
89	HCRTR1	rs199680510	ms	O43613	197	R/L	-2,238	0,000139	0,000	11	4,2
90	HDAC4	rs745530862	ms	P56524	362	G/S	-1,438	0,002058	0,042	15	2,5
91	ICOSLG	rs759358911	ms	O75144	199	V/M	1,690	0,015019	0,915	13	3,2
92	IFNGR1	rs1887415	ms	P15260	467	L/P	2,081	0,000546	0,090	13	1 603
93	IPO4	rs747440936	ms	Q8TEX9	182	R/C	1,054	0,038898	0,870	12	0,7
94	IRF3	rs1568461744	ms	Q14653	101	H/D	-1,830	0,000084	0,841	11	0
95	ISX	rs7291048	ms	Q2M1V0	158	A/V	1,265	0,015182	0,191	13	1 181
96	JCAD	rs184216138	ms	Q9P266	1074	I/T	1,115	0,004479	0,039	12	56
97	KAT8	rs185459113	ms	Q9H7Z6-2	446	R/Q	-1,639	0,026243	0,000	11	89
98	KCNT1	rs753401695	ms	Q5JUK3	674	M/V	1,002	0,007134	0,001	14	3,0
99	KCNU1	rs775599941	ms	A8MYU2	1121	P/L	1,014	0,051835	0,000	12	4,2
100	KCTD1	rs192197412	ms	Q719H9	106	E/V	-1,059	0,000749	0,000	21	114
101	KIF14	rs771069928	ms	Q15058	1583	E/G	-1,025	0,009378	0,010	13	0
102	KLHL18	rs374972341	ms	O94889	292	A/T	-1,039	0,004474	0,997	18	45
103	KMT5A	rs1309240986	ms	Q9NQR1	37	P/S	-1,555	0,022263	0,621	14	0,4
104	LAMB1	rs766731970	ms	P07942	445	Y/C	-1,216	0,042208	0,989	10	0,4
105	LAMB3	rs747338921	ms	Q13751	907	A/D	-1,040	0,018506	0,791	12	0,7
106	LLGL1	rs761027899	ms	Q15334	329	E/K	-1,410	0,009989	0,001	11	2,3
107	LOXHD1	rs754654876	ms	Q8IVV2	1572	R/Q	1,369	0,001659	0,918	14	0,8
108	LRP8	rs139703435	ms	Q14114	725	M/R	-1,016	0,010003	0,246	10	31
109	LRRN2	rs374611937	ms	O75325	577	R/Q	-1,001	0,002439	0,001	22	7,6
110	MAGEL2	rs1566783726	ms	Q9UJ55	944	S/N	-1,094	0,019026	0,020	11	0,9
111	MAP3K1	rs764525244	ms	Q13233	504	E/K	1,291	0,031982	0,447	14	4,2
112	MAPK8IP2	rs1569064809	ms	Q13387	421	A/T	-1,040	0,027579	0,000	18	0,7
113	MBD1	rs142015383	ms	Q9UIS9	315	E/K	1,077	0,001920	0,712	15	41
114	MCEE	rs111033538	stop	Q96PE7	47	R/*	1,215	0,009228	1,000	11	30
115	MCF2L2	rs773833576	ms	Q86YR7	376	K/E	-1,063	0,012535	0,215	12	0,4
116	MKI67	rs1565001712	stop	P46013	2225	Q/*	1,181	0,011340	1,000	16	0,1
117	MMP21	rs554501102	ms	O75900	97	A/V	-1,789	0,002314	0,392	11	60

118	*MRPL20*	rs775815488	ms	Q9BYC9	126	E/K	1,240	0,006840	0,996	19	1,1
119	*MTCL1*	rs147496732	ms	Q9Y4B5	728	R/C	1,525	0,003417	1,000	12	2,3
120	*MYO1F*	rs557720157	ms	O00160	658	R/W	-1,000	0,009984	0,846	12	0,4
121	*MYOM1*	rs866006864	ms	P52179	229	A/V	-1,019	0,007773	0,000	12	6,8
122	*NAA35*	rs1564301637	ms	Q5VZE5	219	R/G	-1,708	0,001180	0,150	10	0,1
123	*NAGPA*	rs1567142778	ms	Q9UK23	173	R/C	-1,106	0,000437	0,977	19	0,8
124	***NCOR2***	rs745312035	ms	Q9Y618	2483	A/T	-1,147	0,000139	0,036	24	12
125	***NCOR2***	rs761145072	ms	-	2423	R/G	1,079	0,003446	0,999	24	0,7
126	*NFILZ*	rs755954670	ms	A0A5F9ZHS7	153	R/Q	1,066	0,012246	0,014	18	0,7
127	*NOVA1*	14:26448097:T/G	ms	P51513	462	E/D	-1,074	0,011270	0,149	11	N/A
128	*NXNL2*	rs751445572	stop	Q5VZ03	142	Q/*	1,212	0,004748	1,000	14	0,7
129	*OCSTAMP*	rs1568897913	stop	Q9BR26	243	W/*	1,173	0,024410	1,000	10	0
130	*OR11H1*	rs201166644	ms	Q8NG94	52	V/F	1,324	0,027400	0,195	11	133
131	*OR2A5*	rs201829543	ms	Q96R48	223	A/G	-1,014	0,010106	0,001	15	150
132	*OR2M5*	rs142232947	ms	A3KFT3	80	K/T	-1,026	0,026984	0,885	10	48
133	*OR51C1P*	rs141670434	ms	A0A3B3IT45	122	R/H	-1,053	0,039579	0,125	12	105
134	*OR52H1*	rs761950199	ms	Q8NGJ2	263	A/T	1,162	0,000300	0,179	13	1,1
135	*OR5L2*	rs143953099	ms	Q8NGL0	254	T/I	1,505	0,012528	0,984	13	49
136	*OSBPL10*	rs1559483397	ms	Q9BXB5	181	A/V	1,191	0,010324	0,015	10	0
137	*PCDHGC4*	rs1562119204	ms	Q9Y5F7	747	N/S	1,417	0,002367	0,060	11	0,8
138	*PHRF1*	rs202005033	ms	Q9P1Y6	744	H/R	1,327	0,003328	0,234	19	48
139	*PIGP*	rs2276231	ms	P57054	112	R/S	-1,306	0,029909	0,255	10	2 302
140	*PILRA*	rs1563114512	ms	Q9UKJ1	101	W/C	-1,492	0,013181	0,998	15	0
141	***PLXNA1***	rs370434411	ms	-	443	R/Q	1,060	0,002239	0,009	18	8,3
142	***PLXNA1***	rs147504334	ms	Q9UIW2	1635	S/G	1,029	0,048631	0,373	12	127
143	*POTEF*	rs755588157	ms	A5A3E0	883	R/W	1,136	0,014870	0,431	13	0,4
145	*PRKCA*	rs748202197	ms	P17252	381	V/M	1,074	0,001778	0,920	12	1,5
144	*PRRC2B*	rs908183601	ms	Q5JSZ5	1106	S/R	1,112	0,018219	0,200	14	1,3
146	*PSD4*	rs1558893290	ms	Q8NDX1	637	R/G	1,206	0,014207	0,018	12	0,4
147	*PTPN13*	rs772350639	ms	Q12923	1893	L/M	1,182	0,020599	0,961	22	1,5
148	*RAB11FIP5*	rs114626386	ms	Q9BXF6	933	E/K	-1,078	0,000999	0,000	13	745
149	*RILP*	rs34982553	ms	Q96NA2	281	R/Q	-1,114	0,041984	0,968	11	897
150	*RMDN2*	rs142128542	ms	Q96LZ7	226	D/N	-1,132	0,015245	0,020	12	18
151	*RNF145*	rs545563752	ms	Q96MT1	536	I/T	1,006	0,045136	0,307	10	0,2
152	*RNF169*	rs759737139	ms	Q8NCN4	119	R/H	-2,509	0,000674	0,837	12	75
153	*RNF213*	rs1568136656	ms	Q63HN8	3911	A/D	-1,633	0,000401	0,006	11	0
154	*ROGDI*	rs773589345	ms	Q9GZN7	187	D/H	1,212	0,023385	0,984	12	1,9
155	*SANBR*	rs746911974	ms	Q6NSI8	269	N/K	-1,057	0,006760	0,834	12	0
156	*SBF2*	rs775360425	ms	Q86WG5	1578	K/R	1,109	0,033647	0,005	12	1,5
157	*SBNO2*	rs200703494	ms	Q9Y2G9	142	P/S	-1,299	0,026765	0,005	18	7,2
158	*SCAMP4*	rs201067147	ms	Q969E2	181	G/R	-1,149	0,013593	0,837	11	1,5
159	*SEMA3F*	rs757110934	ms	Q13275	628	P/A	-1,202	0,016683	0,015	11	0,7
160	*SH2D3C*	rs61761896	ms	Q8N5H7	443	A/S	-1,026	0,000266	0,003	24	29
161	*SLA*	rs372398209	ms	Q13239	202	R/K	-1,056	0,006720	0,000	16	2,6
162	*SLC12A1*	rs6493311	stop	Q13621	538	Y/*	-1,023	0,051200	1,000	11	39 492
163	*SLC34A3*	rs202109348	ms	Q8N130	307	L/M	1,617	0,002139	0,223	13	54
164	*SLC6A17*	rs775258138	ms	Q9H1V8	483	T/M	-1,089	0,027689	0,999	15	0,4
165	*SLC6A8*	rs782208622	ms	P48029	274	V/M	1,340	0,026390	0,915	18	6,4
166	*SMARCC1*	rs764145318	ms	Q92922	744	A/G	-1,039	0,004475	0,660	18	11
167	*SMARCD2*	rs1567759892	ms	Q92925	493	R/T	1,309	0,009592	0,990	12	0
168	*SMG7*	rs1239743198	ms	Q92540	698	P/L	-1,008	0,003779	0,076	17	0,1
168	*SNRNP35*	rs142113918	ms	Q16560	216	P/L	-1,024	0,015954	0,000	18	28
170	*SORCS3*	10:105157142:C/G	ms	Q9UPU3	496	A/G	-1,301	0,001605	0,640	14	N/A
171	*SPDYE2B*	rs1791901438	ms	A6NHP3	246	I/V	1,159	0,005486	0,332	18	0
172	*SPEG*	rs373080805	ms	Q15772	2131	F/L	1,171	0,012117	0,095	10	2,6
173	*SPRR2D*	rs1846857	ms	P22532	20	T/A	1,148	0,036101	0,000	25	6190
174	*SPTBN4*	rs753897679	ms	Q9H254	163	R/C	-1,152	0,000476	1,000	13	0,8
175	*SPTBN5*	rs756377333	ms	Q9NRC6	372	R/Q	-1,033	0,026587	0,018	16	1,1
176	*ST3GAL4*	rs765192401	ms	Q11206	120	C/Y	-1,186	0,005391	1,000	11	0,8
177	*STK10*	rs753084325	ms	O94804	918	R/Q	1,245	0,006678	0,860	11	1,9
178	*STK11*	rs1169900277	ms	A0AAQ5BHW9	573	R/H	1,068	0,040474	0,033	13	0,4

179	*SUSD2*	rs766473126	ms	Q9UGT4	576	P/S	**1,021**	0,005562	0,231	**10**	0,8
180	*SYT4*	rs149020215	ms	Q9H2B2	384	R/Q	**1,084**	0,001435	0,009	**15**	**15**
181	*TANC1*	2:159219263:G/A	ms	Q9C0D5	1135	G/D	**-1,032**	0,015280	0,186	**10**	N/A
182	*TBC1D16*	rs1020548320	ms	Q8TBP0	55	L/R	**-1,746**	0,014260	0,161	**17**	7,1
183	*TBC1D32*	rs200973240	ms	Q96NH3	770	R/G	**1,184**	0,049320	0,021	**10**	**21**
184	*TGFBI*	rs201158209	ms	Q15582	354	N/S	**-1,230**	0,041089	0,261	**11**	**24**
185	*THEMIS*	rs373934545	ms	Q8N1K5	251	E/Q	**-1,069**	0,003751	**0,990**	**11**	2,6
186	*TLR3*	rs73025939	ms	O15455	643	R/C	**1,422**	0,00004264	**0,985**	**11**	158
187	*TMEM161B*	rs547282299	ms	Q8NDZ6	443	P/L	**-1,017**	0,026711	0,000	**14**	**17**
188	*TMEM259*	rs1568399471	fs	Q4ZIN3	427	S/X	**-1,299**	0,026843	**1,000**	**15**	0
189	*TMF1*	rs768748751	ms	P82094	284	S/L	**1,060**	0,042153	0,170	**11**	0
190	*TNNT2*	rs367785431	ms	P45379	296	R/C	**-1,161**	0,000845	**0,924**	**14**	1,9
191	*TNRC18*	rs200928367	ms	O15417	1549	G/S	**1,262**	0,003029	0,000	**13**	**46**
192	*TNRC6B*	rs577376433	ms	Q9UPQ9	556	P/S	**1,102**	0,000748	**0,986**	**19**	3,8
193	*TOP2A*	rs746667611	ms	P11388	1118	V/I	**-1,173**	0,008711	0,066	**17**	1,5
194	*TRAP1*	rs148549350	ms	Q12931	469	R/C	**1,098**	0,005334	**0,999**	**15**	**35**
195	*TRIM22*	rs780626396	ms	Q8IYM9	74	L/R	**1,771**	0,003284	**0,995**	**13**	**26**
196	*TRIO*	rs201650861	ms	O75962	507	S/L	**-1,500**	0,041992	0,000	**15**	1,5
197	*TRIP6*	rs1310784349	ms	Q15654	374	G/D	**-1,422**	0,018084	**0,958**	**11**	0,8
198	*TRPC3*	rs765481480	ms	Q13507	108	S/N	**1,014**	0,000277	0,007	**16**	0
199	*UCHL5*	rs146351256	ms	Q9Y5K5	321	A/T	**1,088**	0,005784	0,000	**14**	**38**
200	*UCN3*	rs782142877	ms	Q969E3	94	R/W	**1,168**	0,012078	**0,997**	**12**	1,9
201	*UGT2B17*	rs186138322	ms	O75795	480	A/T	**-1,453**	0,000228	**0,921**	**15**	**59**
202	*USF3*	rs745332813	ms	Q68DE3	196	V/L	**-1,454**	0,005321	0,048	**16**	2,5
203	*USP28*	rs142728209	ms	Q96RU2	631	R/I	**1,220**	0,042863	**0,698**	**11**	**42**
204	*USP29*	rs144679292	ms	Q9HBJ7	784	L/P	**-1,131**	0,004096	0,122	**11**	153
205	*UTP15*	rs776437309	ms	Q8TED0	288	Y/D	**1,216**	0,043359	**0,973**	**10**	0
206	*VPS13B*	rs61754112	ms	Q7Z7G8	1452	L/F	**1,137**	0,014719	0,015	**15**	2,6
207	*WASF3*	rs375670514	ms	Q9UPY6	31	N/S	**-1,365**	0,019915	**0,934**	**11**	1,5
208	*ZBTB38*	rs201503155	ms	Q8NAP3	588	N/I	**1,113**	0,017012	0,225	**11**	8,3
209	*ZBTB48*	rs141447787	ms	P10074	273	A/V	**-1,162**	0,000797	0,000	**18**	5,3
210	*ZFYVE1*	rs1433679547	ms	Q9HBF4	756	P/H	**-1,317**	0,011957	**1,000**	**12**	0
211	*ZKSCAN2*	rs1567354833	stop	Q63HK3	185	R/*	**-2,018**	0,025704	**1,000**	**12**	0,1
212	*ZNF189*	rs200682925	ms	O75820	622	T/I	**-1,004**	0,000589	0,031	**18**	0,8
213	*ZNF395*	rs1226932987	ms	Q9H8N7	2	A/V	**1,555**	0,009779	0,000	**12**	0
214	*ZNF408*	rs769357381	ms	Q9H9D4	442	C/R	**1,523**	0,038991	**0,999**	**12**	1,1
215	*ZNF638*	rs137918800	ms	Q14966	897	L/F	**1,588**	0,031441	0,044	**13**	1,9

We took data from Table S1 and excerpted only variants that dramatically changed blood ACE levels (beta value > 1.000 in absolute values). Those *ACE* mutations, which frequency in Iceland was more than 10-fold higher than in dbSNP cohort (i.e. preferential for Iceland), were highlighted with light magenta. Those mutations which frequencies were 10-fold lower in Iceland than in dbSNP cohort were highlighted with light yellow. Polyphen-2: PolyPhen-2 (dbNSFP version 3.3a) annotation based on HumanVar database and consists of score and categorical prediction. There are three possible predictions: Probably damaging, score≥0.909; Possibly damaging, 0.446≤score≤0.908; Benign, score≤0.445).

Table S7. Mutations from Iceland's list in 129 control samples with normal range of blood ACE.

							MAF / 100 000		RSMU	RSMU	RGNKC	RUSEQ	RSMU
	**Controls**								45	31	19	19	15
No.	Gene	rsID	AA substitution	Beta value	P value	PolyPhen-2 Score	Iceland	dbSNP	4657	4657	490	12976	637
1	*ACSS3*	rs61745251	H/Y	0,508	0,00500	0,001	55	5548	2	2			
2	***ADAMTS7***	rs61754850	p.P126L	0,539	0,02540	0,028	24	192				1	
3	*ADAMTSL5*	rs142558769	R/H	1,494	0,01088	0,995	15	411	2				
4	*AFTPH*	rs3770740	p.E301K/X	-1,384	0,00116	0,006	18	12911	2	2	2		
5	*AGXT*	rs121908529	G/R	-0,701	0,033683	0,986	18	53					1
6	*AIP*	rs145047094	R/H	-0,677	0,04043	0,615	23	171	1				
7	*ALDH3B2*	rs144564033	A/T	0,510	0,03713	0,981	37	14	2				
8	*ALDH9A1*	rs1065756	S/T	0,514	0,026897	0,000	30	2041		2			
9	*ANXA4*	rs2228202	S/R	-1,320	0,028467	0,718	15	9410		1			
10	*AP3D1*	rs25673	p.I1134L	-0,537	0,03839	0,007	26	11762			3	3	
11	*APCDD1*	rs200939423	R/H	-0,565	0,02924	0,048	30	13	1				
12	***APOA5***	rs747958115	p.R211L	0,961	0,02450	0,238	15	3,8			1		
13	*APOA5*	rs201201147	H/L	-0,790	0,01673	0,771	12	15	1				
14	*ATAD5*	rs112921454	E/K	0,419	0,03725	0,162	45	95	1				
15	*ATAT1*	rs150614928	G/V	0,453	0,03007	0,151	57	678	2				
16	*ATPAF1*	rs757491853	p.E245Q	-0,451	0,00760	0,937	65	1,4			2		
17	*ATXN2*	rs7969300	S/N	-0,726	0,00921	0,036	37	8372	1	1			
18	***BCKDHA***	rs11549936	p.P39K	-0,662	0,00445	0,000	16	8739			2	5	
19	*BMS1*	rs117641896	A/T	1,062	0,00396	0,054	20	566	6	5	3		
20	***BSN***	rs148125466	A/T	-0,397	0,048019	0,958	43	289		1			
21	*C12orf43*	rs141346965	A/V	0,561	0,001704	0,983	46	40					1
22	*C2CD2L*	rs140064567	E/K	0,434	0,00224	0,111	105	92	1				
23	*CADM4*	rs112876885	Y/H	-0,489	0,010187	0,802	37	536		1			
24	*CAPZA3*	rs146799170	C/F	-1,019	0,009792	0,998	13	178		1			
25	*CCDC181*	rs137855652	p.E167G	-0,398	0,04954	0,544	36	49		1	1		
26	*CDCP2*	rs150513591	p.P150S	-0,970	0,02753	0,000	11	19			1		
27	***CDH23***	rs74145660	D/E	-0,376	0,03068	0,057	50	882	1				
28	*CETN1*	rs115842359	A/T	0,654	0,04546	0,131	16	272	1				
29	*CFAP46*	rs140205673	A/T	0,699	0,00159	0,000	34	205	1				
30	*CFAP92*	rs187885262	D/G	0,359	0,045027	0,646	46	221	1				
31	*CHAT*	rs3810948	D/E	-0,964	0,04013	0,000	17	3232	2	2	1		3
32	*CHTF18*	rs200765427	D/N	-0,526	0,00479	0,049	51	320	1				
33	*CLCA4*	rs199643661	A/V	-0,598	0,01246	0,127	27	92	1				2
34	***CNTNAP3B***	rs1191153640	p.D838Y	-0,801	0,06177	0,994	11	0,7			1		
35	*DIAPH3*	rs200189161	p.R1042H	-0,547	0,00734	0,975	46	36			1		
36	*DMWD*	rs766488376	S/L	0,583	0,03527	0,000	31	20	1				
37	*DNAH8*	rs862432	p.T2661N/S	1,586	0,00219	0,012	12	7598				1	
38	*EDNRA*	rs192190120	I/V	-0,367	0,03471	0,003	44	46	1	1			1
39	***ENGASE***	rs199859024	V/I	-0,609	0,026188	0,038	28	9,4		1			
40	*ERAP2*	rs34261036	L/R	2,240	0,030061	0,980	13	352		1			2
41	*FAM83F*	rs149665361	p.R334C	-0,580	0,03618	0,924	13	87		1	1		
42	*FLNA*	rs187029309	p.S1991L	0,655	0,00216	0,966	28	216		1		1	1
43	*FLVCR2*	rs199824003	R/Q	-0,710	0,017058	0,025	11	2,6		1			
44	*FMNL2*	rs189416564	L/V	0,755	0,020482	0,578	29	152	1				
45	*FOXD4L4*	rs1256582592	R/H	-0,841	0,02393	1,000	14	76	2				
46	*GBGT1*	rs117595304	Y/C	-0,363	1,22E-09	0,817	457	311					1
47	***GHR***	rs6182	C/F	-0,356	0,00083	0,078	113	1604	2				1
48	***GHR***	rs6184	P/T	-0,356	0,00083	0,071	113	1602	2				1
49	*GNL1*	rs142398523	E/K	0,453	0,03006	0,235	57	534	2				
50	***GOLGA8M***	rs564738040	E/Q	0,670	4,2E-05	0,000	59	195		1			
51	*GP9*	rs3796130	A/T	1,198	0,04672	0,191	11	2150	3	1		1	1
52	*GPC1*	rs137923533	p.K528T	0,654	0,03754	0,070	16	32			1		
53	*GPD1L*	rs72552293	I/V	-0,392	0,04661	0,000	40	91	1	1			1
54	*GRIP2*	rs188992337	T/I	0,469	0,01787	0,046	47	234	4		1		
55	*GRK7*	rs34429284	R/H	0,616	0,00835	0,299	27	283	1				
56	*HLCS*	rs191115811	K/M	0,752	0,04122	0,710	21	72	1				
57	*HMGXB3*	rs374393844	p.T1169M	0,622	0,04507	0,009	17	28				1	
58	*HTR7*	rs114969659	P/L	0,901	0,01455	0,026	10	180	1				
59	*IBTK*	rs149695576	p.N1168S	0,484	0,00975	0,005	37	22				1	
60	*IFNGR1*	rs1887415	L/P	2,081	0,00055	0,090	13	1603	2	1	1		1
61	*IGF2R*	rs8191808	p.L817V	-0,496	0,01946	0,999	32	559			1		

62	*INPPL1*	rs17847215	K/N	-0,747	0,01761	0,004	19	1218	1	1			
63	*ISX*	rs7291048	p.A158V	1,265	0,01518	0,191	13	1181		1	2		
64	*JCAD*	rs184216138	I/T	1,115	0,004479	0,039	12	56		1			
65	*KATNIP*	rs750193636	p.D1036N	0,360	0,04081	0,876	29	7,6			1		
66	*KCTD1*	rs192197412	p.E106V	-1,059	0,00075	0,000	21	114			1		
67	*KDM4E*	rs182574403	P/L	-0,434	0,03743	0,998	49	38	1	1			
68	***KIF16B***	rs192942360	G/R	-0,953	0,025066	0,999	11	15		2			
69	*KIF26A*	rs774219038	R/H	-0,529	0,042261	0,868	26	8,3					1
70	***KLHDC4***	rs74931760	T/A	0,898	0,03491	0,009	13	1170	1		1		
71	***KLK14***	rs201296079	D/G	-0,762	0,02821	1,000	13	230	1				1
72	*KMT5A*	rs1309240986	p.P37S	-1,555	0,02226	0,624	14	0,4			1		
73	*LETM1*	rs115233072	K/R	-0,970	0,00080	0,986	20	1712	1				1
74	*LINGO4*	rs61746299	T/S	0,464	0,047103	0,089	43	7045					1
75	***MAP3K19***	rs3905317	E/G	-0,475	0,01272	0,826	56	2713	3	6	1	1	1
76	***MAP3K19***	rs1112542	E/Q	-0,475	0,01272	0,089	58	6756	3	6	1		1
77	*MLH3*	rs61752722	M/V	0,546	0,03612	0,000	31	182	2				
78	***MMP20***	rs17099008	I/L	-0,440	0,00032	0,176	96	1045	2	1			1
79	***MMP20***	rs61730849	T/I	-0,591	0,04988	0,991	26	99	4				
80	***NAA25***	rs12231744	K/R	-0,574	0,03966	0,001	38	7071	2	2			
81	*NAALADL1*	rs147209948	A/V	-0,949	0,02560	0,006	16	220	1				
82	*NMUR2*	rs4958535	p.S298I	-0,502	0,00986	0,011	38	14705			6	4	
83	*NOL11*	rs2291284	V/A	-0,672	0,00006	0,061	75	210	4	1			1
84	*NPAP1*	rs148062269	N/S	-0,444	0,05097	0,497	41	6,4	2				
85	*NPY4R*	rs782151881	p.P23L	-0,776	0,00995	0,000	11	0,7				1	
86	*OPN1LW*	rs148583295	I/T	0,510	0,042997	0,000	10	301		1			
87	*OR2AG1*	rs2659879	p.R299W	0,471	0,01304	0,457	45	24862			8	4	
88	*OR7D2*	rs77836061	M/L	0,398	0,03107	0,062	47	353	1	1			
89	***OSBPL10***	rs146089422	R/Q	0,406	0,00169	0,015	114	230	1				
90	*OSGIN1*	rs147230915	Q/R	0,390	0,04009	0,003	47	76	1	1			
91	*PCDHB15*	rs61745117	p.T508A	0,509	0,04294	0,002	21	252				1	
92	*PIGP*	rs2276231	R/S	-1,306	0,029909	0,255	10	2302		1			
93	*PINLYP*	rs193020817	T/N	-0,367	0,02328	0,899	61	115	1				
94	***PLXNA1***	rs147504334	S/G	1,029	0,04863	0,373	12	127	1	1	1		
95	*POLR1H*	rs17187658	Q/H	0,465	0,02317	0,384	55	1909	2	1			
96	*PON1*	rs141948033	N/D	0,597	0,022134	0,000	32	151					1
97	*POTEG*	rs201401586	R/T	0,425	0,05021	0,984	46	10	2				
98	*PPP1R13B*	rs140395624	p.I170V	-0,600	0,01428	0,000	36	376			1		
99	*PPP1R15A*	rs557806	p.R251Q	-0,471	0,02335	0,030	51	31678			1	1	
100	*PROM2*	rs147967943	p.F790L	-0,664	0,00031	0,143	51	110			1	1	1
101	*PRR12*	rs376580810	A/V	-0,412	0,011867	0,000	44	77		1			
102	*PTPRS*	rs4807697	C/R	0,698	0,04275	0,000	17	118	48	32	19	8	15
103	***RBM12B***	rs202226883	R/C	-0,520	0,01933	0,000	21	128	1				1
104	*RETSAT*	rs139043592	V/M	-0,361	0,01201	0,020	78	1145	6	3			1
105	*RNF39*	rs139005614	R/G	0,474	0,01809	0,132	40	393	1		1		
106	*RNH1*	rs150334174	S/L	0,698	0,035384	0,210	15	34		1			
107	*RRP12*	rs776080417	T/M	0,545	0,04288	0,883	18	1,5	1				
108	*RYR1*	rs138874610	D/N	-0,489	0,00802	1,000	67	59	1				
109	***SCARF1***	rs4790250	p.G667S	-0,964	0,00536	0,010	22	8191	48	32	19	9	15
110	***SCARF1***	rs3760460	p,G748V	-0,964	0,00536	0,919	21	4493			1		
111	*SCML4*	rs142985964	G158D	0,420	0,01870	0,987	52	170		1	2		
112	***SCN4A***	rs201148948	P/S	-0,959	0,00065	0,100	27	190	1				
113	*SETD1B*	rs751321721	P/L	0,450	0,030122	0,742	32	65					1
114	*SH3PXD2A*	rs199610107	E/G	0,594	0,040126	0,222	26	54					1
115	***SLX4***	rs114472821	p.P975L	0,686	0,04841	0,000	14	704				1	
116	***SPDEF***	rs2233639	A/T	0,474	0,04229	0,007	41	7438	3	3		2	1
117	***SPDEF***	rs2233637	P/R	0,474	0,04229	0,000	41	7333	3	3		2	1
118	***SPEG***	rs779078554	A/V	-0,854	0,014106	0,494	13	1,5		1			
119	*SPRR2D*	rs1846857	T/A	1,148	0,03610	0,000	25	6190	3	7	1	1	1
120	*SSH3*	rs151267958	R/Q	-0,677	0,04044	0,003	23	207	1		1		
121	*SUSD3*	rs149456736	Q/K	-0,451	0,03396	0,007	29	226	1			1	
122	*SYNGR2*	rs11557900	L/V	-0,748	0,034856	0,007	18	411		1			
123	***TEX2***	rs150175049	D/N	-0,868	0,00103	0,000	31	119	1				
124	***TM6SF2***	rs201189528	N/K	0,364	0,03091	0,122	54	83	1	2			
125	***TNRC6A***	rs149360617	T/A	-0,727	0,02638	0,005	14	61	1				
126	*TOB2*	rs148432877	I/V	-0,635	0,02181	0,567	20	65	1	1			
127	*TRIM40*	rs113286964	P/H	0,590	0,00804	0,884	51	956	2	1			
128	***TTLL2***	rs200952819	I/F	0,381	0,02837	0,961	42	6,0	1	1			
129	*TUBGCP4*	rs191224065	V/L	-0,525	0,05092	0,007	26	78	1				
130	*UGT2B10*	rs1976666	P/A	0,490	0,00694	0,941	58	857	1	3			

131	***USP17L2***	rs200055041	C/Y	-0,439	0,00721	**0,917**	**68**	179	1	1				
132	*USP29*	rs144679292	L/P	**-1,131**	0,00410	0,122	**11**	153	1					
133	*VLDLR*	rs6149	V/I	0,724	0,00458	0,042	**31**	**6001**	1	1		1		
134	*WFS1*	rs1805070	I/V	0,533	0,01343	0,014	**48**	325	1	2	1			
135	***ZC3H13***	rs144881601	S/N	-0,564	0,00948	0,000	**33**	170	1					
136	*ZNF213*	rs377090397	p.R309Q	0,381	0,03990	0,018	**42**	**36**			1			
137	*ZNF592*	rs151234929	P/L	0,641	0,051305	0,441	**19**	**29**		1				
138	*ZNF792*	rs200278064	A/T	0,750	0,04191	**0,818**	**15**	**77**	1					
139	*ZSCAN26*	rs16893892	Y/C	0,645	0,02084	**0,989**	**29**	**3934**	1	2				

Exomes of 129 control individuals with no missense mutations in the *ACE* gene (as in 48 controls from Fig. 5A) were analyzed for the presence of 2,388 genetic variants previously identified as significantly associated with blood ACE levels in a large Icelandic cohort [Ferkingstad, 2021; Table S1]. We identified 139 such variants among these controls. Variants highlighted in orange were detected in at least 2 control individuals with normal blood ACE levels. Those *ACE* variants whose frequency in the Icelandic cohort was more than 10-fold higher compared to the global dbSNP dataset (i.e., variants preferentially enriched in Iceland) are highlighted in light magenta. Mutations with mean allele frequency (MAF) at least 10-fold lower in the Icelandic cohort compared to the dbSNP reference population are highlighted in light yellow. Bold formatting denotes variants where a single rsID may represent multiple functional consequences. Variants with an absolute β value greater than 1.000 in GWAS are considered strongly associated with ACE levels.

Table S8. Corrected list of mutations (2248) that may influence blood ACE levels (Iceland's list).

Gene	rsID	Effect	UNIPROT	Protein position	AA subs.	Beta value	P value	Poly-Phen-2	ImpMAF /100 000	MAF dbSNP
AAAS	rs138043864	ms	Q9NRG9	37	D/N	-0,497	0,049174	0,018	35	1,5
AATF	rs202139717	ms	Q9NY61	178	S/N	-0,465	0,001559	0,003	67	8,7
AATF	rs761744080	ms	Q9NY61	221	V/G	0,816	0,018534	0,856	14	0,4
ABCA1	rs138880920	ms	O95477	776	K/N	0,395	0,043339	0,986	46	202
ABCA10	rs773731325	ms	Q8WWZ4	1402	E/A	-1,195	0,014695	0,942	12	0
ABCA13	rs1562947824	ms	A0A0A0MT16	2254	V/L	-0,626	0,046415	0,003	13	0
ABCA13	rs202134320	ms	A0A0A0MT16	4046	V/I	-0,879	0,010743	0,000	17	9,4
ABCA13	rs764466926	ms	A0A0A0MT16	4182	T/I	0,417	0,045727	0,006	33	0,7
ABCA6	rs199889249	ms	Q8N139	296	G/V	0,414	0,000036	0,988	150	30
ABCA8	rs1567829652	ms	O94911-3	1079	L/F	0,455	0,022878	0,031	43	0
ABCA8	rs4147979	ms	O94911-3	331	G/S	-0,648	0,035039	0,386	13	24
ABCA8	rs749671878	ms	O94911-3	996	M/I	-1,088	0,045480	0,003	15	0,4
ABCB1	7:87544848:CT/G	fs	P08183	680	R/X	-0,797	0,029990	1,000	12	N/A
ABCB1	rs149196148	ms	P08183	388	I/S	0,620	0,000816	0,993	81	1,9
ABCB5	rs778503529	ms	Q2M3G0-4	620	V/L	0,362	0,038571	0,027	55	0
ABCC1	rs182118381	ms	P33527	1103	G/D	-0,696	0,007635	1,000	27	130
ABCC2	rs766399139	ms	Q92887	1064	I/N	0,535	0,046997	0,975	16	0,4
ABCC3	rs151079073	ms	O15438	436	Q/K	0,740	0,024662	0,997	13	112
ABCC3	rs748107210	ms	O15438	1168	R/W	-0,438	0,017761	0,806	62	0,8
ABCF2	rs1563617859	ms	Q9UG63	139	P/S	-1,281	0,030709	0,993	14	0
ABCG1	rs750210537	ms	P45844-4	228	A/V	0,725	0,000721	0,695	39	1,1
ABCG4	rs1320169801	ms	Q9H172	631	R/Q	-1,157	0,000822	0,968	16	0,4
ABL1	rs370992010	ms	P00519	1021	R/Q	-0,424	0,019859	0,705	57	1,1
ABL2	rs148985886	ms	P42684	608	P/S	-0,769	0,027151	0,062	10	79
ABL2	rs745638514	ms	P42684-3	4	G/E	-0,963	0,004482	0,998	18	0,4
ABLIM2	rs752430305	ms	Q6H8Q1-9	20	T/M	-0,395	0,029098	0,577	43	8,3
ABO	rs8176743	ms	A0A087X009	234	G/S	0,351	5,356E-105	0,168	6,488	10587
ABO	rs8176746	ms	A0A087X009	265	L/M	0,350	8,358E-105	0,090	6,496	10589
ABO	rs8176747	ms	A0A087X009	267	G/A	0,350	9,314E-105	0,003	6,497	10589
ABRACL	rs768476146	ms	Q9P1F3	74	V/I	-0,368	0,041042	0,065	46	0,8
ACACA	rs748364574	ms	Q13085-4	1943	V/M	-0,849	0,040345	0,015	17	1,5
ACAN	rs548481167	ms	H0YMF1	2347	E/K	0,676	0,024650	0,001	24	23
ACAN	rs764412011	ms	H0YMF1	1619	A/P	-0,394	0,005607	0,020	97	0,4
ACAP1	rs746367494	ms	Q15027	217	R/Q	0,458	0,009814	0,455	50	2,3
ACE	rs141186617	ms	P12821	305	N/I	-0,692	0,022607	0,017	18	11
ACE	rs141543325	ms	P12821	228	R/C	0,933	0,007057	1,000	17	30
ACE	rs372416620	ms	P12821	1243	V/I	0,371	6,663E-09	0,061	469	5,7
ACE	rs3730025	ms	P12821	244	Y/C	-1,217	3,306E-291	0,998	1,534	924
ACE	rs571848794	ms	P12821	1013	G/S	-0,468	0,001102	0,183	93	6,8
ACE	rs750712925	ms	P12821	45	G/R	-1,288	6,826E-15	0,142	82	1,9
ACE	rs757694144	ms	P12821	482	R/P	-1,349	1,501E-07	0,246	34	0,4
ACKR1	rs529272627	ms	Q16570	124	R/L	0,416	0,007551	0,000	74	5,8
ACLY	rs750779834	ms	P53396	296	G/V	0,364	0,000331	0,437	159	3,0
ACOX3	rs759828894	ms	O15254	22	D/H	0,625	0,002260	0,067	31	2,6
ACP4	rs745993743	ms	Q9BZG2	42	G/S	-0,363	0,024882	1,000	69	0,6
ACR	rs1445899395	ms	P10323	368	P/S	0,784	0,024021	0,015	24	1,4
ACRBP	rs760419396	ms	Q8NEB7	162	R/C	-0,706	0,032420	0,679	15	0,4
ACSL3	rs760062740	ms	O95573	632	L/V	0,688	0,037619	0,634	11	0,3
ACTR1A	rs746393164	ms	P61163	340	T/M	0,381	0,032302	1,000	42	0,3
ADAM11	rs776835838	ms	O75078	678	P/A	-0,467	0,016173	0,080	42	0,1
ADAM22	rs201832352	ms	H7C3I4	630	G/E	-0,999	0,032157	0,021	12	14
ADAM29	rs755175800	ms	Q9UKF5	408	G/R	0,589	0,005401	0,918	29	0,4
ADAMDEC1	rs1207275267	ms	O15204	318	R/C	1,294	0,013085	0,970	10	0,4
ADAMTS10	rs144596955	ms	A0A0A0MQW6	669	V/L	0,742	0,032795	0,003	11	29
ADAMTS13	rs145825553	ms	Q76LX8-2	421	R/C	0,394	2,447E-08	0,994	334	45
ADAMTS13	rs748223519	ms	Q76LX8-2	439	E/K	-0,891	0,003181	0,205	24	0,7
ADAMTS14	rs150863733	ms	Q8WXS8	1110	S/L	-0,536	0,015959	0,000	29	41
ADAMTS17	rs146934810	ms	Q8TE56	183	K/R	-0,407	0,006986	0,000	79	221
ADAMTS18	rs148442712	ms	Q8TE60	660	S/N	0,519	0,008542	0,007	38	77
ADAMTS7	rs376434066	ms	Q9UKP4	1506	G/R	-0,918	0,005338	1,000	11	0,7
ADAMTS9	rs75938827	ms	Q9P2N4-3	1912	V/I	0,356	0,005406	0,000	96	192
ADAMTSL1	rs758299352	ms	Q8N6G6-3	387	A/T	-0,516	0,029702	0,802	32	1,5
ADAR	rs1557881072	ms	P55265	601	T/I	-0,593	0,027836	0,000	21	0
ADAT1	rs147999655	ms	Q9BUB4	417	Q/R	0,492	0,018278	0,953	32	33
ADCY2	rs1560953294	ms	Q08462	668	S/T	-0,351	0,046270	0,198	50	0,2
ADCY2	rs765899703	ms	Q08462	129	G/S	0,471	0,044594	0,177	25	0
ADGRA2	rs749263093	ms	Q96PE1	386	T/I	-1,077	0,006264	0,521	12	0,1
ADGRA3	rs756147612	ms	Q8IWK6	955	P/T	-0,395	0,000399	0,274	111	2,3
ADGRB3	rs147658947	ms	O60242	730	D/H	0,444	0,012257	0,842	47	0,4
ADGRE1	rs372416153	ms	Q14246	655	G/S	-0,578	0,036681	0,761	20	5,3
ADGRF4	rs755972498	ms	Q8IZF3	136	R/H	-0,878	0,001113	0,003	21	5,4
ADGRG6	rs191332808	ms	Q86SQ4-3	575	V/A	0,403	0,022701	0,003	50	15
ADORA2B	rs1567785802	ms	P29275	219	M/K	0,384	0,024868	0,001	44	0
ADORA2B	rs759669984	ms	P29275	8	A/E	-0,399	0,041289	0,077	65	0,7
ADPRHL1	rs532183571	ms	A0A0U1RQK4	1195	G/D	0,498	0,044893	0,970	21	0
ADRA2B	rs200889434	ms	P18089	358	R/H	0,584	0,050762	0,953	20	13
AEN	rs140747161	ms	Q8WTP8	19	I/F	0,370	0,003689	0,031	91	12
AEN	rs775685764	ms	Q8WTP8	131	R/C	0,725	0,033441	0,958	24	3,4
AFAP1L2	rs147799118	ms	Q8N4X5	381	H/R	0,512	0,043082	0,759	29	13

**AFAP1L2**	rs561584886	ms	Q8N4X5	218	V/M	-0,697	0,016007	0,942	22	0,4
AFF3	rs897323624	ms	P51826	801	P/L	0,784	0,046995	0,001	13	3,8
AGBL3	rs753584513	ms	Q8NEM8-4	878	L/I	0,613	0,020071	0,955	22	0,4
AGFG2	rs749263005	ms	O95081	241	P/A	0,833	0,019771	0,004	14	0,7
AGMAT	rs745384069	ms	Q9BSE5	285	A/V	0,600	0,041364	0,987	19	4,9
AGRN	rs149159118	ms	O00468-6	1118	T/K	0,700	0,020151	0,413	21	152
AGT	rs369425934	ms	P01019	118	V/M	0,446	0,040524	0,005	34	4,9
AGTPBP1	rs547428998	ms	Q9UPW5	691	R/C	-0,370	0,003025	0,720	121	1,5
AHCTF1	1:246850185:T/C	ms	Q8WYP5	1941	R/G	-0,545	0,032890	0,212	15	N/A
AHDC1	rs1557659784	ms	Q5TGY3	1063	V/I	-0,656	0,006647	0,097	27	0
**AHNAK2**	rs1566908248	ms	Q8IVF2	2573	S/R	0,640	0,016098	0,514	25	0
**AHNAK2**	rs202107671	ms	Q8IVF2	661	Q/K	0,361	0,042874	0,041	49	19
**AHNAK2**	rs767586966	ms	Q8IVF2	680	F/Y	-0,683	0,029822	0,731	18	1,9
**AHNAK2**	rs772834815	ms	Q8IVF2	5598	Q/H	-0,652	0,015460	0,737	21	0,2
AIFM2	rs771695112	ms	Q9BRQ8	244	G/S	-0,857	0,006595	0,993	18	0,8
AKAP12	rs199947814	ms	Q02952	1621	T/A	0,737	0,014305	0,000	13	12
**ALDH1B1**	rs1821278295	ms	P30837	135	Y/C	0,462	0,044309	0,030	28	0,2
**ALDH1B1**	rs142427338	stop	P30837	378	Q/*	0,399	0,026535	1,000	43	102
ALDH1L1	rs149080804	ms	O75891	107	P/L	-0,452	0,029996	0,970	36	71
ALDH3B1	rs369689965	ms	P43353	431	R/C	-0,560	0,044907	0,997	20	1,1
**ALMS1**	rs199573929	ms	Q8TCU4	679	Y/C	-0,754	0,030229	0,123	23	148
**ALMS1**	rs200266868	ms	Q8TCU4	1821	P/L	-0,796	0,021100	0,962	11	82
ALOX5AP	rs201182270	ms	P20292	117	R/C	-0,469	0,039286	1,000	27	0,8
ALPI	rs751686908	ms	P09923	212	N/D	-0,498	0,038468	0,998	34	1,9
**ALPK3**	rs139666355	ms	Q96L96	1134	A/V	-0,928	0,046406	0,007	12	92
**ALPK3**	rs767926913	ms	Q96L96	1107	Q/E	0,878	0,038700	0,038	11	8,3
ALPP	rs2981374	ms	P05187	501	P/H	1,097	0,012381	0,000	17	0,8
ALX3	rs975515598	ms	O95076	100	A/D	0,992	0,004368	0,699	12	0
AMBN	rs781315593	ms	Q9NP70	299	G/S	-0,972	0,012688	0,980	14	0
AMT	rs201189946	ms	P48728	379	E/A	0,436	0,024511	0,864	47	19
AMY1C	rs1430206652	ms	P0DTE8	9	T/S	-0,583	0,009503	0,000	33	0
AMY2B	rs753265856	ms	P19961	404	R/H	0,755	0,002053	0,209	24	3,0
ANAPC11	rs756571746	ms	Q9NYG5	71	Q/H	-0,390	0,042152	0,062	73	0,9
ANAPC13	rs756834266	ms	Q9BS18	18	A/V	-0,455	0,017991	0,548	44	0,9
ANGEL2	1:213013315:T/C	ms	Q5VTE6	55	M/V	0,477	0,051281	0,007	23	N/A
ANGPTL4	rs140744493	ms	Q9BY76	336	R/C	0,468	0,016221	0,980	45	246
**ANKFN1**	rs770360843	ms	A0A590UK59	14	N/I	0,755	0,016978	0,000	18	0
**ANKFN1**	rs773925251	ms	Q8N957	120	T/A	0,812	0,026185	0,013	12	1,9
**ANKFY1**	rs1567972969	ms	Q9P2R3	15	L/H	-1,160	0,002731	0,998	14	0
**ANKFY1**	rs763606938	ms	Q9P2R3	1131	R/H	0,682	0,024838	0,999	19	0,1
ANKHD1	rs745822360	ms	Q8IWZ3	2151	T/A	0,376	0,006643	0,511	76	0,4
ANKRD12	rs778977438	ms	Q6UB98	89	W/C	-0,824	0,017677	0,625	16	4,3
ANKRD20A1	rs1379856671	ms	Q5TYW2	403	I/K	0,617	0,007206	0,098	25	0,4
ANKRD27	rs372391792	ms	Q96NW4	944	S/L	0,541	0,019908	0,017	31	2,6
ANKRD30A	rs772253614	ms	Q9BXX3	235	D/G	-0,368	0,039127	0,637	46	1,5
ANKRD34B	rs200804923	ms	A5PLL1	282	N/S	-0,374	0,002712	0,006	126	26
ANKRD62	18:12125936:A/C	ms	A6NC57	705	Q/H	-0,736	0,000679	0,788	33	N/A
ANKS1A	rs986675244	ms	Q92625	438	M/V	-1,015	0,050749	0,000	13	0,4
ANO6	rs372908985	ms	Q4KMQ2	579	Y/S	-0,549	0,027306	0,730	29	4,5
ANO7	rs138809031	ms	A0A6I8PRE6	54	R/W	-0,542	0,044076	0,226	19	18
ANTXRL	rs750794572	ms	A6NF34	6	S/C	-0,380	0,023433	0,791	69	4,7
ANXA2R	rs756512520	ms	Q3ZCQ2	94	P/S	-0,505	0,001979	0,982	66	0,2
AOC3	rs145964446	ms	Q16853	272	D/N	0,541	0,041555	0,003	18	12
AP2A2	rs200859967	ms	O94973	658	S/L	-0,500	0,046662	0,883	43	25
AP2B1	rs749754793	ms	P63010-2	235	R/G	-0,665	0,002945	0,263	30	4,7
AP4B1	rs200590674	ms	Q9Y6B7	415	C/Y	-0,615	0,050783	0,673	18	24
APAF1	rs770595178	ms	O14727	51	Q/R	0,770	0,050745	0,003	14	0,4
APC	rs34919187	ms	P25054	2274	A/V	-0,545	0,031215	0,005	17	94
APCDD1L	rs145873693	ms	Q8NCL9	330	V/M	1,311	0,002047	0,069	21	67
APOA1	rs138407155	ms	P02647	95	F/Y	0,450	0,010564	0,904	63	47
APOB	rs374473614	stop	P04114	725	Q/*	1,016	0,005874	1,000	19	0,4
APOBEC3D	22:39031830:T/C	ms	Q96AK3	300	V/A	0,603	0,019377	0,962	16	N/A
APOL1	rs73885316	ms	O14791	264	N/K	0,937	0,001830	0,572	21	839
APPL2	rs775024469	ms	Q8NEU8	532	M/K	-0,899	0,005984	0,014	17	0
ARAP2	rs1560721800	ms	Q8WZ64	190	T/A	-1,043	0,041357	0,001	16	0
ARFGEF3	rs145180404	ms	Q5TH69	691	S/P	-0,366	0,018544	0,000	48	33
**ARHGAP32**	rs377531423	ms	A0A804HK06	1957	R/S	0,400	0,008489	0,996	64	9,1
**ARHGAP32**	rs746664458	ms	A0A804HK06	1202	G/R	-0,459	0,001278	0,308	71	0,5
ARHGAP35	rs373252908	ms	Q9NRY4	577	R/W	0,645	0,042049	0,943	22	1,1
ARHGDIA	rs150457411	ms	P52565	155	E/K	-0,810	0,025235	0,763	13	7,2
ARHGEF11	rs766422545	ms	O15085-2	520	M/V	0,542	0,044379	0,070	23	1,1
ARHGEF16	rs745895895	ms	Q5VV41	147	R/W	-0,532	0,041445	0,995	33	1,5
ARHGEF28	rs756796241	ms	Q8N1W1	593	P/R	0,696	0,026927	0,305	25	0,4
ARHGEF40	rs892869054	ms	Q8TER5	695	R/W	-0,474	0,029272	0,982	28	0,8
ARHGEF5	rs2053728428	ms	Q12774	1306	L/F	-0,680	0,040086	0,998	21	0
ARID5B	rs199637139	ms	Q14865	901	T/M	-1,574	0,008946	0,295	12	2,0
ARL11	rs779514414	ms	Q969Q4	129	A/T	0,665	0,014751	0,999	14	0,6
ARPC5L	rs367615280	ms	Q9BPX5	99	S/T	-0,743	0,010944	0,010	28	2,6
ARPP19	rs541368973	ms	P56211	29	E/A	0,575	0,039796	0,011	14	0,6
ART1	rs199916833	stop	P52961	29	R/*	-0,574	0,002220	1,000	39	16
AS3MT	rs200488263	ms	Q9HBK9	117	Y/H	0,573	0,011871	0,108	17	14
ASAP3	rs140136454	ms	Q8TDY4	717	A/D	0,449	0,001145	0,670	93	527
ASB14	rs202057906	ms	A6NK59-3	386	P/L	0,549	0,048831	0,998	18	8,7
ASB16	rs755814254	ms	Q96NS5	350	P/L	0,506	0,020507	0,391	32	6,0

ASB3	rs760580086	ms	Q9Y575	140	G/R	0,792	0,044430	0,803	22	0,4
ASGR2	rs377144273	ms	Q7Z4G9	116	V/M	0,652	0,013707	0,106	28	13
ASIC1	rs746957755	ms	P78348-2	253	D/N	0,459	0,049083	0,019	20	0,4
ASPHD2	rs140657637	ms	Q6ICH7	53	V/M	0,553	0,020682	0,212	31	2,3
ASPM	rs1557956556	ms	Q8IZT6	1117	M/V	1,062	0,041921	0,970	19	0,4
ASXL3	rs201152513	ms	Q9C0F0	1140	P/Q	-0,495	0,004076	0,382	84	3,8
ATAD2	rs746856878	ms	Q6PL18	1339	T/P	0,722	0,012543	0,085	19	0
ATCAY	rs147684273	ms	Q86WG3	124	V/M	0,906	0,013274	0,382	15	5,7
ATF7IP	rs777624434	ms	Q6VMQ6	750	T/A	-0,731	0,020773	0,003	14	0,8
ATG9A	rs779637846	ms	Q7Z3C6	731	H/Y	0,457	0,044702	0,934	29	0,4
ATP12A	13:24706450:G/A	ms	P54707	719	G/D	-0,647	0,049475	0,994	10	N/A
ATP1A4	rs377713967	ms	Q13733	1019	P/L	0,368	0,021009	0,928	69	35
ATP2C2	rs763173008	ms	O75185	567	T/M	-0,713	0,001096	0,308	35	0,8
ATP5IF1	rs758401235	ms	Q9UII2	79	V/F	0,603	0,016526	0,058	17	0
ATP5PB	rs151251385	ms	P24539	208	R/C	0,616	0,035845	0,994	15	4,3
ATP6V0A1	rs141898946	ms	Q93050-2	774	V/M	0,522	0,019485	0,272	22	39
ATP6V0E2	rs761485568	ms	Q8NHE4	46	V/I	-0,444	0,024509	0,174	57	0,8
ATRAID	rs147821107	ms	Q6UW56	114	R/C	-0,393	0,050338	0,830	49	10
ATRN	rs775375659	ms	O75882	820	N/S	0,538	0,006562	0,025	45	30
ATRNL1	rs1229952017	ms	Q5VV63	971	H/Y	1,074	0,003547	0,000	12	0,8
AXIN2	rs115931022	ms	Q9Y2T1	412	N/S	-0,385	0,002435	0,000	124	494
B3GNTL1	rs201686706	ms	Q67FW5	203	G/S	0,586	0,027334	0,001	23	0,7
BAG2	rs573834496	ms	O95816	181	I/T	-0,360	0,044023	0,532	71	4,5
BAG6	rs755726048	ms	P46379-3	634	D/H	-0,369	0,048306	0,811	47	1,9
BARD1	rs142155101	ms	Q99728	761	S/N	-0,574	0,023373	0,005	25	73
BAZ1A	rs1566551387	ms	Q9NRL2	1078	H/R	0,758	0,015930	0,033	12	0,4
BAZ1A	rs759984179	ms	Q9NRL2	1372	P/T	0,733	0,026105	0,981	11	1,4
BBIP1	rs1473657985	ms	A8MTZ0	45	I/M	1,050	0,007087	0,581	11	0,8
BCAS3	rs771742994	ms	Q9H6U6-2	480	S/I	-1,162	2,188E-12	0,991	88	6,4
BCHE	rs116097205	ms	P06276	61	Y/C	0,863	0,033205	1,000	12	0,8
BCKDHA	rs779476349	ms	P12694	31	R/W	-0,753	0,009169	0,245	19	0
BCO1	rs139655280	ms	Q9HAY6	288	D/N	0,406	0,011986	0,265	62	6,0
BDKRB1	rs757505732	ms	P46663	219	V/I	0,662	0,017464	0,005	22	1,1
BEAN1	rs755281092	ms	Q3B7T3	42	V/I	-0,469	0,034902	0,972	30	9,8
BEST3	rs199545316	ms	Q8N1M1-2	211	M/I	-0,374	0,023061	0,129	51	29
BICD1	rs200717707	ms	Q96G01	397	R/W	0,440	0,025753	0,916	44	0,8
BIN3	rs746437989	ms	Q9NQY0	233	R/W	-0,532	0,009151	0,676	40	1,3
BIRC6	rs1353906962	ms	Q9NR09	4009	G/E	0,870	0,041127	0,997	10	0,4
BIRC7	rs149161695	ms	Q96CA5	206	R/T	0,369	0,019169	0,005	60	22
BIRC7	rs201155031	ms	Q96CA5	245	R/Q	-1,306	0,000911	0,000	12	1,9
BLTP2	rs200477170	ms	Q14667	36	R/W	-0,770	0,025382	0,091	16	1,5
BMAL1	rs769220676	ms	O00327-2	58	H/N	-0,772	0,025251	0,051	14	0,2
BMAL1	rs777322872	ms	O00327-2	46	Y/C	0,471	0,027682	0,874	27	1,5
BMP3	rs61729826	ms	P12645	345	K/N	-0,590	0,023115	0,998	24	1103
BOC	rs78524315	ms	Q9BWV1-3	534	R/C	0,513	0,008626	0,566	40	35
BOLA2	16:29454204:T/G	ms	Q9H3K6	17	E/A	-0,453	0,025124	0,010	29	N/A
BOP1	rs1160618575	ms	Q14137	673	P/R	-1,512	0,003735	0,015	11	0
BORCS5	rs565487491	ms	Q969J3	135	R/C	0,386	0,021797	0,989	54	12
BPIFA2	rs1075435	ms	Q96DR5	236	V/L	-1,106	0,033823	0,000	15	8747
BPTF	rs748420257	ms	Q12830-2	240	N/S	-1,662	0,031778	0,043	13	0
BPTF	rs780473362	ms	Q12830-2	1622	K/E	0,451	0,012177	0,309	54	3,8
BRD2	rs34530779	ms	P25440	569	A/T	0,456	0,001069	0,002	99	610
BRD3	rs201916092	ms	Q15059	429	A/V	-0,399	0,050012	0,003	40	0,4
BRME1	rs769178284	ms	Q0VDD7	519	S/Y	-0,430	0,031732	0,788	40	4,2
BSN	rs749714827	ms	Q9UPA5	1867	M/L	-0,429	0,006319	0,001	62	0,4
BTBD17	rs145160027	ms	A6NE02	117	D/E	0,484	0,033120	0,042	45	80
BTBD17	rs752498641	ms	A6NE02	117	D/N	-0,538	0,014078	0,869	30	1,1
BTBD8	rs778129490	ms	Q5XKL5-3	1263	K/R	-0,445	0,003843	0,999	83	0
BYSL	rs368935062	ms	Q13895	25	A/G	-0,387	0,020314	0,003	60	0,7
BYSL	rs754194109	ms	Q13895	364	F/L	-0,519	0,000570	0,994	59	0,3
C10orf95	rs755941591	ms	A0A1B0GTG0	171	T/A	-0,560	0,002708	0,129	54	26
C11orf58	rs771370191	ms	O00193	155	E/K	0,950	0,001622	0,807	10	1,1
C12orf60	rs140864709	ms	Q5U649	56	I/F	0,565	0,000894	0,085	47	226
C13orf42	rs529205663	ms	A0A1B0GVH6	154	E/K	0,810	0,039636	0,011	14	4,3
C14orf39	rs1566665407	ms	Q8N1H7	369	K/Q	0,747	0,009816	0,440	16	0
C15orf39	rs1567139783	ms	Q6ZRI6	629	P/A	0,920	0,030618	0,000	17	0,1
C16orf96	rs1567456672	ms	A6NNT2	907	F/Y	-0,590	0,013213	0,970	26	0
C18orf54	rs1568181806	ms	Q8IYD9-2	192	N/D	-0,548	0,040945	0,306	16	0,2
C19orf67	rs553160046	ms	A6NJJ6	65	T/M	0,793	0,043969	0,000	14	14
C1orf159	rs139486403	ms	Q96HA4	304	D/E	-0,845	0,022047	0,631	15	34
C1QTNF9B	rs371732332	ms	B2RNN3	69	T/A	0,553	0,003163	0,000	50	20
C1R	rs200899486	ms	A0A3B3ISR2	294	D/N	-0,384	0,040329	0,012	40	11
C20orf204	rs557949001	ms	A0A1B0GTL2	110	G/R	-0,368	0,017654	0,788	67	139
C2orf92	rs143227941	fs	A0A1B0GVN3	246	E/X	0,414	0,024967	1,000	54	5084
C2orf92	rs17029878	ms	A0A1B0GVN3	188	Q/L	0,414	0,024967	0,617	54	5091
C3orf20	rs750004016	stop	Q8ND61	606	R/*	-0,470	0,016256	1,000	40	1,4
C3orf70	rs777199280	ms	A6NLC5	110	S/P	0,827	0,051272	0,000	16	2,3
C4A	6:31992420:G/A	ms	P0C0L4	582	R/Q	0,571	0,048817	0,017	29	N/A
C4B	6:32025158:G/A	ms	P0C0L5	582	R/Q	0,571	0,048817	0,057	29	N/A
C4orf3	rs190403527	ms	Q8WVX3	24	G/R	0,718	0,051515	0,000	13	87
C5AR1	rs200740207	ms	P21730	44	F/L	0,475	0,039870	0,000	34	11
C6	rs778566926	ms	P13671	593	A/V	-0,697	0,003610	0,791	33	0,8
C8A	rs142382705	ms	P07357	207	R/W	-1,172	0,046057	0,999	10	5,4
CA9	rs1563923396	ms	Q16790	332	T/S	-0,599	0,016175	1,000	17	0,1

CABP1	rs755254500	ms	Q9NZU7	66	R/C	-0,653	0,012227	0,976	31	0,7
**CACNA1C**	rs121912775	ms	Q13936-12	490	G/R	-0,595	0,039862	0,795	16	44
**CACNA1C**	rs749190892	ms	Q13936-12	1704	G/D	0,362	0,001148	0,762	103	0
CACNA1G	rs368939625	ms	O43497	1574	D/Y	-0,957	0,032026	0,426	10	3,3
CACNA1H	rs774565789	ms	O95180	281	T/M	1,508	0,000317	0,830	10	2,3
CACNB1	rs141322943	ms	Q02641-2	233	G/S	0,721	0,015278	0,000	19	322
CADPS	rs755251601	ms	Q9ULU8	32	R/L	0,382	0,003534	0,000	116	1,1
CADPS2	rs1563339160	ms	Q86UW7	787	M/V	-0,763	0,038445	0,135	13	0
**CAMSAP1**	rs748961061	ms	Q5T5Y3	76	P/L	0,420	0,023329	0,999	57	0,8
**CAMSAP1**	rs758753847	ms	Q5T5Y3	1592	P/L	-0,474	0,016720	0,994	48	0,4
CAMTA1	rs141259598	ms	Q9Y6Y1	707	S/C	-0,428	0,036325	0,441	42	48
CAND2	rs763105870	ms	O75155	575	L/R	-0,899	0,007464	0,300	15	0,4
CAPN10	rs200682095	ms	Q9HC96	340	R/Q	0,824	0,012042	0,007	21	14
CAPN12	rs147906486	ms	Q6ZSI9	211	V/M	-0,362	0,004801	0,927	86	56
CAPN2	rs745412257	stop	P17655	612	R/*	0,363	0,048989	1,000	37	1,1
CAPN3	rs1414687788	ms	P20807	248	M/L	-0,828	0,051781	0,001	11	0,8
CAPNS2	rs146637108	ms	Q96L46	125	R/Q	-0,589	0,045470	0,962	16	164
CARD10	rs1569161077	ms	Q9BWT7	1030	S/I	1,304	0,005090	0,774	19	0,6
**CARD6**	rs148780446	ms	Q9BX69	857	S/L	0,875	0,011883	0,000	17	257
**CARD6**	rs1561216545	ms	Q9BX69	603	K/T	-0,635	0,043358	0,883	23	0
CASC3	rs768468747	ms	O15234	271	P/L	0,685	0,005181	0,077	21	2,7
CASD1	rs1562947072	ms	Q96PB1	494	L/V	0,650	0,010219	0,434	30	0
CASKIN2	rs150397249	ms	Q8WXE0	410	V/M	-0,718	0,002670	0,114	32	35
CASP14	rs368748073	ms	P31944	90	G/R	-0,454	0,022962	1,000	35	2,6
CASP4	rs56008239	ms	P49662	344	R/Q	-1,008	0,004577	0,167	16	23
CASZ1	rs1194855522	ms	Q86V15	1171	A/V	-0,688	0,009429	0,972	19	0,7
**CATSPER4**	rs371089081	ms	Q7RTX7	205	R/H	0,599	0,049872	0,145	13	2,8
**CATSPER4**	rs778169515	ms	Q7RTX7	73	A/T	-0,622	0,020198	0,003	15	4,0
CAVIN3	rs1228057325	ms	Q969G5	83	A/V	0,711	0,023820	0,972	21	0,7
CBARP	rs771515759	fs	Q8N350-4	407	P/X	-0,789	0,029755	1,000	14	65
CBARP	rs777136090	ms	Q8N350-4	429	A/G	0,507	0,038990	0,000	37	12
CBFA2T3	rs201916925	ms	O75081	447	G/S	-0,374	0,005197	0,009	88	11
CBLIF	rs748783434	ms	P27352	266	M/R	-0,508	0,022442	0,949	39	0,7
CCDC110	rs141263491	ms	Q8TBZ0	540	M/V	0,483	0,049447	0,014	38	27
CCDC15	rs371930839	ms	Q0P6D6	121	S/L	0,801	0,021411	0,194	18	11
CCDC17	rs766641176	ms	Q96LX7-4	231	Y/H	0,983	0,002674	0,665	13	0,7
CCDC172	10:116378454:G/A	ms	P0C7W6	229	D/N	-0,677	0,040231	0,511	10	N/A
CCDC178	rs140513669	ms	Q5BJE1	504	R/H	0,434	0,050775	0,000	31	8,7
CCDC18	rs774207938	ms	A0A8I5KWA2	915	E/Q	-0,638	0,014526	0,171	32	1,1
**CCDC182**	rs149716379	ms	A6NF36	131	R/Q	-0,749	0,003280	0,000	32	339
**CCDC182**	rs769238436	ms	A6NF36	60	R/G	-0,554	0,049648	0,062	26	5,9
CCDC47	rs778565800	ms	Q96A33	390	I/L	0,420	0,006050	0,015	68	1,1
CCDC77	rs148861801	ms	Q9BR77	338	S/N	-0,699	0,001850	0,000	35	7,9
CCDC8	rs774700757	ms	Q9H0W5	268	K/N	0,479	0,038348	0,752	33	0
CCDC82	rs772526152	ms	Q8N4S0	280	E/A	-0,392	0,050512	0,140	35	0,8
**CCDC88C**	rs763668804	ms	Q9P219	697	R/C	-0,392	0,003467	0,692	83	2,6
**CCDC88C**	rs769113965	ms	Q9P219	82	V/L	-0,365	0,010916	0,176	73	0,8
CCDC9	rs201241449	ms	Q9Y3X0	230	R/W	0,647	0,034373	0,000	19	92
CCER1	rs377744456	ms	Q8TC90	164	A/V	0,410	0,049208	0,044	47	4,2
**CCER2**	rs181740944	ms	I3L3R5	209	R/H	0,461	0,027214	0,000	36	235
**CCER2**	rs778686851	ms	I3L3R5	74	A/V	-0,897	0,004277	0,000	24	1,9
CCM2	rs747384033	ms	Q9BSQ5	15	S/L	-0,485	0,001587	0,514	51	0,4
CCN4	rs752881842	ms	O95388	365	I/T	-0,705	0,024856	0,893	15	4,9
CCNG2	rs1560422355	ms	Q16589	152	E/Q	0,458	0,044025	0,036	20	0
CCNI	4:77055296:T/G	ms	Q14094	182	T/P	0,523	0,030330	0,929	20	N/A
CCR4	rs1410395535	ms	P51679	89	V/M	1,068	0,000605	0,999	21	0,4
CCR7	rs143854738	ms	P32248	359	R/Q	0,760	0,025907	0,192	18	0,4
**CCT8**	rs766748030	ms	P50990	459	K/E	-0,363	0,047655	0,763	50	0
**CCT8**	rs776634991	ms	P50990	4	H/R	-0,363	0,047644	0,006	50	0,4
CCT8L2	rs149623775	ms	Q96SF2	133	R/Q	-0,432	0,015507	0,366	51	99
CD200R1	rs200744866	ms	Q8TD46-4	277	I/V	0,600	0,017731	0,017	19	8,3
CD300E	rs772510010	ms	Q496F6	117	R/C	-0,625	0,015550	0,946	19	0,9
CD3E	rs148647954	ms	P07766	71	D/H	0,398	0,006876	0,000	94	49
CD44	rs762054400	ms	P16070	304	I/V	0,994	0,019366	0,001	15	0,2
CD53	rs150734121	ms	P19397	165	R/Q	0,764	0,004364	0,000	25	65
CD81	rs538164293	ms	P60033	152	E/Q	0,376	0,022388	0,820	53	0,8
**CD93**	rs1000470607	ms	Q9NPY3	409	C/Y	-0,453	0,015333	0,998	36	0,8
**CD93**	rs1050339456	ms	Q9NPY3	249	D/N	0,565	0,014834	0,162	27	2,0
**CDA**	rs752974013	ms	P32320	38	V/L	-0,422	0,034594	0,876	32	0,4
**CDA**	rs758866204	ms	P32320	38	V/A	-0,422	0,034594	1,000	32	0,4
CDC14A	rs755456667	ms	Q9UNH5-2	609	P/L	0,365	0,044713	0,130	53	2,3
CDC23	5:138202147:TT/G	fs	Q9UJX2	127	E/X	0,377	0,020556	1,000	47	N/A
CDC45	rs1568931035	ms	O75419	305	V/L	-0,789	0,044752	0,001	16	0
CDH13	rs190049290	ms	P55290	565	A/S	-0,620	0,038192	0,996	20	0,8
**CDH23**	rs111033494	ms	Q9H251	2199	I/S	-0,983	0,020435	0,129	14	402
**CDH23**	rs764515216	ms	Q9H251	211	D/E	0,700	0,011638	0,013	18	1,9
CDH4	rs202190196	ms	P55283	565	T/M	0,589	0,041327	0,863	32	14
CDH8	rs1264566952	ms	P55286	524	D/N	-0,847	0,013990	0,115	22	1,1
CDHR1	rs766579684	ms	Q96JP9	223	D/N	0,914	0,008024	0,003	13	1,5
CDHR3	rs201055622	ms	Q6ZTQ4	347	K/N	0,451	0,012901	0,035	50	7,9
CDHR4	rs768814405	ms	A6H8M9	229	V/F	0,532	0,030119	0,152	18	6,5
CDK12	17:39530722:G/C	ms	Q9NYV4	1293	L/F	0,533	0,044982	0,000	28	N/A
CDK15	rs757611284	ms	Q96Q40	152	L/V	0,477	0,048545	0,999	19	3,0
CEBPD	rs935024096	ms	P49716	130	A/V	-0,350	0,023110	0,011	67	15

CEBPZ	rs757307902	ms	Q03701	206	K/R	-0,671	0,000429	0,080	37	0,5
CEMIP	rs144685141	ms	Q8WUJ3	1277	F/L	-0,593	0,040341	0,000	16	7,9
CEND1	rs538479515	ms	Q8N111	62	K/T	-0,549	0,025033	0,617	47	4,9
CENPC	rs756146915	ms	Q03188	607	D/E	0,486	0,040916	0,000	15	0
CENPF	rs775339410	ms	P49454	2591	K/E	-1,463	0,015151	0,010	14	0,2
CENPJ	rs17081389	ms	Q9HC77	55	P/A	0,874	0,026512	0,061	13	2747
CENPP	rs770185935	ms	Q6IPU0	46	N/S	0,589	0,011606	0,000	23	1,1
CEP112	rs777140697	ms	Q8N8E3	73	M/T	0,415	0,000004	0,781	195	0,4
CEP164	rs1324239157	stop	Q9UPV0	1021	Q/*	-0,909	0,021243	1,000	12	0,1
**CEP290**	rs184018899	ms	O15078	557	R/H	1,030	0,048276	0,946	10	68
**CEP290**	rs765211180	ms	O15078	31	S/P	0,559	0,025991	0,351	35	0,2
CEP295	rs748809577	ms	Q9C0D2	2470	R/H	0,432	0,037640	0,067	42	3,0
CERK	rs143397270	ms	Q8TCT0	384	V/I	-0,535	0,019237	0,003	27	100
**CFAP**418	rs369850560	ms	Q96NL8	47	A/P	0,621	0,032667	0,003	14	1,9
**CFAP54**	rs1565919386	ms	Q96N23	1347	M/V	-0,722	0,050280	0,000	14	0,8
**CFAP54**	rs746538349	ms	Q96N23	1492	H/R	0,357	0,030149	0,899	61	11
CFAP57	rs769255988	ms	Q96MR6	217	T/R	-0,796	0,011386	0,029	14	0,9
CFAP65	rs773282060	ms	Q6ZU64	403	E/K	-0,614	0,031171	0,001	17	4,5
CFAP73	rs61738699	ms	A6NFT4	45	A/T	-0,412	0,026376	0,031	52	675
CFAP74	rs771618335	ms	A0A804HLA9	58	T/S	0,593	0,036773	0,003	18	4,9
CFAP95	rs142028833	ms	Q5VTT2	159	D/G	-0,581	0,046827	0,123	23	82
CFHR1	rs145057542	stop	Q03591	331	*/W	-0,513	0,016653	1,000	23	41
CFHR5	rs1558287665	ms	Q9BXR6	308	M/V	0,531	0,024341	0,001	25	0,3
CGN	rs376782764	ms	Q9P2M7	211	R/W	-1,060	0,005845	0,999	14	3,4
CHAC2	rs531154748	ms	Q8WUX2	127	I/T	0,558	0,045585	0,936	19	0,7
CHCHD6	rs1559811150	ms	Q9BRQ6	118	A/T	-0,820	0,035315	0,006	12	0
CHCT1	rs756919587	ms	Q86WR6	233	E/D	-0,548	0,006172	0,330	45	26
CHD3	rs1567833092	ms	Q12873	70	K/T	0,498	0,024487	0,413	28	0,4
CHD4	rs769476360	ms	Q14839	1762	N/S	-0,706	0,032468	0,983	15	1,9
CHD6	rs1345334517	ms	Q8TD26	2487	M/T	-1,596	0,023908	0,025	12	0,4
CHD8	rs754064184	ms	Q9HCK8	1469	R/H	0,519	0,045465	0,892	25	0,4
CHRDL2	rs141077727	ms	Q6WN34	15	A/T	1,269	0,001294	0,087	16	168
CHRM3	rs757383989	ms	P20309	275	A/V	-0,724	0,016255	0,101	14	0
CHRND	rs777430398	ms	Q07001	481	M/T	0,878	0,010930	0,991	14	3,8
CHRNE	rs121909516	ms	Q04844	163	S/L	-0,449	0,004708	0,951	70	46
CHRNG	rs375987895	ms	P07510	332	R/Q	-0,392	0,035836	0,834	52	2,5
CHST5	rs145210445	ms	Q9GZS9	22	M/I	0,386	0,010940	0,390	56	43
**CIC**	rs1568486138	ms	A0A7P0T9K5	372	A/V	-0,578	0,045390	0,015	16	0
**CIC**	rs1568487984	ms	A0A7P0T9K5	821	P/L	0,653	0,034965	0,856	12	0
CIDEA	rs149990783	ms	O60543	98	H/R	-0,719	0,001551	0,631	30	15
CIITA	rs763945523	ms	A0A0B4J1S1	383	A/D	0,490	0,025484	0,811	22	9,8
**CIT**	rs1204187421	ms	O14578-4	1910	I/M	-1,115	0,001211	0,960	11	0,2
**CIT**	rs747447396	ms	O14578-4	947	R/C	0,538	0,044801	0,441	25	1,1
CKM	rs200170574	ms	P06732	83	E/K	-0,569	0,034546	0,353	16	0,4
CKMT2	rs199560112	ms	P17540	116	Y/C	-0,764	0,006132	1,000	24	1,9
CLASP2	rs114138303	ms	A0A0U1RQI6	476	A/T	-0,809	0,00005573	0,697	42	58
CLDN9	rs755885384	ms	O95484	215	D/V	-0,480	0,016891	0,274	38	1,5
CLEC16A	rs202155030	ms	Q2KHT3	501	V/M	0,470	0,031765	0,996	23	12
CLEC18C	16:70184579:A/G	ms	Q8NCF0	357	N/S	0,382	0,016033	0,622	71	N/A
CLEC2L	rs1430032217	ms	P0C7M8	101	P/S	-0,875	0,039988	0,829	14	2,6
CLEC4M	rs140767813	ms	Q9H2X3	263	R/H	-0,976	0,004897	0,771	15	17
CLEC9A	rs201379734	ms	Q6UXN8	202	E/Q	-0,560	0,051893	0,003	17	15
CLMN	rs148836979	ms	Q96JQ2	158	P/A	-1,077	0,001091	0,635	26	96
CLN6	rs769900670	ms	Q9NWW5	310	R/Q	-0,370	0,000388	0,000	131	1,5
CLTC	rs769713826	ms	Q00610	1138	M/T	-0,431	0,011711	0,280	57	0
CLUAP1	rs35065818	ms	Q96AJ1	196	V/F	0,707	0,031917	0,726	16	838
CMKLR2	rs779420589	ms	P46091	137	I/N	-0,689	0,006507	0,607	14	0,6
CMTR1	rs771375770	ms	Q8N1G2	605	G/S	-0,437	0,024352	0,000	51	4,2
CMYA5	rs769699893	ms	Q8N3K9	1459	V/I	-0,567	0,005201	0,007	44	0,8
CNBD1	rs750531941	ms	Q8NA66	421	K/R	0,479	0,020795	0,565	32	0,3
CNGA3	rs141086649	ms	Q16281	37	S/L	0,506	0,026202	0,000	40	16
CNGB1	rs369817749	ms	Q14028	311	P/L	0,459	0,029420	0,005	51	5,3
CNIH3	rs529360966	ms	Q8TBE1	85	A/V	-0,749	0,040748	0,682	23	0,4
CNNM4	rs561036245	ms	Q6P4Q7	122	N/I	-1,037	0,003385	0,261	19	9,8
CNTF	rs776744796	ms	P26441	172	S/A	0,494	0,007477	0,989	61	0
CNTN3	rs1559690789	ms	Q9P232	655	G/R	1,346	0,000260	0,999	10	0
CNTN4	rs375898825	ms	Q8IWV2	86	I/F	-0,377	0,047936	0,971	44	1,9
CNTN6	rs149799168	ms	Q9UQ52	310	G/S	0,423	0,008229	0,999	67	103
**CNTNAP3B**	rs1200081323	ms	Q96NU0	817	V/M	1,319	0,012235	0,085	10	1,3
CNTNAP5	rs757893588	ms	A0A804HKY0	1054	L/S	0,724	0,016171	0,999	16	0
COG1	rs751977123	ms	Q8WTW3	892	T/M	0,603	0,025804	0,051	19	1,5
COG4	rs772833758	ms	J3KNI1	461	I/L	0,734	0,034230	0,292	18	0,8
COIL	rs138873632	ms	P38432	419	R/Q	0,479	0,011847	0,987	45	22
COL16A1	rs760281570	ms	Q07092	20	H/R	0,394	0,045730	0,000	30	0,2
COL18A1	rs772587390	ms	P39060-2	146	Q/H	0,886	0,004456	0,000	21	0,1
COL1A2	rs1282395129	ms	P08123	437	D/V	-0,632	0,012548	0,997	23	0,4
COL20A1	rs199924678	ms	Q9P218	782	G/E	0,556	0,036858	0,669	25	4,9
COL22A1	rs764460053	ms	Q8NFW1	604	R/Q	-0,764	0,038113	0,000	13	0,4
COL24A1	rs773642410	stop	Q17RW2	1175	Q/*	0,877	0,005274	1,000	14	6,8
COL27A1	rs752587458	ms	Q8IZC6	303	T/S	0,535	0,007147	0,000	46	0,4
COL4A4	rs1559515185	ms	P53420	843	G/A	-0,689	0,003875	0,000	27	0,3
COL5A1	rs147589613	ms	P20908	114	A/D	0,603	0,009283	0,000	21	88
COL6A5	rs182112053	ms	H0Y393	1936	R/W	-0,396	0,025719	0,003	48	136
COL9A2	rs781428852	ms	Q14055	634	N/K	0,375	0,051653	0,826	61	3,3

COL9A3	rs780489761	ms	Q14050	584	G/S	-0,886	0,043537	0,998	10	0,4
COLEC12	rs1001323032	ms	Q5KU26	496	R/H	-0,766	0,027514	0,000	12	1,0
COPA	rs1557861801	ms	P53621	751	N/H	0,761	0,006373	0,058	25	0,4
COPG2	rs1174813614	ms	Q9UBF2	466	T/M	1,005	0,010779	0,518	23	3,0
COPG2	rs900527557	ms	Q9UBF2	513	L/I	-0,702	0,009171	0,719	21	17
COPS4	rs769306260	ms	Q9BT78	347	R/C	0,487	0,013098	0,999	37	0,9
CPAMD8	rs199816113	ms	Q8IZJ3	51	V/M	-0,848	0,031603	0,948	11	11
CPD	rs766105447	ms	O75976	318	K/R	-0,571	0,041620	0,017	25	0,4
CPEB1	rs549292365	ms	A0A087WVR7	23	I/S	0,633	0,005429	0,000	31	9,1
CPEB1	rs200188266	ms	A0A087WXG7	114	R/C	0,470	0,023830	0,879	36	16
CPSF2	rs140484405	ms	Q9P2I0	526	T/A	0,382	0,041955	0,074	49	96
CPT1A	rs1566348900	fs	P50416	569	F/X	-1,527	0,004625	1,000	15	0
CPT1B	rs773375016	ms	Q92523	517	R/W	-0,887	0,015027	0,999	14	0,4
CR1	rs1306545385	ms	E9PDY4	965	I/T	0,392	0,035544	0,023	56	1180
CR1	rs1553290579	ms	E9PDY4	515	I/T	0,392	0,035562	0,065	57	0
CR1	rs1553292784	ms	E9PDY4	1415	I/T	0,387	0,041084	0,006	56	686
CRACDL	rs753045151	ms	Q6NV74	692	S/L	-0,372	0,044683	0,996	52	1,0
CRAT	rs759917365	ms	P43155	244	N/K	-0,824	0,004506	0,045	35	5,7
CRAT	rs762626138	ms	P43155	605	H/R	0,358	0,041665	0,000	52	9,4
CRB2	rs545270434	ms	Q5IJ48	327	R/W	-0,375	0,018390	0,761	49	0,8
CRB2	rs753967204	ms	Q5IJ48	747	V/M	-0,679	0,009851	0,988	25	0,9
CRHR1	rs2062265678	ms	P34998-2	179	E/K	-0,803	0,049250	0,030	11	0,4
CRHR1	rs372181383	ms	P34998-2	95	A/T	0,365	0,000237	0,001	168	6,4
CRHR2	rs151248954	ms	Q13324	367	R/C	0,514	0,011909	0,901	47	6,4
CRISP1	rs150372078	ms	P54107	71	A/T	0,964	0,014500	0,924	13	87
CRISP2	rs781375574	ms	P16562	176	M/V	-0,824	0,012522	0,000	20	0,4
CRNN	rs780465907	ms	Q9UBG3	363	S/R	-1,140	0,003788	0,649	17	0,3
CRY1	rs749506981	ms	Q16526	348	R/C	-0,838	0,010967	0,984	15	0,8
CRYBG1	rs1676015	ms	A0A0J9YWL0	1853	T/S	0,404	0,021599	0,353	42	6642
CSH1	rs1426728052	ms	P0DML2	124	A/D	0,438	0,00008572	0,251	153	0,4
CSH2	rs1439972034	ms	P0DML3	124	A/D	0,438	0,00008558	0,010	153	0,3
CSHL1	rs149910727	ms	Q14406	98	T/M	-0,419	0,043080	0,171	46	5,3
CSMD1	rs552956828	ms	Q96PZ7	2464	R/L	-0,455	0,034290	0,300	34	1,1
CSMD1	rs571113460	ms	Q96PZ7	1485	P/L	0,519	0,038605	0,985	32	2,5
CSMD1	rs571852185	ms	Q96PZ7	167	I/M	-1,032	0,015289	0,845	10	0,8
CSN1S1	rs201502829	ms	P47710	4	L/P	-1,057	0,007322	0,568	13	15
CSN3	rs757594677	ms	P07498	118	I/T	0,626	0,013363	0,131	19	0
CSPG4	rs142305247	ms	Q6UVK1	660	R/Q	0,593	0,049003	0,046	23	82
CSPG4	rs144427836	ms	Q6UVK1	657	V/M	0,593	0,049003	0,905	23	88
CSRP3	rs759269008	ms	P50461	145	F/S	0,731	0,035730	0,815	12	0,3
CST3	rs776109203	ms	P01034	8	P/R	-0,635	0,003369	0,000	37	0,6
CSTF2T	rs1564612476	ms	Q9H0L4	118	P/S	0,642	0,025865	0,042	22	0
CTAGE15	rs1444511885	ms	A4D2H0	148	C/Y	0,907	0,024606	0,013	11	0
CTC1	rs778742090	ms	Q2NKJ3	86	S/L	-0,511	0,016442	0,514	41	2,6
CTC1	rs780355193	ms	Q2NKJ3	744	R/H	0,586	0,003944	0,711	38	0,7
CTDSP1	rs547460809	ms	Q9GZU7	48	R/W	0,670	0,012971	0,457	22	1,5
CTNND1	rs767264268	ms	O60716	34	R/G	-0,443	0,018404	0,978	43	7,6
CTNS	rs759156059	ms	O60931	77	V/L	0,600	0,007065	0,000	29	0,4
CTSV	9:97036711:C/A	ms	O60911	145	G/C	-0,765	0,029408	1,000	12	N/A
CUX1	7:102111745:C/G	ms	P39880	193	A/G	0,696	0,031200	0,178	13	N/A
CUX1	rs761867945	ms	P39880	436	R/H	-0,529	0,040149	0,963	25	0,4
CUX2	rs201097767	ms	O14529	1242	G/R	0,514	0,020960	0,106	35	21
CYB561D1	rs368199735	ms	Q8N8Q1	152	R/W	0,583	0,030487	0,434	25	0,8
CYBC1	rs142011515	ms	Q9BQA9	129	R/W	-0,802	0,036527	0,999	11	11
CYFIP2	rs766783609	ms	Q96F07-2	323	A/T	-0,654	0,020488	0,005	20	0,1
CYP2C9	rs772291418	ms	P11712	202	N/D	0,470	0,018604	0,098	60	0
CYP2D6	rs77578877	ms	P10635	185	V/L	0,791	0,011583	0,096	30	18
CYP4A22	rs369829008	ms	Q5TCH4	17	G/R	0,537	0,040217	0,250	23	2,6
CYP8B1	rs35764459	ms	Q9UNU6	238	K/R	-0,448	0,023120	0,101	31	2505
DACT1	rs771524719	ms	Q9NYF0-2	564	V/I	-0,694	0,016292	0,170	19	1,1
DAGLA	rs778280333	ms	Q9Y4D2	768	A/V	-0,719	0,025970	0,000	12	6,0
DBNL	rs139647339	ms	Q9UJU6	91	D/H	-0,427	0,040463	1,000	41	90
DBT	rs185492864	ms	P11182	301	R/C	-0,474	0,004526	0,861	49	23
DCAF7	rs745412598	ms	P61962	41	E/G	0,511	0,021338	0,979	30	0,2
DCAF7	rs780790401	ms	P61962	154	Q/H	0,498	0,030969	0,003	38	0
DCLK1	rs145262053	ms	O15075	711	A/V	-0,379	0,031671	0,000	46	382
DCTN1	rs774489951	ms	Q14203	992	E/G	1,005	0,009554	0,242	11	0
DDX42	rs117181531	ms	Q86XP3	754	S/I	0,429	4,482E-09	0,114	317	180
DDX5	rs559742081	ms	P17844	461	R/H	0,496	0,001674	0,887	61	3,0
DDX5	rs758505488	ms	P17844	75	T/P	0,356	0,047379	0,374	35	0,1
DDX50	rs148713144	ms	Q9BQ39	132	R/C	0,366	0,017729	0,000	80	116
DDX54	rs761117277	ms	Q8TDD1	765	R/P	0,545	0,032962	0,540	26	0,2
DDX56	rs1562584773	ms	Q9NY93	285	T/I	-0,663	0,050008	0,148	12	0
DDX60	4:168221750:T/C	ms	Q8IY21	1652	I/M	0,695	0,034865	0,003	13	N/A
DEFB1	rs140503947	ms	P60022	35	Y/C	0,847	0,036104	0,981	12	3,3
DEFB106A	8:7825178:T/C	start_lost	Q8N104	1	M/T	0,627	0,020502	0,044	18	N/A
DEFB110	rs770964208	ms	Q30KQ9	55	Y/C	-0,387	0,014267	0,622	62	0
DEFB4A	8:7896588:C/A	ms	O15263	58	T/K	0,867	0,016606	0,000	13	N/A
DEFB4B	rs756098185	ms	O15263	58	T/K	0,672	0,008552	0,000	22	5,0
DENND1A	rs745972611	ms	A0A0A0MS48	1064	K/R	-0,617	0,051954	0,003	13	0,7
DENND1C	rs760319460	ms	Q8IV53	751	P/S	-0,964	0,008908	0,511	11	1,3
DENND2D	rs201272881	ms	Q9H6A0	420	R/Q	-0,530	0,010901	0,352	24	19
DENND2D	rs145695008	stop	Q9H6A0	16	R/*	-0,626	0,010945	1,000	26	58
DENND4B	rs752750941	ms	O75064	1097	L/I	0,511	0,011794	0,903	32	0,4

DGKB	rs1355918054	ms	B5MBY2	34	E/G	-0,910	0,000213	0,074	34	0,8
DGKI	rs770128558	ms	A0A087WV00	132	S/L	0,558	0,024197	0,003	28	1,1
DHRS7	rs377160812	ms	Q9Y394	227	I/V	-0,385	0,032310	0,994	41	2,2
DHX34	rs116000939	ms	Q14147	159	R/Q	0,460	0,007256	0,023	57	321
DHX36	rs749710536	ms	Q9H2U1	137	E/K	-0,963	0,038784	0,010	11	0,4
DHX40	rs141945224	ms	Q8IX18	713	R/C	-0,365	0,002525	0,349	148	15
DHX57	rs745664385	ms	Q6P158	408	V/I	-0,361	0,032686	0,998	62	7,0
DHX9	rs752226276	ms	Q08211	1223	R/Q	-0,504	0,046347	0,001	28	0,6
DIP2C	rs746715099	ms	Q9Y2E4	77	R/Q	0,556	0,007849	0,018	32	4,2
**DISC1**	rs138886515	ms	Q9NRI5	470	E/K	-0,488	0,049751	0,925	25	2,6
**DISC1**	rs79978593	ms	Q9NRI5	13	A/G	0,863	0,028379	0,000	17	269
DLX2	rs1422322241	ms	Q07687	22	T/R	0,744	0,023351	0,031	12	0,3
DLX5	rs148894146	ms	P56178	238	H/N	-0,518	0,013816	0,006	26	14
DMAC1	rs765696614	ms	Q96GE9-2	108	A/T	-0,996	0,011941	0,911	15	0,4
DMGDH	rs138871430	ms	Q9UI17	643	K/E	0,390	0,007524	0,029	65	73
DMRTC2	rs141103699	stop	Q8IXT2	346	R/*	0,409	0,023076	1,000	55	19
**DMXL2**	rs143811213	ms	H0YLM8	372	N/H	-0,652	0,030206	0,367	16	16
**DMXL2**	rs762703183	ms	H0YLM8	2895	S/C	-0,591	0,003875	0,322	44	5,0
DNAH10	12:123790015:T/C	ms	A0A669KB38	570	V/A	-1,378	0,008236	0,571	10	N/A
DNAH11	rs749125298	ms	Q96DT5	358	R/C	0,444	0,051080	0,000	23	3,0
DNAH14	rs761301731	ms	A0A804HLD3	3062	K/E	0,862	0,006152	0,290	18	0
DNAH17	rs775238626	ms	Q9UFH2	4432	R/H	0,593	0,020575	0,980	28	6,4
**DNAH3**	rs766110188	ms	A0A8V8TLI9	269	Y/D	0,936	0,027800	0,937	12	0,5
**DNAH3**	rs772685691	ms	A0A8V8TLI9	534	K/R	-0,674	0,019741	0,003	12	0,2
DNAH5	rs140700961	ms	Q8TE73	192	A/T	0,515	0,007894	0,068	42	25
DNAH6	rs200844717	ms	Q9C0G6	2666	S/Y	0,818	0,014248	0,997	19	129
DNAH7	rs189913342	ms	Q8WXX0	2595	L/Q	-0,903	0,013169	1,000	11	10
DNAJA4	rs777655450	stop	Q8WW22	284	R/*	-0,460	0,030013	1,000	39	0,9
DNAJC13	rs762566567	ms	O75165	1396	P/T	-0,563	0,007391	0,998	23	0,8
DNASE1	rs8176927	ms	P24855	2	R/S	0,618	0,026663	0,003	21	3914
DNER	rs772555280	ms	Q8NFT8	256	R/P	0,626	0,050652	0,554	21	0,4
DOC2B	rs548884378	ms	Q14184	4	R/Q	-1,129	0,019165	0,000	14	60
DOCK1	rs140799120	ms	A0A096LNH6	255	I/F	0,457	0,035335	0,342	33	0,4
DOCK6	rs763348704	ms	Q96HP0	615	F/L	-0,896	0,042099	0,994	16	9,8
**DOCK8**	rs202110964	ms	Q8NF50	249	D/N	-0,773	0,037826	0,529	11	10
**DOCK8**	rs373337056	ms	Q8NF50	1484	T/I	-0,735	0,048313	0,422	11	0,8
**DOCK8**	rs530454954	ms	Q8NF50	1766	R/Q	-0,765	0,051173	0,017	24	4,5
DOP1B	21:36263581:C/T	ms	Q9Y3R5	1784	S/L	1,174	0,024190	0,461	11	N/A
DPH6	rs143353998	ms	Q7L8W6	46	D/N	-0,592	0,047582	0,953	23	76
DPP10	rs752853823	ms	Q8N608	701	T/I	0,853	0,004632	0,106	21	0,2
DPP9	rs1309548680	ms	Q86TI2-2	692	R/W	0,521	0,018901	0,981	28	0,7
DPYSL4	rs757669088	ms	O14531	381	V/I	0,410	0,017294	0,266	54	0,8
**DROSHA**	rs748009983	ms	Q9NRR4	1102	P/A	0,412	0,043789	0,783	31	0,2
**DROSHA**	rs750540831	ms	Q9NRR4	250	R/Q	-0,663	0,033701	0,234	25	7,8
DSCAML1	rs549967851	ms	Q8TD84	1980	P/Q	0,544	0,030873	0,012	24	47
DSP	rs144465638	ms	P15924	1349	S/N	0,761	0,010453	0,005	20	2,0
**DSPP**	rs61738508	ms	Q9NZW4	268	G/W	-0,742	0,044153	0,999	12	1091
**DSPP**	rs61738509	ms	Q9NZW4	231	P/T	-0,743	0,044043	0,949	12	1091
DST	rs768885705	ms	A0A7P0T890	507	E/V	0,582	0,015948	0,890	50	0
DTNA	rs150147476	ms	Q9Y4J8-17	124	H/R	-0,409	0,019098	0,001	80	9,8
DUOX2	rs202111673	ms	X6RAN8	1425	Q/E	0,434	0,033503	0,006	37	6,0
DUSP4	rs1817800187	ms	Q13115	61	I/M	0,961	0,039400	0,859	10	0,1
DUSP8	rs755470031	ms	Q13202	75	R/C	-1,148	0,000238	0,846	10	0,4
DYNC1H1	rs771773704	ms	Q14204	3925	Q/E	0,687	0,006623	0,000	41	0,4
DYNC2H1	rs745928254	ms	Q8NCM8	1850	N/S	-0,553	0,016142	0,057	53	1,1
DYNC2I1	rs73167274	ms	Q8WVS4	923	P/Q	0,462	0,029006	0,987	44	238
DYNC2I2	rs778308337	ms	Q96EX3	467	K/Q	0,917	0,026003	0,046	15	1,3
DYRK1A	21:37505403:G/A	ms	Q13627-2	445	D/N	1,248	0,038008	0,140	11	N/A
DYSF	rs1559177530	ms	O75923-13	936	F/Y	0,823	0,036955	0,997	13	0
E2F7	rs765296391	ms	Q96AV8	643	G/S	0,352	0,036073	0,005	50	0,4
EBF4	rs1479421192	stop	Q9BQW3-2	568	Q/*	-0,549	0,038848	1,000	25	1,1
ECEL1	2:232486040:A/G	ms	O95672	205	I/T	-0,630	0,032974	0,907	11	N/A
EEFSEC	rs768235736	ms	P57772	566	A/T	0,588	0,014005	0,000	24	0,7
EFCAB11	rs147386709	ms	Q9BUY7	19	E/A	-0,797	0,044479	0,005	14	35
EFCAB12	rs759073143	ms	Q6NXP0	375	T/I	0,823	0,042306	0,998	10	0,8
EFCAB13	rs746496282	ms	Q8IY85	23	N/D	0,438	0,021181	0,000	49	0,1
EFCAB3	rs1567727823	ms	Q8N7B9	191	K/T	0,729	0,045934	0,139	16	0
EFCAB6	rs750687549	ms	Q5THR3	1458	D/N	0,553	0,033623	0,993	14	0,4
EFTUD2	rs760166112	ms	Q15029	245	H/R	0,744	0,023227	0,839	24	0,4
EGFL8	rs138835391	ms	Q99944	219	R/L	-0,405	0,010530	0,174	55	63
EGFR	rs771995749	ms	P00533	730	L/R	-0,472	0,037279	0,794	33	0,8
EHMT1	rs144871446	ms	Q9H9B1	246	R/Q	0,551	0,050310	0,090	29	108
EIF2AK3	rs752973018	ms	Q9NZJ5	779	N/S	0,608	0,009257	0,000	26	0,1
EIF5B	rs192772936	ms	O60841	616	R/Q	-0,461	0,012354	0,416	49	49
ELAPOR2	rs34772926	ms	A8MWY0	605	R/H	-1,099	0,034969	0,146	12	46
ELK3	rs138659793	ms	P41970	315	A/V	-0,385	0,041795	0,685	35	5,3
**ELL**	rs139501652	ms	P55199	243	D/N	-0,659	0,007326	0,404	28	165
**ELL**	rs201725032	ms	P55199	424	R/C	0,372	0,027766	0,003	63	91
ELMO1	rs765065086	ms	Q92556	331	A/T	-1,249	0,037612	1,000	11	0
ELN	rs139718810	ms	P15502-2	162	G/S	0,493	0,039395	0,000	27	3,0
ELP2	rs148576942	ms	Q6IA86	618	T/R	0,367	0,002537	0,868	98	7,6
EMID1	rs201794555	ms	Q96A84-3	129	R/Q	-1,117	0,026225	0,263	16	1,1
EMILIN2	rs781538463	ms	Q9BXX0	40	P/R	0,560	0,031525	0,778	14	147
**ENGASE**	rs753581791	ms	Q8NFI3	334	R/G	-0,435	0,028193	0,999	66	1,7

ENPP7	rs144007702	ms	Q6UWV6	238	R/S	-0,651	0,012946	0,007	39	0
ENPP7	rs1568484654	ms	Q6UWV6	18	P/L	-0,964	0,031065	0,000	14	0
EP300	rs142823793	ms	Q09472	543	M/V	-0,824	0,018030	0,295	15	1,9
EPC2	rs770290421	ms	Q52LR7	98	K/Q	0,477	0,007762	0,995	37	0
EPDR1	rs149095633	ms	Q9UM22	121	P/L	0,363	0,039689	0,280	51	80
EPG5	rs778147277	ms	Q9HCE0	502	V/E	0,623	0,029146	0,975	11	0
EPHA1	rs780968060	ms	P21709	233	P/T	-0,641	0,051975	0,090	18	0
EPHA3	rs1559602030	ms	P29320	187	G/D	-0,594	0,006089	0,999	38	0
EPHA7	rs756964921	ms	Q15375	552	Q/R	0,836	0,016201	0,772	14	1,5
EPHB3	rs749686296	ms	P54753	970	V/I	0,690	0,040025	0,674	19	0,4
EPHB3	rs779216599	ms	P54753	436	P/L	1,433	0,005975	0,548	13	0,2
EPHB4	rs1562967203	ms	P54760	791	A/S	-0,783	0,038120	0,999	11	0
EPPK1	rs371322120	ms	P58107	1687	T/M	0,371	0,026482	0,939	73	11
EPS8L2	rs1564981510	ms	A0A3B3ISQ4	721	A/V	-0,704	0,006751	0,000	19	0,8
EPS8L2	rs780112279	ms	Q9H6S3	62	M/L	-0,373	0,006073	0,262	88	13
EPX	rs758186180	ms	P11678	112	F/L	-0,392	0,002923	0,089	123	6,4
ERBB2	rs767813285	ms	P04626	964	E/Q	0,514	0,004527	0,708	47	0,8
ERBB3	rs769634421	ms	P21860	94	F/V	-0,781	0,009530	0,628	16	0,7
ERCC1	rs771438243	ms	P07992	152	Y/C	0,572	0,00003741	0,218	97	1,1
ERCC5	rs1882915924	ms	P28715	889	L/F	-0,874	0,011984	0,540	14	4,8
ERCC8	rs770585176	ms	Q13216	379	S/T	0,600	0,046391	0,000	14	0,1
ERFL	rs776261803	ms	A0A1W2PQ73	117	F/L	-0,389	0,044303	0,096	48	0
ERN1	rs374518749	ms	O75460	374	K/Q	-0,368	0,008824	0,199	64	7,6
ERV3-1	rs1259230422	ms	Q14264	514	R/C	-0,659	0,036640	0,537	13	6,7
ERVV-2	rs191385202	ms	B6SEH9	526	F/L	-0,601	0,045985	0,494	25	44
ESYT3	rs200676435	ms	A0FGR9	236	G/E	-0,416	0,003008	0,976	70	3,8
ETNPPL	rs143646148	ms	Q8TBG4	296	A/G	0,533	0,047569	0,855	11	149
ETV2	rs780706581	ms	O00321	112	A/T	-1,176	0,005721	0,193	21	15
ETV5	rs1281944233	ms	P41161	482	P/L	1,242	0,017184	0,998	13	0,4
ETV5	rs1467805143	ms	P41161	245	R/W	-1,061	0,007116	0,970	17	0,3
EVPL	rs199543579	ms	Q92817	1033	A/T	0,410	0,001503	0,013	98	27
EVPL	rs769457633	ms	Q92817	1119	R/H	-0,411	0,004464	0,924	95	1,3
EVX2	rs151290628	ms	Q03828	114	M/R	0,626	0,046649	0,502	16	6,0
EXD1	rs777472629	ms	Q8NHP7-3	535	V/E	0,417	0,049266	0,000	22	0,8
EXOC3L2	rs1216771253	ms	A0A1C7CYX0	645	R/G	0,652	0,037995	0,674	11	0,5
EXOC6	rs141813516	ms	Q8TAG9	89	K/N	-0,507	0,019405	0,138	25	19
EXOC6B	rs762944459	ms	Q9Y2D4	639	S/N	0,467	0,044997	0,983	27	0,2
EXPH5	rs762635762	ms	Q8NEV8	1969	D/N	0,822	0,000308	1,000	52	0,7
EXPH5	rs777861207	ms	Q8NEV8	431	A/S	-0,720	0,017973	0,040	16	0,8
EXTL1	rs760822796	ms	Q92935	208	R/Q	-0,994	0,014590	0,146	23	0,4
EXTL3	rs1563222520	ms	O43909	786	L/V	0,564	0,008993	0,438	34	0,4
F13B	rs764924610	ms	P05160	577	L/W	-0,511	0,017159	0,871	22	0,9
FAAP20	rs369043951	ms	Q6NZ36	164	D/E	-0,356	0,031282	0,385	63	3,0
FAM118A	rs777058813	ms	Q9NWS6	85	R/W	-1,027	0,007808	0,397	14	11
FAM120B	rs775947746	ms	Q96EK7	623	R/H	0,766	0,010501	0,994	31	2,6
FAM151A	rs374572716	ms	Q8WW52	37	R/Q	0,538	0,008563	0,022	38	23
FAM151A	rs768990252	ms	Q8WW52	71	T/S	-0,390	0,008306	0,421	75	0,4
FAM163B	rs555269190	ms	P0C2L3	136	G/R	0,850	0,007457	0,213	14	4,2
FAM171A2	rs570503128	ms	A8MVW0	128	R/W	0,601	0,039658	0,991	20	9,4
FAM200A	rs147653498	ms	Q8TCP9	126	L/V	0,525	0,037952	0,024	21	15
FAM20A	rs754357363	ms	Q96MK3	48	G/S	-0,643	0,000141	0,019	69	3,4
FAM228B	rs777345681	ms	P0C875	30	C/R	-0,394	0,027970	0,001	48	0,7
FAM237A	rs200894319	ms	A0A1B0GTK4	65	V/I	-0,416	0,013907	0,000	47	21
FAM72A	rs782341403	ms	Q5TYM6	27	A/P	-0,933	0,002435	0,161	20	2129
FAM83H	rs1336225373	stop	Q6ZRV2	1143	E/*	0,630	0,039507	1,000	11	0,8
FAM86B2	rs1470859049	ms	P0C5J1	10	E/K	0,415	0,014609	0,003	52	4,9
FAM91A1	rs776239012	ms	Q658Y4	281	L/M	-0,601	0,009923	0,963	23	3,4
FANCA	rs200220791	ms	O15360	292	H/D	-0,373	0,014696	0,000	75	16
FANCF	rs1055714341	ms	Q9NPI8	66	W/R	1,130	0,007984	0,694	10	0,7
FANCG	rs754927660	ms	O15287	480	L/F	-0,508	0,00004911	0,995	113	0,2
FANCM	rs550238354	ms	Q8IYD8	573	R/Q	-0,585	0,043229	1,000	27	0,8
FARSA	rs747427583	ms	Q9Y285	23	S/G	-0,533	0,011976	0,896	34	0,8
FASN	rs2228307	ms	P49327	1888	I/V	-0,767	0,019199	0,041	12	3017
FASN	rs760665615	ms	P49327	342	A/S	-0,767	0,019194	0,194	12	15
FASTKD2	rs771505646	ms	Q9NYY8	70	S/L	-0,535	0,047246	0,037	21	0
FAT1	rs774367320	ms	Q14517	4055	P/L	-0,384	0,010429	0,999	72	0,4
FAT2	rs149549832	ms	Q9NYQ8	2694	P/S	-0,864	0,039211	0,998	12	0,7
FAT3	rs188857169	ms	Q8TDW7	800	N/Y	-0,624	0,038320	0,912	23	16
FAT3	rs201524480	ms	Q8TDW7	1152	I/T	1,142	0,003821	0,479	12	29
FBLN2	rs200898109	ms	P98095-2	642	R/C	0,443	0,028885	0,897	33	4,5
FBN3	rs148024558	ms	Q75N90	371	M/T	0,475	0,046090	0,005	22	286
FBN3	rs149806821	ms	Q75N90	445	G/S	0,385	0,016567	0,998	56	26
FBN3	rs1568355723	ms	Q75N90	2535	P/S	-0,841	0,004318	0,996	26	0,4
FBXL5	rs374329849	ms	Q9UKA1	212	V/I	-0,401	0,022558	0,993	49	7,6
FBXO27	rs774999328	ms	Q8NI29	267	Y/C	0,673	0,027529	0,976	21	0,4
FBXW10	rs774691557	ms	Q5XX13	124	W/R	-0,496	0,004442	0,994	50	0,4
FBXW10B	rs1201781215	ms	O95170	15	R/H	-1,111	0,002331	0,003	12	0,8
FCGBP	rs150766794	ms	A0A087WXI2	872	Y/C	0,699	0,033889	1,000	17	99
FCGBP	rs376286887	ms	A0A087WXI2	1135	S/N	0,788	0,045419	0,015	11	5,3
FCRL4	rs769507733	ms	Q96PJ5	512	D/N	-0,466	0,017274	0,386	38	0
FCSK	rs764653879	stop	Q8N0W3	969	E/*	-1,058	0,032116	1,000	10	0,4
FEM1B	rs1567114340	ms	Q9UK73	181	N/D	0,530	0,011089	0,612	32	0
FER1L5	rs368841036	ms	A0AVI2	43	V/M	0,612	0,012861	0,738	27	5,5
FER1L6	rs1160334898	ms	Q2WGJ9	459	E/K	0,703	0,036451	0,080	25	1,0

FETUB	rs143600252	ms	Q9UGM5	335	Q/E	0,413	0,021014	0,081	39	1,1
FGF6	rs139049599	ms	P10767	120	I/V	-0,787	0,005064	0,830	39	22
FGFBP2	rs142331717	ms	Q9BYJ0	101	R/H	-0,372	0,007183	0,000	87	86
FGFR2	rs536181987	ms	P21802	450	R/C	0,500	0,028125	0,672	30	5,7
FGGY	rs199980192	ms	Q96C11	17	V/I	1,017	0,020776	1,000	11	108
FHAD1	rs1419575009	ms	A0A804HIA4	1300	K/Q	0,844	0,015240	0,224	13	0,8
FHIP1A	rs775802962	ms	Q05DH4	437	K/N	0,369	0,033960	0,648	47	0,2
FIBIN	rs138273386	ms	Q8TAL6	96	R/H	-0,356	0,039715	0,957	56	316
FILIP1L	rs764581624	ms	H7C4M0	964	E/K	-0,957	0,009495	0,468	16	1,1
FKBP10	rs781768559	ms	Q96AY3	408	C/F	-0,669	0,002322	1,000	34	0,4
FLII	rs61741784	ms	Q13045	364	E/V	0,392	0,006232	0,165	97	429
FLNC	rs374847180	ms	Q14315	437	R/C	0,544	0,015231	0,953	37	4,9
FLYWCH1	rs200267787	ms	Q4VC44	387	R/Q	0,508	0,007436	0,003	50	39
FLYWCH1	rs748059598	ms	Q4VC44	528	R/Q	0,532	0,008791	0,978	25	0,4
FMN1	rs368779133	ms	Q68DA7-5	199	A/V	-0,391	0,033370	0,000	48	3,8
FMO2	rs145876121	ms	Q99518	438	E/K	-0,373	0,046652	0,336	43	69
FMO2	rs772086521	ms	Q99518	439	L/F	1,258	0,004932	0,971	15	4,2
FNDC1	rs774630537	ms	Q4ZHG4	165	R/C	0,469	0,027246	0,685	33	3,0
FNDC7	1:108730742:A/G	ms	Q5VTL7	565	I/V	1,349	0,025181	0,000	10	N/A
FNDC7	rs3006870	ms	Q5VTL7	367	N/S	-0,862	0,028966	0,483	15	9237
FOXD1	rs917127030	ms	Q16676	356	A/G	0,406	0,000325	0,000	127	37
FOXD4L5	rs1404314114	ms	Q5VV16	343	R/S	0,658	0,049006	0,234	12	0,4
FOXI2	rs1377314581	ms	Q6ZQN5	53	Y/C	-0,395	0,023236	0,000	55	0
FOXI3	rs745918234	ms	A8MTJ6	369	S/G	-0,773	0,021071	0,003	10	0,4
FOXQ1	rs773628191	ms	Q9C009	375	P/S	-0,588	0,041341	0,960	25	30
FRAS1	rs748159035	ms	Q86XX4-2	3585	A/T	-0,363	0,003615	0,001	105	0,8
FRAS1	rs774824172	ms	Q86XX4-2	644	G/R	-1,211	0,009411	0,830	12	0,7
FREM1	rs201154402	ms	Q5H8C1	441	D/N	0,846	0,033524	0,599	11	13
FREM1	rs760645412	ms	Q5H8C1	1529	L/V	-0,608	0,045681	0,001	14	1,1
FRMPD2	rs760390374	ms	Q68DX3	666	H/N	0,640	0,047273	0,001	15	0,4
FSIP2	rs111265848	ms	Q5CZC0	568	Y/F	1,725	0,000945	0,000	12	132
FSIP2	rs113773415	ms	Q5CZC0	3988	S/F	1,725	0,000945	0,000	12	63
FTCDNL1	rs1559218391	ms	E5RQL4	135	E/K	0,571	0,010084	0,000	38	0
FTSJ3	rs146889707	ms	Q8IY81	356	S/P	0,413	0,009661	0,312	88	62
FTSJ3	rs747668148	ms	Q8IY81	138	L/V	0,408	0,002506	0,999	90	1,7
FTSJ3	rs760234926	ms	Q8IY81	776	R/Q	-0,497	0,030306	0,968	34	0,8
FTSJ3	rs765433762	ms	Q8IY81	626	E/K	-0,585	0,048324	0,007	21	0,8
FTSJ3	rs767462503	ms	Q8IY81	494	R/Q	0,613	0,018297	0,000	23	1,5
FUCA1	rs768929138	ms	P04066	73	G/A	-0,719	0,029409	0,273	22	0,8
FXYD3	rs202118232	ms	S4R445	48	R/G	-0,694	0,042966	0,000	11	27
FYB1	rs768403381	ms	O15117-2	533	Q/P	0,511	0,009373	0,997	29	0
FYB2	rs755707826	ms	Q5VWT5	558	I/T	0,501	0,022191	0,110	22	0,2
GAB4	rs61740195	ms	Q2WGN9	414	E/D	0,646	0,025323	0,224	18	83
GABRD	rs139300921	ms	O14764	220	R/C	-0,378	0,000311	0,741	150	70
GABRG3	rs375624145	ms	Q99928	395	V/I	0,552	0,000925	0,009	53	4,2
GAD1	rs45566933	ms	Q99259	228	I/L	0,539	0,045535	0,062	33	48
GANC	rs370258614	ms	Q8TET4	583	V/M	-0,644	0,050599	0,788	15	7,0
GARIN1A	rs879682991	ms	Q6NXP2-2	106	A/T	-0,779	0,048088	0,273	13	3,4
GARIN4	rs780034561	ms	Q8IYT1	387	M/T	-0,403	0,018124	0,000	57	1,0
GBP3	rs139129340	ms	Q9H0R5	163	N/S	1,332	0,010678	0,074	12	99
GBP3	rs760485167	ms	Q9H0R5	432	C/R	-0,962	0,020912	0,000	14	0,8
GBP7	rs772256061	ms	Q8N8V2	283	E/Q	-0,416	0,046400	0,124	47	0,4
GCKR	rs545938878	ms	A0A0C4DFN2	612	R/L	-1,565	0,000795	0,455	13	0,4
GCKR	rs755683893	stop	A0A0C4DFN2	610	Q/*	0,493	0,043509	1,000	42	2,6
GEMIN2	rs762142842	start_lost	O14893-5	1	M/L	0,399	0,021143	0,000	70	1,1
GEMIN5	rs749935126	ms	Q8TEQ6	333	H/R	-0,939	0,027477	0,994	22	0,7
GFRA4	rs148812242	ms	Q9GZZ7-2	8	A/V	-0,432	0,042235	0,009	37	7,2
GGA3	rs751312773	ms	Q9NZ52	71	A/T	0,564	0,046101	0,999	20	0,6
GGCT	rs539867008	ms	O75223-4	109	G/E	0,409	0,026381	0,000	65	55
GGN	rs1375969871	ms	Q86UU5	464	P/T	0,868	0,041273	0,000	11	0
GGTLC2	rs146282307	ms	Q14390	107	S/L	-0,556	0,049955	0,710	21	17
GH1	rs61762497	ms	P01241	82	E/D	-0,393	0,036652	0,005	58	9,8
GH2	rs199558095	ms	P01242	103	R/C	-0,429	0,000203	1,000	129	11
GH2	rs16947229	ms	P01242-2	208	G/E	0,449	0,00008001	0,000	141	4,2
GIMD1	rs769087374	ms	P0DJR0	60	R/H	0,383	0,015758	0,711	67	3,4
GIN1	rs759917916	ms	Q9NXP7	491	T/M	0,655	0,004077	0,019	31	3,4
GINS1	rs137901350	ms	Q14691	83	R/C	-0,376	0,024916	1,000	52	33
GJB5	rs116644255	ms	O95377	22	R/C	0,509	0,043272	0,999	20	88
GLG1	rs752528979	ms	Q92896	393	S/L	0,536	0,016260	0,173	24	0,8
GLI1	rs1565600997	ms	P08151	516	G/C	0,771	0,010149	0,425	11	0
GLO1	rs777974212	ms	Q04760	105	W/R	-0,583	0,000106	1,000	66	0
GLYATL1B	rs112516845	ms	A0A0U1RQE8	244	G/A	-0,428	0,040621	0,000	28	2,6
GMPPA	rs772485317	ms	Q96IJ6	200	R/H	0,595	0,027245	0,003	14	2,6
GMPR2	rs1566680407	stop	Q9P2T1	91	Q/*	-0,889	0,007035	1,000	15	0
GNA13	rs1062597	ms	Q14344	221	V/L	-0,520	0,000001066	0,044	153	220
GNAT2	rs1189352767	ms	P19087	254	C/R	-1,066	0,037318	1,000	12	0,2
GNE	rs751107200	ms	Q9Y223-2	14	Q/E	0,565	0,005558	0,000	46	0,1
GOLGA2	rs16912752	ms	A0A8J9BZL8	456	M/V	1,206	0,021948	0,006	10	10362
GOLGA8H	rs767587258	ms	P0CJ92	141	Q/E	-2,113	0,002525	0,003	16	0,8
GOLGA8M	rs563033964	ms	H3BSY2	136	Q/R	-0,507	0,044758	0,226	19	159
GOLGA8M	rs576118690	ms	H3BSY2	136	Q/K	-0,507	0,044758	0,022	19	55
GOLGA8S	rs777558701	ms	H3BPF8	635	R/G	-0,818	0,005821	0,984	10	0
GOLGA8T	rs1160569396	ms	H3BQL2	324	A/S	0,478	0,030840	0,011	33	0
GON4L	rs756015284	ms	Q3T8J9	1821	P/S	-1,091	0,007105	0,007	12	1,1

GON4L	rs774683992	ms	Q3T8J9	1105	L/P	-0,442	0,033314	0,116	43	1,5
GORASP1	rs145676119	ms	Q9BQQ3	302	V/I	0,602	0,000523	0,992	57	39
GOT2	rs1567487563	ms	P00505	225	Q/E	-0,770	0,036597	0,416	15	0
GP1BA	rs756370087	ms	P07359	222	P/A	1,948	0,011850	0,874	12	0,4
GP6	rs779914827	ms	Q9HCN6-3	360	R/Q	-0,716	0,051903	0,773	14	0
GPATCH8	rs118151586	ms	Q9UKJ3	957	R/W	-0,448	0,016902	0,000	67	179
GPLD1	rs61754637	ms	P80108	103	N/S	-0,752	0,012543	0,786	13	125
GPR107	rs1564670898	ms	Q5VW38-2	265	L/V	0,511	0,026339	0,144	19	0
GPR108	rs368266462	ms	Q9NPR9	308	L/F	0,424	0,030025	0,358	39	3,0
GPR148	rs1332617530	ms	Q8TDV2	210	Q/H	-0,606	0,016837	0,003	19	0,8
GPR162	rs141534197	ms	Q16538	380	R/W	0,729	0,014786	0,349	13	50
GPR20	rs765239165	ms	Q99678	159	R/C	0,541	0,002123	0,041	44	1,9
GPR83	rs756043372	ms	Q9NYM4	51	D/G	0,469	0,013400	0,352	30	0,1
GPX1	rs769147194	ms	P07203-2	85	V/A	0,551	0,013208	0,001	44	1,0
GRAMD1B	rs200540342	ms	A0A1B0GUD6	507	V/I	0,414	0,00006618	0,162	147	156
GRB14	rs779123855	ms	Q14449	358	S/N	-0,820	0,017937	0,010	17	0,1
GRB7	rs758631692	ms	Q14451	18	C/Y	0,514	0,004529	0,000	47	2,3
GRB7	rs775378357	ms	Q14451	125	R/C	-0,433	0,035827	1,000	43	4,2
GREB1	rs142904474	ms	Q4ZG55	1464	N/D	-0,432	0,050712	0,999	35	77
GREB1	rs760195039	ms	Q4ZG55	465	R/H	0,841	0,031107	0,937	11	1,3
GREB1L	rs764201914	ms	Q9C091	1263	A/G	0,630	0,029345	0,095	18	32
GRIK1	rs73197503	ms	E7ENK3	450	I/T	0,571	0,039277	0,739	22	116
GRIK5	rs766852259	ms	Q16478	127	R/H	-0,892	0,010232	0,851	12	1,5
GRIP1	rs776702622	ms	Q9Y3R0	1081	V/L	-0,863	0,025966	0,978	23	0,4
GRM3	rs776290250	ms	Q14832	102	D/Y	-1,100	0,034914	1,000	12	0,2
GRPEL1	rs753276386	ms	Q9HAV7	25	R/Q	-0,477	0,002695	0,883	61	1,9
GSAP	rs763484733	ms	A4D1B5	142	L/R	-0,551	0,029210	0,999	44	0,1
GSC2	rs73390724	ms	O15499	205	C/S	-0,493	0,010473	0,267	45	253
GSE1	rs771372000	ms	Q14687	230	D/N	0,726	0,020826	0,000	12	0,4
GSG1	rs374196061	ms	A0A494C0G6	18	E/V	0,755	0,022384	0,965	15	5,3
GSR	rs151187899	ms	P00390	289	V/A	1,016	0,000033	0,987	29	148
GSR	rs200685394	ms	P00390	500	T/M	-0,451	0,037941	0,998	42	2,6
GSTK1	rs41275042	ms	Q9Y2Q3	161	T/M	-0,774	0,033967	0,717	16	34
GTF2IRD2B	rs1467815120	ms	Q6EKJ0	393	I/T	0,426	0,035733	0,999	34	0
GTF3C3	rs375124162	ms	Q9Y5Q9	802	R/Q	-0,402	0,004566	0,077	61	0,8
GTF3C5	rs150056568	ms	Q9Y5Q8	162	Q/K	-0,458	0,000002658	0,763	155	77
GTF3C5	rs995906811	ms	Q9Y5Q8	248	R/W	-0,623	0,048089	0,806	15	0,5
GULP1	rs777660706	ms	Q9UBP9	206	S/N	0,637	0,009319	0,000	22	0,1
GYG1	rs140175164	ms	P46976	147	N/D	-0,467	0,031398	0,189	38	100
H2AC1	rs1159899764	ms	Q96QV6	63	I/V	-1,070	0,011965	0,000	11	1,4
H2AC11	rs765983355	ms	P0C0S8	129	G/D	0,713	0,040453	0,010	19	0,2
H2AC16	rs190008233	ms	P0C0S8	83	H/R	-0,462	0,012271	0,511	40	8,2
H2AC4	rs139976301	ms	P04908	74	N/K	-0,793	0,031548	0,999	11	12
HADHA	rs772164983	ms	P40939	354	F/S	1,133	0,014997	0,653	11	0
HAL	rs137949606	ms	P42357	165	G/S	0,462	0,047890	0,998	21	14
HAL	rs1565990642	ms	P42357	343	G/V	1,008	0,030083	0,999	11	0,1
HAP1	rs142535684	ms	P54257-2	187	P/H	0,471	0,016691	0,127	46	113
HAPLN4	rs771835797	ms	Q86UW8	210	N/K	-0,443	0,037575	0,995	39	1,5
HCRTR1	rs199680510	ms	O43613	197	R/L	-2,238	0,000139	0,000	11	4,2
HDAC4	rs745530862	ms	A0A7I2SVS4	362	G/S	-1,438	0,002058	0,042	15	2,5
HDAC4	rs757619942	ms	A0A7I2SVS4	185	A/V	-0,385	0,016945	0,814	61	5,7
HDAC7	rs369848964	ms	Q8WUI4-5	681	A/T	0,654	0,047512	0,018	13	9,9
HDAC9	rs199944204	ms	Q9UKV0-7	167	G/A	0,621	0,020500	0,998	28	0,8
HEATR1	rs148574750	ms	Q9H583	783	V/M	0,604	0,030069	0,001	13	3,0
HEATR4	rs761598455	stop	Q86WZ0	191	W/*	-0,738	0,015735	1,000	26	4,2
HECTD4	rs748066013	ms	A0A804HJX8	3726	P/L	0,705	0,028014	0,199	21	3,0
HEG1	rs201404346	ms	Q9ULI3	342	T/M	-0,526	0,020948	0,011	39	40
HELZ	rs369453596	ms	P42694	1480	N/S	-0,636	0,006468	0,000	41	4,5
HELZ	rs777941893	ms	P42694	625	T/S	-0,475	0,019221	0,014	51	0,4
HELZ2	rs201125994	ms	A0AAA9XBX5	824	G/S	-0,369	0,017372	0,009	68	6,8
HERC2	rs149493788	ms	O95714	876	A/V	0,811	0,039166	0,019	10	11
HERC6	rs374213974	ms	Q8IVU3	545	Q/E	-0,594	0,027431	0,007	16	0,4
HERPUD2	rs201373686	ms	Q9BSE4	104	H/Y	-0,973	0,036842	0,025	17	0,8
HGS	rs34868130	ms	O14964	400	E/D	-0,767	0,019232	0,003	13	1360
HIGD2A	rs752348331	ms	Q9BW72	47	P/L	-0,436	0,014798	0,981	51	0,4
HIKESHI	rs769149509	ms	Q53FT3	86	G/D	-0,515	0,003007	0,600	49	0,7
HIP1R	rs748671805	ms	O75146	809	R/H	-0,621	0,024931	0,993	25	2,3
HJURP	rs146575864	ms	Q8NCD3	585	D/N	-0,436	0,036637	0,989	28	45
HLTF	rs749916690	ms	Q14527	320	I/N	0,611	0,023301	0,003	20	0,4
HLTF	rs769261535	ms	Q14527	617	T/K	0,509	0,007557	0,770	47	8,3
HMBS	rs189159450	ms	P08397	22	R/C	0,651	0,024626	0,983	18	2,3
HMCN1	rs771301411	ms	Q96RW7	2028	P/S	0,714	0,017879	0,259	15	0,2
HMCN2	rs1564815835	fs	Q8NDA2-5	1945	V/X	-0,683	0,038865	1,000	23	0
HNF1B	rs140562402	ms	P35680	82	D/N	0,374	0,044434	0,539	61	76
HNF1B	rs141193981	ms	P35680	532	M/V	-0,761	0,002714	0,978	26	17
HNRNPA1L3	rs756869693	ms	A0A2R8Y4L2	41	T/M	0,646	0,000441	0,163	47	6,4
HOOK1	rs1011037152	ms	Q9UJC3	16	D/E	0,357	0,037369	0,011	45	1,1
HOXB13	rs778047967	stop	Q92826	126	E/*	-0,572	0,000410	1,000	72	0,4
HOXB3	rs200264312	ms	P14651	284	P/S	0,365	0,040866	0,000	53	15
HRNR	rs1158627978	ms	Q86YZ3	1893	H/N	0,354	0,048229	0,000	38	0,7
HRNR	rs374494412	ms	Q86YZ3	807	Y/D	-0,671	0,033062	0,000	11	4,2
HRNR	rs764209221	ms	Q86YZ3	853	S/T	-0,671	0,033062	0,000	11	7,4
HSD17B1	rs61738799	ms	P14061	62	T/R	-0,360	0,038249	0,041	62	0,8
HSP90B1	rs144242572	ms	P14625	293	M/V	-0,549	0,013010	0,000	48	134

HUNK	rs1349105042	ms	P57058	675	R/C	-0,861	0,013189	0,993	14	1,5
HVCN1	rs749906449	ms	Q96D96	162	R/C	0,494	0,023462	0,153	23	0,4
HYAL4	rs117488620	ms	Q2M3T9	477	R/Q	-0,525	0,013421	0,000	53	285
HYAL4	rs143985361	ms	Q2M3T9	404	S/G	-0,628	0,012993	0,147	19	170
HYDIN	rs778831543	ms	Q4G0P3	4151	I/V	-0,511	0,016457	0,008	41	4,2
IAH1	rs768894310	ms	Q2TAA2	173	C/S	-0,845	0,002448	0,093	19	0,8
ICE1	rs199992370	ms	Q9Y2F5	1026	R/S	-0,639	0,033751	0,041	15	9,8
ICOSLG	rs759358911	ms	O75144	199	V/M	1,690	0,015019	0,915	13	3,2
IDUA	rs781136336	ms	P35475	526	L/P	0,762	0,015288	0,975	17	41
IFFO1	rs1027116185	ms	A0A087WZ16	188	S/L	-0,388	0,038344	0,024	60	0
IFNA5	rs769493970	ms	P01569	83	M/I	0,817	0,038985	0,183	19	0,2
IFNK	rs751052890	ms	Q9P0W0	3	T/I	0,753	0,004263	0,003	11	1,2
IFT172	rs149117098	ms	Q9UG01	1645	V/I	-0,393	0,050352	0,022	49	49
IFT56	rs772818942	ms	A0AVF1	162	Y/S	0,460	0,008833	0,952	60	1,9
IGHMBP2	rs147038490	ms	P38935	790	R/Q	0,717	0,039389	0,012	36	34
IGLL1	rs8138122	ms	P15814	189	R/H	0,657	0,022017	0,006	18	3692
IGSF22	rs191927164	ms	Q8N9C0-2	1036	V/L	0,770	0,010599	0,938	23	35
IL10RA	rs202121581	ms	Q13651	262	R/H	-0,493	0,017318	0,007	40	6,8
IL27RA	rs772182491	ms	Q6UWB1	118	V/I	-0,735	0,001623	0,001	28	2,6
IMMP1L	rs146915142	ms	Q96LU5	116	E/Q	0,905	0,021772	0,603	14	27
INMT	rs201737289	ms	O95050	110	A/V	0,391	0,041941	0,000	50	11
INO80	rs766550330	ms	Q9ULG1	327	K/R	-0,410	0,040996	0,040	41	0,8
INO80D	rs768886465	ms	Q53TQ3	689	G/E	0,720	0,022210	0,202	18	0,4
INSC	rs556785106	ms	Q1MX18-2	227	R/C	-0,404	0,044130	0,824	43	1,1
INSR	rs768172890	ms	P06213	690	E/V	-0,935	0,000131	0,000	30	0,8
INTS14	rs369418631	ms	Q96SY0	367	A/T	-0,996	0,035397	0,000	10	1,5
IPO4	rs747440936	ms	Q8TEX9	182	R/C	1,054	0,038898	0,870	12	0,7
IQCA1L	rs199771708	ms	A6NCM1	209	A/T	0,444	0,050860	0,978	42	147
IQCC	rs765596362	ms	Q4KMZ1	182	N/S	-0,756	0,039833	0,497	30	0,4
IQCD	rs60284450	ms	Q96DY2	19	R/K	-0,412	0,026384	0,005	52	1718
IQGAP2	rs759083181	ms	Q13576	786	K/R	-0,407	0,046218	0,597	49	0,2
IRF3	rs1568461744	ms	Q14653	101	H/D	-1,830	0,000084	0,841	11	0
IRS2	rs1046261311	ms	Q9Y4H2	446	G/S	-0,537	0,024641	0,005	24	3,4
ITGA11	rs368307734	ms	Q9UKX5	936	R/Q	-0,691	0,046327	0,000	15	3,4
ITGA6	rs778993295	ms	P23229-2	1019	L/P	0,603	0,036043	0,862	16	1,1
ITGAV	rs754174458	ms	P06756	428	R/L	-0,722	0,050363	0,000	10	2,3
ITGAV	rs781221559	ms	P06756	131	K/Q	-0,656	0,050991	0,007	15	0
ITPRID1	rs137892596	ms	Q6ZRS4	958	H/P	0,514	0,047501	0,009	25	15
ITSN1	rs1164637837	ms	Q15811	1588	R/H	-0,389	0,007964	0,149	78	0,4
JAK3	rs747131454	ms	P52333	651	R/W	-0,845	0,014988	0,050	16	0,4
JMY	rs759301275	ms	Q8N9B5	397	R/Q	-0,367	0,028494	0,358	45	0,4
JUP	rs374008304	ms	P14923	50	G/R	-0,979	0,045561	0,000	11	3,4
KANK1	9:738430:G/C	ms	Q14678	1160	G/A	0,707	0,033633	0,750	20	N/A
KANK4	rs377253083	ms	Q5T7N3	185	P/S	0,933	0,028470	0,001	11	6,6
KANSL1	rs151099014	ms	Q7Z3B3	765	A/V	0,494	0,026922	0,000	43	46
KANSL1	rs780685635	ms	Q7Z3B3	943	R/Q	-0,786	0,031409	0,932	11	0,1
KAT6A	rs1291997106	ms	Q92794	311	R/L	0,742	0,027106	0,979	15	0,8
KAT6B	rs1311062859	ms	Q8WYB5	1242	L/P	-0,992	0,030772	0,369	19	0,4
KAT8	rs185459113	ms	Q9H7Z6-2	446	R/Q	-1,639	0,026243	0,000	11	89
KCNA10	rs1159665771	ms	Q16322	407	S/N	-0,634	0,013252	0,982	26	0,2
KCNA3	rs779056951	ms	P22001	531	V/L	0,522	0,051738	0,014	39	0,4
**KCNH6**	rs148230267	ms	J9JID4	726	R/Q	0,380	0,000003736	0,000	258	11
**KCNH6**	rs369961665	ms	J9JID4	450	G/S	-0,440	0,000141	0,009	129	1,9
KCNJ16	rs761312168	ms	Q9NPI9	115	V/I	0,765	0,027167	0,988	11	3,8
KCNK13	rs200021633	ms	Q9HB14	148	R/H	-0,960	0,024121	0,998	13	0,8
KCNK5	rs41273124	ms	O95279	365	K/I	-0,554	0,033594	0,136	18	197
**KCNT1**	rs566157365	ms	Q5JUK3-3	657	P/L	0,467	0,014815	0,001	49	1,1
**KCNT1**	rs753401695	ms	Q5JUK3-3	674	M/V	1,002	0,007134	0,001	14	3,0
KCNU1	rs775599941	ms	A8MYU2	1121	P/L	1,014	0,051835	0,000	12	4,2
KCNV1	rs1035112026	ms	Q6PIU1	474	R/W	0,511	0,043188	0,476	20	0,2
KCNV2	rs147022958	ms	Q8TDN2	254	F/L	0,392	0,022353	0,036	52	0
KCTD13	rs1167342922	ms	Q8WZ19	40	S/N	-0,571	0,027479	0,059	25	0,4
KCTD20	rs748850127	ms	Q7Z5Y7	195	I/V	-0,472	0,017077	0,007	45	2,6
KCTD8	rs138935237	ms	Q6ZWB6	137	F/Y	-0,825	0,002433	1,000	30	23
KDM6B	rs748896517	ms	O15054-2	212	A/V	-0,419	0,027861	0,494	41	0,6
KDM8	rs145879289	ms	Q8N371	156	R/H	-0,724	0,037239	0,000	16	2,9
KIAA0232	rs780512122	ms	Q92628	1060	P/S	-0,476	0,002782	0,996	61	0,4
KIAA1671	rs6004404	ms	Q9BY89	1400	R/W	-0,449	0,016244	0,003	33	17
KIAA1755	rs202213297	ms	Q5JYT7	486	P/L	0,665	0,034246	0,370	17	4,7
KIF13B	rs200053841	ms	Q9NQT8	1812	D/N	-0,640	0,033592	0,015	20	49
KIF13B	rs761773190	ms	Q9NQT8	369	R/Q	-0,453	0,027397	0,943	56	2,3
KIF14	rs145426227	ms	Q15058	1207	Q/R	-0,375	0,031677	0,000	41	20
KIF14	rs771069928	ms	Q15058	1583	E/G	-1,025	0,009378	0,010	13	0
KIF15	rs112882527	ms	Q9NS87	938	V/I	-0,695	0,022159	0,003	18	73
KIF15	rs149445851	ms	Q9NS87	1120	E/G	0,790	0,031478	0,170	11	41
KIF15	rs776781117	ms	Q9NS87	842	V/I	-0,390	0,040532	0,000	36	3,8
KIF16B	rs759579752	stop	A0A1B0GVS8	1584	R/*	-0,395	0,040948	1,000	32	6,0
KIF16B	rs200695292	ms	Q96L93	1200	A/S	-0,787	0,048349	0,057	12	14
KIF16B	rs565686380	ms	Q96L93	1274	V/M	0,405	0,017903	0,784	52	0,7
KIF19	rs61746578	ms	Q2TAC6	891	R/W	-0,496	0,011660	0,891	40	1331
KIF24	rs556845604	ms	Q5T7B8	497	P/R	-0,480	0,032741	0,997	22	0,7
KIF26B	rs762051944	ms	Q2KJY2	463	R/Q	0,718	0,051657	0,052	17	0
KIF5C	rs749228157	ms	O60282	57	T/M	0,419	0,026273	0,958	48	0,4
KIF7	rs151317163	ms	Q2M1P5	746	R/Q	-0,610	0,019396	0,000	18	58

KIR2DL1	rs145961423	ms	P43626	141	A/T	0,537	0,043491	0,014	20	102
KIRREL1	rs762885869	ms	Q96J84	377	R/Q	0,427	0,033668	0,706	34	19
KLF18	rs768832850	ms	A0A0U1RQI7	969	E/K	-0,352	0,026052	0,017	72	1,1
KLHDC4	rs757939376	ms	Q8TBB5	432	R/H	0,677	0,007467	0,999	21	2,3
KLHL1	rs144867124	ms	Q9NR64	4	S/C	0,358	0,038566	0,996	50	44
KLHL14	rs143044789	ms	Q9P2G3	172	L/F	0,480	0,008325	0,005	58	1,1
KLHL18	rs374972341	ms	O94889	292	A/T	-1,039	0,004474	0,997	18	45
KLHL29	rs910946041	ms	Q96CT2	205	S/L	0,782	0,005359	0,028	22	1,1
KLHL5	rs758807194	ms	Q96PQ7-6	369	A/V	0,568	0,041489	0,224	19	0,6
KLHL5	rs767326522	ms	Q96PQ7-6	280	R/C	-0,636	0,043276	0,998	22	0,8
KLK14	rs1318729894	start_lost	Q9P0G3	1	M/K	0,578	0,037013	0,234	16	1,9
KLRC3	rs61743085	ms	Q07444	228	R/W	-0,874	0,044607	0,685	15	6,0
KMT2A	rs748830529	ms	Q03164-3	3146	Q/R	0,385	0,012663	0,163	54	0
KMT2D	rs747613578	ms	O14686	2207	A/V	-0,458	0,005881	0,000	72	16
KNL1	rs369348708	ms	Q8NG31-2	1148	I/V	0,417	0,049210	0,009	21	7,2
KPTN	rs142867197	ms	Q9Y664	63	R/Q	0,455	0,006389	0,048	66	137
KRI1	rs755712390	ms	Q8N9T8	50	D/V	-0,367	0,001517	0,473	102	3,8
***KRT**13*	rs144967807	ms	P13646	426	V/I	0,569	0,012923	0,000	41	104
KRT19	rs149987744	ms	P08727	327	T/M	0,645	0,033629	0,975	24	96
KRT23	rs1567799084	ms	Q9C075	183	D/G	0,539	0,027269	1,000	19	0,3
KRT31	rs1567681376	ms	Q15323	378	C/F	-0,564	0,048518	0,142	24	0
KRT32	rs117304287	ms	Q14532	99	T/I	0,695	0,017061	1,000	17	186
KRT7	rs138391469	ms	P08729	64	R/C	-0,436	0,044907	0,672	55	76
KRT71	rs749611724	ms	Q3SY84	205	E/G	-0,473	0,011448	0,987	42	0,4
KRT9	rs374710110	ms	P35527	326	M/I	0,386	0,008387	0,952	67	0,4
KRTAP4-12	rs368157227	ms	Q9BQ66	31	C/R	-0,747	0,031087	0,000	17	12
KRTAP4-8	rs777688954	ms	Q9BYQ9	176	R/H	-0,357	0,026763	0,000	73	2,9
KSR2	rs376902515	ms	Q6VAB6	373	A/T	-0,493	0,023456	0,000	32	11
L2HGDH	14:50267794:A/C	ms	Q9H9P8	341	F/L	0,739	0,032981	0,009	16	N/A
L3HYPDH	rs777937282	ms	Q96EM0	66	M/L	-0,648	0,029111	0,012	14	0,8
LACTBL1	rs780707524	stop	H0Y608	515	Q/*	-0,403	0,028944	1,000	53	34
LAMA4	rs767693296	ms	A0A0A0MQS9	229	Y/C	0,391	0,002163	0,419	124	0,3
***LAMB**1*	rs766731970	ms	P07942	445	Y/C	-1,216	0,042208	0,989	10	0,4
LAMB2	rs760185781	ms	P55268	37	P/L	-0,946	0,016490	0,000	16	0,2
LAMB2	rs760500807	ms	P55268	532	P/L	0,365	0,049489	0,134	44	0
LAMB3	rs747338921	ms	Q13751	907	A/D	-1,040	0,018506	0,791	12	0,7
LAMB3	rs767004520	ms	Q13751	552	R/C	0,594	0,041677	0,975	22	7,9
LAMB4	rs758145192	stop	A4D0S4	942	Q/*	0,691	0,010285	1,000	22	1,1
LAMB4	rs755389093	ms	C9JMJ0	712	Q/R	-0,670	0,000170	0,000	56	0
LAMC1	rs150421474	ms	P11047	1435	A/T	0,546	0,020550	0,000	19	41
LANCL2	rs758368934	ms	Q9NS86	88	M/V	-0,413	0,005007	0,052	58	0
LARGE2	rs376236828	ms	Q8N3Y3	426	P/S	0,517	0,002353	0,003	51	6,0
LARP1B	rs371480626	ms	Q659C4	748	H/Y	0,633	0,023153	0,811	16	0,4
LBR	rs773059198	ms	Q14739	310	G/R	0,464	0,018569	0,878	32	19
LCOR	rs1247398150	ms	Q96JN0	373	S/N	-0,662	0,030349	0,528	17	0,8
LDHAL6A	rs139641299	ms	Q6ZMR3	288	F/L	-0,905	0,033644	0,999	11	155
LDHB	rs746464388	ms	P07195	181	I/V	0,547	0,013969	0,059	27	0,1
LENG1	rs150820847	ms	Q96BZ8	176	S/R	0,563	0,051777	0,000	25	127
LGALS4	rs748499184	ms	P56470	41	E/Q	-0,935	0,027414	0,010	13	0
LIG4	rs756725710	ms	P49917	208	H/R	0,692	0,041797	1,000	22	0,3
LIPA	rs762796693	ms	P38571	4	R/Q	-0,508	0,051383	0,001	26	0,8
LIPF	rs17333991	ms	P07098	348	P/S	-0,439	0,017173	0,854	48	208
LIPG	rs748470810	ms	Q9Y5X9	212	D/N	0,537	0,000592	0,893	55	0,4
LIPH	rs1720462199	ms	Q8WWY8	43	L/V	0,826	0,051507	0,992	16	0,4
LLGL1	rs200829360	ms	Q15334	11	A/T	0,391	0,006363	0,000	97	265
LLGL1	rs761027899	ms	Q15334	329	E/K	-1,410	0,009989	0,001	11	2,3
LMF2	rs373075701	ms	Q9BU23	177	R/C	0,936	0,001848	1,000	19	12
LOC112267897	rs148363228	ms	A0A3B3IRQ3	89	G/A	0,492	0,026246	0,000	40	4849
LONRF1	rs200496434	ms	Q17RB8	457	V/I	0,660	0,045201	0,000	30	59
LOXHD1	rs754654876	ms	A0A2R8Y7K4	1572	R/Q	1,369	0,001659	0,918	14	0,8
LPCAT3	rs200922476	ms	Q6P1A2	331	T/I	-0,578	0,003930	0,409	38	12
LRP1	rs150485916	ms	Q07954	2015	V/I	0,531	0,040819	0,974	23	12
LRP1B	rs754422175	ms	Q9NZR2	104	P/L	0,788	0,001864	0,005	34	3,8
LRP2	rs1365452628	ms	P98164	3815	L/M	-0,657	0,030641	0,080	15	1,5
LRP2	rs140022108	ms	P98164	3726	R/C	0,751	0,040530	1,000	19	1,1
LRP6	rs773818278	ms	O75581	356	L/S	-0,749	0,003452	0,204	34	0,2
LRP8	rs139703435	ms	Q14114	725	M/R	-1,016	0,010003	0,246	10	31
LRP8	rs201950519	ms	Q14114	571	N/S	0,684	0,049347	0,993	15	20
LRPPRC	rs771539737	ms	P42704	1306	S/T	0,470	0,040782	0,009	43	0,1
LRRC25	rs772139017	ms	Q8N386	107	R/H	0,430	0,035373	0,031	32	1,5
LRRC37A	17:46297037:T/C	ms	A6NMS7	635	I/T	0,564	0,018485	0,012	32	N/A
LRRC37A2	17:46513049:T/C	ms	A6NM11	113	W/R	-0,695	0,039575	0,030	17	N/A
LRRC37A2	17:46514616:T/C	ms	A6NM11	635	I/T	0,678	0,006997	0,000	27	N/A
LRRC37A3	rs1463425308	ms	O60309	483	P/L	0,406	0,001153	0,998	95	1,2
LRRC37A3	rs200759066	ms	O60309	17	R/H	0,558	0,047344	0,370	17	5,4
LRRC37A3	rs77916588	ms	O60309	1130	V/I	-0,453	0,00004171	0,017	144	505
LRRC39	rs140255458	ms	Q96DD0	72	W/R	-0,443	0,021005	0,997	51	24
LRRC41	rs774231085	ms	Q15345-2	39	G/C	0,585	0,034917	0,742	17	12
LRRC46	17:47835758:T/C	ms	Q96FV0	122	I/T	-0,896	0,030450	0,990	14	N/A
LRRC46	rs146740911	ms	Q96FV0	242	V/M	-0,568	0,044504	0,142	26	55
LRRC66	rs201283430	ms	Q68CR7	11	I/V	-0,722	0,028597	0,030	19	2,0
LRRC7	rs144289156	ms	A0A494C1A4	1160	A/T	-0,451	0,015763	0,056	53	19
LRRFIP1	rs773197164	ms	Q32MZ4-4	314	T/A	0,495	0,044210	0,421	18	0,4
LRRFIP2	rs776740745	ms	Q9Y608	114	F/L	0,548	0,035700	0,000	18	2,6

LRRIQ1	rs143253287	ms	Q96JM4-4	835	S/G	0,615	0,047287	0,000	**23**	**96**
LRRN2	rs374611937	ms	O75325	577	R/Q	**-1,001**	0,002439	0,001	**22**	7,6
LSR	rs749613670	ms	S4R3V8	576	S/W	0,435	0,046716	**0,874**	**21**	0
LSR	rs771635414	ms	S4R3V8	579	D/N	0,409	0,002513	0,355	104	0,5
LTBP3	rs770492133	ms	Q9NS15	549	P/R	0,818	0,026933	0,001	**14**	0,7
LVRN	rs747264765	ms	Q6Q4G3	768	R/H	-0,674	0,025079	**0,986**	**19**	1,1
LY6E	rs199552296	ms	Q16553	81	V/I	-0,684	0,018000	0,003	**28**	**12**
LY75	rs201265039	ms	O60449	48	K/E	0,656	0,014886	0,084	**17**	0,4
LZTS3	rs1391065205	ms	O60299	645	G/R	-0,717	0,051715	0,251	**13**	0,4
LZTS3	rs759999966	ms	O60299	199	G/R	0,372	0,050002	**0,955**	**53**	1,4
MACF1	rs140978060	ms	H3BPE1	1734	A/V	-0,583	0,049995	0,162	**14**	**34**
MACIR	rs140904641	ms	Q96GV9	88	M/L	0,362	0,046181	0,000	**35**	0,7
MACROH2A2	rs1489682038	ms	Q9P0M6	22	I/V	0,557	0,036316	0,125	**18**	0,2
MAD1L1	rs200917713	ms	Q9Y6D9	696	R/H	0,512	0,001665	0,001	**50**	**74**
MADD	rs755791135	ms	A0A9L9PXF1	831	P/A	-0,672	0,007887	**0,999**	**31**	1,5
MAG	rs778697016	ms	P20916	624	R/Q	-0,598	0,015200	**0,645**	**21**	5,1
MAGEL2	rs1566783726	ms	Q9UJ55	944	S/N	**-1,094**	0,019026	0,020	**11**	0,9
MAGI2	rs145648453	ms	Q86UL8	1304	G/D	-0,778	0,003904	0,169	**16**	197
MAGI3	rs1557839712	ms	Q5TCQ9-4	300	E/A	0,626	0,030652	**0,999**	**16**	0,4
MAN2B2	rs142638040	ms	Q9Y2E5	975	R/H	-0,660	0,045838	0,001	**13**	**12**
MANSC1	rs370523906	ms	Q9H8J5	243	K/N	-0,679	0,031011	**0,532**	**15**	**16**
MANSC1	rs748098987	ms	Q9H8J5	301	A/T	0,378	0,018408	**0,717**	**71**	2,3
MAP1A	rs200211262	ms	P78559	1185	R/C	-0,525	0,050933	0,255	**25**	**18**
MAP3K1	rs764525244	ms	Q13233	504	E/K	**1,291**	0,031982	**0,447**	**14**	4,2
MAP3K13	rs199572011	ms	O43283	474	R/Q	0,958	0,020641	0,078	**13**	**12**
MAP3K19	rs56349597	ms	Q56UN5	1154	R/H	-0,458	0,035948	**0,997**	**46**	**63**
MAP3K21	rs767156199	ms	Q5TCX8	1029	E/Q	0,382	0,013969	0,003	**59**	1,2
MAP3K3	rs774603132	ms	Q99759	185	R/L	-0,912	0,048087	0,028	**11**	0,7
MAP6	rs770189526	ms	Q96JE9	701	P/S	-0,707	0,008969	0,180	**24**	0
MAPK3	rs375146669	ms	P27361	31	V/G	-0,383	0,022901	0,235	**67**	0,4
MAPK8IP2	rs1569064809	ms	Q13387	421	A/T	**-1,040**	0,027579	0,000	**18**	0,7
MAPK8IP3	rs201803478	ms	A0A087WYG2	1273	S/L	-0,496	0,035550	0,000	**27**	**33**
MARCHF10	rs140159918	ms	Q8NA82	123	S/N	0,357	0,000233	0,054	191	**14**
MARCHF10	rs200034579	ms	Q8NA82	309	C/Y	-0,422	0,003276	0,000	**87**	**39**
MARF1	rs1567589768	ms	Q9Y4F3	215	L/V	0,724	0,007106	**0,991**	**19**	0
MARF1	rs761713203	ms	Q9Y4F3	1631	S/L	-0,568	0,003283	**0,998**	**22**	0,8
MARK3	rs56157932	ms	P27448-5	468	A/V	0,679	0,024066	0,051	**15**	3,0
MBD1	rs142015383	ms	Q9UIS9	315	E/K	**1,077**	0,001920	**0,712**	**15**	**41**
MBD3	rs371220154	ins	O95983	278-279	-/E	0,793	0,005774	-	**26**	**3385**
MBLAC1	rs199848609	ms	A4D2B0	231	H/Q	0,516	0,031111	**0,995**	**20**	1,9
MBLAC2	rs147831525	ms	Q68D91	42	V/L	0,393	0,027521	0,009	**49**	**29**
MBTPS1	rs768417679	ms	Q14703	367	I/M	-0,480	0,039689	**0,872**	**22**	0,8
MBTPS1	rs776110165	ms	Q14703	332	M/I	-0,588	0,001421	**0,637**	**43**	5,3
MCC	rs75148264	ms	P23508-2	183	A/T	0,589	0,034567	0,007	**15**	197
MCEE	rs111033538	stop	Q96PE7	47	R/*	**1,215**	0,009228	**1,000**	**11**	**30**
MCF2L	rs145119738	ms	O15068-9	564	S/F	-0,602	0,030563	**0,796**	**25**	110
MCF2L2	rs773833576	ms	Q86YR7	376	K/E	**-1,063**	0,012535	0,215	**12**	0,4
MCM2	rs376214773	ms	P49736	198	R/H	-0,504	0,045674	**0,987**	**26**	1,5
MCM4	rs755560078	ms	P33991	487	G/R	-0,476	0,051773	**0,992**	**27**	0,8
MCM7	rs747875490	ms	P33993	455	R/H	0,681	0,018502	**0,998**	**19**	0,6
MCM8	rs764765085	ms	Q9UJA3	726	L/M	0,385	0,029704	0,037	**69**	0
MCM9	rs182607789	ms	Q9NXL9	882	H/R	0,586	0,020552	0,000	**28**	1,1
MCM9	rs185696063	ms	Q9NXL9	715	D/V	0,518	0,025751	0,213	**38**	166
MCM9	rs553491848	ms	Q9NXL9	563	R/Q	0,527	0,027734	0,151	**24**	5,6
MCMDC2	rs1563368437	ms	Q4G0Z9	165	I/M	0,420	0,042213	0,318	**35**	0
MCOLN1	rs760411989	ms	Q9GZU1	472	D/N	-0,542	0,043040	**0,988**	**15**	0,5
MCU	rs766307644	ms	Q8NE86	351	D/H	-0,792	0,039889	**0,993**	**14**	1,3
MDGA2	rs1566621686	ms	Q7Z553-3	377	Y/C	0,776	0,033505	**1,000**	**19**	0,4
MDK	rs750294696	ms	P21741	122	V/I	-0,543	0,044057	**0,673**	**32**	0,4
MDN1	rs1562203020	ms	Q9NU22	664	A/V	-0,722	0,028505	**0,832**	**15**	0,2
MECR	rs1558480060	ms	Q9BV79	110	A/V	-0,653	0,025026	**0,492**	**20**	0
MED13	rs35998015	ms	Q9UHV7	39	T/A	-0,584	0,033484	**0,896**	**26**	218
MED24	rs775765230	ms	O75448	789	P/S	0,662	0,015664	**1,000**	**31**	0,8
MED27	rs780440896	ms	Q6P2C8	211	R/Q	-0,447	0,051771	**0,560**	**24**	0,3
MED30	rs770113741	ms	Q96HR3	142	I/L	-0,479	0,048220	0,003	**43**	0,2
MEF2B	rs781653583	ms	Q02080-2	308	G/S	-0,512	0,045871	0,003	**27**	0,8
MEFV	rs104895112	ms	O15553-2	329	R/H	0,459	0,015755	0,000	**56**	124
MEIOC	rs745376874	ms	A2RUB1-4	934	H/R	0,768	0,026627	0,003	**11**	0,8
MELK	rs146537035	ms	Q14680	331	R/H	0,420	0,029377	0,031	**37**	**16**
MEP1B	rs751683259	ms	Q16820	511	R/C	-0,741	0,001473	**0,975**	**37**	0,8
MEX3D	rs949994849	del	Q86XN8	133	P/-	-0,378	0,017986	-	**64**	1,4
MFN2	rs387906991	ms	O95140	362	T/M	0,728	0,035929	**0,930**	**15**	4,2
MFSD5	rs142428878	ms	Q6N075	278	G/R	0,715	0,014290	**0,552**	**19**	**16**
MFSD5	rs751354324	ms	Q6N075-2	85	Y/F	-0,485	0,048668	0,003	**20**	0,6
MFSD6L	rs367887663	ms	Q8IWD5	323	H/D	0,505	3,462E-08	**0,902**	166	7,2
MGA	rs759572856	ms	A0A994J6L2	2622	L/V	-0,409	0,041401	**0,991**	**41**	0,8
MGAM	rs1563229730	ms	O43451-2	2634	I/T	-0,446	0,044981	**0,941**	**29**	0
MGAT4B	rs776812428	ms	Q9UQ53	495	R/W	0,543	0,049365	**0,850**	**21**	1,1
MIA2	rs147941751	ms	Q96PC5-3	569	S/P	0,579	0,045106	0,092	**14**	**33**
MICAL2	rs376168249	ms	O94851	740	R/H	0,667	0,035817	0,000	**16**	3,8
MICALL2	rs138933154	ms	Q8IY33	532	P/S	0,552	0,001273	0,002	**47**	3,0
MIER1	rs189050888	ms	Q8N108-12	460	S/P	-0,358	0,048007	0,370	**65**	181
MIER1	rs759562232	ms	Q8N108-12	158	D/Y	0,480	0,004103	**0,711**	**55**	1,1
MILR1	rs1352161075	ms	Q7Z6M3	159	T/I	0,376	1,217E-10	0,035	539	3,4

MIPOL1	rs749772798	ms	Q8TD10	306	N/S	-0,628	0,037037	0,337	15	1,1
MITF	rs542163629	ms	O75030	377	R/Q	-0,806	0,015633	0,413	18	2,6
MIXL1	rs1471523698	ms	Q9H2W2	49	P/S	0,648	0,049749	0,650	19	0
MKI67	rs61729196	ms	P46013	1059	A/E	0,514	0,015813	0,025	45	82
MKI67	rs1565001712	stop	P46013	2225	Q/*	1,181	0,011340	1,000	16	0,1
MKNK1	rs764595768	ms	A0A499FJN1	226	D/N	0,811	0,031747	1,000	21	1,5
MKS1	rs199832333	ms	Q9NXB0	40	H/Y	0,371	0,042693	0,034	49	59
MLLT3	rs767771641	ms	P42568	416	E/K	-0,501	0,037361	0,597	31	0,4
MLST8	rs766259271	ms	Q9BVC4	117	R/Q	0,840	0,048451	0,053	13	2,2
MLXIP	rs777847505	ms	Q9HAP2	161	R/Q	-0,402	0,029331	0,970	37	0,1
MMD2	rs761881427	ms	Q8IY49-2	172	P/L	-0,492	0,024161	1,000	33	0,1
MME	rs769414577	ms	Q3KQS6	77	R/G	0,919	0,015592	0,000	24	0,4
MMP21	rs554501102	ms	Q8N119	97	A/V	-1,789	0,002314	0,392	11	60
MMP3	rs148579119	ms	P08254	153	Y/H	0,449	0,004839	0,005	73	232
MMRN1	rs763071708	ms	Q13201	62	S/L	0,537	0,039639	0,006	13	0,4
MMRN1	rs745711366	stop	Q13201	177	R/*	-0,943	0,031835	1,000	16	0,8
MMRN2	rs200500870	ms	Q9H8L6	53	V/I	-0,373	0,011629	0,001	71	1,9
MN1	rs1568986531	ms	Q10571	618	F/L	-0,676	0,001428	0,079	57	0
MN1	rs200030766	ms	Q10571	19	G/D	0,440	0,026800	0,982	50	114
MN1	rs529726950	ms	Q10571	58	P/T	-0,756	0,040886	0,478	14	0,7
MOB3A	rs201880293	ms	Q96BX8	55	E/K	-0,499	0,031221	0,443	21	4,9
MPHOSPH9	rs147793766	ms	Q99550	447	T/A	-0,454	0,008938	0,037	56	254
MPIG6B	rs530881306	ms	O95866	173	P/Q	0,529	0,015135	0,003	27	1,3
MPP2	rs76832034	ms	D3DX48	261	I/M	0,484	0,042905	0,995	44	128
MRAP2	rs545003385	ms	Q96G30	106	R/H	0,433	0,010710	0,001	57	6,8
MRC1	rs368257559	ms	P22897	295	R/C	-0,494	0,002556	0,652	47	1,3
MRC2	rs146172137	ms	Q9UBG0	352	S/R	0,391	0,000009068	0,951	203	111
MRC2	rs201959743	ms	Q9UBG0	114	R/W	0,397	0,0001036	0,900	169	32
MRC2	rs749626570	ms	Q9UBG0	1054	M/I	-0,442	0,000327	0,007	118	0,7
MRC2	rs751681043	ms	Q9UBG0	1459	P/S	0,637	0,000527	0,154	36	0,4
MRC2	rs755074437	ms	Q9UBG0	547	R/H	-0,973	0,000005914	0,826	29	2,6
MRC2	rs776632141	ms	Q9UBG0	1439	R/C	-0,584	0,003967	0,998	30	3,0
MRGPRX3	rs749858617	ms	Q96LB0	55	R/H	-0,682	0,023600	0,078	10	5,4
MROH2A	rs770367514	ms	A6NES4	997	T/I	0,500	0,012736	0,007	39	0,1
MROH2B	rs563729861	ms	Q7Z745	868	A/T	-0,637	0,049621	0,990	26	5,3
MROH7	rs775705383	ms	Q68CQ1-7	623	D/V	-0,561	0,044513	0,029	27	1,7
MROH9	rs368219054	ms	Q5TGP6-2	179	C/W	0,475	0,047506	0,988	13	1,5
MRPL10	rs941871889	ms	Q7Z7H8-2	11	P/L	-0,792	0,000694	0,000	33	6,7
MRPL20	rs775815488	ms	Q9BYC9	126	E/K	1,240	0,006840	0,996	19	1,1
MRPL32	rs148103531	ms	Q9BYC8	18	G/E	0,357	0,037544	0,061	59	14
MRPL45	rs781545260	ms	A0A087X2D5	30	A/V	0,864	0,024341	0,024	11	1,2
MRPS12	rs760757324	ms	O15235	97	G/R	-0,357	0,018666	0,985	74	0,2
MS4A8	rs748203143	ms	Q9BY19	143	I/V	-0,392	0,003871	0,567	71	0
MSH3	rs778661757	ms	P20585	908	Q/P	0,367	0,033334	0,934	46	0,8
MSS51	rs372657650	ms	Q4VC12	242	R/Q	-0,408	0,038662	0,045	52	7,2
MST1R	rs149428633	ms	Q04912	778	N/T	-0,800	0,030054	0,007	19	140
MST1R	rs373976686	ms	Q04912	1305	R/H	-0,569	0,008189	0,999	50	3,0
MTAP	rs775627882	ms	Q13126	274	A/T	-0,486	0,004044	0,054	58	0,8
MTCL1	rs147496732	ms	A0A8Q3WKN6	728	R/C	1,525	0,003417	1,000	12	2,3
MTCL1	rs34690009	ms	A0A8Q3WKN6	879	M/V	-0,386	0,029320	0,423	49	1206
MTCL2	rs190032659	ms	O94964-2	1052	R/W	-0,428	0,022210	0,990	30	40
MTMR4	rs369444194	ms	A0A804HJV7	127	R/W	-0,514	0,000037	0,994	86	9,1
MTMR7	rs370669270	ms	Q9Y216	131	D/N	-0,629	0,009392	0,001	28	16
MTOR	rs748631718	ms	P42345	173	R/C	-0,939	0,002697	0,892	15	1,1
MTOR	rs778866134	ms	P42345	534	D/N	-0,620	0,000863	0,122	36	3,0
MTPAP	rs151258340	ms	Q9NVV4	152	K/M	0,494	0,005145	0,852	40	1,9
MTPAP	rs765614188	ms	Q9NVV4	170	P/A	0,950	0,000770	0,005	20	0
MTR	rs774231870	ms	Q99707	674	R/H	0,976	0,021926	0,999	12	0,4
MUC12	rs548320109	ms	Q9UKN1-2	1353	P/L	-0,569	0,005400	0,000	37	0,8
MUC16	rs142323715	ms	A0AAA9YHI4	13218	V/I	-0,773	0,049538	0,253	17	831
MUC16	rs200135063	ms	A0AAA9YHI4	6559	A/E	0,395	0,009397	0,000	78	123
MUC16	rs751898712	ms	A0AAA9YHI4	6623	T/R	-0,783	0,000263	0,000	39	0,7
MUC16	rs752215341	ms	A0AAA9YHI4	6148	V/F	0,530	0,022957	0,000	24	0,8
MUC16	rs768836638	ms	A0AAA9YHI4	1615	T/I	-0,725	0,048685	0,617	10	0,4
MUC16	rs998924452	ms	A0AAA9YHI4	7103	T/I	-0,783	0,000263	0,000	39	0,1
MUC17	rs377637063	ms	Q685J3	723	T/N	0,376	0,016549	0,000	50	6,8
MUC17	rs374623804	stop	Q685J3	2753	L/*	-0,459	0,041900	1,000	36	5,7
MUC20	rs115826755	ms	Q8N307	672	P/L	0,887	0,004812	0,000	14	130
MUC4	rs148587168	stop	Q99102	5215	R/*	0,521	0,019339	1,000	27	58
MUC5AC	rs1324038977	ms	P98088	1773	I/F	-0,826	0,049518	0,677	14	2,8
MUC5AC	rs1325110644	ms	P98088	3523	R/Q	0,862	0,002865	0,007	23	6,7
MUC5AC	rs1428522876	ms	P98088	3956	R/Q	0,862	0,002866	0,007	23	0,4
MUC5AC	rs1564913808	ms	P98088	2250	S/F	-0,829	0,045330	0,000	13	0
MUC5B	rs190158159	ms	Q9HC84	4893	T/R	0,432	0,016783	0,000	49	28
MUS81	rs1178001089	ms	Q96NY9	143	L/Q	0,464	0,041881	0,999	33	0,4
MYBL1	rs191885262	ms	P10243	365	I/T	-0,538	0,028764	0,489	33	6,0
MYBPC1	rs747245712	ms	Q00872-4	92	T/P	0,357	0,025341	0,951	89	0
MYBPC1	rs750820996	ms	Q00872-4	608	G/R	0,372	0,048947	0,415	62	2,3
MYBPC3	rs730880619	ms	Q14896	165	E/D	0,707	0,020949	0,124	18	1,9
MYCBP2	rs1315072581	ms	O75592	56	P/H	-0,607	0,000951	0,000	47	0,4
MYH13	17:10320355:T/C	ms	Q9UKX3	1085	K/E	0,779	0,024411	0,176	20	N/A
MYH15	rs199682590	ms	Q9Y2K3	1910	N/S	0,501	0,020825	0,970	48	9,1
MYH2	rs201040489	ms	Q9UKX2	445	R/H	-0,406	0,019307	0,985	48	18
MYH2	rs1567727764	stop	Q9UKX2	1551	E/*	0,560	0,018655	1,000	30	0,7

MYH3	rs143973840	ms	P11055	56	Q/E	-0,369	0,022519	0,000	**54**	**55**
MYH9	rs727503281	ms	P35579	1936	R/W	0,534	0,015971	**0,831**	**24**	4,2
MYO15A	rs145292219	ms	Q9UKN7	462	G/D	0,384	0,006460	**0,934**	104	310
MYO15B	rs769135244	ms	Q96JP2	673	R/C	-0,450	0,011251	0,007	**62**	0
MYO18A	rs748958172	ms	Q92614	785	M/V	-0,393	0,049062	0,000	**50**	0,1
MYO1F	rs557720157	ms	O00160	658	R/W	**-1,000**	0,009984	**0,846**	**12**	0,4
MYO1G	rs751701889	ms	B0I1T2	566	I/M	-0,385	0,033327	**0,933**	**44**	0
MYO1H	rs1566045601	stop	A0A140TA25	907	K/*	-0,583	0,030419	**1,000**	**31**	0,1
MYO5B	rs750051198	ms	Q9ULV0	125	Y/C	-0,598	0,050211	0,124	**17**	0,4
MYO7B	rs143550775	ms	A0A8C8KL71	1918	R/Q	0,556	0,030326	**0,984**	**29**	137
MYO7B	rs547914012	ms	A0A8C8KL71	394	R/Q	-0,668	0,010504	0,027	**39**	0,8
MYO9B	rs1568284597	ms	Q13459	682	V/M	0,462	0,022236	**0,485**	**23**	0,7
MYOM1	rs866006864	ms	P52179	229	A/V	**-1,019**	0,007773	0,000	**12**	6,8
MYRFL	rs765678851	ms	Q96LU7	716	R/Q	-0,621	0,020464	0,000	**37**	2,6
N4BP2	rs769146729	ms	Q86UW6	739	L/F	-0,714	0,023186	0,000	**16**	0,2
NAA25	rs746012353	ms	Q14CX7	74	L/R	-0,725	0,009316	**0,835**	**16**	6,0
NAA35	rs1564301637	ms	Q5VZE5	219	R/G	**-1,708**	0,001180	0,150	**10**	0,1
NABP1	rs764450900	ms	Q96AH0	198	P/L	-0,442	0,035796	**0,999**	**27**	0,8
NACAD	rs552765962	ms	O15069	1241	P/T	-0,416	0,021649	0,010	**46**	105
NACC2	rs759435250	ms	Q96BF6	541	E/K	0,888	0,000350	**0,646**	**18**	7,6
NADSYN1	rs143959747	ms	Q6IA69	60	S/L	-0,431	0,001849	**0,653**	**93**	4,5
NAGPA	rs1567142778	ms	Q9UK23	173	R/C	**-1,106**	0,000437	**0,977**	**19**	0,8
NAIF1	rs138010554	ms	Q69YI7	297	R/C	-0,986	0,001908	**0,874**	**17**	**60**
NAPG	rs376629414	ms	Q99747	293	D/G	-0,602	0,045270	0,003	**15**	9,1
NAV2	rs776348337	ms	Q8IVL1	1647	R/C	0,593	0,039251	0,069	**23**	4,5
NBEAL1	rs756929853	ms	A0A804HKS6	2124	Y/C	0,536	0,028777	**1,000**	**39**	0,1
NBPF14	rs1449518994	ms	A0A087WVU4	2974	T/R	-0,440	0,039055	0,000	**39**	2,1
NBPF8	1:120449308:G/C	ms	A0A8V8TN03	298	Q/H	-0,674	0,041223	**0,921**	**20**	N/A
NBPF9	rs1349107435	ms	P0DPF3	714	W/C	0,922	0,003405	0,000	**30**	**12**
NBPF9	rs1418431567	ms	P0DPF3	520	R/W	-0,670	0,019821	0,024	**31**	1,5
NCAPD3	rs199849895	ms	P42695	1404	E/K	-0,921	0,005266	0,000	**18**	**16**
NCF4	rs150976323	ms	Q15080	151	R/C	0,796	0,017743	**0,942**	**14**	1,5
NCKAP5	rs370409499	ms	O14513	795	Y/C	0,592	0,033956	0,003	**17**	4,9
NCMAP	rs113438005	ms	Q5T1S8	73	A/T	0,607	0,043935	0,000	**18**	6,8
NCOA6	rs113308271	ms	Q14686	163	A/V	0,441	0,041936	0,102	**22**	3,8
NCOR2	rs1396687230	ms	Q9Y618	1523	A/V	-0,537	0,007622	0,201	**31**	2,3
NCOR2	rs745312035	ms	Q9Y618	2483	A/T	**-1,147**	0,000139	0,036	**24**	**12**
NCOR2	rs761145072	ms	Q9Y618	2423	R/G	**1,079**	0,003446	**0,999**	**24**	0,7
NDRG1	rs145871479	ms	Q92597	11	A/T	0,495	0,038489	0,011	**35**	175
NDST1	rs138889348	ms	P52848	707	A/V	-0,392	0,019422	0,043	**43**	2,0
NDUFA8	rs776160822	ms	P51970	35	Q/R	-0,836	0,011373	**0,903**	**17**	0,4
NEB	rs750990726	ms	P20929-2	3295	R/W	-0,628	0,037046	**0,988**	**21**	3,4
NEDD4L	rs746580879	ms	Q96PU5-7	3	R/C	0,822	0,002146	0,203	**28**	7,6
NEIL1	rs759056574	ms	Q96FI4	373	R/Q	-0,655	0,037206	0,000	**12**	6,8
NEK3	rs34076988	ms	P51956	461	D/N	0,882	0,003367	0,282	**20**	**23**
NELFB	rs1564441238	ms	Q8WX92-2	139	K/N	0,391	0,049650	0,142	**25**	0,4
NELL2	rs375958961	ms	Q99435	766	R/C	-0,822	0,037169	**0,741**	**16**	**12**
NEMP1	rs1006583185	ms	O14524	397	T/M	0,649	0,042793	0,336	**23**	1,8
NEMP1	rs753618647	ms	O14524	379	R/Q	0,690	0,036435	**1,000**	**15**	2,3
NF2	rs900545157	ms	P35240	497	S/T	0,462	0,033501	0,021	**30**	2,6
NFIC	rs35952068	ms	P08651	265	T/S	0,854	0,002184	0,144	**21**	**63**
NFILZ	rs755954670	ms	A0A5F9ZHS7	153	R/Q	**1,066**	0,012246	0,014	**18**	0,7
NGEF	rs764647434	ms	Q8N5V2	591	R/C	0,689	0,031945	**0,761**	**16**	0,3
NHERF1	rs141613848	ms	O14745	301	D/V	0,568	0,020556	**0,895**	**28**	108
NHERF2	rs376297831	ms	Q15599	274	R/L	-0,479	0,021758	0,417	**44**	1,1
NHERF4	rs759297320	ms	Q86UT5	25	L/I	0,632	0,016991	0,374	**25**	1,1
NID1	rs201024490	ms	P14543	274	V/M	0,849	0,018617	**0,910**	**13**	4,2
NID1	rs34606818	ms	P14543	334	P/L	-0,950	0,004262	0,033	**31**	4,5
NID2	rs149533842	ms	Q14112	462	T/M	0,369	0,012335	0,000	**78**	**62**
NIFK	rs750327736	ms	Q9BYG3	17	Q/R	-0,358	0,034571	0,012	**52**	0,2
NIPAL1	rs1560326020	ms	Q6NVV3	287	V/I	-0,751	0,025387	0,416	**12**	0
NKAPD1	rs761966583	ms	Q6ZUT1-2	129	Q/E	0,970	0,037813	**0,935**	**10**	0
NKD1	rs140589227	ms	Q969G9	67	V/M	0,764	0,000447	0,003	**38**	5,3
NLRC3	rs138457262	ms	Q7RTR2	601	R/H	0,707	0,031914	**0,791**	**16**	568
NLRP11	rs1199059111	ms	P59045	113	F/S	-0,949	0,013463	0,025	**11**	1,3
NLRP12	rs200996095	ms	P59046	341	T/I	0,445	0,017156	**1,000**	**41**	**15**
NME6	3:48295131:T/C	ms	O75414	113	D/G	0,893	0,021765	0,163	**10**	N/A
NME8	rs754300578	ms	Q8N427	204	I/K	0,648	0,039185	0,003	**16**	0,4
NME9	rs2052125802	ms	Q86XW9	248	V/A	0,633	0,018887	0,040	**12**	0
NMI	rs185889672	ms	Q13287	264	G/D	-0,407	0,035144	0,000	**32**	2,3
NOS2	rs1567632363	ms	P35228	1002	H/Q	-0,603	0,021928	0,120	**20**	0,1
NOS2	rs745804011	ms	P35228	1002	H/R	-0,603	0,021928	0,003	**20**	0,8
NOTCH1	rs536167222	ms	P46531	2455	T/A	-0,399	0,001527	0,265	104	3,4
NOVA1	14:26448097:T/G	ms	P51513-4	462	E/D	**-1,074**	0,011270	0,149	**11**	N/A
NOVA1	rs777508869	ms	P51513-4	384	A/G	0,501	0,025642	0,095	**18**	0
NPC2	rs202134174	ms	G3V3E8	157	W/R	-0,628	0,014741	0,000	**25**	4,2
NPEPPS	rs201842079	ms	P55786	912	R/Q	0,712	0,015034	0,020	**18**	1,5
NPEPPS	rs571371035	ms	P55786	297	N/S	-0,560	0,015577	0,040	**32**	**16**
NPHP3	rs1559999380	ms	Q7Z494	1231	Q/H	0,509	0,030262	**0,988**	**26**	0,4
NPHP4	rs372565083	ms	O75161	482	P/L	0,916	0,008471	0,003	**14**	**14**
NPIPA1	rs539477828	ms	Q9UND3	303	D/E	0,647	0,030701	**0,930**	**26**	69
NPIPA2	16:14750871:C/T	ms	A0A0B4J2F6	20	A/V	0,628	0,018339	0,125	**25**	N/A
NPIPA3	16:14711747:C/T	ms	F8WFD2	20	A/V	0,663	0,021293	0,000	**26**	N/A
NPIPB15	rs920554826	ms	A6NHN6	194	R/Q	0,822	0,048740	**0,992**	**16**	0

NPIPB2	rs530343762	ms	A6NJ64	280	T/I	0,774	0,010180	0,998	26	0,8
NPIPB5	rs1421452584	ms	A8MRT5	646	M/V	0,559	0,043620	0,000	24	0
NQO2	rs766428660	ms	P16083	136	G/S	0,610	0,000903	0,998	59	5,4
NRG1	rs1563846753	ms	Q02297-10	28	S/C	0,581	0,030867	0,996	23	0
NRIP1	rs758399379	ms	P48552	624	T/M	0,449	0,025039	0,793	39	0,4
NSUN6	rs775368041	ms	Q8TEA1	181	R/C	-0,406	0,022017	1,000	46	1,5
NT5C	rs923470976	ms	Q8TCD5	86	E/K	-0,879	0,033730	0,360	11	1,5
NTF4	rs750511722	ms	P34130	110	A/T	-0,972	0,022342	0,995	15	0,4
NTN3	rs775996713	ms	O00634	321	R/H	-0,417	0,008602	0,995	42	2,6
NTN5	rs770743005	ms	Q8WTR8	461	R/Q	0,352	0,003457	0,065	120	1,5
NTSR2	rs1227438548	ms	O95665	120	Y/H	-0,398	0,028385	0,706	50	0,8
NUDT22	rs771324654	ms	Q9BRQ3	126	A/V	0,629	0,028987	0,003	10	0
**NUP107**	rs139991199	ms	P57740	54	R/Q	-0,492	0,028343	0,009	29	57
**NUP107**	rs200326452	ms	P57740	885	R/H	-0,355	0,046728	0,015	43	9,4
NUP205	rs78254699	ms	Q92621	1019	N/S	-0,408	0,004439	0,198	56	79
NUP210	rs138332591	ms	Q8TEM1	1178	R/Q	0,459	0,024832	0,953	49	8,7
NUP214	rs28594669	ms	P35658	1592	G/A	0,432	0,004022	0,278	65	970
NUP35	rs770715723	ms	Q8NFH5	236	I/L	-0,433	0,019623	0,298	32	1,3
NUP62	rs772416904	ms	P37198	74	F/L	0,577	0,014900	0,903	42	2,6
**NUP88**	rs772267425	fs	Q99567	212	I/X	-0,396	0,013610	1,000	70	0,4
**NUP88**	17:5405165:A/T	stop	Q99567	312	C/*	0,933	0,037030	1,000	15	N/A
NUTM2A	rs770632205	ms	Q8IVF1	784	S/L	0,370	0,002694	0,042	98	1,1
NUTM2B	rs1181064857	ms	A6NNL0	230	V/I	-0,370	0,027757	0,006	64	9,3
NUTM2E	rs1428647934	ms	B1AL46	230	V/I	-0,447	0,009085	0,010	61	0
NXNL2	rs751445572	stop	Q5VZ03	142	Q/*	1,212	0,004748	1,000	14	0,4
OAS3	rs377672802	ms	Q9Y6K5	443	R/C	-0,974	0,013515	0,001	12	5,3
OAT	rs778350564	ms	P04181	150	C/S	-0,550	0,048573	0,985	27	0,4
**OBSCN**	rs201385101	ms	A0A7P0Z489	5935	G/D	0,736	0,018230	0,962	14	20
**OBSCN**	rs375770928	ms	A0A7P0Z489	570	A/V	-0,436	0,048912	0,161	33	0,4
**OBSCN**	rs765706284	stop	Q5VST9-3	6524	R/*	0,869	0,027590	1,000	12	3,0
OCA2	rs372899234	ms	Q04671	421	R/W	-0,510	0,048989	0,978	18	5,7
OCSTAMP	rs1568897913	stop	Q9BR26	243	W/*	1,173	0,024410	1,000	10	0
OLFML2A	rs200109271	ms	Q68BL7	103	E/K	-0,444	0,028625	0,706	40	17
ONECUT2	rs781095849	ms	O95948	22	P/L	0,526	0,044109	0,007	17	1,1
OPN1MW	X:154191728:G/A	ms	P04001	207	V/M	-0,479	0,025769	0,982	57	N/A
OPN3	rs138406816	ms	Q9H1Y3	317	R/Q	-0,523	0,039876	0,992	25	65
OPRM1	rs200207721	ms	P35372	67	M/V	-0,361	0,00001722	0,000	260	0,4
OR10A4	rs1564922932	ms	Q9H209	153	G/E	0,749	0,017180	0,995	15	0
OR10A5	rs780579227	ms	Q9H207	220	T/S	0,386	0,034426	0,037	35	0
OR10C1	rs767356219	ms	Q96KK4	282	P/S	-0,383	0,011076	0,237	62	0
OR10G9	rs768689141	ms	Q8NGN4	15	L/F	-0,402	0,026754	0,005	52	3,0
OR10H4	rs1568263662	ms	Q8NGA5	129	H/Y	-0,838	0,020365	0,014	19	0
OR11H1	rs201166644	ms	A0A126GWF9	52	V/F	1,324	0,027400	0,195	11	133
OR11H4	rs142720326	ms	A0A286YFI7	9	V/M	-0,503	0,016773	0,795	36	114
OR1M1	rs771073707	ms	Q8NGA1	97	C/G	0,703	0,025232	0,986	11	0,8
OR2A5	rs201829543	ms	Q96R48	223	A/G	-1,014	0,010106	0,001	15	150
OR2D3	rs768917280	ms	Q8NGH3	152	M/T	-0,549	0,041307	0,431	25	0
OR2M3	1:248203600:T/C	ms	Q8NG83	178	F/S	0,696	0,037344	0,932	20	N/A
OR2M5	rs142232947	ms	A3KFT3	80	K/T	-1,026	0,026984	0,885	10	48
OR2T34	rs755824111	ms	Q8NGX1	197	V/I	-0,428	0,031577	0,003	45	0,9
OR2T35	rs768155855	ms	Q8NGX2	14	V/I	0,389	0,021787	0,000	54	8,0
OR2V1	rs768276571	ms	Q8NHB1	255	A/E	-0,815	0,009587	0,982	17	5,3
OR3A1	rs201927505	ms	P47881	131	R/Q	-0,549	0,011163	0,003	39	4,4
OR4K13	rs763176758	ms	Q8NH42	139	R/W	0,427	0,048124	0,115	30	2,5
OR51C1P	rs141670434	ms	A0A3B3IT45	122	R/H	-1,053	0,039579	0,125	12	105
OR51E1	rs149084031	ms	Q8TCB6	73	D/G	0,521	0,022172	0,661	23	11
OR51F1	rs781353738	ms	A6NGY5	174	P/R	0,794	0,044223	0,005	20	0,4
OR52H1	rs761950199	ms	Q8NGJ2	263	A/T	1,162	0,000300	0,179	13	1,1
OR52I1	rs768334451	ms	Q8NGK6	91	S/L	0,358	0,045049	0,099	41	6,8
OR52K1	rs200081615	ms	Q8NGK4	53	A/S	0,698	0,025046	0,001	15	2,0
OR56A4	rs1474585239	ms	Q8NGH8	216	L/F	0,718	0,022558	0,568	21	1,5
OR5AN1	rs777894055	ms	Q8NGI8	39	S/Y	0,423	0,008503	0,006	74	0,2
**OR5D14**	rs144838984	ms	Q8NGL3	299	V/M	-0,612	0,032912	0,964	25	94
**OR5D14**	rs201786123	ms	Q8NGL3	291	I/T	0,642	0,007345	0,995	21	8,3
OR5K1	rs759801175	ms	Q8NHB7	194	I/T	0,360	0,034809	0,801	47	0,4
OR5L2	rs143953099	ms	Q8NGL0	254	T/I	1,505	0,012528	0,984	13	49
OR5M3	rs143720540	stop	Q8NGP4	162	Y/*	-0,566	0,031033	1,000	23	1,9
OR5V1	rs151167090	ms	Q9UGF6	121	D/G	0,507	0,006861	0,997	45	10
OR6B1	rs769317140	ms	O95007	218	Y/H	0,577	0,037836	1,000	13	0,1
OR6J1	rs536617937	ms	Q8NGC5	144	V/I	0,573	0,023570	0,018	20	3,8
OR6K3	rs138237790	ms	A0A0C4DFU5	37	F/C	0,466	0,028677	0,999	25	4,5
OR6K3	rs754875892	ms	A0A0C4DFU5	52	D/N	-0,460	0,006309	0,052	65	8,1
OR8B12	rs146463957	ms	Q8NGG6	35	T/M	0,540	0,033026	0,000	30	20
OR8H2	rs146032852	ms	Q8N162	30	M/V	-0,708	0,046489	0,000	12	0,8
OR9Q1	rs148066479	ms	Q8NGQ5	287	L/I	0,642	0,007341	0,031	21	31
ORM2	rs779305528	ms	P19652	104	G/R	0,790	0,012866	0,996	13	0,8
**OSBPL10**	rs1559483397	ms	Q9BXB5	228	A/V	1,191	0,010324	0,834	10	0
OSCP1	rs773468810	stop	Q8WVF1-3	160	Q/*	0,587	0,051420	1,000	11	0,4
OSGIN2	rs115758475	ms	Q9Y236-2	449	K/N	0,366	0,037508	0,443	49	107
OTOG	rs755375116	ms	H9KVB3	516	R/C	0,467	0,015782	0,956	32	8,1
OTOGL	rs766038825	stop	Q3ZCN5	1635	Q/*	-0,783	0,017680	1,000	13	0,2
OTOP1	rs150117288	ms	Q7RTM1	206	S/L	0,621	0,003479	0,036	39	3,4
OTOP3	rs773800453	ms	Q7RTS5-2	68	A/T	-0,558	0,012593	0,063	26	0,7
OTUD1	rs1294078058	ms	Q5VV17	109	T/M	-0,544	0,047053	0,928	14	1,8

OXA1L	rs756119586	ms	Q15070	342	L/V	-0,591	0,049629	0,245	**15**	6,1
OXSR1	rs750858309	ms	O95747	126	I/V	-0,392	0,033042	0,020	**38**	0
P4HA2	rs371295093	ms	O15460-2	380	R/W	0,514	0,028003	**1,000**	**40**	0,8
PABIR1	rs777582132	ms	Q96E09	49	P/L	0,395	0,026398	0,000	**52**	0,4
PACS1	rs762496747	ms	Q6VY07	716	G/R	0,857	0,012978	**1,000**	**10**	0,8
PAN2	rs147436117	ms	Q504Q3-2	208	F/C	0,557	0,045798	**0,897**	**18**	**41**
PAN2	rs185741202	ms	Q504Q3-2	492	R/H	0,596	0,015335	**0,538**	**33**	0
PAOX	rs1167907763	ms	Q6QHF9-2	391	M/V	0,887	0,037675	**0,621**	**12**	0,4
PAPOLG	rs374304234	ms	Q9BWT3	23	S/T	-0,572	0,048085	**0,780**	**32**	3,0
PARD6G	rs1007114977	ms	Q9BYG4	278	G/V	0,591	0,016185	0,044	**33**	0,4
PARP10	rs769913263	ms	Q53GL7	216	T/A	-0,640	0,002238	**0,532**	**53**	0,3
PARP4	rs4986818	ms	Q9UKK3	792	P/L	-0,762	0,011319	**0,996**	**19**	**76**
PATE1	rs754444895	ms	Q8WXA2	76	M/T	-0,490	0,014732	0,001	**43**	0,1
PCCB	rs770769499	ms	P05166	34	S/F	-0,582	0,007726	0,176	**27**	0,2
PCDHA11	rs745813666	ms	Q9Y5I1	353	S/F	0,385	0,005343	**0,978**	**77**	0,7
PCDHA12	rs73263833	ms	Q9UN75	662	T/M	-0,487	0,003067	**0,774**	**44**	590
PCDHB1	rs141650927	ms	Q9Y5F3	768	F/V	0,395	0,029803	**0,981**	**54**	**11**
PCDHB14	rs199617011	ms	Q9Y5E9	509	D/G	-0,355	0,024234	0,078	**68**	**11**
PCDHGC4	rs1562119204	ms	Q9Y5F7	747	N/S	**1,417**	0,002367	0,060	**11**	0,3
PCGF1	rs1558594802	ms	Q9BSM1	215	P/T	-0,798	0,011231	0,219	**20**	0
PCGF6	rs571651624	ms	Q9BYE7	17	T/S	-0,549	0,027269	0,000	**30**	**22**
PCK1	rs200480357	ms	P35558	436	R/C	-0,396	0,010804	**1,000**	**60**	6,4
PCLO	rs1562983254	ms	Q9Y6V0-5	879	P/H	-0,935	0,027890	0,000	**14**	0,1
PCLO	rs778633853	ms	Q9Y6V0-5	570	P/R	0,925	0,046309	0,000	**11**	0,5
PCM1	rs749269864	ms	Q15154	1062	Y/C	-0,907	0,039011	**1,000**	**11**	2,6
PCMTD2	rs778503642	ms	Q9NV79	285	R/C	-0,356	0,046388	**0,544**	**42**	3,0
PCNT	rs373849607	ms	O95613	2142	I/T	-0,452	0,001901	0,158	**80**	6,8
PCNT	rs757715830	ms	O95613	899	R/C	0,390	0,016158	0,000	**52**	0,8
PCSK5	rs1824271458	ms	A0A669KA35	846	N/S	-0,801	0,008268	0,007	**11**	0,4
PCSK6	rs372998470	ms	H7BXT3	661	T/I	-0,601	0,044507	0,000	**28**	138
PDCD4	rs138747298	ms	Q53EL6	129	Q/E	-0,863	0,042689	0,000	**11**	1,1
PDE10A	rs1430464939	ms	A0A087WUD0	39	R/Q	-0,699	0,034116	0,000	**17**	0,4
PDE4DIP	rs1375289217	ms	Q5VU43-4	2342	Q/R	0,475	0,038551	0,177	**20**	1828
PDLIM5	rs768674311	ms	Q96HC4	150	F/C	0,846	0,015182	**0,993**	**13**	0,4
PDS5B	rs771715623	ms	Q9NTI5	775	L/V	0,630	0,044944	0,113	**13**	0,8
PDX1	13:27920241:T/G	ms	P52945	35	C/G	-0,606	0,006410	**0,994**	**20**	N/A
PECAM1	rs1568032195	ms	P16284	247	M/V	-0,382	0,035283	0,000	**47**	0
PEG10	rs201604172	ms	A0A087WX23	577	V/L	-0,749	0,007122	0,034	**18**	**41**
PELO	rs754185927	ms	Q9BRX2	301	K/E	0,604	0,036723	0,015	**15**	0
PELP1	rs746811925	ms	Q8IZL8	242	R/Q	0,468	0,026459	0,000	**32**	0,8
PEPD	rs1380412288	fs	A0A8V8TLP4	419-420	-/X	0,781	0,033998	**1,000**	**14**	1,1
PER2	rs141480763	ms	O15055	240	H/R	0,558	0,006834	**0,890**	**41**	3,4
PERCC1	rs545814553	ms	A0A1W2PR82	127	R/Q	0,362	0,049707	0,030	**44**	**81**
PEX1	rs748239213	ms	O43933	581	R/W	0,353	0,036555	**0,952**	**49**	0,8
PFKP	rs778429333	ms	Q01813	410	N/S	0,568	0,049244	0,006	**30**	3,8
PGM3	rs781153408	ms	O95394	474	P/A	0,403	0,016322	**1,000**	**58**	0,1
PGPEP1	rs367878913	ms	Q9NXJ5	145	G/S	0,566	0,000600	**1,000**	**58**	2,3
PHC2	rs147330538	ms	Q8IXK0-5	532	L/F	0,645	0,050832	**0,994**	**15**	**60**
PHF12	17:28913910:T/C	ms	Q96QT6	421	E/G	-0,814	0,035586	**0,462**	**17**	N/A
PHF2	rs146063352	ms	O75151	480	P/L	-0,395	0,000723	**0,981**	125	242
PHF20	20:35941003:A/G	ms	Q9BVI0	951	D/G	-0,786	0,004681	**0,990**	**22**	N/A
PHGDH	rs141408688	ms	O43175	528	A/D	0,769	0,051341	**0,778**	**11**	3,1
PHKG2	rs930832189	ms	P15735	322	A/V	-0,637	0,027631	0,017	**11**	0,4
PHLDB2	rs370795450	ms	Q86SQ0	1054	E/K	-0,427	0,049802	0,163	**37**	9,4
PHLPP1	rs772545306	ms	O60346	497	L/F	0,467	0,026163	**0,996**	**24**	0,4
PHLPP2	rs772778829	ms	Q6ZVD8	841	E/G	-0,462	0,033747	**0,998**	**37**	0,7
PHRF1	rs202005033	ms	Q9P1Y6	744	H/R	**1,327**	0,003328	0,234	**19**	**48**
PHYHIPL	10:59245166:T/G	ms	Q96FC7	236	F/V	-0,925	0,028552	**0,965**	**14**	N/A
PIGF	rs139098189	ms	Q07326	124	T/A	-0,813	0,039281	**0,462**	**15**	162
PIGQ	rs750660902	ms	Q9BRB3	589	R/C	-0,620	0,038659	0,118	**19**	1,7
PIGQ	rs767700650	ms	Q9BRB3-2	487	L/P	-0,688	0,012124	**0,902**	**36**	0,1
PIK3C2A	rs61755370	ms	O00443	133	F/L	0,936	0,026456	0,000	**12**	207
PIK3C2G	rs201664666	ms	O75747	202	E/V	0,367	0,044890	0,286	**42**	**23**
PIK3C3	rs777277652	ms	Q8NEB9	394	M/V	0,556	0,008726	0,106	**34**	0
PIK3CA	rs200404201	ms	P42336	694	A/S	0,537	0,014547	0,053	**47**	**11**
PIK3CG	rs542155244	ms	P48736	1095	K/R	-0,428	0,024623	**0,948**	**36**	1,5
PIK3R4	rs779308946	ms	Q99570	86	P/L	-0,593	0,019138	**0,996**	**27**	0,1
PILRA	rs1563114512	ms	Q9UKJ1	101	W/C	**-1,492**	0,013181	**0,998**	**15**	0
PIP5K1C	rs772615202	ms	O60331-3	681	P/S	-0,442	0,041192	0,027	**34**	0
PIWIL4	rs752333176	ms	Q7Z3Z4	272	T/M	0,359	0,048195	**0,976**	**41**	1,1
PJA2	rs187439518	ms	O43164	272	N/D	0,625	0,036766	0,001	**37**	0,3
PKD1	rs375440448	ms	P98161	1961	A/V	0,458	0,044017	0,140	**30**	**16**
PKD1	rs753792665	ms	P98161	3670	M/T	-0,942	0,009349	0,104	**16**	1,5
PKD1	rs763775092	ms	P98161	2479	F/L	0,810	0,004974	**0,691**	**24**	0,4
PKD1	rs770514810	ms	P98161	1336	N/K	0,627	0,037144	**0,992**	**12**	**13**
PKD1L1	rs767709684	ms	Q8TDX9	365	D/A	0,482	0,043967	0,014	**31**	4,9
PKD2	rs1426011277	ms	Q13563	67	P/S	0,373	0,028957	**0,862**	**47**	0,8
PKHD1	rs201812542	stop	P08F94	4048	Q/*	-0,418	0,012452	**1,000**	**63**	**15**
PKHD1L1	8:109508249:G/A	ms	Q86WI1	3794	V/I	0,619	0,048753	0,216	**13**	N/A
PKM	rs777998040	ms	P14618	162	K/E	-0,879	0,024313	**0,999**	**20**	0,4
PLA2G4E	rs756827449	ms	A0A8Q3WM91	319	R/W	-0,402	0,009013	**1,000**	**61**	0,8
PLCE1	rs752386035	ms	Q9P212	614	T/M	0,433	0,001883	**0,898**	111	3,4
PLCE1	rs776791387	ms	Q9P212	657	L/F	-0,491	0,051383	**0,832**	**26**	0,4
PLCG2	rs573799583	ms	P16885	641	H/N	0,822	0,012706	0,427	**15**	0,7

PLCH1	rs752280073	ms	A0A2U3TZV8	1521	V/M	-0,649	0,026774	0,031	**25**	3,4
PLD4	rs1566891650	ms	Q96BZ4	503	V/L	0,603	0,035020	0,003	**18**	0,4
PLEC	rs200887085	ms	Q15149-4	746	R/H	0,432	0,019276	0,000	**40**	149
PLEC	rs763436354	ms	Q15149-4	3530	C/Y	0,371	0,026375	0,000	**76**	1,2
PLEC	rs781892147	ms	Q15149-4	681	R/W	0,603	0,037025	0,000	**22**	0,8
PLEC	rs953750194	ms	Q15149-4	1835	L/F	0,418	0,035443	**0,998**	**42**	0,4
PLEKHA4	rs150889080	ms	Q9H4M7	75	R/H	-0,496	0,033019	**0,981**	**50**	**22**
PLEKHM1	rs762560450	ms	Q9Y4G2	1027	V/I	0,652	0,006516	**0,927**	**22**	5,3
PLEKHN1	rs779526083	ms	Q494U1	362	R/C	0,791	0,023007	0,003	**13**	6,4
PLG	rs149145958	ms	P00747	200	T/A	-0,663	0,005447	**0,959**	**33**	**66**
PLIN5	rs758198765	ms	Q00G26	463	F/L	0,399	0,045737	**0,938**	**38**	0,4
PLPP6	rs72695803	ms	Q8IY26	293	S/R	-0,682	0,035006	0,036	**12**	**83**
PLXNA1	rs370434411	ms	Q9UIW2	443	R/Q	**1,060**	0,002239	0,009	**18**	8,3
PLXNA1	rs576960383	ms	Q9UIW2	2	P/L	-0,596	0,039574	0,003	**24**	**58**
PLXNA2	rs368547025	ms	O75051	1474	I/T	-0,979	0,008718	**0,999**	**19**	3,8
PLXNA2	rs770760535	ms	O75051	1728	S/N	-0,628	0,010733	0,082	**24**	1,1
PLXNA3	rs369435958	ms	P51805	519	R/H	0,421	0,047746	0,049	**30**	0
PLXNA3	rs370403729	ms	P51805	287	V/M	-0,354	0,021806	0,202	**70**	**17**
PLXND1	rs1445315536	ms	Q9Y4D7	897	E/K	0,710	0,018356	0,376	**15**	0,4
PM20D1	rs148054590	ms	Q6GTS8	78	E/D	-0,496	0,002180	0,017	**57**	**46**
PMAIP1	rs772510569	ms	Q13794	40	Q/K	-0,797	0,021814	**0,737**	**15**	0,4
PMPCA	rs746125261	ms	Q5SXN9	487	R/K	-0,869	0,043216	0,001	**27**	3,4
PMPCB	rs761593488	ms	O75439	122	E/A	0,744	0,043541	**0,961**	**19**	0,4
PNKP	rs1353421318	ms	Q96T60	296	N/S	0,711	0,029379	0,117	**18**	0,4
PNLDC1	rs745361800	ms	Q8NA58-2	498	R/H	0,458	0,043923	**0,844**	**25**	1,5
PNMA1	rs762946571	ms	Q8ND90	55	F/L	0,366	0,026697	0,356	**51**	1,3
PNPLA6	rs143072391	ms	A0A384DVU0	1238	Q/E	-0,399	0,019805	0,076	**68**	**16**
PODN	rs754036511	ms	Q7Z5L7	80	V/A	-0,795	0,031269	**0,978**	**13**	2,6
POFUT2	rs184052047	ms	Q9Y2G5-3	130	G/D	-0,471	0,023682	**0,993**	**44**	0,8
POGK	rs894770585	ms	Q9P215	524	N/H	0,728	0,048584	**0,907**	**15**	1,8
POLA2	rs199754207	ms	Q14181	175	G/S	0,518	0,007575	**0,994**	**43**	**40**
POLD3	rs2031617851	ms	Q15054	49	R/Q	0,675	0,046564	**0,485**	**19**	0,4
POLN	rs142462434	ms	Q7Z5Q5	681	V/M	0,710	0,001735	**0,990**	**29**	**20**
POLN	rs148062138	ms	Q7Z5Q5	630	R/H	-0,762	0,010469	**1,000**	**12**	**43**
POLN	rs201300268	ms	Q7Z5Q5	630	R/S	-0,762	0,010469	**1,000**	**12**	5,7
POLR2H	rs776239606	ms	P52434	98	R/S	0,405	0,036203	**0,791**	**49**	0,4
POLR2J2	7:102666957:G/C	ms	A6NFM0	150	P/A	-0,472	0,007951	**0,550**	**48**	N/A
POLRMT	rs139383492	ms	O00411	870	D/N	-0,538	0,011386	0,350	**31**	**1027**
POM121	rs759615955	ms	Q96HA1-3	993	S/C	0,630	0,030783	0,000	**19**	0,4
POM121C	rs201031798	ms	A8CG34-2	766	T/I	0,417	0,010488	0,157	**55**	0,4
POM121C	rs202010067	ms	A8CG34-2	764	Q/H	0,389	0,040861	0,003	38	267
POM121L12	rs1156584443	ms	Q8N7R1	172	P/S	-0,653	0,006138	0,162	**19**	0,4
POMGNT1	rs200643988	ms	Q8WZA1	168	A/V	0,413	0,020751	0,120	**33**	0,8
POR	rs779082897	ms	P16435	565	G/S	-0,519	0,029963	**0,997**	**37**	6,4
POSTN	rs771635660	ms	Q15063	569	P/S	0,801	0,013628	0,318	**12**	0,1
POTEF	rs755588157	ms	A5A3E0	883	R/W	**1,136**	0,014870	0,431	**13**	0,4
POU5F1B	rs562626736	ms	Q06416	17	G/C	-0,979	0,011605	**0,959**	**21**	0,4
PPARGC1B	rs930128955	ms	Q86YN6	4	N/S	0,520	0,051483	0,000	**19**	0,4
PPIP5K2	rs559400858	ms	O43314	1083	R/H	0,601	0,004589	0,020	**36**	4,0
PPL	rs200709609	ms	O60437	275	S/G	0,773	0,024901	0,017	**23**	**18**
PPM1D	rs770814597	ms	O15297	321	P/S	-0,585	0,032458	0,221	**31**	1,9
PPM1E	rs200685038	ms	Q8WY54-2	750	W/L	-0,407	0,033836	0,057	**58**	0,8
PPP1R12B	rs770806770	ms	E1CKY7	18	Q/R	-0,931	0,018316	**0,617**	**20**	6,4
PPP1R21	rs1558437735	ms	Q6ZMI0	151	A/V	-0,544	0,027062	0,201	**22**	0,3
PPP1R3A	7:113879662:A/T	ms	Q16821	477	I/N	0,746	0,004033	0,035	**25**	N/A
PPP1R3C	rs778620061	fs	Q9UQK1	224	P/X	-0,461	0,028877	**1,000**	**34**	0,4
PPP1R9B	rs539885092	ms	Q96SB3	138	P/L	0,862	0,025998	**0,722**	**10**	0,1
PPP6R1	rs547677018	ms	Q9UPN7	763	R/C	-0,470	0,017089	0,000	**46**	**24**
PRAMEF15	rs1394626954	ms	P0DUQ1.12,P0DUQ2	442	N/K	-0,565	0,008879	0,000	**49**	0,4
PRAMEF17	rs201071276	ms	Q5VTA0	294	P/L	-0,610	0,035305	0,082	**11**	0,4
PRAMEF17	rs555191694	ms	Q5VTA0	306	A/T	-0,610	0,035304	0,000	**11**	**21**
PRB3	rs369321112	ms	Q04118	274	G/R	0,389	0,010377	0,000	**65**	**48**
PRDM1	rs753053806	ms	O75626	558	G/S	-0,549	0,027141	**0,980**	**19**	0,8
PRDM2	rs776188300	ms	Q13029	1097	M/V	0,451	0,049946	0,003	**41**	0,4
PRDM5	rs757199736	ms	Q9NQX1	135	Q/R	-0,908	0,032976	0,070	**26**	0
PRKAR2A	rs748291376	ms	P13861	131	Q/R	-0,631	0,019133	**0,768**	**13**	0,2
PRKCA	rs1567761731	ms	P17252	48	T/I	0,798	0,014934	**0,654**	**18**	0,1
PRKCA	rs748202197	ms	P17252	381	V/M	**1,074**	0,001778	**0,920**	**12**	1,5
PRKDC	rs55924155	ms	P78527	3677	P/S	-0,785	0,023872	0,174	**13**	**55**
PRKDC	rs56080897	ms	P78527	1337	V/I	0,726	0,027659	0,003	**11**	92
PRMT9	rs139425779	ms	Q6P2P2	594	L/V	0,664	0,002135	0,214	**25**	**24**
PRORP	rs45626032	ms	O15091	229	A/V	-0,769	0,048835	**0,668**	**13**	130
PROSER2	rs200622168	ms	Q86WR7	362	A/T	-0,460	0,030193	0,000	**35**	**48**
PRPF3	rs1560126950	ms	O43395	675	S/N	-0,723	0,003864	0,090	**21**	0
PRPF31	rs367896277	ms	Q8WWY3	408	R/W	0,659	0,043020	**1,000**	**16**	0,7
PRPF40B	rs745405961	ms	F8VU11	333	Y/C	0,368	0,045906	**0,997**	**38**	0,2
PRR14	rs761866966	ms	Q9BWN1	43	P/S	0,378	0,048100	0,046	**43**	0
PRR14	rs778010296	ms	Q9BWN1	99	G/R	-0,695	0,021036	0,000	**17**	3,8
PRR15	rs774986809	ms	Q8IV56	96	R/L	-0,476	0,041195	**0,773**	**36**	0,8
PRR29	rs1567838278	ms	P0C7W0	29	L/F	-0,672	0,014169	0,019	**20**	0
PRR29	rs774072605	ms	P0C7W0	109	H/Y	0,815	0,035005	**0,999**	**13**	0,4
PRR30	rs377666702	ms	Q53SZ7	257	R/P	-0,393	0,050246	0,153	**49**	0
PRRC2A	rs201074309	ms	P48634	1397	R/W	0,416	0,051172	0,000	**32**	**65**
PRRC2B	rs908183601	ms	Q5JSZ5	1106	S/R	**1,112**	0,018219	0,200	**14**	1,3

PRRT2	rs1567380661	ms	Q7Z6L0	331	S/F	0,469	0,049994	0,998	32	0
PRRT3	rs199883920	ms	Q5FWE3	150	P/S	0,831	0,015907	0,987	14	46
PRSS33	rs186640650	ms	Q8NF86	278	V/I	0,643	0,031603	0,005	17	83
PRSS54	rs753895790	ms	Q6PEW0	326	G/E	0,758	0,021477	0,000	19	0
PSAPL1	rs201866250	ms	Q6NUJ1	364	R/H	-0,361	0,010182	0,803	74	162
PSD4	rs1558893290	ms	Q8NDX1	637	R/G	1,206	0,014207	0,018	12	0,4
PSMC5	rs1231484066	ms	P62195	43	R/W	-0,591	0,025967	0,692	15	0,4
PSMD1	rs201791497	ms	Q99460	89	N/S	-0,383	0,050862	0,007	33	8,7
PSMD12	rs780349428	ms	O00232	234	D/N	0,774	0,004686	0,024	32	3,4
PSME3IP1	rs746174590	ms	Q9GZU8	146	K/T	-0,809	0,000273	0,964	30	0,4
PSRC1	rs116496512	ms	Q6PGN9-2	86	A/V	0,963	0,014683	0,986	13	177
PTCD3	rs144366369	ms	Q96EY7	682	D/N	0,845	0,032231	0,000	18	60
PTPN13	rs758235474	ms	Q12923	156	H/L	-0,377	0,042326	0,898	58	0,4
PTPN13	rs772350639	ms	Q12923	1893	L/M	1,182	0,020599	0,961	22	1,5
PTPN14	rs148337405	ms	Q15678	1068	T/M	0,375	0,009301	0,999	95	23
PTPN3	rs200101992	ms	P26045	436	H/Y	0,508	0,010660	0,000	49	7,6
PTPN5	rs753563589	ms	P54829	45	E/K	0,690	0,027725	0,000	19	1,1
PTPRC	rs140403368	ms	P08575-3	433	D/N	-0,407	0,037750	0,000	48	90
PTPRD	rs760402372	ms	P23468	1869	V/G	-0,498	0,036245	0,997	34	0
PTPRF	rs771277235	ms	P10586	1336	D/N	0,523	0,045164	0,143	22	0,4
PTPRF	rs887326029	ms	P10586	493	A/V	0,932	0,018146	0,611	13	5,7
PTPRJ	rs183923997	ms	Q12913	959	R/H	0,520	0,009112	0,335	61	27
PTPRJ	rs779058301	ms	Q12913	1336	I/N	0,547	0,001375	0,920	64	1,5
PTPRO	12:15569445:T/C	ms	Q16827	926	Y/H	0,780	0,034337	0,921	11	N/A
PTPRZ1	rs1188819495	ms	P23471	1466	L/F	-0,676	0,031690	0,000	14	0,4
PTPRZ1	rs139790820	ms	P23471	187	T/A	0,582	0,044226	0,000	28	60
PUS1	rs199863621	ms	Q9Y606	122	R/Q	0,397	0,026247	0,007	36	6,0
PUS7	rs752082590	ms	Q96PZ0	253	S/T	0,533	0,031816	0,333	24	0
PYCR1	rs757343922	ms	P32322	4	G/C	0,419	0,003520	1,000	64	0,4
PYGM	rs115690781	ms	P11217	259	N/S	-0,393	0,042959	0,935	31	6,4
QRICH2	rs759856315	ms	A0A7P0T7G7	277	S/R	0,550	0,008128	0,000	45	0,8
QTRT1	rs139560362	ms	Q9BXR0	117	L/M	0,523	0,017103	0,909	36	160
R3HCC1L	rs145569032	ms	A0A384DVK4	539	I/M	-0,784	0,017497	0,264	17	19
RAB11FIP1	rs375424635	ms	Q6WKZ4-4	966	A/S	-0,450	0,024418	0,230	28	2,3
RAB11FIP3	rs1352095348	ms	O75154	643	R/W	0,758	0,015808	0,046	13	1,5
RAB11FIP5	rs114626386	ms	A0A1B0GTL5	933	E/K	-1,078	0,000999	0,000	13	745
RAB34	rs1567732776	ms	Q9BZG1	90	E/Q	0,685	0,018760	0,999	11	0,4
RAB37	rs772174577	ms	Q96AX2	133	Q/P	-0,621	0,016129	0,994	19	14
RAB3GAP2	rs377291864	ms	Q9H2M9	991	A/G	-0,525	0,042343	0,000	30	1,6
RAB3GAP2	rs747310001	ms	Q9H2M9	120	W/C	-0,672	0,020130	0,977	16	0,1
RAB3GAP2	rs754005017	ms	Q9H2M9	303	Q/R	0,350	0,035170	0,292	49	0,4
RAB3IL1	rs1281765508	stop	Q8TBN0	249	R/*	-0,461	0,048476	1,000	21	0
RABEP1	rs368832529	ms	Q15276	793	V/I	-0,364	0,037696	0,014	52	4,9
RABEP1	rs761934519	ms	Q15276	862	T/S	0,706	0,009912	0,958	22	2,2
RABGEF1	rs1562896004	ms	Q9UJ41-2	447	R/T	0,793	0,016162	0,019	10	0,2
RABGEF1	rs373315327	ms	Q9UJ41-2	96	R/C	0,409	0,042234	0,676	34	0,4
RABL6	rs761584878	ms	Q3YEC7	595	P/S	0,374	0,041172	0,647	62	0,9
RAD51C	rs199886026	ms	O43502	244	I/V	0,425	0,000102	0,127	152	3,8
RAD51C	rs756727559	ms	O43502	183	I/T	0,433	0,041228	0,600	38	1,9
RAD54L2	rs746877630	ms	Q9Y4B4	534	I/T	0,510	0,021923	0,734	24	0,3
RALGAPA1	rs376013955	ms	A0A7P0TAR5	405	R/T	-0,603	0,024713	0,428	33	2,6
RALGAPA2	rs778008706	ms	Q2PPJ7	1050	E/K	-0,428	0,014171	0,000	54	1,1
RALGDS	rs764488895	ms	Q12967	262	S/L	0,728	0,007455	0,316	24	2,6
RANBP6	rs1842548384	ms	O60518	202	A/P	0,832	0,003456	0,962	11	0,2
RAP1B	rs372360254	ms	P61224	111	M/V	-0,579	0,037921	0,922	16	2,7
RASA2	rs764501312	ms	Q15283-2	210	T/I	-0,484	0,021478	0,972	23	0
RASA4B	7:102501161:T/C	ms	C9J798	193	M/V	0,661	0,031486	0,010	29	N/A
RASA4B	rs1253896488	ms	C9J798	633	G/S	-0,780	0,047755	0,000	11	0
RBM12B	rs200294553	ms	Q8IXT5	685	R/Q	0,834	0,023712	0,030	25	16
RBM19	rs763546088	ms	Q9Y4C8	125	E/V	0,675	0,008847	0,160	23	0,2
RBM33	rs371318332	ms	Q96EV2	836	A/P	0,501	0,043719	0,536	24	2,3
RC3H1	rs377392987	ms	Q5TC82	557	I/V	0,445	0,012498	0,000	54	3,0
RCAN1	rs1568938287	ms	P53805	58	E/D	-0,880	0,010558	0,046	14	0
RCN3	rs764847109	ms	Q96D15	57	E/K	-0,948	0,018724	0,958	13	0,8
RD3	rs747677203	ms	Q7Z3Z2	44	Q/K	0,548	0,035771	0,550	26	0,8
RECQL5	rs756694533	ms	O94762	490	G/D	0,746	0,018287	0,766	10	2,7
RELCH	rs746069802	ms	A0A2R8Y566	700	H/Y	0,562	0,005905	0,073	52	0,4
RELCH	rs755098911	ms	A0A2R8Y566	325	A/T	0,727	0,015258	0,079	15	0,4
REST	rs1560451173	ms	Q13127	342	S/A	0,761	0,020945	0,993	10	0
REXO1	rs544520974	ms	Q8N1G1	566	P/L	0,488	0,020417	0,000	50	15
RFX5	rs149605765	ms	P48382	284	L/P	0,700	0,012148	0,000	26	19
RFXAP	rs1483760429	ms	O00287	19	P/S	-0,714	0,039846	0,810	12	3,4
RGPD1	rs1266171383	ms	A0A286YES2	977	Q/P	-0,489	0,042248	0,548	22	0
RGPD2	2:87784094:T/G	ms	P0DJD1	977	Q/P	-0,489	0,042290	0,801	23	N/A
RGPD5	2:109803997:C/T	ms	Q99666	91	P/S	-0,649	0,033501	0,880	20	N/A
RGPD6	2:110540064:G/A	ms	Q99666	865	P/S	0,550	0,047134	0,022	24	N/A
RGPD8	rs1369419755	ms	O14715	91	P/S	-0,431	0,014526	0,961	60	105
RGPD8	rs200798390	ms	O14715	1128	A/T	-0,431	0,014526	0,486	60	94
RGR	rs146536539	ms	P47804	77	L/F	-0,496	0,004957	0,036	64	44
RGS1	rs150745219	ms	Q08116	156	R/Q	0,400	0,002236	0,009	92	16
RGS16	rs753151197	ms	O15492	22	R/H	-0,833	0,006492	0,506	21	4,2
RGS3	rs137990160	ms	A0A8Q3WKG2	875	G/S	-0,514	0,008371	0,015	39	42
RHBDF1	rs368764349	ms	Q96CC6	234	R/C	-0,435	0,045636	0,023	41	7,9
RHOBTB2	rs146491810	ms	Q9BYZ6	245	V/A	0,486	0,011830	0,499	36	132

RHPN1	rs371033148	ms	Q8TCX5	347	K/N	-0,935	0,042249	**0,999**	**13**	**13**
RIC8B	rs762850738	ms	B7WPL0	479	K/R	0,688	0,047682	**0,848**	**13**	4,9
RILP	rs34982553	ms	Q96NA2	281	R/Q	**-1,114**	0,041984	**0,968**	**11**	897
RIMBP3C	rs1555881974	ms	A6NJZ7	667	G/R	-0,392	0,049856	0,022	**57**	0
RIOK1	rs547473770	ms	Q9BRS2	220	R/W	0,772	0,047832	**1,000**	**14**	0,8
RLBP1	rs760538477	ms	P12271	60	E/K	0,433	0,015995	0,039	**38**	0,4
RMDN2	rs142128542	ms	A0A0C4DFM4	226	D/N	**-1,132**	0,015245	0,020	**12**	**18**
RND1	rs148733686	ms	Q92730	145	A/V	-0,475	0,019541	0,001	**44**	**19**
RND2	rs748749886	ms	P52198	127	L/R	-0,712	0,011781	**0,962**	**18**	0
RNF115	rs774535104	ms	Q9Y4L5	276	D/E	0,640	0,027036	0,084	**29**	0,4
RNF123	rs2080162391	ms	Q5XPI4	846	L/P	-0,538	0,036043	0,382	**25**	5,1
RNF123	rs761494332	ms	Q5XPI4	38	Y/C	-0,429	0,006319	**0,985**	**62**	0,4
RNF145	rs545563752	ms	Q96MT1	536	I/T	**1,006**	0,045136	0,307	**10**	0,2
RNF169	rs759737139	ms	Q8NCN4	119	R/H	**-2,509**	0,000674	**0,837**	**12**	**75**
RNF17	rs776307815	ms	Q9BXT8-3	1182	V/I	-0,468	0,009183	0,007	**45**	1,1
RNF213	rs1568136656	ms	A0A0A0MTR7	3911	A/D	**-1,633**	0,000401	0,006	**11**	0
RNF4	rs776582971	ms	P78317	138	G/R	-0,675	0,003795	0,019	**18**	0,8
RNF44	rs201402074	ms	Q7L0R7	199	P/S	-0,798	0,042629	0,034	**15**	**23**
ROBO4	rs776645625	ms	Q8WZ75	435	V/I	-0,516	0,036068	0,033	**17**	0,4
ROGDI	rs1567600743	ms	Q9GZN7	139	G/D	0,801	0,042088	**0,712**	**13**	0
ROGDI	rs773589345	ms	Q9GZN7	187	D/H	**1,212**	0,023385	**0,984**	**12**	1,9
RP1	rs758050819	ms	P56715	15	T/M	-0,467	0,000847	**0,462**	104	0,4
RP1L1	rs200588941	ms	Q8IWN7	2204	E/A	0,861	0,008967	0,000	**12**	**25**
RPF1	rs145776320	ms	Q9H9Y2	4	A/V	0,403	0,032791	0,000	**38**	373
RPGRIP1L	rs376935464	ms	Q68CZ1	1195	S/R	-0,393	0,020723	0,004	**62**	1,1
RPL6	rs754262209	ms	Q02878	248	I/V	0,679	0,024131	0,000	**14**	0,7
RPP25	rs192149396	ms	Q9BUL9	156	P/T	-0,489	0,041293	0,001	**22**	1,1
RPRD2	rs373089873	ms	Q5VT52	1176	E/Q	-0,603	0,044977	**0,539**	**18**	0,4
RPRD2	rs375366241	ms	Q5VT52	780	R/Q	0,956	0,024926	0,000	**10**	8,7
RRBP1	rs1021672375	ms	Q9P2E9	1162	A/T	0,532	0,046064	0,021	**23**	3,0
RREB1	rs140206154	ms	Q92766-2	315	R/C	-0,562	0,026290	**0,997**	**37**	**38**
RRN3	rs774512208	ms	Q9NYV6	111	S/N	0,514	0,037743	0,003	**27**	0,4
RTEL1	rs1568726633	ms	Q9NZ71-6	1215	S/F	-0,428	0,032638	0,377	**34**	0,4
RTN4RL1	rs201050462	ms	Q86UN2	67	G/S	0,358	0,043787	0,003	**59**	**86**
RUFY3	rs764540621	ms	Q7L099-3	601	E/K	0,656	0,008922	0,120	**29**	0,2
RUNDC3B	rs752955187	ms	Q96NL0-5	26	A/V	0,641	0,020537	0,195	**19**	0,4
RXRA	rs779808577	ms	P19793	215	R/W	0,691	0,047013	**0,549**	**18**	0,8
S100PBP	rs757895019	ms	Q96BU1	177	L/F	-0,809	0,001500	0,240	**25**	5,7
S1PR4	rs779911002	ms	O95977	128	A/T	-0,649	0,038833	**0,719**	**14**	4,0
SAFB	rs749699724	ms	Q15424-3	886	R/W	-0,780	0,034375	0,000	**10**	5,1
SAMD1	rs370432894	ms	E9PIW9	291	P/L	0,488	0,021868	0,000	**38**	1,3
SAMD11	rs1557610691	stop	A0A087WU74	625	E/*	0,683	0,038339	**1,000**	**14**	0,1
SAMD14	rs369309802	ms	Q8IZD0	402	R/Q	-0,679	0,020367	0,000	**21**	5,3
SANBR	rs746911974	ms	Q6NSI8	269	N/K	**-1,057**	0,006760	**0,834**	**12**	0
SARDH	rs200897249	ms	Q9UL12	642	D/E	0,449	0,032484	0,013	**53**	**15**
SART1	rs773035344	ms	O43290	86	A/G	0,353	0,032103	0,000	**67**	**15**
SBF2	rs775360425	ms	Q86WG5	1578	K/R	**1,109**	0,033647	0,005	**12**	1,5
SBNO2	rs200703494	ms	Q9Y2G9	142	P/S	**-1,299**	0,026765	0,005	**18**	7,2
SCAF1	rs763412878	ms	Q9H7N4	329	P/A	-0,445	0,027164	0,000	**38**	0
SCAF8	rs147069918	ms	A0A0A0MT33	56	A/V	-0,518	0,003842	0,000	**61**	295
SCAMP4	rs201067147	ms	Q969E2	181	G/R	**-1,149**	0,013593	**0,837**	**11**	1,5
SCAMP4	rs369144370	ms	Q969E2	136	S/L	0,702	0,015522	0,161	**20**	**20**
SCG3	rs748925597	ms	Q8WXD2	323	E/G	0,670	0,041935	**0,987**	**17**	0,1
SCIMP	rs554330535	ms	Q6UWF3-2	109	S/P	-0,410	0,004949	**0,514**	**87**	**63**
SCLY	rs762082693	ms	Q96I15	367	R/Q	0,394	0,035662	0,000	**41**	2,0
SCN3A	rs202004044	ms	Q9NY46-3	563	R/C	-0,530	0,042408	**0,953**	**18**	**15**
SCN4A	rs1567828204	ms	P35499	260	C/Y	-0,858	0,026601	**0,995**	**16**	0
SCN4A	rs202106192	ms	P35499	1564	V/I	0,410	0,000173	0,009	129	209
SCN4A	rs531694454	ms	P35499	1765	A/T	-0,599	0,020264	0,104	**18**	3,8
SCN4A	rs754847636	ms	P35499	148	M/I	-0,469	0,000002168	0,254	167	0
SCN4A	rs78592515	ms	P35499	18	R/S	0,420	0,000002085	0,091	223	174
SCNN1D	rs141251506	ms	P51172-3	739	A/G	-0,501	0,036542	**0,860**	**32**	204
SCP2D1	rs376844337	ms	Q9UJQ7	111	P/L	-0,592	0,009206	**0,646**	**26**	8,3
SCRN1	rs139915877	ms	Q12765	62	V/I	0,615	0,001822	**0,743**	**34**	0,4
SDK1	rs370417123	ms	Q7Z5N4	233	I/L	0,493	0,009438	**0,982**	**48**	**15**
SDK2	rs749556489	ms	Q58EX2	1689	P/L	-0,356	0,044763	0,045	**55**	0,1
SDK2	rs761561326	ms	Q58EX2	885	N/S	-0,406	0,049264	**0,718**	**33**	7,6
SEC14L6	rs751086784	ms	B5MCN3	347	V/L	0,420	0,020258	0,069	**40**	**12**
SEC16A	rs201222771	ms	O15027	346	R/H	0,858	0,014483	0,000	**12**	**94**
SEC16A	rs375839951	ms	O15027	1027	P/L	-0,529	0,024748	0,258	**39**	6,9
SEC23B	rs121918223	ms	Q15437	530	R/W	0,439	0,011174	**1,000**	**50**	0,6
SECISBP2L	rs1566849658	ms	Q93073	1077	G/E	-0,662	0,044472	0,000	**10**	0,2
SEL1L2	rs200767636	ms	Q5TEA6	124	Q/H	-0,772	0,049975	**0,997**	**11**	**35**
SELE	rs138813218	ms	P16581	136	A/S	0,707	0,000913	**0,999**	**49**	0
SELENOF	rs189422667	ms	O60613	38	S/L	0,551	0,029690	0,242	**22**	**87**
SELENON	rs982364753	ms	Q9NZV5	2	G/C	-0,547	0,050071	0,000	**11**	**27**
SEMA3F	rs757110934	ms	Q13275	628	P/A	**-1,202**	0,016683	0,015	**11**	0,7
SEMA3F	rs768027884	ms	Q13275	724	T/M	0,564	0,017273	**0,738**	**26**	3,0
SEMA3G	rs758775695	ms	Q9NS98	728	R/S	0,446	0,017453	**0,904**	**63**	8,1
SEMA4C	rs145704940	ms	Q9C0C4	665	V/M	0,500	0,040567	**0,666**	**13**	163
SEMA4D	rs765159211	ms	Q92854	5	T/I	-0,581	0,019263	0,003	**32**	0
SENP7	rs758250523	ms	Q9BQF6	756	T/A	-0,480	0,046683	0,015	**21**	0,2
SEPTIN3	rs761632941	ms	A0A2R8Y4H2	3	H/Y	-0,826	0,012211	0,000	**14**	**35**
SERINC4	rs554825719	ms	A6NH21	204	A/T	0,948	0,006917	**0,999**	**17**	1,0

SERPINA10	rs142013081	ms	Q9UK55	386	T/A	0,470	0,042241	0,000	35	5,3
SETD1A	rs746152746	ms	O15047	31	A/S	-0,680	0,005272	0,862	43	0,4
SETD2	rs2107638067	ms	Q9BYW2	1982	D/N	0,739	0,023289	0,996	16	0
SFI1	rs756654178	ms	A8K8P3	877	A/V	0,498	0,037112	0,994	28	11
SFPQ	rs1283676090	ms	P23246	640	I/V	0,539	0,033185	0,146	17	0,2
SFXN1	rs769192280	ms	Q9H9B4	301	E/K	-0,377	0,030243	0,701	49	1,3
SFXN5	rs764726890	ms	Q8TD22	268	P/L	-0,955	0,017489	0,968	12	8,3
SGCA	rs765429436	ms	Q16586	351	R/H	0,474	0,022045	0,999	45	1,9
SGCE	rs142744182	ms	A0A2R8YGQ3	46	I/T	0,881	0,011272	0,000	16	214
SGCG	rs774245371	ms	Q13326	115	A/V	0,578	0,030930	0,984	25	1,1
SGCZ	rs748048633	ms	Q96LD1-2	289	G/D	-0,413	0,021895	0,999	35	0,4
SGO1	rs770048758	ms	Q5FBB7-6	379	D/N	-0,892	0,010254	0,715	13	1,9
SGSM1	rs772084972	ms	Q2NKQ1-4	303	D/N	-0,533	0,009606	0,913	27	0
SH2D3C	rs1564413099	ms	Q8N5H7	417	P/R	0,827	0,037400	0,999	13	0,1
SH2D3C	rs61761896	ms	Q8N5H7	443	A/S	-1,026	0,000266	0,003	24	29
SH2D5	rs761666159	ms	Q6ZV89	416	E/K	-0,441	0,013324	0,955	66	2,3
SH2D7	rs145893682	ms	A6NKC9	46	G/S	0,640	0,017325	1,000	22	417
SH3BP4	rs73995756	ms	Q9P0V3	471	N/S	0,486	0,034397	0,000	38	364
SH3D21	rs761828672	ms	A4FU49-6	171	D/N	-0,770	0,014400	0,053	10	0,2
SH3RF3	rs199936909	ms	Q8TEJ3	533	P/L	-0,723	0,037707	0,003	13	110
SH3TC1	rs778072816	ms	Q8TE82	392	S/F	0,482	0,019533	0,950	36	0
SHANK3	rs1013974683	ms	A0A8I5KZC4	945	E/D	0,356	0,022084	0,817	89	0
SHARPIN	rs1428652617	ms	Q9H0F6	49	D/N	0,385	0,034548	0,834	40	0
SHPK	rs144071313	stop	Q9UHJ6	119	R/*	-0,467	0,025112	1,000	37	70
SHROOM1	rs751394319	ms	Q2M3G4	757	E/Q	0,663	0,017393	0,879	15	0,1
SI	rs200451408	stop	P14410	1124	R/*	0,432	0,005961	1,000	77	11
SIK2	rs371556602	ms	Q9H0K1	302	R/Q	-0,743	0,013687	0,191	18	0,6
SIK2	rs748313173	ms	Q9H0K1	397	E/G	0,798	0,018517	0,000	12	0
SIPA1L3	19:38164839:T/C	ms	O60292	1381	Y/H	-0,878	0,002525	0,015	16	N/A
SIRPB1	rs141668681	ms	O00241	180	T/I	0,871	0,026947	0,636	10	60
SIRPB2	rs773180408	ms	Q5JXA9	144	G/D	-0,410	0,048342	0,999	36	0
SKIDA1	rs200920908	ms	Q1XH10	789	R/Q	-0,537	0,024759	0,968	49	63
SLA	rs372398209	ms	Q13239	202	R/K	-1,056	0,006720	0,000	16	2,6
SLC10A7	rs779082973	ms	Q0GE19-2	86	A/T	-0,485	0,013547	0,000	30	0,4
SLC11A2	rs17222449	ms	P49281-2	290	R/Q	-0,626	0,021349	0,031	24	1,5
SLC12A1	rs6493311	stop	Q13621	538	Y/*	-1,023	0,051200	1,000	11	39432
SLC17A2	rs774991867	ms	O00624-3	467	Q/P	0,531	0,007756	0,996	44	1,9
SLC18A1	rs375952046	ms	P54219	157	V/M	0,408	0,006716	1,000	67	4,1
SLC1A5	rs747541617	ms	Q15758	135	F/L	-0,561	0,028837	0,999	37	0
SLC22A2	rs576766802	ms	O15244	136	V/I	0,511	0,029864	0,999	18	2,3
SLC22A23	rs199852691	ms	A1A5C7	122	D/N	0,835	0,001949	0,356	14	38
SLC24A4	rs199781252	ms	Q8NFF2	562	V/M	0,978	0,00008227	0,929	26	0,8
SLC25A23	rs748793177	ms	Q9BV35	74	Y/S	0,395	0,021268	0,997	47	0,7
SLC25A39	rs763200621	ms	Q9BZJ4	303	T/A	0,412	0,046614	0,997	43	0
SLC25A47	rs757658344	ms	Q6Q0C1	73	T/N	-0,811	0,039106	0,703	15	0,1
SLC27A2	15:50182606:T/G	ms	O14975	60	L/R	0,592	0,013739	0,020	35	N/A
SLC27A6	rs199924682	ms	Q9Y2P4	148	I/N	-0,386	0,037309	0,730	73	22
SLC2A1	rs144389023	ms	P11166	417	I/T	0,730	0,032569	0,093	11	6,0
SLC2A10	rs763889166	ms	O95528	194	T/I	-0,496	0,049581	0,039	17	3,0
SLC2A9	rs149454410	ms	Q9NRM0	243	V/I	0,423	0,011714	0,005	61	37
SLC30A5	rs752773548	ms	Q8TAD4	248	L/F	0,400	0,019159	0,015	61	0,4
SLC30A8	rs770454987	ms	Q8IWU4	327	S/T	-0,580	0,030304	0,027	18	4,5
SLC33A1	rs144015992	ms	O00400	484	N/T	0,360	0,022997	0,012	95	193
SLC34A2	rs769110830	ms	O95436	423	G/R	-0,846	0,021499	0,999	15	1,5
SLC34A3	rs202109348	ms	Q8N130	307	L/M	1,617	0,002139	0,223	13	54
SLC35A4	rs1244740037	ms	Q96G79	240	G/S	-0,507	0,051945	0,997	20	1,1
SLC35B4	rs1563214104	ms	Q969S0	246	N/S	0,682	0,002492	0,739	25	0,7
SLC39A4	rs782236196	ms	Q6P5W5	20	A/V	0,824	0,016831	0,000	17	4,2
SLC3A1	rs745473969	ms	Q07837	366	Q/H	-0,979	0,034114	0,028	12	1,9
SLC44A1	rs148518626	ms	Q8WWI5	502	T/A	-0,470	0,036172	0,042	35	72
SLC45A3	rs765451038	ms	Q96JT2	247	R/H	-0,791	0,032130	0,342	17	6,0
SLC46A3	rs200182693	stop	Q7Z3Q1	244	R/*	0,393	0,041880	1,000	49	62
SLC50A1	rs755400797	ms	Q9BRV3	35	M/T	-0,506	0,034739	0,003	18	0
SLC6A12	rs143648821	ms	P48065	203	I/V	0,373	0,007983	0,669	56	28
SLC6A17	rs775258138	ms	Q9H1V8	483	T/M	-1,089	0,027689	0,999	15	0,4
SLC6A20	rs141811843	ms	Q9NP91	549	K/R	-0,389	0,041285	0,010	36	25
SLC6A8	rs782208622	ms	P48029	274	V/M	1,340	0,026390	0,915	18	6,4
SLC7A11	rs762190755	ms	Q9UPY5	119	G/C	-0,770	0,010230	0,999	26	3,0
SLC7A2	rs370916645	ms	P52569	628	A/T	-0,478	0,008467	0,000	45	3,2
SLC8A3	rs144289733	ms	P57103-2	789	V/M	0,427	0,015218	0,999	54	62
SLC9A9	rs191690859	ms	Q8IVB4	254	I/V	0,595	0,039837	0,056	16	1,1
SLCO4A1	rs1244058546	ms	Q96BD0	2	P/S	0,539	0,044974	0,973	17	0,4
SLF2	rs748706051	ms	Q8IX21	133	R/W	-0,582	0,047264	0,529	12	0,8
SLFN12L	rs1567648042	ms	A0A8I5QCZ1	123	N/T	-0,578	0,048330	0,060	22	0
SLTM	rs142696944	ms	Q9NWH9	314	G/V	-0,557	0,037421	0,000	12	25
SLX4	rs114014006	ms	Q8IY92	942	E/Q	0,686	0,048406	0,127	15	1876
SLX4	rs115694169	ms	Q8IY92	385	P/T	0,707	0,031935	0,007	16	987
SLX9	rs140102661	ms	Q9NSI2	180	R/W	0,712	0,006237	0,868	34	6,4
SMARCC1	rs764145318	ms	Q92922	744	A/G	-1,039	0,004475	0,660	18	11
SMARCD2	rs1567759892	ms	Q92925	493	R/T	1,309	0,009592	0,990	12	0
SMARCD2	rs201723860	ms	Q92925	201	T/M	0,478	6,987E-08	0,985	242	15
SMC4	rs1419891152	ms	Q9NTJ3	432	P/S	-0,911	0,030250	0,900	14	0,4
SMCO1	rs11926701	ms	Q147U7	64	R/W	0,766	0,011016	0,000	14	10486
SMCO1	rs1427577811	ms	Q147U7	214	N/K	-0,829	0,002956	0,042	17	0,4

SMDT1	rs776628036	ms	Q9H4I9	5	A/V	0,662	0,046862	0,003	18	12
SMG7	rs1239743198	ms	A0A8I5KYV3	698	P/L	-1,008	0,003779	0,076	17	0,1
SMURF2	rs777947628	ms	Q9HAU4	90	G/D	-0,755	0,003436	0,385	32	0
SMYD2	rs766703409	ms	Q9NRG4	348	G/S	-0,546	0,049639	0,987	30	0,4
SNAI3	rs369333608	ms	Q3KNW1	123	R/W	-0,750	0,041636	0,228	15	3,4
SNAPC4	rs201740904	ms	Q5SXM2	1293	R/C	-0,552	0,016342	0,000	26	19
**SNAPC5**	rs143176301	ms	O75971	12	E/V	-0,656	0,051031	0,000	13	85
**SNAPC5**	rs151153879	ms	O75971	12	E/K	-0,656	0,051031	0,000	13	85
SNRNP35	rs142113918	ms	Q16560	216	P/L	-1,024	0,015954	0,000	18	28
SNTA1	rs141724500	ms	Q13424	147	T/I	0,392	0,004498	0,418	68	95
SNX29	rs150300274	ms	Q8TEQ0	285	T/S	-0,900	0,023700	0,034	10	320
SON	rs184100114	ms	P18583	788	T/A	-0,598	0,045359	0,209	23	6,4
SORCS3	10:105157142:C/G	ms	Q9UPU3	496	A/G	-1,301	0,001605	0,640	14	N/A
SORT1	rs773074476	ms	Q99523	541	S/I	-0,555	0,007670	0,089	22	0,2
SOS1	rs371024396	ms	Q07889	1316	H/D	0,466	0,051443	0,517	22	7,9
SOX5	rs769073811	ms	P35711	635	V/M	-0,440	0,004650	0,978	73	0,2
SP2	rs1211672947	ms	Q02086	251	S/N	0,698	0,029854	0,024	20	0,4
SPAG11B	rs775076868	ms	Q08648	91	L/S	-0,801	0,021330	0,000	13	6,4
SPAG17	rs144147243	ms	Q6Q759	2133	G/D	-0,737	0,010565	0,995	17	4,2
SPART	rs778812974	ms	Q8N0X7	334	R/W	-0,669	0,042445	0,961	24	2,3
SPATA18	rs778351262	ms	Q8TC71	417	S/R	-0,612	0,051557	0,514	13	2,3
SPATA2	rs756953070	ms	Q9UM82	222	T/M	-0,550	0,025242	0,357	20	1,5
SPATA31A3	rs945622093	ms	Q5VYP0	930	S/R	0,472	0,049638	0,292	25	4,9
SPATA31F1	9:34725839:A/C	ms	Q6ZU69	467	D/E	-0,351	0,051933	0,053	44	N/A
SPATS2	12:49526133:C/A	ms	Q86XZ4	506	H/N	-0,611	0,050625	0,053	11	N/A
SPCS3	rs762898441	ms	P61009	116	P/L	-0,954	0,015476	0,220	12	0,4
SPDYE21	rs567907678	ms	A0A494C086	191	V/M	0,530	0,048343	0,860	21	6,8
SPDYE2B	rs1791901438	ms	A6NHP3	246	I/V	1,159	0,005486	0,332	18	0
SPECC1L	rs202183866	ms	Q69YQ0	914	Q/E	-0,554	0,041995	0,091	28	7,4
**SPEG**	rs373080805	ms	Q15772-5	2131	F/L	1,171	0,012117	0,095	10	2,6
**SPEM1**	rs191212954	ms	Q8N4L4	185	V/I	-0,378	0,027498	0,000	52	670
**SPEM1**	rs571769033	ms	Q8N4L4	253	R/W	0,870	0,009140	0,663	14	2,6
**SPEN**	rs1438984165	ms	Q96T58	1017	K/E	-0,598	0,041807	0,934	21	0,8
**SPEN**	rs766851755	ms	Q96T58	2013	A/T	-0,354	0,037955	0,066	37	3,0
**SPEN**	rs773726905	ms	Q96T58	2880	V/M	0,372	0,039668	0,013	48	0,4
SPG11	rs1216723582	ms	Q96JI7	1155	P/T	0,820	0,018216	0,998	18	1,1
SPHK1	rs1567822886	ms	Q9NYA1	33	L/V	-0,635	0,004021	0,301	34	0
SPMIP9	rs201634174	ms	Q96LM6	49	R/W	0,848	0,031485	0,000	16	24
**SPPL2C**	rs748111980	ms	Q8IUH8	600	S/T	-0,663	0,023520	0,077	29	1,0
**SPPL2C**	rs753862379	ms	Q8IUH8	542	S/L	-0,522	0,001102	0,025	74	3,8
**SPPL2C**	rs1490540043	stop	Q8IUH8	567	R/*	-0,901	0,013577	1,000	14	1,1
SPRED1	rs765603808	ms	Q7Z699	334	R/C	0,513	0,015005	0,994	37	2,3
SPRR3	rs1557889683	ms	Q9UBC9	23	Q/L	0,873	0,021308	0,000	15	0
SPRY4	rs200364529	ms	Q9C004	258	V/M	-0,370	0,005690	0,423	73	28
SPRYD3	rs776074767	ms	Q8NCJ5	72	R/Q	0,696	0,020823	0,028	28	0,4
SPTAN1	rs779759134	ms	Q13813-2	1039	A/T	0,507	0,028535	0,001	27	1,5
SPTBN2	rs769616053	stop	O15020-2	2356	R/*	0,524	0,044905	1,000	27	9,4
**SPTBN4**	rs748745941	ms	Q9H254	818	L/F	0,477	0,008397	0,033	49	0,4
**SPTBN4**	rs753897679	ms	Q9H254	163	R/C	-1,152	0,000476	1,000	13	0,8
**SPTBN5**	rs201037092	ms	Q9NRC6	3600	R/W	-0,471	0,023701	0,005	41	11
**SPTBN5**	rs756377333	ms	Q9NRC6	372	R/Q	-1,033	0,026587	0,018	16	1,1
SRP68	rs771151407	ms	Q9UHB9	76	R/Q	0,826	0,008951	0,783	13	0,7
SRP72	rs17524437	ms	O76094	7	G/W	0,464	0,019956	0,966	30	0
**SRRM2**	rs1323436648	ms	Q9UQ35	2031	R/K	0,567	0,050105	0,000	18	0,4
**SRRM2**	rs139637110	ms	Q9UQ35	2485	S/F	0,834	0,011406	0,904	22	124
SSC5D	rs748135422	ms	A1L4H1	867	V/M	-0,367	0,009253	0,997	88	3,0
SSRP1	rs201685642	ms	Q08945	606	D/H	0,642	0,007345	0,300	21	7,9
ST3GAL1	rs762669104	ms	Q11201	232	P/L	-0,627	0,019942	0,671	29	0,8
ST3GAL4	rs765192401	ms	Q11206	120	C/Y	-1,186	0,005391	1,000	11	0,8
ST6GALNAC5	rs752770582	ms	Q9BVH7	268	P/L	0,415	0,040983	0,997	44	0,2
ST8SIA6	rs200933958	ms	P61647	315	G/S	0,363	0,039419	0,994	57	15
STAB2	rs141041254	ms	Q8WWQ8	2377	E/K	0,675	0,040900	0,374	12	52
STAG3	rs1562987401	fs	D6W5U7	703	L/X	0,482	0,034151	1,000	47	0
STAT5A	rs759535072	ms	P42229	191	Q/H	-0,587	0,048324	0,417	14	0,1
STAU2	rs532770072	ms	Q9NUL3	427	R/L	-0,683	0,018067	0,037	29	0,4
STC1	rs146703503	ms	P52823	102	G/R	0,825	0,012403	1,000	14	17
STK10	rs753084325	ms	O94804	918	R/Q	1,245	0,006678	0,860	11	1,9
STK11	rs1169900277	ms	A0AAQ5BHW9	573	R/H	1,068	0,040474	0,033	13	0,4
STK32A	rs200548298	ms	Q8WU08	109	R/H	-0,490	0,045418	0,999	23	24
STOX1	rs372305853	ms	Q6ZVD7	639	H/R	-0,694	0,043510	0,000	12	9,4
STOX2	rs1734108048	ms	Q9P2F5	591	C/R	-0,881	0,023839	0,963	14	0,4
STPG1	rs560627804	ms	Q5TH74	305	G/V	0,600	0,000916	0,239	44	9,4
STPG2	rs1479947625	stop	Q8N412	13	E/*	0,929	0,029064	1,000	10	0,2
STPG3	rs752473487	ms	Q8N7X2-4	238	L/V	0,608	0,001892	0,001	29	5,9
STRADA	rs750512077	ms	Q7RTN6	215	R/H	-0,405	2,801E-09	0,999	352	1,5
STRN4	rs765739479	ms	Q9NRL3	373	P/S	0,358	0,001366	0,992	112	1,5
STX5	rs564823443	ms	Q13190	193	V/I	0,404	0,014596	0,729	55	9,1
SUCLG2	rs201519398	ms	Q96I99	374	N/S	-0,573	0,026768	0,060	24	20
SULF1	rs140542311	ms	Q8IWU6	359	V/I	-0,680	0,030464	0,249	13	31
SULF2	rs752358752	ms	Q8IWU5	205	R/H	-0,688	0,003884	1,000	28	1,3
SULT1A3	rs2073441082	ms	P0DMM9	108	H/Y	-0,362	0,010698	0,944	84	0,3
SUN1	rs779427205	ms	O94901-8	406	G/A	-0,533	0,002839	0,061	45	0
SUPT3H	rs150635035	ms	O75486	88	R/H	0,505	0,024291	0,996	19	1,1
SUPV3L1	rs199507911	ms	Q8IYB8	415	N/S	-0,775	0,007378	0,003	22	24

**SUSD2**	rs766473126	ms	Q9UGT4	576	P/S	1,021	0,005562	0,231	10	0,8
**SUSD2**	rs776550297	stop	Q9UGT4	168	Y/*	-0,393	0,050349	1,000	35	0,2
SUZ12	rs1272910292	ms	Q15022	691	G/V	0,663	0,005657	0,001	30	0
SYCN	rs766050306	ms	Q0VAF6	35	T/R	0,393	0,024871	0,691	52	0,4
**SYNE2**	rs199743242	ms	Q8WXH0-2	2171	L/V	-0,775	0,024441	0,995	24	51
**SYNE2**	rs200319405	ms	Q8WXH0-2	757	L/S	-0,775	0,024416	0,825	24	34
**SYNE2**	rs750823503	ms	Q8WXH0-2	4	S/T	-0,425	0,018640	0,006	63	0,4
SYNPO	rs371137506	ms	Q8N3V7	39	E/D	0,651	0,011639	0,871	23	4,9
SYNPO2L	rs1564989451	ms	Q9H987	894	A/S	-0,513	0,032216	0,688	20	0,1
SYT12	rs141165304	ms	Q8IV01	380	R/H	0,490	0,016793	0,017	22	119
SYT4	rs149020215	ms	Q9H2B2	384	R/Q	1,084	0,001435	0,009	15	15
SYT6	rs370048571	ms	Q5T7P8	481	E/K	0,545	0,030816	0,998	23	1,3
SYTL1	rs1557548678	ms	Q8IYJ3	471	P/S	0,845	0,047288	1,000	15	0,2
**SZT2**	rs532357677	ms	Q5T011	1574	R/Q	-0,402	0,008728	0,264	58	1,9
**SZT2**	rs757113547	ms	Q5T011	1444	R/H	-0,402	0,008728	0,000	59	0,4
TAAR1	rs753259390	ms	Q96RJ0	7	N/T	0,611	0,037382	0,022	26	0,4
TACC2	rs142194146	ms	O95359-4	948	R/W	0,469	0,005440	0,117	72	29
TAF7	rs139292924	ms	Q15545	131	I/M	0,630	0,045063	0,723	24	74
TAFA1	rs372722058	ms	Q7Z5A9	119	A/T	0,923	0,031314	0,145	12	3,0
TALDO1	rs1804554	ms	P37837	300	E/K	0,914	0,031960	0,953	15	3,4
TANC1	2:159219263:G/A	ms	Q9C0D5	1135	G/D	-1,032	0,015280	0,186	10	N/A
**TANC2**	rs370863314	ms	A0A8I5KXR5	2015	R/P	0,479	8,155E-09	0,066	232	12
**TANC2**	rs554732900	ms	A0A8I5KXR5	1884	R/Q	-0,388	0,009026	0,114	78	0,4
**TANC2**	rs746422304	ms	A0A8I5KXR5	273	G/E	0,828	0,008412	0,063	12	7,2
**TANC2**	rs780001108	ms	A0A8I5KXR5	160	A/T	-0,441	0,000145	0,896	132	2,3
TAOK2	rs138875458	ms	Q9UL54-2	750	I/V	0,723	0,048268	0,087	16	19
TARBP2	rs1565897406	ms	Q15633	12	T/A	0,886	0,022850	0,022	20	0
**TAS1R1**	rs140284805	ms	Q7RTX1	470	T/A	0,427	0,004335	0,087	71	137
**TAS1R1**	rs200196173	ms	Q7RTX1	678	G/S	0,377	0,001659	1,000	105	3,6
TAS1R3	rs763938709	ms	Q7RTX0	500	S/L	-0,887	0,015586	0,686	16	0,8
TAS2R19	rs767546611	ms	P59542	260	V/L	-0,462	0,037825	0,062	30	0,1
TAS2R3	rs771250168	ms	Q9NYW6	274	M/V	-0,446	0,043922	0,072	30	0,4
TAS2R31	rs201717335	ms	P59538	92	G/S	0,712	0,008217	0,007	19	58
TAS2R5	rs147887777	ms	Q9NYW4	55	R/Q	-0,546	0,022841	0,970	38	168
**TBC1D16**	rs1020548320	ms	Q8TBP0	55	L/R	-1,746	0,014260	0,161	17	7,1
**TBC1D16**	rs755651950	ms	Q8TBP0	116	T/I	-0,478	0,037211	0,000	29	8,7
TBC1D2	rs767385150	ms	Q9BYX2	245	E/V	0,486	0,007072	0,368	58	1,5
TBC1D31	rs780616960	ms	Q96DN5	487	W/R	0,361	0,030574	1,000	44	1,5
TBC1D32	rs200973240	ms	Q96NH3	770	R/G	1,184	0,049320	0,021	10	21
TBC1D3B	rs1231609801	ms	A6NDS4	284	R/H	-0,659	0,006936	0,941	34	80
TBC1D3F	rs1184505888	ms	A0A087WT91	323	R/S	0,448	0,037997	0,009	37	0
TBC1D8	rs201324556	ms	J3KQ40	509	R/H	0,523	0,015971	0,983	30	41
TBCD	rs761639016	ms	Q9BTW9	579	I/T	0,644	0,022827	0,823	19	0
TBCE	rs143917509	ms	Q15813	489	L/I	-0,421	0,014077	0,009	55	80
**TBCK**	rs766079704	ms	Q8TEA7	344	E/Q	-0,430	0,028330	0,012	60	0,7
**TBCK**	rs781374842	ms	Q8TEA7	43	Q/R	-0,685	0,014036	0,988	30	1,9
TBX10	rs149888346	ms	O75333	200	F/L	0,485	0,017763	0,106	48	25
TBX21	rs2032313569	ms	Q9UL17	417	M/V	-0,971	0,001800	0,000	17	2,9
**TBX3**	rs1012420316	ms	O15119-2	71	A/S	0,424	0,037558	0,973	25	0,9
**TBX3**	rs762031545	ms	O15119-2	454	P/L	-0,580	0,037356	0,001	25	6,0
**TCAF2**	7:143723411:C/G	ms	A6NFQ2	710	R/G	0,663	0,022168	0,928	15	N/A
**TCAF2**	rs1809518551	ms	A6NFQ2	518	C/R	-0,390	0,009113	0,584	81	0
TCF3	rs138963927	ms	P15923	77	E/K	0,377	0,045207	0,134	49	11
TCF7L1	rs1434102693	ms	Q9HCS4	67	S/L	-0,988	0,002769	0,015	14	0,8
TCTE1	rs146833594	ms	Q5JU00	310	E/K	0,474	0,022987	0,163	35	152
TDP2	rs763334299	ms	O95551	282	D/A	0,739	0,001455	1,000	34	0
TDRD10	rs146616270	ms	Q5VZ19-2	263	A/T	-0,585	0,036376	0,996	18	92
TEC	rs761984494	ms	P42680	269	G/D	0,729	0,006290	0,413	16	0
TECTB	rs746498190	ms	Q96PL2	214	Y/H	0,663	0,022162	0,901	23	4,2
TEPSIN	rs777674986	ms	A0A1B0GV70	593	A/S	0,699	0,034135	0,005	10	2,0
**TEX14**	rs1454744660	ms	Q8IWB6-3	291	E/K	-0,741	0,008761	0,826	31	1,9
**TEX14**	rs775971945	ms	Q8IWB6-3	537	Y/C	-0,790	0,000412	0,010	33	0,4
TEX15	rs1563237429	ms	A0A2R8Y358	1595	H/R	0,688	0,013468	0,006	20	0,4
**TEX2**	rs372604630	ms	Q8IWB9	496	S/R	0,593	0,040906	0,595	18	15
**TEX2**	rs780968826	ms	Q8IWB9	375	E/K	0,366	9,003E-10	0,003	486	0,5
TEX29	rs375512703	ms	Q8N6K0	54	A/V	0,433	0,051362	0,871	36	2,3
TF	rs121918677	ms	P02787	671	G/E	-0,373	0,051829	1,000	35	275
TG	rs781688385	ms	P01266	2123	F/Y	0,458	0,001178	0,740	76	0,2
TGFBI	rs201158209	ms	Q15582	354	N/S	-1,230	0,041089	0,261	11	24
TGFBR3	rs137909765	ms	Q03167	790	I/F	0,353	0,021755	0,999	76	82
THEM4	rs776286488	ms	Q5T1C6	12	L/P	-0,358	0,050404	0,553	61	2,1
THEMIS	rs373934545	ms	Q8N1K5	251	E/Q	-1,069	0,003751	0,990	11	2,6
TIAM1	rs16987932	ms	Q13009	844	Q/H	-0,905	0,006001	0,003	17	5119
TIGIT	rs13098836	ms	Q495A1	33	I/V	0,557	0,023424	0,039	34	278
TIMELESS	rs766677008	ms	Q9UNS1	753	R/C	0,433	0,041829	0,916	30	6,0
TIMM21	rs145075415	ms	Q9BVV7	127	T/M	-0,713	0,040170	0,723	16	1,9
TIMP2	rs769578055	ms	P16035	173	L/P	0,538	0,001621	1,000	49	5,3
TJAP1	rs1561823029	ms	Q5JTD0	123	S/N	0,649	0,024771	0,567	20	0
**TKT**	rs757757097	ms	P29401	350	I/V	-0,444	0,002308	0,010	72	1,5
**TKT**	rs781993830	ms	P29401	559	V/M	-0,908	0,001606	1,000	30	2,3
**TKT**	rs762983878	stop	P29401	514	E/*	-0,371	0,001071	1,000	128	1,1
TLE6	rs373921962	ms	Q9H808	133	R/Q	0,690	0,028007	0,044	14	3,8
TLK2	rs746810639	ms	Q86UE8-2	70	Y/H	-0,903	0,029379	0,003	11	0,1
TLR3	rs73025939	ms	O15455	643	R/C	1,422	0,00004264	0,985	11	138

TM6SF2	rs763226075	ms	Q9BZW4	109	G/A	0,415	0,020088	**0,996**	**50**	0,8
TMC6	rs147815166	ms	Q7Z403	583	R/Q	0,579	0,024841	0,022	**26**	**98**
TMC8	rs144981818	ms	Q8IU68	131	V/L	0,735	0,012062	0,354	**18**	5,6
TMCO3	rs764955516	ms	Q6UWJ1	561	A/T	-0,559	0,046625	0,062	**28**	0,8
TMED4	rs771263996	ms	Q7Z7H5	36	E/K	0,678	0,014931	**0,675**	**29**	0,2
TMEM101	rs756067708	ms	Q96IK0	136	R/H	0,680	0,005484	**0,991**	**21**	0,6
TMEM131	rs889603986	ms	Q92545	13	T/N	0,530	0,046246	0,000	**26**	**42**
TMEM138	rs187122512	ms	A0A8I5QKQ3	129	E/K	0,423	0,035437	0,000	**37**	274
TMEM151B	rs753916563	ms	Q8IW70	408	G/D	0,438	0,028596	0,125	**36**	1,9
TMEM161B	rs547282299	ms	E9PCX5	443	P/L	**-1,017**	0,026711	0,000	**14**	**17**
TMEM176B	rs753351031	ms	Q3YBM2	171	F/V	0,503	0,027036	0,000	**26**	4,3
TMEM208	rs374909823	ms	Q9BTX3	161	R/Q	0,375	0,036014	0,000	**42**	3,4
TMEM229A	rs376966840	ms	B2RXF0	266	G/E	0,595	0,039742	0,153	**29**	3,8
TMEM231	rs199605221	ms	Q9H6L2	264	E/A	0,468	0,019860	**0,897**	**35**	**76**
TMEM233	rs371582871	ms	B4DJY2	83	K/M	-0,684	0,007155	**0,808**	**29**	7,9
TMEM238L	rs200099798	ms	A6NJY4	10	C/R	-0,761	0,015198	**0,598**	**19**	**79**
TMEM259	rs1568399471	fs	Q4ZIN3	427	S/X	**-1,299**	0,026843	**1,000**	**15**	0
TMEM262	rs765511199	ms	E9PQX1	31	G/D	0,821	0,026048	**0,970**	**16**	**12**
TMEM44	rs142364954	ms	Q2T9K0-2	369	V/I	0,359	0,018037	0,007	**56**	**28**
TMEM88	rs201535986	ms	Q6PEY1	111	R/H	0,498	0,024512	**0,711**	**28**	**42**
TMF1	rs1559628559	ms	P82094	921	R/H	0,670	0,042366	0,005	**13**	0,1
TMF1	rs768748751	ms	P82094	284	S/L	**1,060**	0,042153	0,170	**11**	0
TMPRSS11B	rs575638339	ms	Q86T26	207	R/H	0,944	0,042826	0,003	**13**	2,6
TMPRSS13	rs749737407	ms	Q9BYE2-4	460	P/S	0,365	0,023288	0,042	**73**	0,1
TMPRSS5	rs1263487635	ms	Q9H3S3	249	A/V	0,909	0,030113	**0,482**	**15**	1,1
TMX1	rs766355722	ms	Q9H3N1	269	R/C	0,535	0,044735	0,024	**27**	1,9
TNC	rs139280264	ms	P24821	1066	R/C	-0,536	0,030794	**0,577**	**23**	**83**
TNC	rs149986851	ms	P24821	203	G/V	0,369	0,030467	**0,980**	**65**	**28**
TNC	rs751818291	ms	P24821	1903	A/S	-0,477	0,003632	**0,999**	**62**	0,4
TNFRSF9	rs183916313	ms	Q07011	193	A/V	-0,467	0,015713	0,030	**36**	3,0
TNFSF8	rs776716866	ms	P32971	58	M/T	-0,936	0,003198	0,111	**19**	1,9
TNIP1	rs768349035	stop	Q15025-2	636	*/Q	0,569	0,024516	**1,000**	**21**	0,1
TNNT2	rs367785431	ms	P45379	296	R/C	**-1,161**	0,000845	**0,924**	**14**	1,9
TNPO1	rs772921669	ms	Q92973	749	I/M	0,871	0,040867	0,010	**14**	1,7
TNR	rs770776102	ms	Q92752	347	D/Y	-0,366	0,047540	**0,990**	**48**	0
TNRC18	rs200928367	ms	O15417	1549	G/S	**1,262**	0,003029	0,000	**13**	**46**
TNRC6A	16:24789589:C/G	ms	Q8NDV7	316	S/C	-0,687	0,048084	0,308	**15**	N/A
TNRC6B	rs577376433	ms	Q9UPQ9-3	556	P/S	**1,102**	0,000748	**0,986**	**19**	3,8
TNS1	rs767046731	ms	Q9HBL0-3	176	H/R	-0,540	0,023520	**0,996**	**26**	0,4
TNS3	rs201649683	ms	Q68CZ2	815	V/I	0,435	0,001819	0,000	**84**	**45**
TNXB	rs185207099	ms	P22105-3	1163	G/E	-0,826	0,025638	**0,869**	**14**	**73**
TNXB	rs190411129	ms	P22105-3	2798	R/C	-0,506	0,006461	**0,931**	**55**	**23**
TOGARAM1	rs770089950	ms	G3XAE9	1176	S/P	0,843	0,046743	**0,997**	**12**	0,2
TOP2A	rs746667611	ms	P11388	1118	V/I	**-1,173**	0,008711	0,066	**17**	1,5
TOP2A	rs764177670	ms	P11388	530	T/M	0,506	0,012587	**0,931**	**30**	6,4
TOP3A	rs139844084	ms	Q13472	629	E/K	0,392	0,006224	0,213	**97**	244
TOP3A	rs777874588	ms	Q13472	542	I/M	-0,799	0,021123	**0,969**	**20**	1,1
TOP3B	rs1250857443	ms	O95985	755	A/V	-0,772	0,000118	0,000	**27**	0,8
TOPORS	rs200405067	ms	Q9NS56	118	R/C	0,375	0,032095	0,000	**53**	**16**
TPCN1	rs199608038	ms	Q9ULQ1	728	R/Q	0,813	0,002531	0,013	**25**	2,3
TPCN2	rs34510004	ms	Q8NHX9	546	M/I	0,856	0,044722	0,003	**16**	**63**
TPM3	rs755506890	ms	P06753	278	A/V	0,406	0,046156	0,189	**27**	0,2
TPMT	rs1800462	ms	P51580	80	A/P	0,685	0,017907	**0,972**	**24**	255
TPTE2	rs747723029	ms	Q6XPS3	399	K/I	-0,455	0,012198	**0,927**	**61**	119
TRAF3IP1	rs749544012	ms	Q8TDR0	442	P/T	0,930	0,007497	0,000	**17**	0,4
TRANK1	rs559712651	ms	A0A2R8YEM9	1500	R/W	-0,659	0,022770	**0,812**	**25**	2,3
TRANK1	rs749619179	ms	A0A2R8YEM9	822	I/L	0,548	0,035741	0,005	**18**	0
TRAP1	rs148549350	ms	Q12931	469	R/C	**1,098**	0,005334	**0,999**	**15**	**35**
TRAPPC12	rs777185825	ms	Q8WVT3	212	D/E	-0,452	0,037985	0,029	**37**	**15**
TRAPPC2B	rs1168689303	ms	P0DI82	98	F/L	-0,554	0,038417	**0,959**	**19**	1,1
TRAPPC9	rs143778652	ms	Q96Q05	340	A/V	-0,752	0,030515	**0,987**	**12**	**55**
TRAPPC9	rs771541938	ms	Q96Q05	409	R/C	-0,767	0,010254	**0,990**	**15**	0,4
TREM1	rs756591461	ms	Q9NP99	112	Q/H	-0,432	0,025425	**0,451**	**58**	0,1
TRIM11	rs1558445080	ms	Q96F44	303	S/F	-0,949	0,016106	**0,985**	**11**	0,4
TRIM11	rs758801204	ms	Q96F44	147	R/Q	0,647	0,049985	0,005	**19**	4,2
TRIM22	rs780626396	ms	Q8IYM9	74	L/R	**1,771**	0,003284	**0,995**	**13**	**26**
TRIM37	rs143642427	ms	O94972	789	D/E	-0,395	0,000341	0,005	141	**40**
TRIM37	rs376721539	ms	O94972	777	A/T	0,566	0,038809	**0,626**	**16**	4,9
TRIM37	rs771449822	ms	O94972	51	C/R	-0,773	0,025588	**1,000**	**11**	0
TRIM44	rs777604610	ms	Q96DX7	334	G/R	0,487	0,043492	0,000	**19**	0,8
TRIM67	rs534389734	ms	Q6ZTA4-3	273	G/D	0,357	0,021432	**0,960**	**72**	154
TRIM71	rs781070829	ms	Q2Q1W2	336	R/Q	0,505	0,030332	0,249	**34**	**11**
TRIM72	rs757603858	ms	Q6ZMU5	196	R/C	0,665	0,036015	**0,755**	**15**	1,1
TRIM72	rs780638717	ms	Q6ZMU5	206	R/L	0,447	0,032042	0,001	**30**	**17**
TRIM75	rs750797794	ms	A6NK02	176	V/M	-0,639	0,033711	0,120	**59**	0,2
TRIO	rs1487014323	ms	O75962	702	S/L	-0,771	0,036487	0,000	**11**	1,1
TRIO	rs201650861	ms	O75962	507	S/L	**-1,500**	0,041992	0,000	**15**	1,5
TRIO	rs892410126	ms	O75962	2310	G/V	-0,420	0,020194	0,009	**30**	**16**
TRIP11	rs1566861259	ms	Q15643	534	Q/E	-0,512	0,042055	0,000	**17**	0
TRIP6	rs778314148	ms	Q15654	160	A/P	0,509	0,044196	**0,965**	**19**	0,4
TRIP6	rs1310784349	ms	Q15654	374	G/D	**-1,422**	0,018084	**0,958**	**11**	0,8
TRMT2A	rs771115771	ms	Q8IZ69	604	P/A	0,409	0,021942	0,001	**56**	0,3
TRMT44	rs778957821	ms	Q8IYL2	11	Y/D	0,614	0,029604	0,000	**15**	0,4
TRMT6	rs148182864	ms	Q9UJA5	166	R/C	-0,612	0,029056	**0,879**	**29**	**44**

TRNAU1AP	rs774377370	ms	Q9NX07	267	A/T	0,505	0,011431	0,879	41	0,8
TRPC3	rs765481480	ms	Q13507-2	108	S/N	1,014	0,000277	0,007	16	0
TRPC6	rs745835425	ms	Q9Y210	870	A/G	-0,536	0,046867	0,697	19	0,3
TRPM2	rs747955952	ms	O94759	816	V/M	-0,847	0,029540	0,942	10	1,6
TRPM8	rs139760142	ms	Q7Z2W7	939	L/M	-0,607	0,010846	0,159	26	2,3
TRPM8	rs370999195	ms	Q7Z2W7	607	D/N	0,589	0,024029	0,285	27	0,8
TSC2	rs45482691	ms	P49815	91	P/L	-0,434	0,045534	0,998	35	53
TSC2	rs45517203	ms	P49815	607	A/T	0,398	0,044683	0,996	49	53
TSPAN33	rs150641480	ms	Q86UF1	139	D/H	0,763	0,038298	0,995	15	7,6
TSPOAP1	rs139104560	ms	O95153	1584	A/P	0,428	0,005177	0,000	63	59
TTBK2	rs758756132	ms	Q6IQ55	698	M/V	0,466	0,016410	0,000	40	0,4
TTC13	rs202049212	ms	Q8NBP0	840	T/M	-0,363	0,036853	0,701	58	31
TTC21A	rs199900796	ms	A0A804HK20	650	P/T	0,418	0,017130	0,021	42	23
TTC23	rs774316772	stop	Q5W5X9	401	Q/*	0,887	0,024383	1,000	13	0
TTC28	rs376490616	ms	Q96AY4	550	R/C	-0,558	0,019721	0,999	28	34
TTC3	rs1568881314	fs	P53804	169	L/X	-0,491	0,026343	1,000	34	0
TTLL12	rs1398457638	ms	Q14166	503	N/S	0,702	0,006262	0,027	21	1,2
TTLL2	rs376612341	ms	Q9BWV7	4	R/Q	-0,356	0,009325	0,000	115	3,8
TTLL5	rs367910225	ms	Q6EMB2	697	G/S	-0,565	0,012336	0,000	33	7,9
TTLL7	rs1557607066	ms	Q6ZT98	657	A/D	-0,819	0,006569	0,000	18	0
TTN	rs150667217	ms	A0A0A0MTS7	22197	V/A	0,495	0,000429	0,149	84	26
TTN	rs1559263086	ms	A0A0A0MTS7	17261	E/G	-0,691	0,036235	0,020	14	0,2
TTN	rs201825412	ms	A0A0A0MTS7	18019	R/Q	0,444	0,001918	0,016	83	15
TTN	rs374656017	ms	A0A0A0MTS7	25580	R/Q	-0,587	0,020496	0,990	35	15
TTN	rs56372592	ms	A0A0A0MTS7	1202	T/K	0,495	0,000431	0,012	84	239
TTN	rs758449770	ms	A0A0A0MTS7	21335	E/K	-0,587	0,020514	0,752	35	0
TTN	rs773791222	ms	A0A0A0MTS7	29106	E/K	0,590	0,008012	0,370	41	6,0
TUBD1	rs776246231	ms	Q9UJT1	151	G/R	0,498	0,009185	1,000	42	0,8
TUBGCP6	22:50221172:C/A	ms	Q96RT7	1063	V/F	0,567	0,001637	0,760	64	N/A
TUBGCP6	22:50221173:C/G	ms	Q96RT7	1062	R/S	0,567	0,001637	0,150	64	N/A
TUFM	rs760673155	ms	P49411	144	T/A	-0,401	0,028354	0,000	41	0,4
TXNDC11	rs146197324	ms	Q6PKC3-2	875	R/H	-0,411	0,023922	0,001	42	33
TXNDC11	rs151281237	ms	Q6PKC3-2	900	T/S	-0,542	0,036761	0,035	27	34
TXNDC16	rs147641850	ms	Q9P2K2	331	P/S	0,607	0,043812	0,080	18	36
TXNIP	rs150672123	ms	Q9H3M7	312	R/Q	-0,721	0,007139	0,000	23	50
TYW1	rs370745036	ms	Q9NV66	518	A/V	0,731	0,010431	0,005	21	3,8
UBAP1	rs1563920368	ms	Q9NZ09	175	D/N	0,616	0,028492	0,990	17	0,2
UBAP2L	rs766782624	ms	Q14157-2	763	G/S	-0,504	0,040444	0,003	29	0,1
UBD	rs200363304	ms	O15205	138	R/K	-1,358	0,009295	0,001	13	6,8
UBXN6	rs200014531	ms	Q9BZV1	45	R/C	-0,734	0,025889	0,940	17	7,2
UCHL5	rs146351256	ms	Q9Y5K5-3	321	A/T	1,088	0,005784	0,000	14	38
UCN3	rs782142877	ms	Q969E3	94	R/W	1,168	0,012078	0,997	12	1,9
UFL1	rs760861177	ms	O94874	240	D/N	-0,517	0,035256	0,878	30	2,6
UFM1	rs1566029075	ms	P61960	5	S/F	0,782	0,046079	0,710	16	0,4
UGGT2	rs1566664454	fs	Q9NYU1	798-799	-/X	-0,888	0,015228	1,000	22	0
UGP2	rs762421534	ms	Q16851	81	P/H	0,394	0,003477	0,934	99	0,8
UGT1A4	rs760663829	ms	P22310	258	R/Q	0,789	0,022985	0,999	16	1,1
UGT2A3	rs137969795	ms	Q6UWM9	394	F/S	0,600	0,002782	0,994	49	9,1
UGT2B17	rs186138322	ms	O75795	480	A/T	-1,453	0,000228	0,921	15	59
UGT3A1	rs766764383	ms	Q6NUS8	334	S/C	0,707	0,041943	0,985	12	0
ULK1	rs146186922	ms	O75385	660	T/M	0,751	0,000718	0,959	31	5,3
UMOD	rs199835347	ms	P07911	142	R/Q	0,771	0,019467	0,014	10	144
UMODL1	rs757493344	ms	Q5DID0	30	S/C	0,731	0,023657	0,997	17	1,1
UNC119	rs146916036	ms	Q13432	168	R/C	-0,797	0,021447	1,000	35	45
UNC13B	rs1829737656	ms	A0A1B0GUS7	1047	V/I	-0,997	0,031536	0,000	13	0
UNC45A	rs566664532	ms	Q9H3U1	358	P/A	0,752	0,031122	0,005	12	1,1
UPF3A	rs376489246	ms	Q9H1J1	63	K/E	0,551	0,034245	0,184	27	3,4
URB1	rs771374770	ms	O60287	105	I/V	-0,574	0,042054	0,556	14	2,3
URB2	rs766210522	ms	Q14146	1063	L/F	-0,453	0,046785	0,554	23	0,3
URI1	rs772890952	ms	O94763	176	R/Q	-0,418	0,030960	0,997	45	1,5
USF3	rs745332813	ms	Q68DE3	196	V/L	-1,454	0,005321	0,048	16	2,5
USH2A	rs139089840	ms	O75445	1076	P/S	-0,390	0,047317	0,998	38	23
USP13	rs1488181405	ms	Q92995	285	A/S	-0,992	0,011828	0,028	15	0,4
USP17L2	rs1563163368	ms	Q6R6M4	19	K/R	-0,776	0,025689	0,618	12	0,7
USP17L2	rs201369910	ms	Q6R6M4	156	G/C	0,351	0,035464	0,985	50	49
USP17L24	4:9326086:T/A	ms	Q0WX57	308	S/T	-0,449	0,027330	0,971	37	N/A
USP17L25	4:9330832:T/A	ms	Q0WX57	308	S/T	-0,449	0,027326	0,971	38	N/A
USP17L26	4:9335579:T/A	ms	Q0WX57	308	S/T	-0,449	0,027321	0,971	38	N/A
USP17L3	rs1820676921	ms	A6NCW0	77	A/V	0,458	0,045963	0,012	31	0
USP19	rs1559995833	ms	O94966-6	1082	S/R	-0,946	0,016492	0,008	16	0
USP19	rs1560004912	ms	O94966-6	884	P/S	0,616	0,040856	0,446	17	0,1
USP22	rs752874123	ms	Q9UPT9	363	T/M	-0,365	0,051370	0,046	61	0,3
USP28	rs142728209	ms	A0A8V8TLZ9	631	R/I	1,220	0,042863	0,698	11	42
USP32	rs140463372	ms	Q8NFA0	1370	I/S	-0,717	0,051820	0,000	14	13
USP36	rs1567975159	ms	Q9P275	44	P/S	-0,956	0,034809	0,998	13	0,2
USP40	rs1559237710	ms	A0A7I2YQ75	846	F/L	0,706	0,041838	0,236	12	0
USP42	rs200908439	ms	Q9H9J4-2	486	G/R	-0,989	0,011678	0,872	15	18
USP43	rs777462170	ms	Q70EL4	872	L/V	-0,413	0,042508	0,006	51	0
UTP15	rs776437309	ms	Q8TED0	288	Y/D	1,216	0,043359	0,973	10	0
UTP4	rs769827893	stop	Q969X6	546	S/*	-0,366	0,020253	1,000	58	0,8
UTRN	rs748187376	ms	P46939	3310	E/D	-0,408	0,020505	0,003	55	1,5
VAC14	rs145938865	ms	Q08AM6	575	R/W	-0,474	0,042376	0,901	36	76
VASH1	rs746572157	ms	Q7L8A9	334	R/C	-0,522	0,020027	0,993	48	0,7
VASN	rs746313383	ms	Q6EMK4	564	A/T	0,413	0,022480	0,070	50	6,8

VEPH1	rs139246642	ms	Q14D04	512	N/K	-0,385	0,008380	0,009	69	62
VEZF1	rs745913326	ms	Q14119	190	N/S	-0,361	0,044866	0,011	38	1,5
VPS11	rs765997836	ms	A0A087WXL6	870	M/T	-0,681	0,018676	0,018	15	0
VPS13B	rs140061281	ms	Q7Z7G8-2	2547	S/G	0,972	0,041496	0,000	12	6,0
VPS13B	rs61754112	ms	Q7Z7G8-2	1452	L/F	1,137	0,014719	0,015	15	2,6
VPS13C	rs1567071824	ms	Q709C8	676	T/A	0,786	0,032903	0,585	16	0
**VPS13D**	rs757964463	ms	Q5THJ4	4228	R/W	-0,684	0,049243	0,990	14	0,1
**VPS13D**	rs758101809	ms	Q5THJ4	1604	Q/P	0,607	0,011056	0,140	24	5,3
VPS18	rs200156144	ms	Q9P253	835	R/Q	-0,359	0,039112	0,000	36	28
VPS41	rs199884913	ms	P49754	765	V/I	0,909	0,038978	0,838	14	5,3
VPS50	rs146809545	ms	Q96JG6	493	R/H	-0,740	0,042676	0,657	11	17
VPS53	rs752241629	ms	A0A7P0T874	626	W/R	0,606	0,045699	0,999	22	1,1
VRK3	rs747070456	ms	Q8IV63	413	P/T	0,577	0,014857	0,003	42	2,7
VWA1	rs747475277	ms	Q6PCB0	71	L/P	-0,539	0,033159	0,748	20	5,7
VWA5B1	rs777449498	ms	Q5TIE3-2	729	P/L	-0,436	0,012571	0,811	55	1,1
VWA8	rs141997392	ms	A3KMH1	625	P/L	0,601	0,031007	0,266	34	53
VWCE	rs529893923	ms	Q96DN2	465	V/I	0,693	0,026833	0,007	11	2,3
WASF3	rs375670514	ms	Q9UPY6	31	N/S	-1,365	0,019915	0,934	11	1,5
WASHC2C	10:45752594:A/C	ms	Q9Y4E1-7	337	E/A	0,535	0,028059	0,558	42	N/A
WASHC5	rs200456170	ms	Q12768	406	N/S	0,508	0,004476	0,007	47	7,6
WDCP	rs147244110	ms	Q9H6R7	502	P/L	-0,385	0,041475	0,052	51	247
WDR12	rs1253312677	ms	Q9GZL7	51	V/M	-0,668	0,006640	0,062	15	3,8
WDR19	rs199783864	ms	Q8NEZ3	310	Y/C	-0,775	0,005425	0,030	21	17
WDR27	rs537173344	ms	A0A3B3ITF5	413	V/I	-0,366	0,028184	0,000	52	143
WDR59	rs1567687673	ms	Q6PJI9	829	G/E	0,676	0,009492	0,026	16	0
WDR64	rs777137576	ms	A0A0C4DG52	524	L/I	-0,412	0,044196	0,000	50	0,4
WDR81	rs1423977378	ms	Q562E7	1706	L/R	0,719	0,006731	0,995	13	0,4
WDR83	rs774285413	ms	Q9BRX9	68	Y/C	-0,453	0,047944	0,894	32	1,9
**WDR90**	rs199805518	ms	Q96KV7	701	A/T	-0,551	0,007461	0,792	42	7,2
**WDR90**	rs766480010	ms	Q96KV7	918	R/C	0,408	0,023511	0,034	28	4,5
WNT10A	rs145641272	ms	Q9GZT5	172	R/W	0,527	0,031530	0,803	33	21
WSCD2	rs780830224	ms	Q2TBF2	234	R/Q	-0,531	0,017038	0,608	15	2,3
WWTR1	rs747164910	ms	Q9GZV5	140	R/K	0,354	0,014274	0,000	53	3,4
YTHDC2	rs760176014	ms	Q9H6S0	921	E/G	-0,429	0,020764	0,089	38	0
ZAN	rs762790622	ms	Q9Y493	333	N/S	-0,705	0,014791	0,007	28	0,1
**ZBTB38**	rs201503155	ms	Q8NAP3	588	N/I	1,113	0,017012	0,225	11	8,3
**ZBTB38**	rs75545796	ms	Q8NAP3	962	V/L	0,651	0,042930	0,000	16	125
ZBTB41	rs200742919	ms	Q5SVQ8	206	N/S	-0,385	0,043801	0,000	31	32
ZBTB48	rs141447787	ms	P10074	273	A/V	-1,162	0,000797	0,000	18	5,3
ZC3H11A	rs779675915	ms	O75152	249	V/I	-0,491	0,050451	0,001	27	1,5
**ZC3H13**	rs746706384	ms	A0A7I2V4I5	513	R/Q	0,616	0,040346	0,000	19	0,6
ZC3H18	rs200921944	ms	Q86VM9	58	P/L	0,814	0,041785	0,000	17	103
ZC3H7A	rs371372684	ms	Q8IWR0	320	P/S	0,456	0,020846	0,454	40	15
ZC3H7B	rs776780190	ms	Q9UGR2	369	R/Q	-0,656	0,023187	0,777	16	1,1
ZC3H8	rs1558927450	ms	Q8N5P1	135	G/V	0,988	0,007432	0,000	16	0,4
ZCCHC8	rs1400262196	ms	Q6NZY4	360	D/N	-0,677	0,040058	0,370	14	0,2
ZDBF2	rs370681691	ms	Q9HCK1	2047	R/W	-0,415	0,051414	0,668	37	4,9
ZDHHC12	rs201386601	ms	Q96GR4	194	A/P	0,620	0,027403	0,573	35	0,4
ZDHHC8	rs764513650	ms	Q9ULC8	532	R/C	0,835	0,048999	0,728	11	6,0
ZFC3H1	rs761708297	ms	O60293	1037	S/C	0,507	0,029768	0,852	26	0,4
ZFHX3	rs754898697	ms	Q15911	3187	T/M	-0,465	0,048444	0,000	43	0
ZFP28	rs142184982	ms	Q8NHY6	193	E/K	-0,483	0,018205	0,003	43	16
ZFP42	rs761262366	ms	Q96MM3	201	N/D	0,765	0,014942	0,003	11	0,7
ZFP64	rs765375562	ms	Q9NTW7-2	19	P/L	0,450	0,032128	0,000	55	5,3
ZFR2	rs199859071	ms	Q9UPR6	916	R/W	-0,535	0,046906	0,000	26	64
ZFYVE1	rs1433679547	ms	Q9HBF4	756	P/H	-1,317	0,011957	1,000	12	0
ZFYVE16	rs372767661	ms	Q7Z3T8	1144	L/P	-0,780	0,023639	0,997	20	5,3
**ZFYVE26**	rs371201801	ms	Q68DK2	1834	H/Y	0,919	0,050384	0,860	12	4,1
**ZFYVE26**	rs765803005	ms	Q68DK2	1714	M/V	-0,642	0,032764	0,001	29	0,8
ZGLP1	rs199597072	ms	A0A9L9PXT6	193	T/S	-0,530	0,012717	0,000	39	18
ZHX1	rs765258778	ms	Q9UKY1	26	S/T	0,706	0,018957	0,018	13	0
ZIC1	rs138004710	ms	Q15915	110	A/P	0,412	0,030365	0,999	54	34
ZKSCAN2	rs1567354833	stop	Q63HK3	185	R/*	-2,018	0,025704	1,000	12	0,1
**ZMIZ1**	rs1564594224	ms	Q9ULJ6	461	P/S	-0,548	0,032053	0,009	20	0
**ZMIZ1**	rs376587677	ms	Q9ULJ6	1050	P/A	-0,351	0,040608	0,000	62	1,3
**ZMIZ2**	rs750569363	ms	Q8NF64	631	N/S	-0,432	0,028222	0,998	53	0,7
**ZMIZ2**	rs751824063	ms	Q8NF64	415	S/N	-0,385	0,033384	0,000	44	0
**ZNF106**	rs371374573	ms	H3BSS6	1301	N/K	0,417	0,049275	0,152	21	1,9
**ZNF106**	rs756910453	ms	H3BSS6	461	P/S	0,932	0,007023	0,003	17	0,4
ZNF131	rs199798066	ms	P52739	536	T/S	0,394	0,033130	0,082	40	29
ZNF142	rs751858230	ms	A0A7P0N7C4	207	R/C	0,759	0,000590	0,824	33	0,5
ZNF16	rs200923173	ms	P17020	269	E/K	0,423	0,031115	0,001	44	2,6
ZNF189	rs200682925	ms	O75820	622	T/I	-1,004	0,000589	0,031	18	0,8
ZNF200	rs61731430	ms	P98182	22	P/L	0,872	0,008199	0,229	17	40
**ZNF213**	16:3141064:T/G	ms	O14771	366	V/G	-0,723	0,009433	0,999	26	N/A
ZNF232	rs200025811	ms	Q9UNY5	291	V/I	0,491	0,035656	0,005	21	6,4
ZNF235	rs757329633	ms	Q14590	140	P/A	0,459	0,046036	0,000	38	0,4
ZNF253	rs1568502375	ms	O75346	442	H/R	-0,677	0,018824	0,994	21	0,6
ZNF263	rs764198314	ms	O14978	139	V/M	-0,694	0,016307	0,000	32	0,7
ZNF268	rs543328816	ms	Q14587	732	S/F	-0,534	0,049956	0,955	14	17
ZNF273	rs768377540	ms	Q14593	448	H/Q	-0,870	0,010722	1,000	11	0,8
ZNF281	rs779663260	ms	Q9Y2X9	243	P/L	-0,492	0,018547	0,981	27	0,4
ZNF285	rs143574007	ms	Q96NJ3	507	I/T	-0,856	0,013758	0,000	10	75
**ZNF292**	rs201167963	ms	O60281	2224	K/N	-0,386	0,045925	0,918	38	7,6

ZNF292	rs752386780	ms	O60281	2048	K/N	0,352	0,044174	0,003	46	0,9
ZNF335	rs368892465	ms	Q9H4Z2	657	R/L	0,482	0,014256	0,185	38	1,5
ZNF354A	rs151334905	ms	O60765	15	T/M	-0,980	0,025856	0,999	15	19
ZNF395	rs1226932987	ms	Q9H8N7	2	A/V	1,555	0,009779	0,000	12	0
ZNF396	rs779377576	ms	Q96N95	148	M/V	-0,405	0,009534	0,000	38	0
ZNF408	rs769357381	ms	Q9H9D4	442	C/R	1,523	0,038991	0,999	12	1,1
ZNF423	rs201588114	ms	A0A7P0Q1F0	468	V/I	0,942	0,001758	0,103	18	36
ZNF430	rs139798103	ms	Q9H8G1	523	S/R	0,569	0,024384	0,003	26	25
ZNF430	rs148598454	ms	Q9H8G1	72	V/A	0,569	0,024384	0,054	26	15
ZNF436	rs773419541	ms	Q9C0F3	62	E/K	-0,856	0,044535	0,301	11	0,4
ZNF442	rs371108711	ms	Q9H7R0	309	R/Q	-0,779	0,023752	0,039	22	9,1
ZNF445	rs1222577044	ms	P59923	399	K/N	-0,771	0,047554	0,549	13	0,2
ZNF445	rs1359759859	ms	P59923	216	P/L	-0,972	0,020343	0,042	16	1,5
ZNF446	rs1041860970	ms	Q9NWS9	294	L/S	0,940	0,024878	0,000	19	1,1
ZNF462	rs1377404584	ms	Q96JM2	2121	L/F	-0,432	0,045357	0,316	20	0,2
ZNF469	rs772817384	ms	Q96JG9	1317	K/R	-0,387	0,022100	0,012	66	46
ZNF470	rs775542377	ms	Q6ECI4	243	L/I	-0,622	0,043099	0,969	18	1,5
ZNF493	rs1568384384	fs	Q6ZR52-2	564	K/X	0,382	0,041242	1,000	55	0
ZNF497	rs200614748	ms	Q6ZNH5	169	K/E	-0,422	0,043593	0,998	24	21
ZNF507	rs769948845	ms	Q8TCN5	477	N/K	-0,525	0,004293	0,996	53	9,4
ZNF525	rs572076035	ms	Q8N782	123	T/I	0,542	0,044167	0,492	22	134
ZNF526	rs750582519	ms	Q8TF50	151	S/L	0,698	0,007361	0,968	25	0,4
ZNF528	rs1397969661	ms	Q3MIS6	192	G/S	-0,758	0,027722	0,498	16	30
ZNF554	rs199517922	ms	Q86TJ5	208	V/L	-0,558	0,032338	0,007	16	28
ZNF562	rs200076265	ms	Q6V9R5	162	S/I	-0,462	0,046038	0,000	37	28
ZNF562	rs200884863	ms	Q6V9R5	163	I/L	-0,462	0,046038	0,000	37	16
ZNF571	rs1568340343	ms	Q7Z3V5	356	I/F	-0,890	0,023891	0,840	16	0
ZNF572	rs768125382	ms	Q7Z3I7	186	K/T	0,363	0,000755	0,998	151	0,2
ZNF587	rs1302318046	ms	Q96SQ5	83	R/G	0,383	0,049432	0,158	36	1,3
ZNF598	rs117633338	ms	H3BPG6	367	Q/R	0,470	0,002273	0,000	51	65
ZNF614	rs1568513712	ms	Q8N883	365	H/Y	0,554	0,026906	0,998	24	0
ZNF618	rs62620240	ms	Q5T7W0	586	A/V	-0,941	0,042038	0,005	16	77
ZNF638	rs137918800	ms	Q14966	897	L/F	1,588	0,031441	0,044	13	1,9
ZNF639	rs780577272	ms	Q9UID6	414	S/G	-0,471	0,034343	0,974	31	0,2
ZNF654	rs774298577	ms	Q8IZM8	727	I/V	-0,482	0,023273	0,006	38	1,9
ZNF66	rs761559630	ms	Q6ZN08	415	H/R	-0,467	0,031163	0,862	20	1,1
ZNF662	rs200562529	ms	Q6ZS27	19	P/L	-0,651	0,046831	0,722	20	6,7
ZNF678	rs1558158212	ms	Q5SXM1	335	H/Y	-0,432	0,049199	0,145	33	0,4
ZNF682	rs761895895	ms	O95780	411	I/V	-0,412	0,004990	0,000	85	3,4
ZNF707	rs201250823	ms	Q96C28	159	R/Q	-0,744	0,024146	0,000	15	15
ZNF714	rs200293321	ms	A0A087WU35	489	A/D	0,569	0,024378	0,022	26	13
ZNF714	rs372317502	ms	A0A087WU35	384	L/F	0,569	0,024378	0,862	26	21
ZNF726	rs1568380835	ms	A6NNF4-3	501	I/T	-0,762	0,028404	0,025	11	0
ZNF727	rs748962952	stop	A8MUV8	126	Q/*	0,415	0,020093	1,000	40	0
ZNF761	rs200379651	ms	Q86XN6	611	C/Y	0,997	0,006851	0,987	29	8,7
ZNF763	rs191167657	stop	Q0D2J5	162	Q/*	-0,507	0,008832	1,000	30	71
ZNF77	rs767350646	ms	Q15935	512	R/S	-0,964	0,008910	0,941	12	0,2
ZNF773	rs112107510	ms	Q6PK81	107	V/M	-0,684	0,014006	0,075	25	109
ZNF8	rs145477083	ms	P17098	234	C/R	-0,486	0,013676	0,462	37	134
ZNF8	rs758111446	ms	P17098	116	E/G	-0,690	0,047064	0,413	22	1,9
ZNF808	rs149957912	ms	Q8N4W9	494	R/H	0,437	0,042813	0,041	33	227
ZNF808	rs779325682	ms	Q8N4W9	807	A/V	0,548	0,027883	0,009	25	1,5
ZNF814	rs190903410	ms	B7Z6K7	210	G/R	0,565	0,025339	0,955	19	164
ZNF814	rs774093902	ms	B7Z6K7	596	R/C	-0,398	0,022114	0,000	46	2,6
ZNF821	rs139528518	ms	O75541	65	T/M	-0,885	0,024777	0,985	16	74
ZNF839	rs760906633	ms	A8K0R7-5	429	R/H	0,586	0,033405	0,391	27	1,3
ZNF841	rs372086918	ms	Q6ZN19-3	69	V/A	0,508	0,049189	0,302	17	7,2
ZNF862	rs200663618	ms	O60290	387	R/Q	-0,586	0,014298	0,007	19	20
ZNF891	rs1566336581	stop	A8MT65	97	Q/*	-0,895	0,014582	1,000	12	0
ZNF99	rs772978635	ms	A8MXY4	316	E/G	0,797	0,048267	0,015	16	0,8
ZNG1F	9:41182447:T/C	ms	Q4V339	120	I/V	0,442	0,030192	0,893	45	N/A
ZNG1F	rs1396385081	ms	Q4V339	157	G/R	0,892	0,035412	0,998	17	1,4
ZP1	rs1156861968	ms	P60852	395	S/L	-0,419	0,047372	0,013	24	0,8
ZSCAN1	rs147014137	ms	Q8NBB4	103	P/S	-0,776	0,017570	1,000	13	68
ZSWIM3	rs148673439	ms	Q96MP5	445	R/Q	-0,449	0,039612	0,001	40	5,7
ZSWIM4	rs1374935163	ms	K7ERJ6	442	G/D	-0,490	0,016327	0,052	40	2,3
ZSWIM4	rs774329836	ms	K7ERJ6	153	V/L	0,925	0,012076	0,135	16	0,8
ZSWIM8	rs373698947	ms	S4R410	847	R/H	-0,484	0,012556	0,201	53	5,1
ZZEF1	rs772878926	ms	O43149	1928	T/M	0,378	0,037554	0,089	44	3,4

PolyPhen-2 (dbNSFP version 3.3a) annotations based on HumVar classifier consist of score and categorical prediction. There are three possible predictions: Probably damaging (score≥0.909), possibly damaging (0.446≤score≤0.908), and benign (score≤0.445).
Compared to the original list (2,388 variants identified in GWAS [Ferkingstad, 2021; Table S1]), this list excludes variants found recurrently or across multiple independent cohorts of individuals with normal ACE levels, thus minimizing potential false-positive associations

Table S10. Missense mutations of *SORL1* in 84 carriers of Y215C mutation.

No.	rsID	Exon	Codons (NM_003105.6)	AA subs. (NP_003096.2)	Poly-Phen-2	CADD_PHRED	MAF (dbSNP) /100 000	Y215C carrier IDs
1	rs117260922	6/48	Gaa/Aaa	p.Glu270Lys	**0.990**	**27.2**	1505.2	HAC581, HWY602, NFV594
2	rs150609294	8/48	aAt/aCt	p.Asn371Thr	**0.487**	**24.7**	91.8	CNW069
3	rs2298813	11/48	Gcc/Acc	p.Ala528Thr	**0.541**	**28.6**	7255.7	ANO132, FWM710, JSO828, KJB939, MTU604, OTX780, RJZ347, TXM676, UWR783, XLK932, XTW087
4	rs1699107	22/48	Caa/Gaa	p.Gln1074Glu	0.001	15.8	1790.8	All
5	rs62617129	24/48	Atc/Gtc	p.Ile1116Val	0.001	0.6	508.1	1340, XLK932
6	rs146353234	31/48	Acc/Tcc	p.Thr1435Ser	0.003	12.9	50.6	RJZ347, YMB654
7	rs1792120	44/48	Gtt/Att	p.Val1967Ile	0.003	4.9	1729.6	All
8	rs140327834	46/48	gAt/gTt	p.Asp2065Val	**0.998**	**26.6**	220.6	715, MZL910, OTS421
9	rs142884576	47/48	aCg/aTg	p.Thr2134Met	**0.825**	**23.4**	0	UVC754

Sequences of 84 patients with Y215C *ACE* mutation (Fig. 1B) were searched for the variants of *SORL1* mutations. We found 9 mutations of *SORL1*.
